# Association of Race/Ethnicity, Gender, and Socioeconomic Status With Sodium-Glucose Cotransporter 2 Inhibitor Use Among Patients With Diabetes in the US

**DOI:** 10.1001/jamanetworkopen.2021.6139

**Published:** 2021-04-15

**Authors:** Lauren A. Eberly, Lin Yang, Nwamaka D. Eneanya, Utibe Essien, Howard Julien, Ashwin S. Nathan, Sameed Ahmed M. Khatana, Elias J. Dayoub, Alexander C. Fanaroff, Jay Giri, Peter W. Groeneveld, Srinath Adusumalli

**Affiliations:** 1 Cardiovascular Division, Perelman School of Medicine at the University of Pennsylvania, Philadelphia; 2 Center for Cardiovascular Outcomes, Quality, and Evaluative Research, University of Pennsylvania, Philadelphia; 3 Penn Cardiovascular Center for Health Equity and Social Justice, University of Pennsylvania, Philadelphia; 4 Leonard Davis Institute of Health Economics at the University of Pennsylvania, Philadelphia; 5 Renal-Electrolyte and Hypertension Division, Perelman School of Medicine at the University of Pennsylvania, Philadelphia; 6 Division of General Internal Medicine, University of Pittsburgh School of Medicine, Pittsburgh, Pennsylvania; 7 Center for Health Equity Research and Promotion, VA Pittsburgh Healthcare System, Pittsburgh, Pennsylvania; 8 Corporal Michael J. Crescenz Veterans Affairs Medical Center, Philadelphia, Pennsylvania; 9 Division of General Internal Medicine, Perelman School of Medicine at the University of Pennsylvania, Philadelphia

## Abstract

**Importance:**

Sodium-glucose cotransporter 2 (SGLT2) inhibitors significantly reduce deaths from cardiovascular conditions, hospitalizations for heart failure, and progression of kidney disease among patients with type 2 diabetes. Black individuals have a disproportionate burden of cardiovascular and chronic kidney disease (CKD). Adoption of novel therapeutics has been slower among Black and female patients and among patients with low socioeconomic status than among White or male patients or patients with higher socioeconomic status.

**Objective:**

To assess whether inequities based on race/ethnicity, gender, and socioeconomic status exist in SGLT2 inhibitor use among patients with type 2 diabetes in the US.

**Design, Setting, and Participants:**

This retrospective cohort study of commercially insured patients in the US was performed from October 1, 2015, to June 30, 2019, using the Optum Clinformatics Data Mart. Adult patients with a diagnosis of type 2 diabetes, including those with heart failure with reduced ejection fraction (HFrEF), atherosclerotic cardiovascular disease (ASCVD), or CKD, were evaluated in the analysis.

**Main Outcomes and Measures:**

Prescription of an SGLT2 inhibitor. Multivariable logistic regression models were used to assess the association of race/ethnicity, gender, and socioeconomic status with SGLT2 inhibitor use.

**Results:**

Of 934 737 patients with type 2 diabetes (mean [SD] age, 65.4 [12.9] years; 50.7% female; 57.6% White), 81 007 (8.7%) were treated with an SGLT2 inhibitor during the study period. Between 2015 and 2019, the percentage of patients with type 2 diabetes treated with an SGLT2 inhibitor increased from 3.8% to 11.9%. Among patients with type 2 diabetes and cardiovascular or kidney disease, the rate of SGLT2 inhibitor use increased but was lower than that among all patients with type 2 diabetes (HFrEF: 1.9% to 7.6%; ASCVD: 3.0% to 9.8%; CKD: 2.1% to 7.5%). In multivariable analyses, Black race (adjusted odds ratio [aOR], 0.83; 95% CI, 0.81-0.85), Asian race (aOR, 0.94; 95% CI, 0.90-0.98), and female gender (aOR, 0.84; 95% CI, 0.82-0.85) were associated with lower rates of SGLT2 inhibitor use, whereas higher median household income (≥$100 000: aOR, 1.08 [95% CI, 1.05-1.10]; $50 000-$99 999: aOR, 1.05 [95% CI, 1.03-1.07] vs <$50 000) was associated with a higher rate of SGLT2 inhibitor use. These results were similar among patients with HFrEF, ASCVD, and CKD.

**Conclusions and Relevance:**

In this cohort study, use of an SGLT2 inhibitor treatment increased among patients with type 2 diabetes from 2015 to 2019 but remained low, particularly among patients with HFrEF, CKD, and ASCVD. Black and female patients and patients with low socioeconomic status were less likely to receive an SGLT2 inhibitor, suggesting that interventions to ensure more equitable use are essential to prevent worsening of well-documented disparities in cardiovascular and kidney outcomes in the US.

## Key Points

**Question:**

Are race/ethnicity, gender, and socioeconomic status associated with use of sodium-glucose cotransporter 2 (SGLT2) inhibitors among patients with type 2 diabetes in the US?

**Findings:**

In a 5-year cohort study of 934 737 commercially insured US patients with type 2 diabetes, the frequency of SGLT2 inhibitor use increased, but use remained low even among patients with heart failure, kidney disease, and cardiovascular disease. Black race, female gender, and lower household income were associated with lower rates of SGLT2 inhibitor use.

**Meaning:**

In this study, racial/ethnic, gender, and socioeconomic inequities were present in access to SGLT2 inhibitor treatment, which if unaddressed, may widen disparities in kidney and cardiovascular outcomes in the US.

## Teaser

This cohort study evaluates whether inequities in race/ethnicity, gender, and socioeconomic status exist in sodium-glucose cotransporter 2 (SGLT2) inhibitor use among US patients with type 2 diabetes.

## Introduction

Diabetes is significantly associated with development of cardiovascular and kidney disease in the US.^1,2^ Sodium-glucose cotransporter 2 (SGLT2) inhibitors decrease kidney glucose reabsorption and thereby increase urinary glucose excretion and improve in blood glucose levels.^3^ In addition to improved glycemic control, since publication of the Empagliflozin Cardiovascular Outcome Event Trial in Type 2 Diabetes Mellitus (EMPA-REG) in 2015,^4^ studies have demonstrated substantial classwide cardioprotective and kidney-protective effect of this medication.^5^ Among patients with type 2 diabetes at high risk for cardiovascular events, SGLT2 inhibitor use has been shown to significantly reduce death from cardiovascular causes and to lower the risk of hospitalization for heart failure and progression of kidney disease.^4,6^ Rates of worsening heart failure or cardiovascular death are lower among patients with heart failure who are treated with dapagliflozin and empagliflozin regardless of diabetes diagnosis.^7,8^ Treatment with dapagliflozin reduces adverse kidney events and decreases mortality among patients with chronic kidney disease (CKD).^9^ Given the demonstrated benefits of SGLT2 inhibitors, the updated American Diabetes Association guidelines^10^ and the American College of Cardiology expert consensus statement^11^ now recommend their use for diabetic patients who have or are at high risk for cardiovascular disease, CKD, or heart failure regardless of current glycemic control.

Black patients have a disproportionately higher burden of cardiovascular and advanced kidney disease.^12,13,14,15,16,17,18,19,20^ Cardiovascular mortality rates remain highest among Black patients in the US, and racial disparities in the prevalence of major risk factors for coronary heart disease, such as type 2 diabetes, are widening.^21,22^ Inequitable care delivery based on race is pervasive.^23^ In addition, inequities in care based on gender and socioeconomic status have been observed.^24^ Historically, there has been decreased adoption of novel therapies among Black patients as well as among female patients and those with low socioeconomic status.^25,26,27^ The objective of this study was to evaluate trends in SGLT2 inhibitor prescription by race/ethnicity, gender, and socioeconomic status between 2015 (the year of publication of EMPA-REG) and 2019. We also assessed the association of race/ethnicity, gender, and socioeconomic status with SGLT2 inhibitor use among commercially insured patients with type 2 diabetes in the US, including those with heart failure with reduced ejection fraction (HFrEF), CKD, and atherosclerotic cardiovascular disease (ASCVD); individuals with these comorbidities were evaluated because of the demonstrated benefit of SGLT2 inhibitor use in these particular subgroups.

## Methods

### Study Data

This cohort study used data from the Optum Clinformatics Data Mart database, a large administrative private payer claims database of recipients of commercial health insurance and Medicare Advantage health plans, from October 1, 2015, to June 30, 2019. This database consists of inpatient, outpatient, and pharmacy claims of more than 17 million patients annually from all 50 states. Data are updated every 6 to 12 months and are available from January 2004 through June 2019. The University of Pennsylvania institutional review board determined that this research was exempt from the regulatory requirements of the federal Common Rule because no protected health information was used. This study followed the Strengthening the Reporting of Observational Studies in Epidemiology (STROBE) reporting guideline.

Patient demographic variables, such as age, gender, and race/ethnicity, were available for each member at enrollment. Socioeconomic data, including median household income, were available through zip code–linked enrollment data from the US Census Bureau. Mean number of outpatient cardiology visits and endocrinology visits per 12 months after cohort entry until the end of available data (June 30, 2019) were determined based on patient visits with a cardiology or endocrinology provider with *Current Procedural Terminology* codes 99201-99205 or 99211-99215 noted in the record. All prescription claims for empagliflozin, dapagliflozin, and canagliflozin (or combination medications) were extracted.

### Study Cohort

We identified adult patients (age, ≥18 years) with a diagnosis of type 2 diabetes based on *International Statistical Classification of Diseases, Tenth Revision, Clinical Modification* (*ICD-10-CM*) codes E11.0, E11.1, or E11.9 between October 2015 and December 2018 to allow for 6 months of continuous enrollment and prescription of therapy after diagnosis given that data were available through June 2019. Each patient was required to have a diagnosis of type 2 diabetes coded on at least 2 occasions on separate dates either as an inpatient or outpatient. Patients entered the cohort on the date of second diagnostic code for type 2 diabetes and were then evaluated for a prescription claim filled for empagliflozin, dapagliflozin, canagliflozin, or any combination medication containing 1 of these SGLT2 inhibitor agents through June 2019. Therefore, the study period for each patient was from the second coded diagnosis of type 2 diabetes to June 2019 (end of available data).

Comorbidities were evaluated from the earliest date of available data to the date of cohort entry. Patients were excluded if they had CKD stage IV or V or end-stage kidney disease (*ICD-10-CM* codes N184, N185, N186, or N189; *International Classification of Diseases, Ninth Revision* codes: 5854, 5855, 5856, or 5859) any time before cohort entry because SGLT2 inhibitors are contraindicated in this patient population. Patients were also excluded if they did not have continuous insurance enrollment for at least 1 year before and at least 6 months after study entry, so that comorbidities, clinical data, and prescription claims could be accurately obtained for all patients. In addition, patients without any pharmacy claims for medication for 1 year before the study period were excluded to ensure that patients’ medication use was being accurately captured in our data.

### Subgroup Analysis

We performed a subgroup analysis of patients with a diagnosis of HFrEF (*International Statistical Classification of Diseases and Related Health Problems, Tenth Revision* [*ICD-10*] codes I50.2, I50.21, I50.22, I50.23, I50.4, I50.41, I40.42, and I50.43), those with CKD (stages I, II, and III: *ICD-10* codes I12.0, I13.1, N18.x, NI9.x, N25.0, Z49.0, Z49.2, Z94.0, and Z99.2), and those with ASCVD based on *ICD-10* codes (eTable 1 in the Supplement). Because rates of lifestyle modification as the sole therapy for type 2 diabetes or adherence to medication therapy may differ between patient groups, we also performed subgroup analysis of patients receiving metformin therapy (prescription claim filled for metformin within 12 months after cohort entry).

### Statistical Analysis

We compared patients who received and did not receive SGLT2 inhibitor treatment over the course of the study period. For each group, summary statistics for patient characteristics are presented as medians with interquartile ranges (IQRs) or means with SDs for continuous data and as total numbers and percentages for categorical data. Continuous variables were compared using the Student *t* test, and categorical variables were compared using the χ^2^ test.

To assess the association of race/ethnicity with the use of an SGLT2 inhibitor, we estimated multivariable logistic regression models with use of an SGLT2 inhibitor as the dependent variable and independent variables that included age, gender, race/ethnicity (Black, Latinx, White, or Asian), region of residence, zip code–linked household income, health insurance subset (commercial only or Medicare Advantage, which provides Medicare benefits through United Healthcare), hyperlipidemia, coronary artery disease, cerebrovascular disease, CKD, hypertension, obesity, peripheral vascular disease, HFrEF, heart failure with preserved ejection fraction (HFpEF), number of Elixhauser comorbidities,^28^ number of visits to a cardiologist per 12 months, number of visits to an endocrinologist per 12 months, insulin use, and metformin use.

For subgroup analyses, we included the same covariates for analysis of patients with ASCVD. To assess the association of race/ethnicity with the use of SGLT2 inhibitor in the subgroup of patients with HFrEF, we used the aforementioned multivariable logistic regression model but also included number of hospitalizations for treatment of heart failure in the prior 12 months and excluded HFpEF as a covariate. To assess the association of race/ethnicity with the use of SGLT2 inhibitor among patients with CKD, we used the aforementioned primary multivariable logistic regression model but also included stage of CKD (stage I vs II vs III) as a categorical covariate. For the subgroup of patients receiving metformin therapy, we used the aforementioned primary multivariable logistic regression model but excluded metformin as a covariate.

Estimated adjusted odds ratios (aORs) are reported with 95% CIs. Statistical analyses were performed using SAS, version 9.4 (SAS Institute Inc). All statistical testing was 2-tailed, with *P* < .05 designated as statistically significant. Adjustments for multiple comparisons were not made; thus, secondary and subgroup analyses should be considered exploratory.

## Results

**Figure 1.**
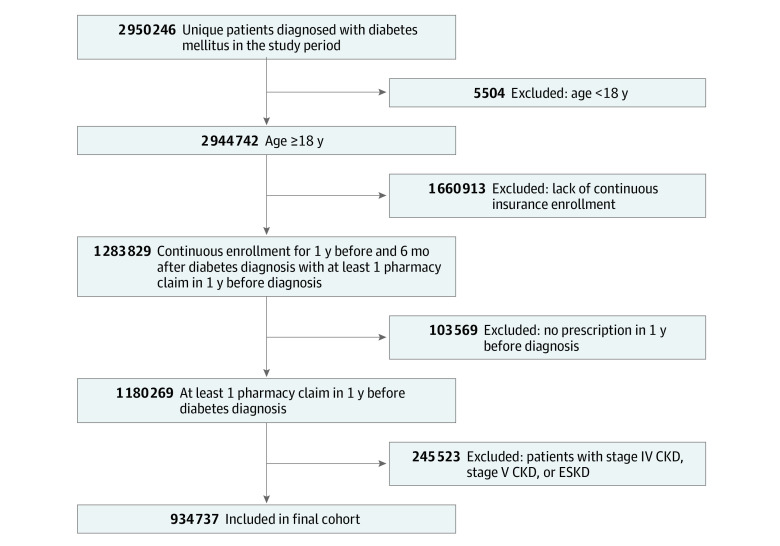
Selection of the Study Population CKD, chronic kidney disease; ESKD, end-stage kidney disease.

A total of 934 737 patients met the inclusion criteria (mean [SD] age, 65.4 years [12.9] years; 50.7% female; 57.6% White) (Figure 1). Overall, 81 007 (8.7%) received SGLT2 inhibitor treatment during the study period, and 853 730 (91.3%) did not. Baseline demographic, socioeconomic, and clinical differences between those who were prescribed an SGLT2 inhibitor and those who were not are summarized in Table 1.

**Table 1.** Baseline Characteristics of Patients With Type 2 Diabetes With and Without Filled Prescription for Sodium-Glucose Cotransporter 2 Inhibitors

Variable	SGLT2 inhibitor treatment^a^		*P* value
	No (n = 853 730)	Yes (n = 81 007)	
Age, median (IQR), y	68 (58-75)	58 (50-67)	<.001
Female	439 542 (51.5)	34 420 (42.5)	<.001
Male	414 105 (48.5)	46 587 (57.5)	
Race/ethnicity			
Asian	40 663 (4.8)	3592 (4.4)	<.001
Black	101 350 (11.9)	8765 (10.8)	
Latinx	127 805 (15.0)	13 082 (16.1)	
White	489 074 (57.3)	48 958 (60.4)	
Unknown	94 838 (11.1)	6610 (8.2)	
Region			
Midwest	174 282 (20.4)	17 607 (21.7)	<.001
Northeast	112 862 (13.2)	7378 (9.1)	
South	371 113 (43.5)	41 781 (51.6)	
West	193 496 (22.7)	14 124 (17.4)	
Unknown	1977 (0.2)	117 (0.1)	
Zip code–linked household income, $			
<50 000	257 661 (30.2)	20 109 (24.8)	<.001
50 000-99 999	256 101 (30.0)	25 008 (30.9)	
≥100 000	157 931 (18.5)	20 783 (25.7)	
Unknown	182 037 (21.3)	15 107 (18.6)	
Insurance type			
Commercial	305 871 (35.8)	55 439 (68.4)	<.001
Medicare Advantage	547 859 (64.2)	25 568 (31.6)	
Insurance plan type			
Exclusive provider organization	39 546 (4.6)	7769 (9.6)	<.001
Health maintenance organization	240 970 (28.2)	14 896 (18.4)	
Indemnity	8604 (1.0)	668 (0.8)	
Point of service	300 070 (35.1)	14 731 (18.2)	
Preferred provider organization	214 162 (25.1)	40 488 (50.0)	
Other	50 378 (5.9)	2455 (3.0)	
Comorbidities^b^			
Dyslipidemia	731 363 (85.7)	71 106 (87.8)	<.001
Myocardial infarction	67 452 (7.9)	4950 (6.1)	<.001
Cerebrovascular disease	159 292 (18.7)	9580 (11.8)	<.001
Chronic kidney disease	87 515 (10.3)	4970 (6.1)	<.001
Obesity	272 134 (31.9)	32 561 (40.2)	<.001
Hypertension	703 155 (82.4)	65 877 (81.3)	<.001
Peripheral vascular disease	152 744 (17.9)	8233 (10.2)	<.001
HFrEF	24 802 (2.9)	1252 (1.5)	<.001
HFpEF	24 647 (2.9)	1009 (1.2)	<.001
Elixhauser comorbidities, No.^b^			
0-1	198 783 (23.3)	23 816 (29.4)	<.001
2-3	325 784 (38.2)	34 115 (42.1)	
4-6	235 725 (27.6)	18 509 (22.8)	
≥7	93 438 (10.9)	4567 (5.6)	
Elixhauser comorbidities, mean (SD), No.^b^	3.4 (2.4)	2.8 (2.0)	<.001
Visits to an endocrinology specialist, No. per 12 mo			
0	779 734 (91.3)	61 170 (75.5)	<.001
1	32 792 (3.8)	7216 (8.9)	
>1	41 204 (4.8)	12 621 (15.6)	
Visits to a cardiology specialist, No. per 12 mo			
0	639 287 (74.9)	60 851 (75.1)	<.001
1	103 383 (12.1)	11 019 (13.6)	
>1	111 060 (13.0)	9137 (11.3)	
Medications			
Metformin	437 605 (51.3)	53 229 (65.7)	<.001
Insulin	117 310 (13.7)	21 081 (26.0)	<.001

Abbreviations: HFpEF, heart failure with preserved ejection fraction; HFrEF, heart failure with reduced ejection fraction; IQR, interquartile range; SGLT2, sodium-glucose cotransporter 2.

^a^ Data are presented as number (percentage) of individuals unless otherwise indicated.

^b^ From beginning of available data to date of cohort study entry.

**Figure 2.**
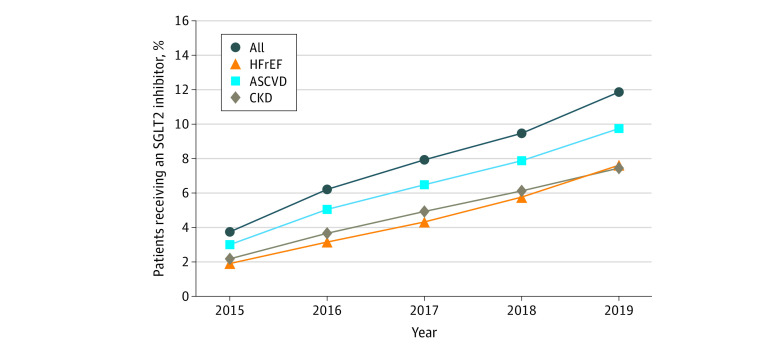
Rates of Treatment With Sodium-Glucose Cotransporter 2 Inhibitor in the Cohort Over Time ASCVD, atherosclerotic cardiovascular disease; CKD, chronic kidney disease; HFrEF, heart failure with reduced ejection fraction.

**Figure 3.**
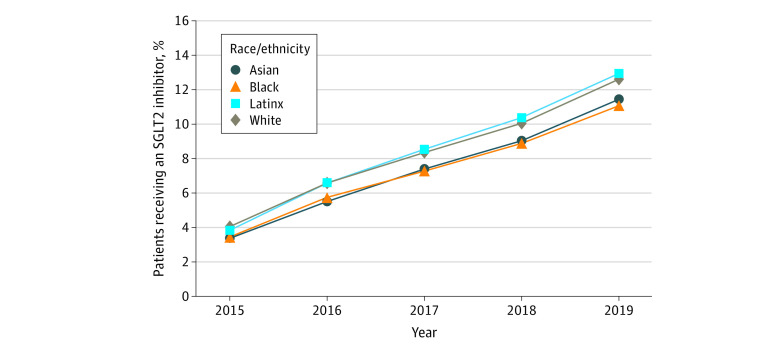
Rates of Treatment With Sodium-Glucose Cotransporter 2 Inhibitor by Race/Ethnicity in the Cohort Over Time

The rate of SGLT2 inhibitor use increased from 3.8% in 2015 to 11.9% in 2019 in the entire cohort (Figure 2). The rate of SGLT2 inhibitor use increased from 3.4% to 11.4% among Asian patients, 3.4% to 11.1% among Black patients, 3.8% to 13.0% among Latinx patients, and 4.0% to 12.6% among White patients (Figure 3). Among those with HFrEF (26 054), the use of SGLT2 inhibitors increased from 1.9% in 2015 to 7.6% in 2019. For those with ASCVD (594 058), the rate of SGLT2 inhibitor use increased from 3.0% to 9.8%, and the rate among patients with CKD (92 485) increased from 2.1% in 2015 to 7.5% in 2019 (Figure 2).

In multivariable analyses (Table 2), Black race (aOR, 0.83; 95% CI, 0.81-0.85; *P* < .001) and Asian race (aOR, 0.94; 95% CI, 0.90-0.98; *P* < .001) were independently associated with lower rates of SGLT2 inhibitor use compared with White race. Female gender was also independently associated with a lower rate of SGLT2 inhibitor use (aOR, 0.84; 95% CI 0.82-0.85; *P* < .001). Higher median household income was associated with a higher rate of SGLT2 inhibitor use, with an aOR of 1.08 (95% CI, 1.05-1.10; *P* < .001) for income of greater than or equal to $100 000 and an aOR of 1.05 (95% CI, 1.03-1.07; *P* < .001) for income ranging from $50 000 to $99 999 compared with income of less than $50 000. Heart failure with reduced ejection fraction (aOR, 0.85; 95% CI, 0.79-0.91; *P* < .001) and HFpEF (aOR, 0.83; 95% CI, 0.77-0.89; *P* < .001) were independently associated with a lower rate of SGLT2 inhibitor use. Chronic kidney disease was not associated with SGLT2 inhibitor use (aOR, 1.03; 95% CI, 0.99-1.07; *P* = .14). A greater number of Elixhauser comorbidities was associated with a lower rate of SGLT2 inhibitor use (aOR, 0.90; 95% CI, 0.89-0.90; *P* < .001). Having more visits to a cardiologist per 12 months (aOR, 1.19 [95% CI, 1.16-1.22] for 1 visit; aOR, 1.15 [95% CI, 1.12-1.18] for >1 visit) and having more visits to an endocrinologist per 12 months (aOR, 2.06 [95% CI, 1.99-2.12] for 1 visit; aOR, 2.84 [95% CI, 2.76-2.92] for >1 visit) were also independently associated with increased SGLT2 inhibitor use.

**Table 2.** Factors Associated With SGLT2 Inhibitor Use Among All Patients in the Multivariable Analysis

Characteristic	Adjusted OR (95% CI)	*P* value
Age	0.98 (0.97-0.98)	<.001
Female	0.84 (0.82-0.85)	<.001
Race/ethnicity		
White	1 [Reference]	NA
Asian	0.94 (0.90-0.98)	.002
Black	0.83 (0.81-0.85)	<.001
Latinx	1.03 (1.01-1.06)	.009
Region of residence		
West	1 [Reference]	NA
Midwest	1.06 (1.03-1.09)	<.001
Northeast	0.93 (0.90-0.97)	<.001
South	1.33 (1.29-1.36)	<.001
Zip code–linked household median income, $		
<500 000	1 [Reference]	NA
≥100 000	1.08 (1.05-1.10)	<.001
50 000-99 999	1.05 (1.03-1.07)	<.001
Commercial insurance	2.17 (2.12-2.22)	<.001
Medicare Advantage	1 [Reference]	NA
Comorbidities		
Dyslipidemia	1.61 (1.56-1.65)	<.001
Myocardial infarction	1.00 (0.97-1.04)	.84
Cerebrovascular disease	0.98 (0.95-1.00)	.09
Chronic kidney disease	1.03 (0.99-1.07)	.14
Obesity	1.33 (1.30-1.36)	<.001
Hypertension	1.49 (1.45-1.53)	<.001
Peripheral vascular disease	1.04 (1.01-1.07)	.03
HFrEF	0.85 (0.79-0.91)	<.001
HFpEF	0.83 (0.77-0.89)	<.001
No. of Elixhauser comorbidities	0.90 (0.89-0.90)	<.001
Visits to an endocrinology specialist, No. per 12 mo		
0	1 [Reference]	NA
1	2.06 (1.99-2.12)	<.001
>1	2.84 (2.76-2.92)	<.001
Visits to a cardiology visits, No. per 12 mo		
0	1 [Reference]	NA
1	1.19 (1.16-1.22)	<.001
>1	1.15 (1.12-1.18)	<.001
Metformin use	1.55 (1.52-1.58)	<.001
Insulin use	1.57 (1.53-1.60)	<.001

Abbreviations: HFpEF, heart failure with preserved ejection fraction; HFrEF, heart failure with reduced ejection fraction; NA, not applicable; OR, odds ratio; SGLT2, sodium-glucose cotransporter 2.

Independent factors associated with SGLT2 inhibitor use treatment among the subgroups of patients with HFrEF, ASCVD, and CKD in multivariable analyses are shown in eTables 2 to 4 in the Supplement, respectively. Results were similar in subgroup analyses. Female gender was independently associated with a lower rate of SGLT2 inhibitor use among patients with type diabetes and a diagnosis of HFrEF (aOR, 0.83; 95% CI, 0.75-0.92), ASCVD (aOR, 0.83; 95% CI, 0.81-0.85), or CKD (aOR, 0.85; 95% CI, 0.81-0.90). Higher median household income was associated with a higher rate of SGLT2 inhibitor use across all subgroups (HFrEF: aOR, 1.21 [95% CI, 1.08-1.34] for income of $50 000-$99 999; aOR, 1.26 [95% CI, 1.11-1.44] for income ≥$100 000 vs <$50 000; ASCVD: aOR, 1.05 [95% CI, 1.02-1.08] for income of $50 000-$99 999; aOR, 1.08 [95% CI, 1.05-1.12] for income ≥$100 000 vs <$50 000; CKD: aOR, 1.15 [95% CI, 1.07-1.24] for income ≥$100 000 vs <$50 000). Among patients with ASCVD or CKD, Black race was associated with a lower rate of SGLT2 inhibitor use (aOR, 0.89 [95% CI, 0.82-0.97] for CKD; aOR, 0.84 [95% CI, 0.81-0.87] for ASCVD).

In the subgroup analysis of patients receiving metformin therapy, Black race (aOR, 0.79; 95% CI, 0.76-0.82), Latinx ethnicity (aOR, 0.97; 95% CI, 0.94-0.99), Asian race (aOR, 0.84; 95% CI, 0.80-0.88), and female gender (aOR, 0.82; 95% CI, 0.80-0.84) were associated with a lower rate of SGLT2 inhibitor use. Higher median household income was associated with a higher rate of SGLT2 inhibitor use (aOR, 1.08 [95% CI, 1.05-1.10] for income of $50 000-$99 999; aOR, 1.13 [95% CI, 1.10-1.17] for income ≥$100 000 vs <$50 000) (eTable 5 in the Supplement).

## Discussion

Between 2015 and 2019, the rate of SGLT2 inhibitor use increased for the management of type 2 diabetes, yet overall use remained low, including for patients with HFrEF, ASCVD, and CKD. The low rate of SGLT2 inhibitor use among patients with HFrEF was consistent with recent evidence from an outpatient heart failure registry.^29^ In the commercially insured population in the present study, there were racial/ethnic, gender, and socioeconomic inequities in receipt of SGLT2 inhibitor therapy. Black race and female gender were associated with a lower rate of SGLT2 inhibitor use, whereas higher household income was independently associated with a higher rate of SGLT2 inhibitor use. Most of these inequities were present among patients with HFrEF, ASCVD, and CKD in addition to type 2 diabetes. To our knowledge, this is the first study to investigate whether there is inequitable access to SGLT2 inhibitor treatment among commercially insured patients with type 2 diabetes in the US.

Barriers to adoption of novel therapeutic agents include decreased access to quality diabetes care and to specialists familiar with the benefits of SGLT2 inhibitor use, structural racism, provider bias that certain groups of patients may be less likely to be adherent to treatment with an expensive agent, and prescription abandonment owing to economic barriers.^30,31,32,33,34,35,36,37,38^ Despite a well-demonstrated benefit of the SGLT2 inhibitor class of medications and a higher burden of adverse sequelae of type 2 diabetes among Black patients,^21^ Black race was independently associated with a lower rate of SGLT2 inhibitor use, which was also a finding in subgroup analyses of patients with ASCVD and CKD. These results are consistent with those of several prior studies^25,26,27^ that have shown decreased use of novel therapeutics among Black patients. Although this finding may reflect differences in specialist consultation and decreased access to providers familiar with the clinical benefits of SLGT2 inhibitor treatment,^30,31,32,33,34,35,36,37,38^ we found that lower rates of SGLT2 inhibitor prescription persisted even after adjustment for visits to cardiology and endocrinology specialists. This result suggests that racism and bias in care delivery may contribute to the findings of this study as well.

Black patients have a disproportionate burden of cardiovascular and kidney disease and experience worse cardiovascular outcomes than White patients.^8,9,12,13,14,15,16,17,18^ Inequitable use of novel pharmacologic agents such as SGLT2 inhibitor agents, a medication class with well-documented cardioprotective and kidney-protective benefit,^4,6,7,8,9^ may contribute to the well-documented racial disparities in cardiovascular and kidney outcomes^12,13,14,15,16,17,18,19,20^; barriers to accessing therapy with clinical benefit may contribute to the widening of disparities in cardiovascular outcomes in the US.^21,22^ Given the increasing amount of evidence supporting the broad clinical benefit of SGLT2 inhibitor treatment,^4,6,7,8,9^ further investigation of barriers to accessing this therapy and implementation of strategies to address structural racism and ensure more equitable use of this therapy among Black patients are essential.

Although inequities were not present in SGLT2 inhibitor use among Latinx patients overall, Asian race was associated with a lower rate of SGLT2 inhibitor use. Barriers to accessing care among certain Asian-American subgroups in the US have been demonstrated.^39^ Furthermore, for Asian patients who have access to care, provider interactions are more frequently characterized by lower rates of patient-centered care and input regarding treatment decisions, which may explain these results.^40^

In addition to racial inequities in SGLT2 inhibitor use, we discovered other structural inequities based on gender and socioeconomic status that can be addressed intersectionally.^41^ Among patients with type 2 diabetes, females were less likely to be prescribed an SGLT2 inhibitor, even among those with HFrEF, ASCVD, and CKD. This is consistent with findings from prior studies^42,43,44,45,46^ of female patients in which guideline-directed therapies were initially adopted more slowly and underused among female patients. Poorer provider communication may also contribute to gender inequity.^47^ Although SGLT2 inhibitor use was not incorporated into American Diabetes Association guidelines until 2019,^48^ these results highlight the need for strategies, such as decision pathways, for new guidelines in order to lessen inequities by reducing subjectivity in making the decision to initiate therapy.^49^

We also found inequitable use of SGLT2 inhibitor therapy based on socioeconomic status even though this study was conducted in a commercially insured population. Those with a median household income of greater than or equal to $100 000 were 8% more likely and those with a median income ranging from $50 000 to $99 999 were 5% more likely to receive SGLT2 inhibitor therapy than were those with a median income of less than $50 000. Affordability and out-of-pocket costs of these agents may be prohibitive, leading to prescription abandonment, especially compared with the cost of older, more traditionally used therapies, such as sulfonylureas, which can be obtained as part of the $4 generic drug program at certain pharmacies.^50,51^ Despite SGLT2 inhibitor coverage being relatively high in 2019 for Medicare beneficiaries, median retail prices for a 30-day supply were $300 (IQR, $285-$303), and estimated annual out-of-pocket costs ranged from $1097 to $1211; thus, these medications are unaffordable for many patients who would receive clinical benefit.^52^ Commercial insurance (vs Medicare Advantage) was one of the factors most strongly associated with SGLT2 inhibitor prescription in this study, suggesting that sufficient coverage and medication cost may be associated with different rates of use. Given the demonstrated cost-effectiveness of these medications,^53^ our results suggest that out-of-pocket costs should be minimized. In addition, provider biases about the ability of patients with lower socioeconomic status to afford and adhere to treatment with an SGLT2 inhibitor may contribute to differential prescribing patterns.^54^

We also found that having a visit with an endocrinologist in the past 12 months was one of the strongest factors positively associated with SGLT2 inhibitor use in the study cohort. The demonstrated clinical benefit, dating from 2015, may not yet be common knowledge among many nonspecialist providers who treat patients with diabetes. Strategies to increase the comfort of all providers with prescribing SGLT2 inhibitor therapy will be essential to address inequitable use and ensure improved cardiovascular and kidney outcomes for all patients with type 2 diabetes.

### Limitations

This study has limitations. Because we used an administrative, insurance claims–based database, we were unable to differentiate between prescriptions offered (biases in treatment strategy) and prescriptions filled (barriers to therapy completion). We were also unable to fully understand the detailed decision-making, clinical context, and physician and patient preference regarding each patient’s unique treatment plan. The database we used did not allow us to evaluate the type of practice or health care professional prescribing therapy, thus limiting understanding of how differences in access to specialty care contributed to our findings. Furthermore, we were unable to evaluate for severity or control of type 2 diabetes, which may also have affected treatment decisions. Some data were missing at baseline, including data on median household income, and more granular individual-level socioeconomic data were unavailable. In addition, zip code– or county-level covariates, such as supply of physicians and specialists, rurality, and other markers of poverty, were not available in this data set, which may have affected the findings. Although the Optum database includes information on patients from all 50 states, the greatest concentration of coverage was in the South and Midwest, which may limit generalizability. Although these findings may not be generalizable to other payor groups, the observed differences may be even greater among those with traditional Medicare or Medicaid or those without health care insurance.

## Conclusions

In this cohort study of a large, diverse, commercially insured population, rates of SGLT2 inhibitor use for the treatment of patients with type 2 diabetes in the US increased from 2015 to 2019, yet rates remained low even among patients with type 2 diabetes and HFrEF, ASCVD, and CKD. Black race, female gender, and lower zip code–linked household income were independently associated with lower rates of SGLT2 inhibitor use, with inequitable use also present among those with HFrEF, ASCVD, and CKD. These findings suggest that racial, gender, and socioeconomic inequities are present in access to SGLT2 inhibitor treatment. Further studies to better understand barriers to these therapies and ensure equitable access are essential.

## Notes

Supplement. **eTable 1.** Included ICD codes for Atherosclerotic Cardiovascular Disease **eTable 2.** Factors Associated with SGLT2 Inhibitor Use Among Patients with Heart Failure with Reduced Ejection Fraction on Multivariable Analysis **eTable 3.** Factors Associated with SGLT2 Inhibitor Use Among Patients with Atherosclerotic Cardiovascular Disease on Multivariable Analysis **eTable 4.** Factors Associated with SGLT2 Inhibitor Use Among Patients with Chronic Kidney Disease on Multivariable Analysis **eTable 5.** Factors Associated with SGLT2 Inhibitor Use Among Patients on Metformin Therapy on Multivariable Analysis
