# Supplementary material for: Association of Race/Ethnicity, Gender, and Socioeconomic Status With Sodium-Glucose Cotransporter 2 Inhibitor Use Among Patients With Diabetes in the US
Source: JAMA Netw Open. 2021 Apr 15;4(4):e216139. doi: 10.1001/jamanetworkopen.2021.6139 (PMC8050743; doi:10.1001/jamanetworkopen.2021.6139)
Supplement: Supplement. — eTable 1. Included ICD codes for Atherosclerotic Cardiovascular Disease eTable 2. Factors Associated with SGLT2 Inhibitor Use Among Patients with Heart Failure with Reduced Ejection Fraction on Multivariable Analysis eTable 3. Factors Associated with SGLT2 Inhibitor Use Among Patients with Atherosclerotic Cardiovascular Disease on Multivariable Analysis eTable 4. Factors Associated with SGLT2 Inhibitor Use Among Patients with Chronic Kidney Disease on Multivariable Analysis eTable 5. Factors Associated with SGLT2 Inhibitor Use Among Patients on Metformin Therapy on Multivariable Analysis [file jamanetwopen-e216139-s001.pdf]

Supplementary Online Content

Eberly LA, Yang L, Eneanya ND, et al. Association of race/ethnicity, gender, and socioeconomic status with sodium-glucose cotransporter 2 inhibitor use among patients with diabetes in the US. *JAMA Netw Open*. 2021;4(4):e216139. doi:10.1001/jamanetworkopen.2021.6139

eTable 1. Included ICD codes for Atherosclerotic Cardiovascular Disease
eTable 2. Factors Associated with SGLT2 Inhibitor Use Among Patients with Heart Failure with Reduced Ejection Fraction on Multivariable Analysis
eTable 3. Factors Associated with SGLT2 Inhibitor Use Among Patients with Atherosclerotic Cardiovascular Disease on Multivariable Analysis
eTable 4. Factors Associated with SGLT2 Inhibitor Use Among Patients with Chronic Kidney Disease on Multivariable Analysis
eTable 5. Factors Associated with SGLT2 Inhibitor Use Among Patients on Metformin Therapy on Multivariable Analysis

This supplementary material has been provided by the authors to give readers additional information about their work.

© 2021 Eberly LA et al. *JAMA Network Open*.

eTable 1. Included ICD codes for Atherosclerotic Cardiovascular Disease

Name	ICD-9-CM	ICD-10-CM
Type 2 diabetes mellitus with atherosclerosis of aorta	250.70, 440.0	E11.51, I70.0
Diabetes mellitus with atherosclerosis of arteries of extremities	250.70, 440.20	E11.51, I70.209
Diabetes type 2 with atherosclerosis of arteries of extremities	250.70, 440.20	E11.51, I70.209
Diabetes type II with atherosclerosis of arteries of extremities	250.70, 440.20	E11.51, I70.209
Type 2 diabetes with atherosclerosis of arteries of extremities	250.70, 440.20	E11.51, I70.209
Diabetes mellitus type 2 with atherosclerosis of arteries of extremities	250.70, 440.20	E11.51, I70.209
Uncontrolled diabetes with atherosclerosis of extremity arteries	250.72, 440.20	E11.51, I70.209, E11.65
Uncontrolled diabetes type 2 with extremity artery atherosclerosis	250.72, 440.20	E11.51, I70.209, E11.65
Uncontrolled diabetes type II with extremity artery atherosclerosis	250.72, 440.20	E11.51, I70.209, E11.65
Uncontrolled type 2 diabetes with extremity artery atherosclerosis	250.72, 440.20	E11.51, I70.209, E11.65
Uncontrolled diabetes mellitus with atherosclerosis of arteries of extremities	250.72, 440.20	E11.51, I70.209, E11.65
Uncontrolled diabetes mellitus type 2 with atherosclerosis of arteries of extremities	250.72, 440.20	E11.51, I70.209, E11.65
Type 2 diabetes mellitus with atherosclerosis of native arteries of extremity with intermittent claudication	250.70, 440.21	E11.51, I70.219
Type 2 diabetes mellitus with atherosclerosis of native arteries of extremity with rest pain	250.70, 440.22	E11.51, I70.229
Angina pectoris associated with type 2 diabetes mellitus	250.80, 413.9	E11.59, I20.9
DM type 2 with diabetic angina pectoris	250.80, 413.9	E11.59, I20.9

© 2021 Eberly LA et al. *JAMA Network Open*.

Multi-infarct dementia due to atherosclerosis	290.40, 440.9	F01.50, I70.209
Transient ischemic attack in basilar artery distribution on medication	435	G45.0
Vertebrobasilar TIAs	435.3	G45.0
VBI (vertebrobasilar insufficiency)	435.3	G45.0
Vertebrobasilar artery insufficiency	435.3	G45.0
Top of basilar syndrome	352.6	G45.0
TIA involving basilar artery	435	G45.0
Transient ischemic attack involving basilar artery	435	G45.0
Transient ischemic attack involving vertebral artery	435.1	G45.0
TIA involving vertebral artery	435.1	G45.0
Vertebro-basilar artery syndrome		G45.0
Insufficiency of basilar, carotid, and vertebral arteries	435.0, 435.1, 435.8	G45.0, G45.1
Carotid artery syndrome	435.8	G45.1
Carotid artery insufficiency syndrome	435.8	G45.1
Carotid artery occlusion syndrome	435.8	G45.1
Insufficiency, arterial, carotid artery	435.8	G45.1
Carotid insufficiency	435.8	G45.1
Carotid artery syndrome hemispheric	435.8	G45.1
TIA involving left internal carotid artery	435.8	G45.1
Transient ischemic attack involving right internal carotid artery	435.8	G45.1
Transient ischemic attack involving carotid artery	435.8	G45.1
TIA involving carotid artery	435.8	G45.1
TIA involving right internal carotid artery	435.8	G45.1
Transient ischemic attack involving left internal carotid artery	435.8	G45.1
Hemispheric carotid artery syndrome	435.8	G45.1
Transient ischemic attack involving internal carotid artery	435.8	G45.1
Carotid artery syndrome (hemispheric)	435.8	G45.1
Carotid artery syndrome (hemispheric)		G45.1

© 2021 Eberly LA et al. *JAMA Network Open*.

Multiple and bilateral precerebral artery syndromes	433.3	G45.2
Multiple and bilateral precerebral artery syndromes		G45.2
Amaurosis fugax	362.34	G45.3
AF (amaurosis fugax)	362.34	G45.3
AFX (amaurosis fugax)	362.34	G45.3
Amaurosis fugax of right eye	362.34	G45.3
Amaurosis fugax, right eye	362.34	G45.3
Amaurosis fugax of left eye	362.34	G45.3
Amaurosis fugax, left eye	362.34	G45.3
Amaurosis fugax, both eyes	362.34	G45.3
Bilateral amaurosis fugax	362.34	G45.3
Retinal amaurosis fugax	362.34	G45.3
Recurrent amaurosis fugax	362.34	G45.3
Amaurosis fugax		G45.3
Subclavian steal syndrome	435.2	G45.8
Other specified transient cerebral ischemias	435.8	G45.8
Brachial-basilar insufficiency syndrome	435.2	G45.8
Steal syndrome, subclavian	435.2	G45.8
Anterior circulation transient ischemic attack	435.9	G45.8
Subclavian artery occlusive syndrome	435.2	G45.8
Steal syndrome, basilar	435.2	G45.8
Acute cerebrovascul insuff, transient focal neurologic signs/symptoms	435.9	G45.8
Acute cerebrovascular insufficiency transient focal neurologic deficit	435.9	G45.8
Acute posterior circulation transient ischemic attack	435.9	G45.8
Acute anterior circulation transient ischemic attack	435.9	G45.8
Transient ischemic attack, anterior circulation, acute	435.9	G45.8
Transient ischemic attack, posterior circulation, acute	435.9	G45.8

© 2021 Eberly LA et al. *JAMA Network Open*.

Acute anterior circulation TIA	435.9	G45.8
Transient ischemic attack in carotid artery distribution on medication	435.8	G45.8
Acute cerebrovascular insufficiency with transient focal neurological signs and symptoms	435.9	G45.8
Other transient cerebral ischemic attacks and related syndromes	435.8	G45.8
Other transient cerebral ischemic attacks and related syndromes		G45.8
Crescendo transient ischemic attacks	435.8	G45.8
Transient brainstem ischemia	435.8	G45.8
Postoperative transient ischemic attack (TIA)	435.8	G45.8, I97.89
Postoperative transient cerebral ischemia	435.8	G45.8, I97.89
Unspecified transient cerebral ischemia	435.9	G45.9
Intermittent cerebral ischemia	435.9	G45.9
TIA (transient ischemic attack)	435.9	G45.9
Transient ischemic attack	435.9	G45.9
Transient ischemic attack (TIA)	435.9	G45.9
Transient cerebral ischemia	435.9	G45.9
Transient cerebral ischemic attack	435.9	G45.9
Brain TIA	435.9	G45.9
Transient ischemic attack, acute	435.9	G45.9
Transient cerebrovascular ischemia	435.9	G45.9
Cerebrovascular ischemia, transient	435.9	G45.9
Precerebral artery insufficiency	435.9	G45.9
Insufficiency, arterial, precerebral	435.9	G45.9
Transient ischemic attack on medication	435.9	G45.9
TIA on medication	435.9	G45.9
Transient ischemic attack with other course	435.9	G45.9
Transient cerebral ischemia, unspecified transient cerebral ischemia type	435.9	G45.9
Transient cerebral ischemic attack, unspecified	435.9	G45.9

© 2021 Eberly LA et al. *JAMA Network Open*.

Transient cerebral ischemia, unspecified type	435.9	G45.9
Transient cerebral ischemic attack, unspecified		G45.9
Transient ischemic attack with visual impairment	435.9, 369.9	G45.9, H54.7
Vascular syndromes of brain in cerebrovascular diseases		G46
Middle cerebral artery syndrome	434.9	G46.0
Middle cerebral artery syndrome		G46.0
Anterior cerebral artery syndrome	434.9	G46.1
Anterior cerebral artery syndrome		G46.1
Posterior cerebral artery syndrome	434.9	G46.2
Posterior cerebral artery syndrome		G46.2
Ventral medullary syndrome	437.8	G46.3
Foville's peduncular syndrome	344.89	G46.3
Millard-Gubler syndrome	344.89	G46.3
Benedikt's syndrome	344.89	G46.3
Mesencephalic tegmental paralysis	344.89	G46.3
Tegmentum syndrome	344.89	G46.3
Weber-Gubler syndrome	344.89	G46.3
Facial-abducens-hemiplegia syndrome	344.89	G46.3
Tegmental syndrome	344.89	G46.3
Millard Gubler syndrome	344.89	G46.3
Benedict syndrome	344.89	G46.3
Millard Gublar syndrome	344.89	G46.3
Posterior inferior cerebellar artery syndrome	434.91	G46.3
Inferior cerebellar artery syndrome	434.91	G46.3
Lateral medullary syndrome	434.91	G46.3
Wallenberg's syndrome	434.91	G46.3
Lateral bulbar syndrome	434.91	G46.3
Vieusseux-Wallenberg syndrome	434.91	G46.3
Wallenberg syndrome	434.91	G46.3
Wallenbergs syndrome	434.91	G46.3
LMS (lateral medullary syndrome)	434.91	G46.3
Dorsolateral medullary syndrome	434.91	G46.3

© 2021 Eberly LA et al. *JAMA Network Open*.

Weber syndrome	344.89	G46.3
Brain stem stroke syndrome	436	G46.3
Brainstem stroke syndrome	436	G46.3
Stroke, Wallenberg's syndrome	434.91	G46.3
Brain stem stroke syndrome		G46.3
Cerebellar stroke syndrome	436	G46.4
Cerebellar stroke syndrome		G46.4
Pure motor lacunar syndrome	434.91	G46.5
Pure motor lacunar syndrome		G46.5
Pure sensory lacunar syndrome	434.91	G46.6
Pure sensory lacunar syndrome		G46.6
Lacunar syndrome	437.8	G46.7
Dysarthria-clumsy hand syndrome	434.91, 784.51	G46.7
Other lacunar syndromes	437.8	G46.7
Other lacunar syndromes		G46.7
Other vascular syndromes of brain in cerebrovascular diseases	437.8	G46.8
Other vascular syndromes of brain in cerebrovascular diseases		G46.8
Flaccid hemiplegia due to nontraumatic subarachnoid hemorrhage, unspecified hemiplegia laterality	342.00, 430	G81.00, I60.9
Flaccid hemiplegia due to nontraumatic intraparenchymal hemorrhage of brain, unspecified hemiplegia laterality	342.00, 431	G81.00, I61.9
Flaccid hemiplegia due to other nontraumatic intracranial hemorrhage, unspecified hemiplegia laterality	342.00, 432.9	G81.00, I62.9
Flaccid hemiplegia due to infarction of brain, unspecified hemiplegia laterality	342.00, 434.91	G81.00, I63.9
Flaccid hemiplegia due to other cerebrovascular disease, unspecified hemiplegia laterality	342.00, 437.8	G81.00, I67.89
Flaccid hemiplegia due to cerebrovascular disease, unspecified cerebrovascular disease type, unspecified hemiplegia laterality	342.00, 437.9	G81.00, I67.9

© 2021 Eberly LA et al. *JAMA Network Open*.

Flaccid hemiplegia of right dominant side due to cerebrovascular disease, unspecified cerebrovascular disease type	342.01, 437.9	G81.01, I67.9
Flaccid hemiplegia of left dominant side due to cerebrovascular disease, unspecified cerebrovascular disease type	342.01, 437.9	G81.02, I67.9
Flaccid hemiplegia of right nondominant side due to cerebrovascular disease, unspecified cerebrovascular disease type	342.02, 437.9	G81.03, I67.9
Flaccid hemiplegia of left nondominant side due to cerebrovascular disease, unspecified cerebrovascular disease type	342.02, 437.9	G81.04, I67.9
Spastic hemiplegia due to nontraumatic intraparenchymal hemorrhage of brain, unspecified hemiplegia laterality	342.10, 431	G81.10, I61.9
Spastic hemiplegia due to infarction of brain, unspecified hemiplegia laterality	342.10, 434.91	G81.10, I63.9
Spastic hemiplegia due to cerebrovascular disease, unspecified cerebrovascular disease type, unspecified hemiplegia laterality	342.10, 437.9	G81.10, I67.9
Spastic hemiplegia of right dominant side due to cerebrovascular disease, unspecified cerebrovascular disease type	342.11, 437.9	G81.11, I67.9
Spastic hemiplegia of left dominant side due to cerebrovascular disease, unspecified cerebrovascular disease type	342.11, 437.9	G81.12, I67.9
Spastic hemiplegia of right nondominant side due to cerebrovascular disease, unspecified cerebrovascular disease type	342.12, 437.9	G81.13, I67.9
Spastic hemiplegia of left nondominant side due to cerebrovascular disease, unspecified cerebrovascular disease type	342.12, 437.9	G81.14, I67.9
Hemiplegia due to nontraumatic subarachnoid hemorrhage, unspecified hemiplegia laterality, unspecified hemiplegia type	342.90, 430	G81.90, I60.9

© 2021 Eberly LA et al. *JAMA Network Open*.

Hemiplegia due to nontraumatic intraparenchymal hemorrhage of brain, unspecified hemiplegia laterality, unspecified hemiplegia type	342.90, 431	G81.90, I61.9
Hemiplegia due to other nontraumatic intracranial hemorrhage, unspecified hemiplegia laterality, unspecified hemiplegia type	342.90, 432.9	G81.90, I62.9
Hemiplegia due to infarction of brain, unspecified hemiplegia laterality, unspecified hemiplegia type	342.90, 434.91	G81.90, I63.9
Hemiplegia due to other cerebrovascular disease, unspecified hemiplegia laterality, unspecified hemiplegia type	342.90, 437.8	G81.90, I67.89
Hemiplegia due to cerebrovascular disease, unspecified cerebrovascular disease type, unspecified hemiplegia laterality, unspecified hemiplegia type	342.90, 437.9	G81.90, I67.9
Hemiplegia of left nondominant side due to nontraumatic intraparenchymal hemorrhage of brain, unspecified hemiplegia type	438.22, 431	G81.94, I61.9
Hemiplegia of left nondominant side due to infarction of brain, unspecified hemiplegia type	438.22, 429.79	G81.94, I63.9
Anoxic-ischemic encephalopathy	348.1, 437.1	G93.1, I67.82
Mild anoxic-ischemic encephalopathy	348.1, 437.1	G93.1, I67.82
Moderate anoxic-ischemic encephalopathy	348.1, 437.1	G93.1, I67.82
Severe anoxic-ischemic encephalopathy	348.1, 437.1	G93.1, I67.82
Compression of brain due to nontraumatic subarachnoid hemorrhage	348.4, 430	G93.5, I60.9
Compression of brain due to spontaneous cerebral hemorrhage	348.4, 431	G93.5, I61.9
TIA occurring during procedure	997.01, 435.9	G97.81, G45.9
Transient ischemic attack during procedure	997.01, 435.9	G97.81, G45.9

© 2021 Eberly LA et al. *JAMA Network Open*.

Transient ischemic attack due to procedure	997.01, 435.9	G97.82, G45.9
TIA resulting from procedure	997.01, 435.9	G97.82, G45.9
Transient retinal artery occlusion		H34.0
Transient arterial occlusion of retina	362.34	H34.00
Transient arterial retinal occlusion	362.34	H34.00
Occlusion, retinal, arterial, transient	362.34	H34.00
Transient retinal artery occlusion	362.34	H34.00
Transient arterial retinal occlusion, unspecified laterality	362.34	H34.00
Occlusion, retinal, arterial, transient, unspecified laterality	362.34	H34.00
Transient arterial occlusion of retina, unspecified laterality	362.34	H34.00
Transient retinal artery occlusion, unspecified laterality	362.34	H34.00
Transient retinal artery occlusion, unspecified eye	362.34	H34.00
Transient retinal artery occlusion, unspecified eye		H34.00
Transient arterial retinal occlusion of right eye	362.34	H34.01
Transient arterial retinal occlusion, right	362.34	H34.01
Transient arterial occlusion of retina, right	362.34	H34.01
Transient retinal arterial occlusion, right	362.34	H34.01
Transient retinal artery occlusion of right eye	362.34	H34.01
Transient retinal artery occlusion, right	362.34	H34.01
Occlusion, retinal, arterial, transient, right	362.34	H34.01
Transient retinal artery occlusion, right eye	362.34	H34.01
Transient retinal artery occlusion, right eye		H34.01
Transient arterial retinal occlusion of left eye	362.34	H34.02
Transient arterial retinal occlusion, left	362.34	H34.02
Transient retinal arterial occlusion, left	362.34	H34.02

© 2021 Eberly LA et al. *JAMA Network Open*.

Transient arterial occlusion of retina, left	362.34	H34.02
Transient retinal artery occlusion of left eye	362.34	H34.02
Transient retinal artery occlusion, left	362.34	H34.02
Occlusion, retinal, arterial, transient, left	362.34	H34.02
Transient retinal artery occlusion, left eye	362.34	H34.02
Transient retinal artery occlusion, left eye		H34.02
Transient arterial retinal occlusion of both eyes	362.34	H34.03
Transient arterial retinal occlusion, bilateral	362.34	H34.03
Transient retinal arterial occlusion, bilateral	362.34	H34.03
Transient arterial occlusion of retina, bilateral	362.34	H34.03
Transient retinal artery occlusion of both eyes	362.34	H34.03
Transient retinal artery occlusion, bilateral	362.34	H34.03
Occlusion, retinal, arterial, transient, bilateral	362.34	H34.03
Transient retinal artery occlusion, bilateral		H34.03
Benign secondary hypertension due to renal artery stenosis	405.11, 440.1	I15.0, I70.1
Malignant secondary hypertension due to renal artery stenosis	405.01, 440.1	I15.0, I70.1
Angina pectoris		I20
Intermediate coronary syndrome	411.1	I20.0
Preinfarction syndrome	411.1	I20.0
Crescendo angina	411.1	I20.0
Unstable angina	411.1	I20.0
Impending infarction	411.1	I20.0
Preinfarction angina	411.1	I20.0
Angina pectoris, unstable	411.1	I20.0
Angina, preinfarctional	411.1	I20.0
Unstable angina pectoris	411.1	I20.0
Intermediate ischemic heart syndrome	411.1	I20.0

© 2021 Eberly LA et al. *JAMA Network Open*.

Angina pectoris, crescendo	411.1	I20.0
Impending, myocardial infarction	411.1	I20.0
Myocardial preinfarction syndrome	411.1	I20.0
Pre-infarction syndrome	411.1	I20.0
Worsening angina	413.9	I20.0
Angina pectoris, preinfarctional	411.1	I20.0
Accelerating angina	411.1	I20.0
Progressive angina	411.1	I20.0
Acute coronary insufficiency syndrome	411.1	I20.0
Unstable chest pain due to insufficient blood supply to heart	411.89	I20.0
Myocardial infarction, impending	411.1	I20.0
Impending myocardial infarction	411.1	I20.0
Aborted myocardial infarction	411.1	I20.0
Unstable angina		I20.0
Angina decubitus	413	I20.8
Angina of effort	413.9	I20.8
Anginal chest pain at rest	413	I20.8
Nocturnal angina	413	I20.8
Syncope anginosa	413.9	I20.8
Angina effort	413.9	I20.8
Effort angina	413.9	I20.8
Refractory angina	413.9	I20.8
Exertional angina	413.9	I20.8
Stable angina	413.9	I20.8
Angina at rest	413.9	I20.8
Exercise-induced angina	413.9	I20.8
Angina pectoris, nocturnal	413	I20.8
Anginal equivalent	413.9	I20.8
Chronic stable angina	413.9	I20.8
Other forms of angina pectoris	413.9	I20.8
Stable angina pectoris	413.9	I20.8
Other forms of angina pectoris		I20.8
Other and unspecified angina pectoris	413.9	I20.9
Anginal syndrome	413.9	I20.9
Cardiac angina	413.9	I20.9
Angina, class I	413.9	I20.9

© 2021 Eberly LA et al. *JAMA Network Open*.

Angina, class II	413.9	I20.9
Angina, class III	413.9	I20.9
Angina, class IV	413.9	I20.9
Status anginosus	413.9	I20.9
Angina pectoris syndrome	413.9	I20.9
Anginal pain	413.9	I20.9
Angor pectoris	413.9	I20.9
Cardiac angina syndrome	413.9	I20.9
Angina pectoris with normal coronary arteriogram	413.9	I20.9
AP (angina pectoris)	413.9	I20.9
Angina pectoris	413.9	I20.9
Acute angina	413.9	I20.9
New-onset angina	413.9	I20.9
Typical angina	413.9	I20.9
2 or more angina events in past 24 hours	413.9	I20.9
Angina pectoris, unspecified	413.9	I20.9
Angina pectoris without myocardial infarction	413.9	I20.9
Angina pectoris, unspecified		I20.9
Recurrent angina status post coronary artery bypass graft	413.9, V45.81	I20.9, Z95.1
Recurrent angina status post coronary stent placement	413.9, V45.81	I20.9, Z95.5
Recurrent angina status post percutaneous transluminal coronary angioplasty	413.9, V45.82	I20.9, Z98.61
Recurrent angina status post rotational atherectomy	413.9, V45.89	I20.9, Z98.890
Recurrent angina status post directional coronary atherectomy	413.9, V45.89	I20.9, Z98.890
Acute myocardial infarction		I21
ST elevation (STEMI) myocardial infarction of anterior wall		I21.0
ST elevation myocardial infarction involving left main coronary artery	410.11	I21.01

© 2021 Eberly LA et al. *JAMA Network Open*.

ST elevation myocardial infarction (STEMI) involving left main coronary artery in recovery phase	410.1	I21.01
ST elevation myocardial infarction (STEMI) involving left main coronary artery with complication	410.1	I21.01
Acute Q wave myocardial infarction involving left main coronary artery	410.1	I21.01
Acute ST elevation myocardial infarction involving left main coronary artery	410.1	I21.01
Acute non-Q wave ST elevation myocardial infarction (STEMI) involving left main coronary artery	410.1	I21.01
Widespread acute non-Q wave ST elevation myocardial infarction (STEMI) involving left main coronary artery	410.1	I21.01
Widespread acute Q wave ST elevation myocardial infarction (STEMI) involving left main coronary artery	410.1	I21.01
Non-Q wave ST elevation myocardial infarction (STEMI) involving left main coronary artery	410.1	I21.01
Silent ST elevation myocardial infarction (STEMI) involving left main coronary artery	410.1	I21.01
Acute ST elevation myocardial infarction (STEMI) involving left main coronary artery	410.1	I21.01
Acute myocardial infarction involving left main coronary artery	410.11	I21.01
Myocardial infarction involving left main coronary artery	410.1	I21.01
Acute Q wave myocardial infarction involving left coronary artery	410.1	I21.01
Acute ST elevation myocardial infarction (STEMI) involving anomalous left coronary artery	410.1	I21.01

© 2021 Eberly LA et al. *JAMA Network Open*.

Acute non-Q wave ST elevation myocardial infarction (STEMI) involving left coronary artery	410.1	I21.01
Acute ST elevation myocardial infarction (STEMI) involving left main coronary artery without development of Q waves	410.1	I21.01
Widespread acute ST elevation myocardial infarction (STEMI) involving left main coronary artery without development of Q waves	410.1	I21.01
Acute ST elevation myocardial infarction (STEMI) involving left coronary artery without development of Q waves	410.1	I21.01
ST elevation myocardial infarction (STEMI) involving left main coronary artery without development of Q waves	410.1	I21.01
ST elevation (STEMI) myocardial infarction involving left main coronary artery	410.11	I21.01
STEMI involving left main coronary artery	410.11	I21.01
ST elevation (STEMI) myocardial infarction involving left main coronary artery		I21.01
ST elevation myocardial infarction involving left anterior descending coronary artery	410.1	I21.02
ST elevation (STEMI) myocardial infarction involving left anterior descending coronary artery	410.1	I21.02
ST elevation myocardial infarction (STEMI) involving left anterior descending (LAD) coronary artery with complication	410.1	I21.02
ST elevation myocardial infarction (STEMI) involving left anterior descending (LAD) coronary artery in recovery phase	410.1	I21.02
Acute Q wave myocardial infarction involving left anterior descending (LAD) coronary artery	410.1	I21.02

© 2021 Eberly LA et al. *JAMA Network Open*.

Acute ST elevation myocardial infarction involving left anterior descending coronary artery	410.1	I21.02
Acute non-Q wave ST elevation myocardial infarction (STEMI) involving left anterior descending (LAD) coronary artery	410.8	I21.02
Widespread acute non-Q wave ST elevation myocardial infarction (STEMI) involving left anterior descending (LAD) coronary artery	410.1	I21.02
Widespread acute Q wave ST elevation myocardial infarction (STEMI) involving left anterior descending (LAD) coronary artery	410.1	I21.02
Non-Q wave ST elevation myocardial infarction (STEMI) involving left anterior descending (LAD) coronary artery	410.1	I21.02
Silent ST elevation myocardial infarction (STEMI) involving left anterior descending (LAD) coronary artery of anterior wall	410.1	I21.02
Acute ST elevation myocardial infarction (STEMI) involving left anterior descending coronary artery	410.1	I21.02
Acute myocardial infarction involving left anterior descending (LAD) coronary artery	410.11	I21.02
Myocardial infarction involving left anterior descending (LAD) coronary artery	410.1	I21.02
ST elevation myocardial infarction involving left anterior descending (LAD) coronary artery	410.1	I21.02
Acute ST elevation myocardial infarction (STEMI) involving left anterior descending (LAD) coronary artery	410.1	I21.02
Acute ST elevation myocardial infarction (STEMI) involving left anterior descending (LAD) coronary artery without development of Q waves	410.8	I21.02

© 2021 Eberly LA et al. *JAMA Network Open*.

Widespread acute ST elevation myocardial infarction (STEMI) involving left anterior descending (LAD) coronary artery without development of Q waves	410.1	I21.02
ST elevation myocardial infarction (STEMI) involving left anterior descending (LAD) coronary artery without development of Q waves	410.1	I21.02
STEMI involving left anterior descending coronary artery	410.1	I21.02
ST elevation (STEMI) myocardial infarction involving left anterior descending coronary artery		I21.02
Acute myocardial infarction of anterolateral wall, episode of care unspecified	410	I21.09
Acute myocardial infarction of anterolateral wall, initial episode of care	410.01	I21.09
Acute myocardial infarction of anterolateral wall, subsequent episode of care	410.02	I21.09
Acute myocardial infarction of other anterior wall, episode of care unspecified	410.1	I21.09
Acute myocardial infarction of other anterior wall, initial episode of care	410.11	I21.09
Acute myocardial infarction of other anterior wall, subsequent episode of care	410.12	I21.09
Acute myocardial infarction of anterior wall	410.1	I21.09
Acute anteroapical myocardial infarction	410.1	I21.09
Acute anteroseptal myocardial infarction	410.1	I21.09
Acute myocardial infarction of anterolateral wall	410	I21.09
ALMI (anterolateral wall myocardial infarction)	410	I21.09
Acute myocardial infarction of anterior wall, initial episode of care	410.11	I21.09

© 2021 Eberly LA et al. *JAMA Network Open*.

Acute anterolateral myocardial infarction	410	I21.09
Additional acute myocardial infarction (anterolateral wall)	410.02	I21.09
Additional heart attack (anterolateral wall)	410.02	I21.09
Anterior myocardial infarction	410.1	I21.09
Acute MI anterior lateral subsequent episode care	410.02	I21.09
Acute MI anterior wall first episode care	410.11	I21.09
Acute MI anterior wall subsequent episode care	410.12	I21.09
ST elevation myocardial infarction (STEMI) of anterolateral wall	410	I21.09
ST elevation myocardial infarction (STEMI) of anterior wall	410.1	I21.09
AMI anterior wall	410.1	I21.09
AMI anterolateral wall	410	I21.09
Acute anterior myocardial infarction	410.1	I21.09
F/u of anterolateral myocardial infarction	410.02	I21.09
Follow-up of anterolateral myocardial infarction	410.02	I21.09
F/u of acute anterolateral myocardial infarction	410.02	I21.09
Follow-up of acute anterolateral myocardial infarction	410.02	I21.09
Acute transmural myocardial infarction of anterior wall	410.1	I21.09
Acute transmural anterior wall MI	410.1	I21.09
Myocardial infarction, anterior wall, initial care	410.11	I21.09
Myocardial infarction, anterolateral wall	410	I21.09
Myocardial infarction, anterolateral wall, initial care	410.01	I21.09
Myocardial infarction, anterior wall	410.1	I21.09
Myocardial infarction, anterior wall, subsequent care	410.12	I21.09

© 2021 Eberly LA et al. *JAMA Network Open*.

Myocardial infarction, anterolateral wall, subsequent care	410.02	I21.09
Myocardial infarction, anterior, acute, initial episode	410.11	I21.09
Myocardial infarction, anterolateral, acute	410	I21.09
Myocardial infarction, anterolateral wall, acute	410	I21.09
Acute transmural myocardial infarction of anterolateral wall	410.01	I21.09
Anteroapical myocardial infarction	410.1	I21.09
Anteroseptal myocardial infarction	410.1	I21.09
Anterolateral myocardial infarction	410	I21.09
Myocardial infarction of anterolateral wall	410	I21.09
Acute MI, anterior wall	410.1	I21.09
Acute MI, anterolateral wall	410	I21.09
Acute MI, anterior wall, initial episode of care	410.11	I21.09
Acute MI, anterolateral wall, initial episode of care	410.01	I21.09
Acute MI, anterior wall, subsequent episode of care	410.12	I21.09
Acute MI, anterolateral wall, subsequent episode of care	410.02	I21.09
Myocardial infarction, anteroseptal	410.1	I21.09
Acute anterior wall MI	410.1	I21.09
Acute anterolateral wall MI	410	I21.09
Anterolateral wall myocardial infarction	410	I21.09
Acute transmural myocardial infarct anterior wall, initial hospitaliz	410.11	I21.09
Transmural anteroseptal myocardial infarction, initial hospitalization	410.11	I21.09
Transmural anteroapical myocardial infarction, initial hospitalization	410.11	I21.09
Acute myocardial infarction, of anterolateral wall, subsequent episode of care	410.02	I21.09

© 2021 Eberly LA et al. *JAMA Network Open*.

Acute myocardial infarction of anterior wall, subsequent episode of care	410.12	I21.09
ST elevation myocardial infarction (STEMI) of anterolateral wall, initial episode of care	410.01	I21.09
ST elevation myocardial infarction (STEMI) of anterolateral wall, subsequent episode of care	410.02	I21.09
ST elevation myocardial infarction (STEMI) of anterior wall, initial episode of care	410.11	I21.09
ST elevation myocardial infarction (STEMI) of anterior wall, subsequent episode of care	410.12	I21.09
Acute transmural myocardial infarction of anterior wall, initial hospitalization	410.11	I21.09
Transmural myocardial infarction of anterolateral wall, initial hospitalization	410.01	I21.09
ST segment elevation myocardial infarction (STEMI) of anterolateral wall, subsequent episode of care	410.02	I21.09
Acute myocardial infarction of other anterior wall	410.1	I21.09
Acute Q wave myocardial infarction of anteroseptal wall	410.1	I21.09
Acute Q wave myocardial infarction of anterolateral wall	410.01	I21.09
Acute myocardial infarction of anterior wall, subsequent to initial episode of care	410.12	I21.09
Acute myocardial infarction of anterolateral wall, subsequent to initial episode of care	410.02	I21.09
ST segment elevation myocardial infarction (STEMI) of anterolateral wall, subsequent to initial episode of care	410.02	I21.09
Anterior wall myocardial infarction	410.1	I21.09
Myocardial infarction of anterior wall	410.1	I21.09
Anterior ST segment elevation	410.1	I21.09

© 2021 Eberly LA et al. *JAMA Network Open*.

ST elevation myocardial infarction (STEMI) involving other coronary artery of anterior wall	410.1	I21.09
Acute ST elevation myocardial infarction (STEMI) involving other coronary artery of anterior wall	410.1	I21.09
Acute myocardial infarction involving other coronary artery of anterior wall	410.1	I21.09
Myocardial infarction involving other coronary artery of anterior wall	410.1	I21.09
AMI anterolatrl wall	410	I21.09
Anterolat AMI, init episd	410.01	I21.09
Anter AMI NEC, episd	410.1	I21.09
Anter AMI NEC, init episd	410.11	I21.09
AMI anterolateral,subseq	410.02	I21.09
AMI anterior wall,subseq	410.12	I21.09
Acute myocardial infarction of other anterior wall, subsequent to initial episode of care	410.12	I21.09
Acute ST elevation myocardial infarction (STEMI) of anterolateral wall	410.1	I21.09
Acute ST elevation myocardial infarction (STEMI) of anteroseptal wall	410.1	I21.09
ST elevation (STEMI) myocardial infarction involving other coronary artery of anterior wall	410.1	I21.09
Acute myocardial infarction of anterior wall involving right ventricle	410.1	I21.09
Acute ST elevation myocardial infarction (STEMI) of anterior wall involving right ventricle	410.1	I21.09
Acute ST elevation myocardial infarction (STEMI) of anterior wall	410.1	I21.09
STEMI involving oth coronary artery of anterior wall	410.1	I21.09

© 2021 Eberly LA et al. *JAMA Network Open*.

ST elevation (STEMI) myocardial infarction involving other coronary artery of anterior wall (CODE)	410.1	I21.09
ST elevation (STEMI) myocardial infarction involving other coronary artery of anterior wall		I21.09
Anterior and lateral ST segment elevation	410	I21.09, I21.29
ST elevation (STEMI) myocardial infarction of inferior wall		I21.1
Transmural myocardial infarction of inferoposterior wall, initial hospitalization	410.31	I21.11
ST elevation myocardial infarction involving right coronary artery	410.31	I21.11
Acute myocardial infarction involving right coronary artery	410.9	I21.11
ST elevation myocardial infarction (STEMI) involving right coronary artery in recovery phase	410.1	I21.11
ST elevation myocardial infarction (STEMI) involving right coronary artery with complication	410.1	I21.11
Acute Q wave myocardial infarction involving right coronary artery	410.1	I21.11
Acute ST elevation myocardial infarction (STEMI) involving right coronary artery	410.1	I21.11
Widespread acute non-Q wave ST elevation myocardial infarction (STEMI) involving right coronary artery	410.1	I21.11
Widespread acute Q wave ST elevation myocardial infarction (STEMI) involving right coronary artery	410.1	I21.11
Acute non-Q wave ST elevation myocardial infarction (STEMI) involving right coronary artery	410.1	I21.11
Non-Q wave ST elevation myocardial infarction (STEMI) involving right coronary artery	410.1	I21.11

© 2021 Eberly LA et al. *JAMA Network Open*.

Silent ST elevation myocardial infarction (STEMI) involving right coronary artery	410.1	I21.11
Myocardial infarction involving right coronary artery	410.3	I21.11
Acute myocardial infarction due to right coronary artery occlusion	410.3	I21.11
Acute ST elevation myocardial infarction (STEMI) involving right coronary artery without development of Q waves	410.1	I21.11
Widespread acute ST elevation myocardial infarction (STEMI) involving right coronary artery without development of Q waves	410.1	I21.11
ST elevation myocardial infarction (STEMI) involving right coronary artery without development of Q waves	410.1	I21.11
ST elevation (STEMI) myocardial infarction involving right coronary artery	410.31	I21.11
STEMI involving right coronary artery	410.31	I21.11
ST elevation (STEMI) myocardial infarction involving right coronary artery		I21.11
Acute myocardial infarction of inferolateral wall, episode of care unspecified	410.2	I21.19
Acute myocardial infarction of inferolateral wall, initial episode of care	410.21	I21.19
Acute myocardial infarction of inferolateral wall, subsequent episode of care	410.22	I21.19
Acute myocardial infarction of inferoposterior wall, episode of care unspecified	410.3	I21.19
Acute myocardial infarction of inferoposterior wall, initial episode of care	410.31	I21.19
Acute myocardial infarction of inferoposterior wall, subsequent episode of care	410.32	I21.19

© 2021 Eberly LA et al. *JAMA Network Open*.

Acute myocardial infarction of other inferior wall, episode of care unspecified	410.4	I21.19
Acute myocardial infarction of other inferior wall, initial episode of care	410.41	I21.19
Acute myocardial infarction of other inferior wall, subsequent episode of care	410.42	I21.19
Acute myocardial infarction of inferior wall	410.4	I21.19
Acute myocardial infarction of diaphragmatic wall	410.4	I21.19
Acute myocardial infarction of inferoposterior wall	410.3	I21.19
Acute myocardial infarction of inferolateral wall	410.2	I21.19
Acute myocardial infarction of inferior wall, initial episode of care	410.41	I21.19
Acute inferior myocardial infarction	410.4	I21.19
Additional acute myocardial infarction (inferoposterior wall)	410.32	I21.19
Additional heart attack (inferoposterior wall)	410.32	I21.19
Additional acute myocardial infarction (inferolateral wall)	410.22	I21.19
Additional heart attack (inferolateral wall)	410.22	I21.19
Inferior MI	410.4	I21.19
Acute MI inferior lateral first episode care	410.21	I21.19
Acute MI inferior lateral subsequent episode care	410.22	I21.19
Acute MI inferior posterior subsequent episode care	410.32	I21.19
Acute MI inferior subsequent episode care	410.42	I21.19
ST elevation myocardial infarction (STEMI) of inferolateral wall	410.2	I21.19
ST elevation myocardial infarction (STEMI) of inferoposterior wall	410.3	I21.19
ST elevation myocardial infarction (STEMI) of inferior wall	410.4	I21.19

© 2021 Eberly LA et al. *JAMA Network Open*.

AMI inferoposterior wall	410.3	I21.19
Acute inferolateral myocardial infarction	410.2	I21.19
F/u of inferolateral myocardial infarction	410.22	I21.19
Follow-up of acute inferolateral myocardial infarction	410.22	I21.19
Follow-up of inferolateral myocardial infarction	410.22	I21.19
F/u of acute inferolateral myocardial infarction	410.22	I21.19
F/u of inferoposterior myocardial infarction	410.32	I21.19
F/u of acute inferoposterior myocardial infarction	410.32	I21.19
Follow-up of acute inferoposterior myocardial infarction	410.32	I21.19
Follow-up of inferoposterior myocardial infarction	410.32	I21.19
Acute transmural myocardial infarction of inferior wall	410.4	I21.19
Acute transmural inferior wall MI	410.4	I21.19
Myocardial infarction, inferior wall, initial care	410.41	I21.19
Myocardial infarction, inferolateral wall	410.2	I21.19
Myocardial infarction, inferolateral wall, initial care	410.21	I21.19
Myocardial infarction, inferoposterior wall	410.3	I21.19
Myocardial infarction, inferoposterior wall, initial care	410.31	I21.19
Myocardial infarction, inferior wall	410.4	I21.19
Myocardial infarction, inferior wall, subsequent care	410.42	I21.19
Myocardial infarction, inferolateral wall, subsequent care	410.22	I21.19
Myocardial infarction, inferoposterior wall, subsequent care	410.32	I21.19
Myocardial infarction, inferior, acute, initial episode	410.41	I21.19

© 2021 Eberly LA et al. *JAMA Network Open*.

Myocardial infarction, inferolateral, acute, initial episode	410.21	I21.19
Myocardial infarction, inferoposterior, acute, initial episode	410.31	I21.19
Acute transmural myocardial infarction of inferolateral wall	410.21	I21.19
Transmural acute myocardial infarction of inferoposterior wall	410.31	I21.19
Transmural acute inferoposterior myocardial infarction	410.31	I21.19
Inferolateral myocardial infarction	410.2	I21.19
Myocardial infarction of inferolateral wall	410.2	I21.19
Myocardial infarction of inferoposterior wall	410.3	I21.19
Inferoposterior myocardial infarction	410.3	I21.19
Acute inferoposterior myocardial infarction	410.3	I21.19
Acute MI, inferolateral wall	410.2	I21.19
Acute MI, inferoposterior wall	410.3	I21.19
Acute MI, inferolateral wall, initial episode of care	410.21	I21.19
Acute MI, inferoposterior wall, initial episode of care	410.31	I21.19
Acute MI, inferolateral wall, subsequent episode of care	410.22	I21.19
Acute MI, inferoposterior wall, subsequent episode of care	410.32	I21.19
Acute MI, inferior wall	410.4	I21.19
Acute MI, inferior wall, initial episode of care	410.41	I21.19
Acute MI, inferior wall, subsequent episode of care	410.42	I21.19
Acute MI, other inferior wall	410.4	I21.19
Inferior myocardial infarction	410.4	I21.19
Complicated inferolateral myocardial infarction	410.2	I21.19

© 2021 Eberly LA et al. *JAMA Network Open*.

Inferolateral myocardial infarction with complication	410.2	I21.19
Transmural acute myocardial infarct inferior wall, initial hospitaliz	410.41	I21.19
Acute myocardial infarction, of inferolateral wall, initial episode of care	410.21	I21.19
Acute myocardial infarction, of inferolateral wall, subsequent episode of care	410.22	I21.19
Acute myocardial infarction, of inferoposterior wall, subsequent episode of care	410.32	I21.19
Acute myocardial infarction of inferior wall, subsequent episode of care	410.42	I21.19
AMI inferior wall	410.4	I21.19
AMI inferolateral wall	410.2	I21.19
ST elevation myocardial infarction (STEMI) of inferolateral wall, initial episode of care	410.21	I21.19
ST elevation myocardial infarction (STEMI) of inferolateral wall, subsequent episode of care	410.22	I21.19
ST elevation myocardial infarction (STEMI) of inferoposterior wall, initial episode of care	410.31	I21.19
ST elevation myocardial infarction (STEMI) of inferoposterior wall, subsequent episode of care	410.32	I21.19
ST elevation myocardial infarction (STEMI) of inferior wall, initial episode of care	410.41	I21.19
ST elevation myocardial infarction (STEMI) of inferior wall, subsequent episode of care	410.42	I21.19
ST elevation myocardial infarction (STEMI) of inferoposterior wall, episode of care unspecified	410.3	I21.19

© 2021 Eberly LA et al. *JAMA Network Open*.

Transmural acute myocardial infarction of inferior wall, initial hospitalization	410.41	I21.19
Transmural acute inferior myocardial infarction, initial hospitalization	410.41	I21.19
Transmural myocardial infarction of inferolateral wall, initial hospitalization	410.21	I21.19
ST segment elevation myocardial infarction (STEMI) of inferolateral wall, subsequent episode of care	410.22	I21.19
ST segment elevation myocardial infarction (STEMI) of inferoposterior wall, subsequent episode of care	410.32	I21.19
Acute myocardial infarction of other inferior wall	410.4	I21.19
Acute Q wave myocardial infarction of inferior wall	410.4	I21.19
Acute Q wave myocardial infarction of inferolateral wall	410.2	I21.19
Acute myocardial infarction of inferior wall, subsequent to initial episode of care	410.42	I21.19
Acute myocardial infarction of inferolateral wall, subsequent to initial episode of care	410.22	I21.19
Acute myocardial infarction of inferoposterior wall, subsequent to initial episode of care	410.32	I21.19
ST segment elevation myocardial infarction (STEMI) of inferolateral wall, subsequent to initial episode of care	410.22	I21.19
ST segment elevation myocardial infarction (STEMI) of inferoposterior wall, subsequent to initial episode of care	410.32	I21.19
ST-segment elevation myocardial infarction (STEMI) of inferior wall	410.4	I21.19
Myocardial infarction of inferior wall	410.4	I21.19
Inferior ST segment elevation	410.4	I21.19

© 2021 Eberly LA et al. *JAMA Network Open*.

Acute ST elevation myocardial infarction (STEMI) involving other coronary artery of inferior wall	410.4	I21.19
ST elevation myocardial infarction (STEMI) involving other coronary artery of inferior wall	410.4	I21.19
Myocardial infarction involving other coronary artery of inferior wall	410.4	I21.19
Acute myocardial infarction involving other coronary artery of inferior wall	410.4	I21.19
Inferolat AMI, init episd	410.21	I21.19
Inferopos AMI, episd	410.3	I21.19
Inferopos AMI, init episd	410.31	I21.19
Infer AMI NEC, init episd	410.41	I21.19
AMI inferolateral,subseq	410.22	I21.19
AMI inferopost, subseq	410.32	I21.19
AMI inferior wall,subseq	410.42	I21.19
Acute myocardial infarction of other inferior wall, subsequent to initial episode of care	410.42	I21.19
ST elevation (STEMI) myocardial infarction involving other coronary artery of inferior wall	410.4	I21.19
Acute ST elevation myocardial infarction (STEMI) of inferior wall	410.4	I21.19
Acute ST elevation myocardial infarction (STEMI) of inferior wall involving right ventricle	410.4	I21.19
Acute myocardial infarction of inferior wall involving right ventricle	410.4	I21.19
STEMI involving oth coronary artery of inferior wall	410.4	I21.19
ST elevation (STEMI) myocardial infarction involving other coronary artery of inferior wall		I21.19
St elevation (stemi) myocardial infarction of other sites		I21.2

© 2021 Eberly LA et al. *JAMA Network Open*.

ST elevation myocardial infarct involv left circumflex coronary artery	410.81	I21.21
ST elevation myocardial infarction involving left circumflex coronary artery	410.81	I21.21
ST elevation myocardial infarction (STEMI) involving left circumflex coronary artery in recovery phase	410.82	I21.21
ST elevation myocardial infarction (STEMI) involving left circumflex coronary artery with complication	410.8	I21.21
Acute Q wave myocardial infarction involving left circumflex coronary artery	410.8	I21.21
Acute ST elevation myocardial infarction (STEMI) involving left circumflex coronary artery	410.8	I21.21
Acute non-Q wave ST elevation myocardial infarction (STEMI) involving left circumflex coronary artery	410.8	I21.21
Widespread acute non-Q wave ST elevation myocardial infarction (STEMI) involving left circumflex coronary artery	410.8	I21.21
Widespread acute Q wave ST elevation myocardial infarction (STEMI) involving left circumflex coronary artery	410.8	I21.21
Non-Q wave ST elevation myocardial infarction (STEMI) involving left circumflex coronary artery	410.7	I21.21
Silent ST elevation myocardial infarction (STEMI) involving left circumflex coronary artery	410.1	I21.21
Acute myocardial infarction involving left circumflex coronary artery	410.81	I21.21
Myocardial infarction involving left circumflex coronary artery	410.8	I21.21
Acute ST elevation myocardial infarction (STEMI) involving left circumflex coronary artery without development of Q waves	410.8	I21.21

© 2021 Eberly LA et al. *JAMA Network Open*.

Widespread acute ST elevation myocardial infarction (STEMI) involving left circumflex coronary artery without development of Q waves	410.8	I21.21
ST elevation myocardial infarction (STEMI) involving left circumflex coronary artery without development of Q waves	410.7	I21.21
ST elevation (STEMI) myocardial infarction involving left circumflex coronary artery	410.81	I21.21
STEMI involving left circumflex coronary artery	410.81	I21.21
ST elevation (STEMI) myocardial infarction involving left circumflex coronary artery		I21.21
Acute myocardial infarction of other lateral wall, episode of care unspecified	410.5	I21.29
Acute myocardial infarction of other lateral wall, initial episode of care	410.51	I21.29
Acute myocardial infarction of other lateral wall, subsequent episode of care	410.52	I21.29
Acute myocardial infarction, true posterior wall infarction, episode of care unspecified	410.6	I21.29
Acute myocardial infarction, true posterior wall infarction, initial episode of care	410.61	I21.29
Acute myocardial infarction of other specified sites, episode of care unspecified	410.8	I21.29
Acute myocardial infarction of other specified sites, initial episode of care	410.81	I21.29
Acute myocardial infarction of other specified sites, subsequent episode of care	410.82	I21.29
Acute myocardial infarction of lateral wall	410.5	I21.29
Acute myocardial infarction of apical-lateral wall	410.5	I21.29
Acute myocardial infarction of basal-lateral wall	410.5	I21.29

© 2021 Eberly LA et al. *JAMA Network Open*.

Acute myocardial infarction of high lateral wall	410.5	I21.29
Acute myocardial infarction of posterolateral wall	410.5	I21.29
True posterior wall infarction	410.6	I21.29
Acute myocardial infarction of posterobasal wall	410.6	I21.29
Acute myocardial infarction of septum	410.8	I21.29
Acute myocardial infarction of septum alone	410.8	I21.29
True posterior wall infarction, initial episode of care	410.61	I21.29
True posterior wall infarction, subsequent episode of care	410.62	I21.29
Acute myocardial infarction, true posterior wall infarction	410.6	I21.29
Myocardial infarction (lateral wall)	410.5	I21.29
Acute myocardial infarction of lateral wall, initial episode of care	410.51	I21.29
True posterior myocardial infarction	410.6	I21.29
Posterior MI	410.6	I21.29
Acute MI lateral subsequent episode care	410.52	I21.29
ST elevation myocardial infarction (STEMI) of lateral wall	410.5	I21.29
ST elevation myocardial infarction (STEMI) of true posterior wall	410.6	I21.29
True posterior infarct	410.6	I21.29
Basal-lateral infarction	410.5	I21.29
High lateral infarction	410.5	I21.29
Posterobasal infarction	410.6	I21.29
Posterolateral infarction	410.5	I21.29
Septal infarction	410.8	I21.29
AMI lateral wall	410.5	I21.29
Strictly posterior infarction	410.6	I21.29
Acute lateral myocardial infarction	410.5	I21.29
Acute posterior myocardial infarction	410.6	I21.29

© 2021 Eberly LA et al. *JAMA Network Open*.

F/u of acute myocardial infarction of other specified sites	410.82	I21.29
F/u of myocardial infarction of right ventricle	410.82	I21.29
Follow-up of myocardial infarction of right ventricle	410.82	I21.29
Myocardial infarction, lateral wall, initial care	410.51	I21.29
Myocardial infarction, lateral wall, subsequent care	410.52	I21.29
Myocardial infarction, true posterior wall, initial care	410.61	I21.29
Myocardial infarction, true posterior wall, subsequent care	410.62	I21.29
Myocardial infarction, true posterior wall	410.6	I21.29
Myocardial infarction, posterobasal	410.6	I21.29
Myocardial infarction, septal, acute	410.8	I21.29
Myocardial infarction, lateral wall, acute, initial episode	410.51	I21.29
Myocardial infarction, posterior wall	410.6	I21.29
Myocardial infarction, posterior wall, initial episode	410.61	I21.29
Apical myocardial infarction	410.8	I21.29
Lateral myocardial infarction	410.5	I21.29
Myocardial infarction of lateral wall	410.5	I21.29
Acute MI, true posterior wall	410.6	I21.29
Acute MI, lateral wall	410.5	I21.29
Acute MI, lateral wall, initial episode of care	410.51	I21.29
Acute MI, lateral wall, subsequent episode of care	410.52	I21.29
Acute MI, true posterior wall, initial episode of care	410.61	I21.29
Acute MI, true posterior wall, subsequent episode of care	410.62	I21.29
Myocardial infarction of septum	410.8	I21.29
Septal myocardial infarction	410.8	I21.29

© 2021 Eberly LA et al. *JAMA Network Open*.

Acute MI, other lateral wall, subsequent episode of care	410.52	I21.29
Acute MI, other specified site, initial episode of care	410.81	I21.29
Acute MI, other specified site, subsequent episode care	410.82	I21.29
Acute MI, other specified site	410.8	I21.29
Acute MI, other lateral wall	410.5	I21.29
Acute MI, other lateral wall, initial episode of care	410.51	I21.29
Myocardial infarction, apical	410.8	I21.29
Acute septal myocardial infarction	410.8	I21.29
Acute myocardial infarction of other sites, initial episode of care	410.81	I21.29
Acute lateral wall myocardial infarction	410.5	I21.29
Lateral wall myocardial infarction	410.5	I21.29
Acute true posterior wall myocardial infarction	410.6	I21.29
Myocardial infarction of posterolateral wall	410.5	I21.29
Posterolateral myocardial infarction	410.5	I21.29
Acute myocardial infarction of lateral wall, subsequent episode of care	410.52	I21.29
ST elevation myocardial infarction (STEMI) of lateral wall, initial episode of care	410.51	I21.29
ST elevation myocardial infarction (STEMI) of lateral wall, subsequent episode of care	410.52	I21.29
ST elevation myocardial infarction (STEMI) of true posterior wall, initial episode of care	410.61	I21.29
ST elevation myocardial infarction (STEMI) of true posterior wall, subsequent episode of care	410.62	I21.29
Transmural myocardial infarction of lateral wall, initial hospitalization	410.51	I21.29

© 2021 Eberly LA et al. *JAMA Network Open*.

Mural thrombus of cardiac apex with acute MI	410.8	I21.29
Mural thrombus of cardiac apex with acute myocardial infarction	410.8	I21.29
Left ventricular apical thrombus with acute myocardial infarction	410.8	I21.29
Left ventricular apical thrombus with acute MI	410.8	I21.29
Left ventricular mural thrombus with acute myocardial infarction	410.8	I21.29
Left ventricular mural thrombus with acute MI	410.8	I21.29
Left ventricular thrombosis with acute MI	410.8	I21.29
Left ventricular thrombus with acute MI	410.8	I21.29
Right ventricular mural thrombus with acute myocardial infarction	410.8	I21.29
Thrombus of right ventricle with acute MI	410.8	I21.29
Mural thrombus of left ventricle with acute MI	410.8	I21.29
Mural thrombus of left ventricular apex with acute MI	410.8	I21.29
Mural thrombus of right ventricle with acute MI	410.8	I21.29
Apical mural thrombus with acute MI	410.8	I21.29
Right ventricular mural thrombus with acute MI	410.8	I21.29
RV (right ventricular) mural thrombus with acute MI	410.8	I21.29
Right ventricular thrombus with acute MI	410.8	I21.29
LV (left ventricular) mural thrombus with acute MI	410.8	I21.29
Ventricular mural thrombus with acute myocardial infarction	410.8	I21.29
Ventricular mural thrombus with acute MI	410.8	I21.29
Right ventricular apical thrombus with acute myocardial infarction	410.8	I21.29

© 2021 Eberly LA et al. *JAMA Network Open*.

Mural thrombus of right ventricle apex with acute MI	410.8	I21.29
Right ventricular apical thrombus with acute MI	410.8	I21.29
Acute thrombus of left ventricle with acute MI	410.8	I21.29
Acute myocardial infarction of other lateral wall	410.5	I21.29
Acute myocardial infarction of other specified sites	410.8	I21.29
Acute Q wave myocardial infarction of lateral wall	410.5	I21.29
Acute myocardial infarction of lateral wall, subsequent to initial episode of care	410.52	I21.29
Acute myocardial infarction of other lateral wall, subsequent to initial episode of care	410.52	I21.29
True posterior wall infarction, subsequent to initial episode of care	410.62	I21.29
Myocardial infarction of true posterior wall	410.6	I21.29
Acute myocardial infarction involving other coronary artery	410.8	I21.29
ST elevation myocardial infarction (STEMI) involving other coronary artery	410.1	I21.29
Acute ST elevation myocardial infarction (STEMI) involving other coronary artery	410.1	I21.29
Myocardial infarction involving other coronary artery	410.8	I21.29
Latrl AMI NEC, episd	410.5	I21.29
Latrl AMI NEC, init episd	410.51	I21.29
Posterior AMI, episd	410.6	I21.29
Posterior AMI, init episd	410.61	I21.29
AMI NEC, initial episd	410.81	I21.29
AMI NEC, subsequent episd	410.82	I21.29
AMI lateral NEC, subseq	410.52	I21.29
True post infarct,subseq	410.62	I21.29

© 2021 Eberly LA et al. *JAMA Network Open*.

Acute myocardial infarction of other specified sites, subsequent to initial episode of care	410.82	I21.29
Acute ST elevation myocardial infarction (STEMI) of septum	410.8	I21.29
Acute ST elevation myocardial infarction (STEMI) of posterior wall	410.6	I21.29
Acute ST elevation myocardial infarction (STEMI) of posterobasal wall	410.6	I21.29
Acute ST elevation myocardial infarction (STEMI) of posterolateral wall	410.5	I21.29
Acute ST elevation myocardial infarction (STEMI) of lateral wall	410.5	I21.29
Encounter for follow-up of myocardial infarction of right ventricle	410.82	I21.29
ST elevation (STEMI) myocardial infarction involving other sites	410.8	I21.29
ST elevation (STEMI) myocardial infarction involving other sites		I21.29
Myocardial necrosis syndrome	410.9	I21.3
Acute ST-segment elevation myocardial infarction	410.9	I21.3
ST elevation myocardial infarction (STEMI)	410.9	I21.3
ST elevation myocardial infarction (STEMI), initial episode of care	410.91	I21.3
ST elevation myocardial infarction (STEMI), subsequent episode of care	410.92	I21.3
STEMI (ST elevation myocardial infarction)	410.9	I21.3
Acute transmural myocardial infarction	410.9	I21.3
Acute transmural MI	410.9	I21.3
Acute ST-elevation myocardial infarction	410.9	I21.3
Myocardial necrosis	410.9	I21.3
Acute ST segment elevation myocardial infarction	410.9	I21.3
ST elevation MI (STEMI)	410.9	I21.3

© 2021 Eberly LA et al. *JAMA Network Open*.

ST elevation (STEMI) myocardial infarction	410.9	I21.3
Acute ST segment elevation MI	410.9	I21.3
Transmural myocardial infarction, initial hospitalization	410.91	I21.3
Widespread acute Q wave myocardial infarction	410.9	I21.3
Acute Q wave myocardial infarction	410.9	I21.3
ST elevation myocardial infarction (STEMI), subsequent to initial episode of care	410.92	I21.3
Acute ST elevation myocardial infarction	410.9	I21.3
ST elevation myocardial infarction (STEMI) in recovery phase	410.92	I21.3
ST elevation myocardial infarction (STEMI) with complication	410.92	I21.3
Acute non-Q wave ST elevation myocardial infarction (STEMI)	410.7	I21.3
Widespread acute non-Q wave ST elevation myocardial infarction (STEMI)	410.7	I21.3
Widespread acute Q wave ST elevation myocardial infarction (STEMI)	410.9	I21.3
Non-Q wave ST elevation myocardial infarction (STEMI)	410.9	I21.3
Silent ST elevation myocardial infarction (STEMI)	410.9	I21.3
Acute ST elevation myocardial infarction (STEMI)	410.9	I21.3
Electrocardiogram suggestive of ST elevation myocardial infarction (STEMI)	410.9	I21.3
ST elevation myocardial infarction (STEMI), unspecified artery	410.9	I21.3
Acute ST elevation myocardial infarction (STEMI), unspecified artery	410.9	I21.3
Acute ST elevation myocardial infarction (STEMI) due to occlusion of left coronary artery	410.9	I21.3

© 2021 Eberly LA et al. *JAMA Network Open*.

Acute ST elevation myocardial infarction (STEMI) due to occlusion of right coronary artery	410.9	I21.3
Electrocardiography suggestive of ST elevation myocardial infarction (STEMI)	410.9	I21.3
Acute ST elevation myocardial infarction (STEMI) without development of Q waves	410.7	I21.3
Widespread acute ST elevation myocardial infarction (STEMI) without development of Q waves	410.7	I21.3
ST elevation myocardial infarction (STEMI) without development of Q waves	410.9	I21.3
ST elevation (STEMI) myocardial infarction of unspecified site	410.9	I21.3
ST elevation (STEMI) myocardial infarction of unspecified site		I21.3
Acute myocardial infarction, subendocardial infarction, episode of care unspecified	410.7	I21.4
Acute myocardial infarction, subendocardial infarction, initial episode of care	410.71	I21.4
Acute myocardial infarction, subendocardial infarction, subsequent episode of care	410.72	I21.4
Acute subendocardial infarction	410.7	I21.4
Acute nontransmural infarction	410.7	I21.4
Subendocardial infarction	410.7	I21.4
Subendocardial infarction, initial episode of care	410.71	I21.4
Subendocardial infarction, subsequent episode of care	410.72	I21.4
Acute myocardial infarction, subendocardial infarction	410.7	I21.4
Subendocardial myocardial infarction	410.7	I21.4
SEMI (subendocardial myocardial infarction)	410.7	I21.4

© 2021 Eberly LA et al. *JAMA Network Open*.

Non-Q wave myocardial infarction	410.7	I21.4
Subendocardial MI first episode care	410.71	I21.4
Subendocardial MI subsequent episode care	410.72	I21.4
Acute non-ST-segment elevation myocardial infarction	410.7	I21.4
Acute non-ST-elevation myocardial infarction	410.7	I21.4
Non-ST elevation myocardial infarction (NSTEMI)	410.7	I21.4
Subendocardial infarct	410.7	I21.4
Nontransmural infarction	410.7	I21.4
NSTEMI (non-ST elevation myocardial infarction)	410.7	I21.4
NTEMI (non-ST elevation myocardial infarction)	410.7	I21.4
Non-ST elevation myocardial infarction (NSTEMI), initial care episode	410.71	I21.4
Non-ST elevation myocardial infarction, subsequent care episode	410.72	I21.4
Acute non Q wave myocardial infarction	410.7	I21.4
Acute non Q wave myocardial infarction, initial episode of care	410.71	I21.4
Acute non Q wave MI (myocardial infarction), initial episode of care	410.71	I21.4
F/u for non Q wave myocardial infarction	410.72	I21.4
Follow-up for non Q wave myocardial infarction	410.72	I21.4
Acute subendocardial infarction, initial episode of care	410.71	I21.4
Acute subendocardial infarction, subsequent episode of care	410.72	I21.4
Acute subendocardial myocardial infarction of anterior wall	410.7	I21.4
Acute subendocardial myocardial infarction of inferior wall	410.7	I21.4

© 2021 Eberly LA et al. *JAMA Network Open*.

Non-STEMI (non-ST elevated myocardial infarction)	410.7	I21.4
Non-ST elevated myocardial infarction (non-STEMI)	410.7	I21.4
NSTEMI (non-ST elevated myocardial infarction)	410.7	I21.4
Myocardial infarction, subendocardial	410.7	I21.4
Myocardial infarction, subendocardial, subsequent care	410.72	I21.4
Acute non Q wave myocardial infarction, subsequent episode of care	410.72	I21.4
Acute non Q wave MI (myocardial infarction), subsequent episode	IMO0001	I21.4
Myocardial infarction, nontransmural	410.7	I21.4
Myocardial infarction, subendocardial, initial episode	410.71	I21.4
Acute non-Q wave anterolateral myocardial infarction	410.7	I21.4
Acute non-Q wave myocardial infarction of anterolateral wall	410.7	I21.4
NSTEMI, initial episode of care	410.71	I21.4
Non-ST elevation myocardial infarction, initial hospitalization	410.71	I21.4
Non-Q wave myocardial infarction, initial hospitalization	410.71	I21.4
Non-Q wave myocardial infarction, initial episode of care	410.71	I21.4
Non-Q wave myocardial infarction, subsequent episode of care	410.72	I21.4
Nontransmural acute myocardial infarction of inferior wall	410.71	I21.4
Acute nontransmural inferior myocardial infarction	410.71	I21.4
Nontransmural inferior myocardial infarction, initial hospitalization	410.71	I21.4
Nontransmural myocardial infarction, initial hospitalization	410.71	I21.4

© 2021 Eberly LA et al. *JAMA Network Open*.

Acute non-Q wave infarction	410.7	I21.4
Acute non-ST segment elevation myocardial infarction	410.7	I21.4
Acute MI, subendocardial	410.7	I21.4
Non-ST elevation MI (NSTEMI)	410.7	I21.4
Non-Q wave infarction	410.7	I21.4
Acute MI, subendocardial, initial episode of care	410.71	I21.4
Acute MI, subendocardial, subsequent episode of care	410.72	I21.4
Non-ST elevation (NSTEMI) myocardial infarction	410.7	I21.4
MI, acute, non ST segment elevation	410.7	I21.4
Acute subendocardial MI of anterior wall	410.7	I21.4
Acute subendocardial MI of inferior wall	410.7	I21.4
Non-Q wave myocardial infarction	410.7	I21.4
Non Q wave myocardial infarction	410.7	I21.4
Non-Q wave myocardial infarction of anterior wall	410.7	I21.4
Non-Q wave myocardial infarction of true posterior wall	410.7	I21.4
Non-Q wave myocardial infarction of lateral wall	410.7	I21.4
Non-Q wave myocardial infarction of inferoposterior wall	410.7	I21.4
Non-Q wave myocardial infarction of inferolateral wall	410.7	I21.4
Non-Q wave myocardial infarction of inferior wall	410.7	I21.4
Non-Q wave myocardial infarction of anteroseptal wall	410.7	I21.4
Non-Q wave myocardial infarction of anterolateral wall	410.7	I21.4
Anterior subendocardial MI	410.7	I21.4
Nontransmural apical myocardial infarction, initial hospitalization	410.71	I21.4

© 2021 Eberly LA et al. *JAMA Network Open*.

Non-ST elevation myocardial infarction (NSTEMI), subendocardial infarction	410.7	I21.4
Non-ST elevation myocardial infarction (NSTEMI), subendocardial infarction, episode of care unspecified	410.7	I21.4
Non-ST elevation myocardial infarction (NSTEMI), subendocardial infarction, initial episode of care	410.71	I21.4
Non-ST elevation myocardial infarction (NSTEMI), subendocardial infarction, subsequent episode of care	410.72	I21.4
Non-ST elevation myocardial infarction (NSTEMI), initial episode of care	410.71	I21.4
Non-ST elevation myocardial infarction (NSTEMI), subsequent episode of care	410.72	I21.4
Nontransmural myocardial infarction of inferior wall, initial hospitalization	410.71	I21.4
Nontransmural myocardial infarction of inferolateral wall, initial hospitalization	410.71	I21.4
Nontransmural anteroapical myocardial infarction, initial hospitalization	410.71	I21.4
Nontransmural anteroseptal myocardial infarction, initial hospitalization	410.71	I21.4
Nontransmural myocardial infarction of lateral wall, initial hospitalization	410.71	I21.4
Nontransmural myocardial infarction of anterolateral wall, initial hospitalization	410.71	I21.4
Nontransmural acute myocardial infarction of lateral wall	410.71	I21.4
Acute nontransmural myocardial infarction of anterior wall	410.7	I21.4
Acute non-Q wave myocardial infarction of inferior wall	410.7	I21.4
Acute non-Q wave myocardial infarction of lateral wall	410.7	I21.4
Widespread acute non-Q wave infarction	410.7	I21.4

© 2021 Eberly LA et al. *JAMA Network Open*.

Acute non-Q wave myocardial infarction of inferolateral wall	410.7	I21.4
Acute non Q wave myocardial infarction, subsequent to initial episode of care	410.72	I21.4
Acute subendocardial infarction, subsequent to initial episode of care	410.72	I21.4
Non-Q wave myocardial infarction, subsequent to initial episode of care	410.72	I21.4
Non-ST elevation subendocardial infarction, subsequent to initial episode of care	410.72	I21.4
Non-ST elevated myocardial infarction	410.7	I21.4
Widespread acute non-Q wave myocardial infarction	410.7	I21.4
Widespread acute non-Q wave non-ST elevation myocardial infarction	410.7	I21.4
Acute non-Q wave non-ST elevation myocardial infarction	410.7	I21.4
Non-Q wave non-ST elevation myocardial infarction	410.7	I21.4
Silent non-ST elevation myocardial infarction	410.9	I21.4
Recent subendocardial infarction	410.7	I21.4
Subendo infrc, episd	410.7	I21.4
Subendo infrc, init episd	410.71	I21.4
Subendo infarct, subseq	410.72	I21.4
Widespread acute non-Q wave non-ST elevation myocardial infarction (NSTEMI)	410.7	I21.4
Encounter for follow-up of non-Q wave myocardial infarction	410.72	I21.4
Subsequent encounter to intial episode of care for acute non-Q wave myocardial infarction	410.72	I21.4
Silent non-ST elevation myocardial infarction (NSTEMI)	410.9	I21.4
Acute non-Q wave non-ST elevation myocardial infarction (NSTEMI)	410.7	I21.4

© 2021 Eberly LA et al. *JAMA Network Open*.

Non-Q wave non-ST elevation myocardial infarction (NSTEMI)	410.7	I21.4
Acute non-ST elevation myocardial infarction (NSTEMI)	410.7	I21.4
Nontransmural myocardial infarction	410.7	I21.4
Subsequent encounter to initial episode of care for acute non-Q wave myocardial infarction	410.72	I21.4
Non-ST elevation (NSTEMI) myocardial infarction		I21.4
Acute infarction of papillary muscle	410.8	I21.9
Acute myocardial infarction of atrium	410.8	I21.9
Acute papillary muscle infarction	410.8	I21.9
Papillary muscle infarction	410.8	I21.9
Atrial infarction	410.8	I21.9
Myocardial infarction, papillary muscle, acute	410.8	I21.9
Myocardial infarction, atrial, acute	410.8	I21.9
AMI NEC, episd	410.9	I21.9
Acute myocardial infarction, unspecified site, episode of care unspecified	410.9	I21.9
Acute myocardial infarction, unspecified site, initial episode of care	410.91	I21.9
Acute myocardial infarction, unspecified site, subsequent episode of care	410.92	I21.9
Cardiac infarction	410.9	I21.9
Acute myocardial infarction	410.9	I21.9
Heart attack	410.9	I21.9
Myocardial infarct	410.9	I21.9
Myocardial infarction	410.9	I21.9
AMI (acute myocardial infarction)	410.9	I21.9
Acute myocardial infarction, initial episode of care	410.91	I21.9
Acute myocardial infarction, subsequent episode of care	410.92	I21.9
Acute MI	410.9	I21.9
MI (myocardial infarction)	410.9	I21.9

© 2021 Eberly LA et al. *JAMA Network Open*.

Myocardial infarction acute	410.9	I21.9
Myocardial infarction syndrome	410.9	I21.9
Acute myocardial infarction of right ventricle	410.8	I21.9
Myocardial infarction, initial care	410.91	I21.9
Silent myocardial infarction	410.9	I21.9
Myocardial infarction, silent	410.9	I21.9
Demand myocardial infarction	410.9	I21.9
Myocardial infarction, demand	410.9	I21.9
Myocardial infarction, acute, care	410.9	I21.9
Myocardial infarction, acute, initial episode of care	410.91	I21.9
Acute right ventricular myocardial infarction	410.8	I21.9
Acute MI, initial	410.91	I21.9
Acute MI, subsequent	410.92	I21.9
Acute myocardial infarction involving left coronary artery	410.9	I21.9
Myocardial infarction within last four weeks	410.9	I21.9
Acute myocardial infarction of right ventricle, subsequent episode of care	410.92	I21.9
Acute myocardial infarction, unspecified site	410.9	I21.9
Myocardial infarction with complication	410.9	I21.9
Acute widespread myocardial infarction	410.9	I21.9
Myocardial infarction in recovery phase	410.92	I21.9
First myocardial infarction	410.91	I21.9
Pain due to myocardial infarction	410.9	I21.9
Acute myocardial infarction of right ventricle, subsequent to initial episode of care	410.92	I21.9
Acute myocardial infarction, subsequent to initial episode of care	410.92	I21.9
Myocardial infarction during current hospitalization	410.9	I21.9
Death due to acute myocardial infarction	410.9	I21.9

© 2021 Eberly LA et al. *JAMA Network Open*.

Admitted for acute myocardial infarction	410.91	I21.9
AMI NOS, subsequent episd	410.92	I21.9
AMI NOS, unspecified	410.9	I21.9
Myocardial infarction less than 4 weeks ago	410.9	I21.9
Subsequent encounter to initial episode of care for acute myocardial infarction of right ventricle	410.92	I21.9
Occlusion of coronary artery with myocardial infarction	410.9	I21.9
Coronary artery occlusion with myocardial infarction	410.9	I21.9
Acute myocardial infarction, unspecified		I21.9
Coronary artery rupture	410.9	I21.9
Ruptured, artery, coronary	410.9	I21.9
Ruptured, coronary artery	410.9	I21.9
Cardiovascular accident	429.2	I21.9
Cardiac necrosis	410.9	I21.9
Mural thrombus of heart with acute myocardial infarction	410.9	I21.9
Mural thrombus of heart with acute MI	410.9	I21.9
History of acute myocardial infarction within last month	410.9	I21.9
History of myocardial infarction within last month	410.9	I21.9
Coronary artery thrombosis with myocardial infarction	410.9	I21.9
Acute myocardial infarction with rupture of free wall	410.9	I21.9, I23.3
Pericarditis secondary to acute myocardial infarction	410.90, 420.0	I21.9, I32
Drug-related myocardial necrosis syndrome	410.90, E947.9	I21.9, T50.905A
Myocardial infarction with cardiac rehabilitation	410.90, V57.89	I21.9, Z51.89
Other type of myocardial infarction		I21.A

© 2021 Eberly LA et al. *JAMA Network Open*.

Non-ST elevation myocardial infarction (NSTEMI) due to mismatch of myocardial oxygen supply and demand	410.7	I21.A1
Non-ST elevation myocardial infarction (NSTEMI), type 2	410.7	I21.A1
Type 2 myocardial infarction	410.9	I21.A1
Myocardial infarction type 2	410.9	I21.A1
Myocardial infarction type 2		I21.A1
Other type of myocardial infarction	410.9	I21.A9
Other myocardial infarction type	410.9	I21.A9
Other myocardial infarction type		I21.A9
Subsequent ST elevation (STEMI) and non-ST elevation (NSTEMI) myocardial infarction		I22
Additional acute myocardial infarction (anterior wall)	410.1	I22.0
Subsequent ST elevation (STEMI) myocardial infarction of anterior wall	410.11	I22.0
Subsequent ST elevation (STEMI) myocardial infarction of anterior wall within 4 weeks of initial infarction	410.1	I22.0
Subsequent ST elevation myocardial infarction (STEMI) of anterior wall within 4 weeks of initial infarction	410.1	I22.0
Subsequent ST elevation (STEMI) myocardial infarction of anterior wall (CODE)	410.11	I22.0
Subsequent ST elevation (STEMI) myocardial infarction of anterior wall		I22.0
Additional acute myocardial infarction (inferior wall)	410.4	I22.1
Additional heart attack (inferior wall)	410.4	I22.1
Subsequent myocardial infarction of inferior wall	410.4	I22.1
Subsequent ST elevation (STEMI) myocardial infarction of inferior wall	410.4	I22.1

© 2021 Eberly LA et al. *JAMA Network Open*.

Subsequent myocardial infarction of inferior wall within 4 weeks of initial infarction	410.4	I22.1
Subsequent ST elevation myocardial infarction (STEMI) involving right coronary artery within 4 weeks of initial infarction	410.11, 410.92	I22.1
Subsequent ST elevation (STEMI) myocardial infarction of inferior wall (CODE)	410.4	I22.1
Subsequent ST elevation (STEMI) myocardial infarction of inferior wall		I22.1
Acute non-ST-elevation MI following previous MI	410.7	I22.2
Subsequent non-ST elevation (NSTEMI) myocardial infarction	410.7	I22.2
Subsequent non-ST elevation (NSTEMI) myocardial infarction within 4 weeks of initial infarction	410.7	I22.2
Subsequent non-ST elevation myocardial infarction within 4 weeks of initial infarction	410.7	I22.2
Subsequent non-ST elevation myocardial infarction (NSTEMI) within 4 weeks of initial infarction	410.7	I22.2
Subsequent non-ST elevation (NSTEMI) myocardial infarction (CODE)	410.7	I22.2
Subsequent non-ST elevation (NSTEMI) myocardial infarction		I22.2
Additional acute myocardial infarction (posterior wall)	410.6	I22.8
Additional heart attack (posterior wall)	410.6	I22.8
Additional acute myocardial infarction (lateral wall)	410.5	I22.8
Additional heart attack (lateral wall)	410.5	I22.8
Additional acute myocardial infarction (subendocardial)	410.7	I22.8
Additional heart attack (subendocardial)	410.7	I22.8

© 2021 Eberly LA et al. *JAMA Network Open*.

Acute subendocardial infarction following prior myocardial infarction	410.7	I22.8
Subsequent ST elevation myocardial infarction (STEMI) involving left anterior descending (LAD) coronary artery within 4 weeks of initial infarction	410.11, 410.92	I22.8
Subsequent ST elevation myocardial infarction (STEMI) involving left circumflex coronary artery within 4 weeks of initial infarction	410.81, 410.92	I22.8
Subsequent ST elevation myocardial infarction (STEMI) involving left main coronary artery within 4 weeks of initial infarction	410.11, 410.92	I22.8
Subsequent ST elevation (STEMI) myocardial infarction of other sites	410.8	I22.8
Subsequent ST elevation (STEMI) myocardial infarction of other sites		I22.8
Subsequent ST elevation (STEMI) myocardial infarction	410.9	I22.9
Subsequent ST elevation (STEMI) myocardial infarction of unspecified site	410.9	I22.9
Additional acute myocardial infarction	410.9	I22.9
Additional heart attack	410.9	I22.9
Myocardial infarction, subsequent	410.9	I22.9
Subsequent myocardial infarction	410.9	I22.9
Subsequent myocardial infarction within 4 weeks of initial infarction	410.9	I22.9
Subsequent ST elevation myocardial infarction (STEMI) within 4 weeks of initial infarction	410.9	I22.9
Subsequent ST elevation (STEMI) myocardial infarction of unspecified site (CODE)	410.92	I22.9
Subsequent ST elevation (STEMI) myocardial infarction of unspecified site		I22.9

© 2021 Eberly LA et al. *JAMA Network Open*.

Certain current complications following ST elevation (STEMI) and non-ST elevation (NSTEMI) myocardial infarction (within the 28 day period)		I23
Hemopericardium as curr complication after acute myocardial infarction	429.79	I23.0
Hemopericardium as current complication following acute myocardial infarction	429.79	I23.0
Hemopericardium as current complication following AMI	429.79	I23.0
Hemopericardium as current complication following acute myocardial infarction		I23.0
Atrial septal defect as curr complic after acute myocardial infarction	429.71	I23.1
Atrial septal defect as current complication following acute myocardial infarction	429.71	I23.1
Atrial septal defect as current complication following AMI	429.71	I23.1
Atrial septal defect as current complication following acute myocardial infarction		I23.1
Ventric septal defect as curr complic after acute myocardial infarct	429.71	I23.2
Ventricular septal defect as current complication following acute myocardial infarction	429.71	I23.2
Ventricular septal defect as current comp following AMI	429.71	I23.2
Ventricular septal defect as current complication following acute myocardial infarction		I23.2
Rupture of heart	410.9	I23.3
Cardiac rupture	410.9	I23.3
Heart rupture	410.9	I23.3
Ruptured, cardiac	410.9	I23.3
Ruptured, heart	410.9	I23.3

© 2021 Eberly LA et al. *JAMA Network Open*.

Acute myocardial infarction with rupture of ventricle	410.9	I23.3
Acute myocardial infarction with ventricular rupture	410.9	I23.3
Free wall rupture	410.9	I23.3
Acute ventricular septal rupture	410.8	I23.3
Rupture of interventricular septum	410.8	I23.3
Ventricular septal rupture, acute	410.8	I23.3
Ventricular septal rupture	410.8	I23.3
Rupture of cardiac wall without hemopericardium as current complication following acute myocardial infarction	429.79	I23.3
Rupture of card wall w/o hemoperic as current comp fol AMI	429.79	I23.3
Rupture of cardiac wall without hemopericardium as current complication following acute myocardial infarction		I23.3
Rupture of chordae tendineae as current complication following acute myocardial infarction	429.5	I23.4
Rupture of chord tendne as current comp following AMI	429.5	I23.4
Rupture of chordae tendineae as current complication following acute myocardial infarction		I23.4
Ruptured papillary muscle complicating acute MI	429.6, 410.90	I23.5
Rupture of papillary muscle as current complication following acute myocardial infarction	429.6, 410.90	I23.5
Post-infarction mitral papillary muscle rupture	429.6	I23.5
Rupture of papillary muscle as current comp following AMI	429.6, 410.90	I23.5
Rupture of papillary muscle as current complication following acute myocardial infarction		I23.5

© 2021 Eberly LA et al. *JAMA Network Open*.

LV (left ventricular) mural thrombus following MI	429.79, 410.82	I23.6
Left ventricular apical thrombus following myocardial infarction	429.79, 410.82	I23.6
Left ventricular apical thrombus following MI	429.79, 410.82	I23.6
Left ventricular mural thrombus following MI	429.79, 410.82	I23.6
Left ventricular thrombosis following MI	429.79, 410.82	I23.6
Left ventricular thrombus following MI	429.79, 410.82	I23.6
Left ventricular mural thrombosis following myocardial infarction	429.79, 410.82	I23.6
Mural thrombus of left ventricle following MI	429.79, 410.82	I23.6
Mural thrombus of left ventricular apex following MI	429.79, 410.82	I23.6
Acute thrombus of left ventricle following MI	429.79, 410.82	I23.6
Mural thrombus of left ventricle following acute myocardial infarction	429.79, 410.82	I23.6
Ventricular thrombus following MI (myocardial infarction)	429.79, 410.92	I23.6
Post-infarction mural thrombus	429.79, 410.92	I23.6
Mural thrombus of cardiac apex following myocardial infarction	429.79, 410.92	I23.6
Mural thrombus of cardiac apex following MI	429.79, 410.92	I23.6
Right ventricular mural thrombosis following myocardial infarction	429.79, 410.82	I23.6
Thrombus of right ventricle following MI	429.79, 410.82	I23.6
Mural thrombus of right ventricle following MI	429.79, 410.82	I23.6
Apical mural thrombus following MI	429.79, 410.92	I23.6
Right ventricular mural thrombus following MI	429.79, 410.82	I23.6
RV (right ventricular) mural thrombus following MI	429.79, 410.82	I23.6

© 2021 Eberly LA et al. *JAMA Network Open*.

Right ventricular thrombus following MI	429.79, 410.82	I23.6
Ventricular mural thrombus following myocardial infarction	429.79, 410.92	I23.6
Ventricular mural thrombus following MI	429.79, 410.92	I23.6
Mural thrombus of heart following myocardial infarction	429.79, 410.92	I23.6
Mural thrombus of heart following MI	429.79, 410.92	I23.6
Right ventricular apical thrombus following myocardial infarction	429.79, 410.82	I23.6
Mural thrombus of right ventricle apex following MI	429.79, 410.82	I23.6
Right ventricular apical thrombus following MI	429.79, 410.82	I23.6
Post-infarction apical thrombus	429.79	I23.6
Post-infarction thrombus of left ventricle	429.79	I23.6
Post-infarction thrombus of right ventricle	429.79	I23.6
Thrombosis of atrium, auricular appendage, and ventricle as current complications following acute myocardial infarction (CODE)	429.79, 410.90	I23.6
Thrombosis of right atrium following myocardial infarction	429.79	I23.6
Thrombus of right atrial appendage following myocardial infarction	429.79	I23.6
Thrombosis of left atrium following myocardial infarction	429.79	I23.6
Thrombus of left atrial appendage following myocardial infarction	429.79	I23.6
Thrombsis of left atrial appendage following myocardial infarction	429.79	I23.6
Thrombsis of right atrial appendage following myocardial infarction	429.79	I23.6
Thrombosis of atrium, auricular appendage, and ventricle as current complications following acute myocardial infarction		I23.6

© 2021 Eberly LA et al. *JAMA Network Open*.

Atrial thrombus following MI	429.79, 410.90	I23.6, I21.3
Thrombosis of atrium, auricular appendage, and ventricle as current complications following acute myocardial infarction	429.79, 410.90	I23.6, I21.3
Atrial thrombus following myocardial infarction	429.79, 410.90	I23.6, I21.3
Thombos of atrium/auric append/ventr as current comp fol AMI	429.79, 410.90	I23.6, I21.3
Post-infarction angina	429.79, 413.9	I23.7
Postinfarction angina	429.79, 413.9	I23.7
Postinfarction angina		I23.7
Other certain sequelae of myocardial infarction, not elsewhere classified	429.79	I23.8
Certain sequelae of myocardial infarction	429.79	I23.8
AMI sequelae	429.79	I23.8
Myocardial infarction sequelae	429.79	I23.8
Myocardial infarction complications	429.79	I23.8
Complications of myocardial infarction	429.79	I23.8
Certain sequelae of myocardial infarction, not elsewhere classified	429.79	I23.8
Sequelae of myocardial infarction	429.79	I23.8
Complication of myocardial infarction	429.79	I23.8
Other AMI sequelae	429.79	I23.8
Other current complications following acute myocardial infarction	429.79	I23.8
Other current complications following acute myocardial infarction		I23.8
Acute complication of myocardial infarction	429.79	I23.8, I21.9
Other acute ischemic heart diseases		I24
Acute thrombus of right ventricle	410.9	I24.0
Coronary artery occlusion	410.9	I24.0
Coronary artery thrombosis	411.81	I24.0
Coronary thrombosis	411.81	I24.0
Coronary occlusion	410.9	I24.0

© 2021 Eberly LA et al. *JAMA Network Open*.

Coronary occlusion without myocardial infarction	411.81	I24.0
CT (coronary thrombosis)	411.81	I24.0
Thrombosis, arteries, coronary	411.81	I24.0
Acute coronary artery obstruction without myocardial infarction	411.81	I24.0
Acute coronary artery obstruction without MI	411.81	I24.0
Occlusion of left anterior descending artery	411.81	I24.0
Occlusion of LAD (left anterior descending) artery	411.81	I24.0
Coronary thrombosis not resulting in myocardial infarction	411.81	I24.0
Coronary occlusion, acute without myocardial infarct	411.81	I24.0
Coronary artery clot	411.81	I24.0
Occlusion of coronary artery, acute	410.9	I24.0
Acute coronary occlusion without myocardial infarction	411.81	I24.0
Acute coronary thrombosis not resulting in myocardial infarction	411.81	I24.0
Blockage of coronary artery of heart	410.9	I24.0
Ischemic heart disease due to coronary artery obstruction	411.81	I24.0
Left coronary artery occlusion	411.81	I24.0
Coronary artery occlusion without or not resulting in myocardial infarction	411.81	I24.0
Coronary artery thrombosis without or not resulting in myocardial infarction	411.81	I24.0
Occlusion of coronary artery	410.9	I24.0
Occlusion of coronary vein	411.81	I24.0
Occlusion of left anterior descending (LAD) artery	411.81	I24.0
Acute coronary thrombosis not resulting in myocardial infarction		I24.0
Left main coronary artery thrombosis	410.9	I24.0

© 2021 Eberly LA et al. *JAMA Network Open*.

Right main coronary artery thrombosis	410.9	I24.0
Acute thrombus of left ventricle	410.9	I24.0
Thrombosis of left circumflex coronary artery	410.9	I24.0
Left anterior descending coronary artery thrombosis	410.9	I24.0
Left anterior descending (LAD) coronary artery thrombosis	410.9	I24.0
Occlusion of right coronary artery	410.9	I24.0
RCA occlusion	410.9	I24.0
Right coronary artery occlusion	410.9	I24.0
Postmyocardial infarction syndrome	411	I24.1
Dressler's syndrome	411	I24.1
Postmyocardial infarction pericarditis	411	I24.1
Dressler syndrome	411	I24.1
Myocardial postinfarction syndrome	411	I24.1
Post MI syndrome	411	I24.1
Post myocardial infarction syndrome	411	I24.1
Pericarditis; post-MI	411	I24.1
Post-cardiac injury syndrome	411	I24.1
Post-MI pericarditis	411	I24.1
Postcardiac injury pericarditis	411	I24.1
Post-infarction pericarditis	411	I24.1
Dressler's syndrome		I24.1
Postmyocardial infarction syndrome following coronary artery bypass graft (CABG) surgery	411.0, 414.04	I24.1, I25.810
Dressler's syndrome post-CABG	411.0, 414.04	I24.1, I25.810
Dressler's syndrome following coronary artery bypass graft (CABG) surgery	411.0, 414.04	I24.1, I25.810
Other acute and subacute form of ischemic heart disease	411.89	I24.8
Subendocardial ischemia	411.89	I24.8
Microinfarct of heart	411.89	I24.8
Coronary insufficiency	411.89	I24.8
Microinfarct, heart	411.89	I24.8
Microinfarction of heart	411.89	I24.8

© 2021 Eberly LA et al. *JAMA Network Open*.

Coronary insufficiency, acute	411.89	I24.8
Acute coronary insufficiency	411.89	I24.8
Demand ischemia	411.89	I24.8
Acute and subacute ischemic heart disease	411.89	I24.8
Acute or subacute form of ischemic heart disease	411.89	I24.8
Coronary artery insufficiency	411.89	I24.8
Insufficiency, arterial, coronary	411.89	I24.8
Demand ischemia of myocardium	411.89	I24.8
Other acute and subacute forms of ischemic heart disease	411.89	I24.8
Other forms of acute ischemic heart disease	411.89	I24.8
Ischemia due to increased oxygen demand	411.89	I24.8
Other forms of acute ischemic heart disease		I24.8
Acute coronary syndrome	411.1	I24.9
ACS (acute coronary syndrome)	411.1	I24.9
Acute ischemic heart disease	410.9	I24.9
Acute coronary syndromes	411.1	I24.9
Ischemic heart disease, acute	410.9	I24.9
Coronary syndrome, acute	411.1	I24.9
Ischemia, myocardial, acute	410.9	I24.9
Acute myocardial ischemia	410.9	I24.9
Acute ischemic heart disease, unspecified	410.9	I24.9
Acute ischemic heart disease, unspecified		I24.9
Chronic ischemic heart disease		I25
Atherosclerotic heart disease of native coronary artery		I25.1
Coronary atherosclerosis of unspecified type of vessel, native or graft	414	I25.10
Coronary atherosclerosis of native coronary artery	414.01	I25.10
ASCVD (arteriosclerotic cardiovascular disease)	429.2, 440.9	I25.10
Cardiovascular arteriosclerosis	429.2, 440.9	I25.10

© 2021 Eberly LA et al. *JAMA Network Open*.

Cardiovascular degeneration with arteriosclerosis	429.2, 440.9	I25.10
Cardiovascular disease with arteriosclerosis	429.2, 440.9	I25.10
Cardiovascular sclerosis with arteriosclerosis	429.2, 440.9	I25.10
Coronary artery disease	414	I25.10
ASHD (arteriosclerotic heart disease)	414	I25.10
Coronary sclerosis	414	I25.10
Coronary arteriosclerosis	414	I25.10
Atherosclerotic heart disease	414	I25.10
Coronary artery atheroma	414	I25.10
Coronary stricture	414	I25.10
Coronary atherosclerosis of native coronary vessel	414.01	I25.10
Arteriosclerotic cardiovascular disease	429.2, 440.9	I25.10
Generalized arteriosclerosis	429.2, 440.9	I25.10
Generalized arteriosclerotic disease	429.2, 440.9	I25.10
Disease or syndrome of cardiovascular system	429.2	I25.10
Atherosclerotic coronary vascular disease	414	I25.10
Coronary disease	414	I25.10
Coronary disorder	414	I25.10
Coronary heart disease	414	I25.10
Coronary atheroma	414	I25.10
Coronary stenosis	414	I25.10
CAD (coronary atherosclerotic disease)	414	I25.10
Arteriosclerotic cardiovascular disease (ASCVD)	429.2, 440.9	I25.10
Cardiovascular degeneration (with mention of arteriosclerosis)	429.2	I25.10
Cardiovascular disease (with mention of arteriosclerosis)	429.2	I25.10
Cardiovascular sclerosis (with mention of arteriosclerosis)	429.2, 440.9	I25.10
CAD (coronary artery disease)	414	I25.10
Coronary atherosclerosis	414	I25.10

© 2021 Eberly LA et al. *JAMA Network Open*.

Arteriosclerotic coronary artery disease	414	I25.10
Arteriosclerotic heart disease	414	I25.10
Disease of the arteries of the heart	414	I25.10
Clogged artery (heart)	414	I25.10
Hardening of the arteries of the heart	414	I25.10
CAD (coronary artery disease), native coronary artery	414.01	I25.10
CHD (coronary heart disease)	414	I25.10
Coronary artery stricture	414	I25.10
Arteriosclerotic heart disease (ASHD)	414	I25.10
Coronary artery arteriosclerosis	414	I25.10
Coronary artery sclerosis	414	I25.10
Chronic coronary artery disease	414	I25.10
Single vessel coronary artery disease	414	I25.10
Coronary artery stenosis	414	I25.10
Plaque in heart artery	414	I25.10
Triple vessel coronary artery disease	414	I25.10
Triple vessel disease of the heart	414	I25.10
Non-occlusive coronary artery disease	414	I25.10
Coronary artery disease, non-occlusive	414	I25.10
Occlusive coronary artery disease	414	I25.10
Coronary artery disease, occlusive	414	I25.10
Non-occlusive coronary artery disease requiring drug therapy	414	I25.10
Occlusive coronary artery disease requiring drug therapy	414	I25.10
Arteriosclerosis of coronary artery	414	I25.10
3-vessel coronary artery disease	414	I25.10
Single vessel coronary disease	414	I25.10
Double vessel coronary artery disease	414	I25.10
Two-vessel coronary artery disease	414	I25.10
2-vessel coronary artery disease	414	I25.10
3-vessel CAD	414	I25.10
Coronary arteriosclerosis in native artery	414.01	I25.10
CAD in native artery	414.01	I25.10
Multiple vessel coronary artery disease	414	I25.10
CAD, multiple vessel	414	I25.10

© 2021 Eberly LA et al. *JAMA Network Open*.

Radiation-induced coronary artery disease	414.00, E926.9	I25.10
Stenosis of left anterior descending artery	414	I25.10
LAD stenosis	414	I25.10
Coronary ostial sclerosis	414	I25.10
Coronary ostial stenosis	414	I25.10
Coronary artery calcification seen on CAT scan	414	I25.10
Nonocclusive coronary atherosclerosis of native coronary artery	414.01	I25.10
Atherosclerotic heart disease of native coronary artery without angina pectoris	414.01	I25.10
Preclinical coronary artery disease	414	I25.10
Pre-clinical coronary artery disease	414	I25.10
Obliterative coronary artery disease	414	I25.10
Left main coronary artery disease	414	I25.10
Atherosclerosis of coronary artery	414	I25.10
Atherosclerosis of coronary artery without graft	414	I25.10
Calcific coronary arteriosclerosis	414	I25.10
Atherosclerotic cardiovascular disease	429.2	I25.10
Atherosclerosis of native coronary artery without angina pectoris	414.01	I25.10
Coronary artery calcification seen on computed tomography	414	I25.10
Coronary artery calcification seen on CT scan	414	I25.10
Coronary artery disease involving left main coronary artery	414	I25.10
Atherosclerosis of coronary artery without history of bypass graft	414	I25.10
Stricture of coronary artery	414	I25.10
Atherosclerosis of coronary artery without angina pectoris	414	I25.10
Coronary artery disease involving native coronary artery	414.01	I25.10

© 2021 Eberly LA et al. *JAMA Network Open*.

Coronary artery disease without angina pectoris	414	I25.10
Coronary artery disease involving native coronary artery without angina pectoris	414.01	I25.10
Hereditary cardiovascular system disorder	429.2	I25.10
Mild CAD	414	I25.10
Mild coronary artery disease	414	I25.10
Multi-vessel coronary artery stenosis	414.01	I25.10
Stenosis of native coronary artery	414.01	I25.10
Asymptomatic arteriosclerosis of coronary artery	414.01	I25.10
Stenosis of left anterior descending (LAD) artery	414	I25.10
Atherosclerosis of native coronary artery	414.01	I25.10
Atherosclerosis of native coronary artery of native heart	414.01	I25.10
Atherosclerosis of native coronary artery of native heart without angina pectoris	414.01	I25.10
Atherosclerosis of coronary artery of native heart	414.01	I25.10
Coronary artery disease involving native coronary artery of native heart	414.01	I25.10
Coronary artery disease involving native coronary artery of native heart without angina pectoris	414.01	I25.10
Atherosclerosis of coronary artery of native heart without angina pectoris	414.01	I25.10
Coronary artery disease involving native heart without angina pectoris	414.01	I25.10
Coronary artery disease involving native heart	414.01	I25.10
Atherosclerosis of native coronary artery without angina pectoris, unspecified whether native or transplanted heart	414.01	I25.10
Cor athrscl-uns vessel	414	I25.10

© 2021 Eberly LA et al. *JAMA Network Open*.

Coronary artery disease involving native coronary artery without angina pectoris, unspecified whether native or transplanted heart	414.01	I25.10
Atherosclerosis of coronary artery, angina presence unspecified, unspecified vessel or lesion type, unspecified whether native or transplanted heart	414	I25.10
Coronary artery disease involving native coronary artery, angina presence unspecified, unspecified whether native or transplanted heart	414.01	I25.10
Coronary artery disease without angina pectoris, unspecified vessel or lesion type, unspecified whether native or transplanted heart	414	I25.10
Coronary artery disease, angina presence unspecified, unspecified vessel or lesion type, unspecified whether native or transplanted heart	414	I25.10
Atherosclerosis of coronary artery without angina pectoris, unspecified vessel or lesion type, unspecified whether native or transplanted heart	414	I25.10
Atherosclerosis of native coronary artery of native heart, angina presence unspecified	414.01	I25.10
Coronary artery disease involving native heart, angina presence unspecified, unspecified vessel or lesion type	414.01	I25.10
Atherosclerosis of coronary artery of native heart, angina presence unspecified, unspecified vessel or lesion type	414.01	I25.10
Atherosclerosis of native coronary artery, angina presence unspecified, unspecified whether native or transplanted heart	414.01	I25.10

© 2021 Eberly LA et al. *JAMA Network Open*.

Coronary artery disease involving native coronary artery of native heart, angina presence unspecified	414.01	I25.10
Atherosclerosis of coronary artery of native heart without angina pectoris, unspecified vessel or lesion type	414.01	I25.10
Coronary artery disease involving native heart without angina pectoris, unspecified vessel or lesion type	414.01	I25.10
Athscl heart disease of native coronary artery w/o ang pctrs	414.01	I25.10
Atherosclerotic heart disease of native coronary artery without angina pectoris		I25.10
Nonobstructive atherosclerosis of coronary artery	414	I25.10
Coronary artery disease with hx of myocardial infarct w/o hx of CABG	414.01, 412	I25.10, I25.2
Coronary artery disease with history of myocardial infarction without history of CABG	414.01, 412	I25.10, I25.2
Arteriosclerosis of coronary artery in patient with history of myocardial infarction	414.00, 412	I25.10, I25.2
Coronary arteriosclerosis in patient with history of previous myocardial infarction	414.00, 412	I25.10, I25.2
Chronic total occlusion of native coronary artery	414.01, 414.2	I25.10, I25.82
Coronary atherosclerosis due to lipid rich plaque	414.3	I25.10, I25.83
Coronary arteriosclerosis due to lipid rich plaque	414.3	I25.10, I25.83
Coronary artery disease due to lipid rich plaque	414.00, 414.3	I25.10, I25.83
Coronary artery calcinosis	414.00, 414.4	I25.10, I25.84
Calcification of native coronary artery	414.01, 414.4	I25.10, I25.84
Coronary atherosclerosis due to calcified coronary lesion	414.00, 414.4	I25.10, I25.84

© 2021 Eberly LA et al. *JAMA Network Open*.

Coronary atherosclerosis due to calcified coronary lesion of native artery	414.00, 414.4	I25.10, I25.84
Coronary artery calcification	414.00, 414.4	I25.10, I25.84
Coronary artery calcification of native artery	414.01, 414.4	I25.10, I25.84
Calcification of coronary artery	414.00, 414.4	I25.10, I25.84
Coronary artery disease due to calcified coronary lesion	414.00, 414.4	I25.10, I25.84
Atherosclerotic cardiovascular disease (ASCVD) involving retina	440.8, 362.13	I25.10, I70.8, H35.019
Presence of stent in coronary artery in patient with coronary artery disease	414.01, V45.82	I25.10, Z95.5
Recurrent coronary arteriosclerosis following PTCA	414.01, V45.82	I25.10, Z98.61
CAD S/P percutaneous coronary angioplasty	414.01, V45.82	I25.10, Z98.61
Recurrent coronary arteriosclerosis after percutaneous transluminal coronary angioplasty	414.01, V45.82	I25.10, Z98.61
Coronary arteriosclerosis after percutaneous transluminal coronary angioplasty (PTCA)	414.00, V45.89	I25.10, Z98.61
Atherosclerotic heart disease of native coronary artery with angina pectoris		I25.11
Atherosclerotic heart disease of native coronary artery with unstable angina pectoris	414.01, 411.1	I25.110
Atherosclerosis of native coronary artery with unstable angina pectoris	414.01, 411.1	I25.110
Atherosclerosis of coronary artery with unstable angina pectoris	414.00, 411.1	I25.110
Coronary artery disease involving native coronary artery with unstable angina pectoris	414.01, 411.1	I25.110
Coronary artery disease with unstable angina pectoris	414.00, 411.1	I25.110

© 2021 Eberly LA et al. *JAMA Network Open*.

Atherosclerosis of native coronary artery of native heart with unstable angina pectoris	414.01, 411.1	I25.110
Coronary artery disease involving native coronary artery of native heart with unstable angina pectoris	414.01, 411.1	I25.110
Atherosclerosis of coronary artery of native heart with unstable angina pectoris	414.01, 411.1	I25.110
Coronary artery disease involving native heart with unstable angina pectoris	414.01, 413.9	I25.110
Atherosclerosis of native coronary artery with unstable angina pectoris, unspecified whether native or transplanted heart	414.01, 411.1	I25.110
Coronary artery disease involving native coronary artery with unstable angina pectoris, unspecified whether native or transplanted heart	414.01, 411.1	I25.110
Atherosclerosis of coronary artery of native heart with unstable angina pectoris, unspecified vessel or lesion type	414.01, 411.1	I25.110
Coronary artery disease with unstable angina pectoris, unspecified vessel or lesion type, unspecified whether native or transplanted heart	414.00, 411.1	I25.110
Atherosclerosis of coronary artery with unstable angina pectoris, unspecified vessel or lesion type, unspecified whether native or transplanted heart	414.00, 411.1	I25.110
Coronary artery disease involving native heart with unstable angina pectoris, unspecified vessel or lesion type	414.01, 413.9	I25.110
Unstable angina pectoris due to coronary arteriosclerosis	411.1, 414.01	I25.110
Athscl heart disease of native cor art w unstable ang pctrs	414.01, 411.1	I25.110

© 2021 Eberly LA et al. *JAMA Network Open*.

Atherosclerotic heart disease of native coronary artery with unstable angina pectoris		I25.110
Atherosclerotic heart disease of native coronary artery with angina pectoris with documented spasm	414.01, 413.9	I25.111
Atherosclerosis of native coronary artery with angina pectoris with documented spasm	414.01, 413.9	I25.111
Atherosclerosis of coronary artery with angina pectoris and documented spasm	414.00, 413.9	I25.111
Coronary artery disease with angina pectoris with documented spasm	414.00, 413.9	I25.111
Coronary artery disease involving native coronary artery with angina pectoris with documented spasm	414.01, 413.9	I25.111
Atherosclerosis of native coronary artery of native heart with angina pectoris with documented spasm	414.01, 413.9	I25.111
Coronary artery disease involving native coronary artery of native heart with angina pectoris with documented spasm	414.01, 413.9	I25.111
Coronary artery disease involving native heart with angina pectoris and documented spasm	414.01, 413.9	I25.111
Atherosclerosis of coronary artery of native heart with angina pectoris and documented spasm	414.01, 413.9	I25.111
Atherosclerosis of native coronary artery with angina pectoris with documented spasm, unspecified whether native or transplanted heart	414.01, 413.9	I25.111
Coronary artery disease involving native coronary artery with angina pectoris with documented spasm, unspecified whether native or transplanted heart	414.01, 413.9	I25.111

© 2021 Eberly LA et al. *JAMA Network Open*.

Coronary artery disease involving native heart with angina pectoris and documented spasm, unspecified vessel or lesion type	414.01, 413.9	I25.111
Atherosclerosis of coronary artery of native heart with angina pectoris and documented spasm, unspecified vessel or lesion type	414.01, 413.9	I25.111
Atherosclerosis of coronary artery with angina pectoris and documented spasm, unspecified vessel or lesion type, unspecified whether native or transplanted heart	414.00, 413.9	I25.111
Coronary artery disease with angina pectoris with documented spasm, unspecified vessel or lesion type, unspecified whether native or transplanted heart	414.00, 413.9	I25.111
Athscl heart disease of native cor art w ang pctrs w spasm	414.01, 413.9	I25.111
Atherosclerotic heart disease of native coronary artery with angina pectoris with documented spasm		I25.111
Athscl heart disease of native cor art w oth ang pctrs	414.01, 413.9	I25.118
Coronary artery disease with other forms of angina pectoris	414.00, 413.9	I25.118
Coronary artery disease involving native coronary artery with other forms of angina pectoris	414.01, 413.9	I25.118
Atherosclerosis of native coronary artery with other form of angina pectoris	414.01, 413.9	I25.118
Atherosclerosis of native coronary artery of native heart with other form of angina pectoris	414.01, 413.9	I25.118
Coronary artery disease involving native coronary artery of native heart with other form of angina pectoris	414.01, 413.9	I25.118

© 2021 Eberly LA et al. *JAMA Network Open*.

Atherosclerosis of coronary artery of native heart with other form of angina pectoris	414.01, 413.9	I25.118
Coronary artery disease involving native heart with other form of angina pectoris	414.01, 413.9	I25.118
Atherosclerosis of coronary artery with other form of angina pectoris	414.00, 413.9	I25.118
Atherosclerosis of native coronary artery with other form of angina pectoris, unspecified whether native or transplanted heart	414.01, 413.9	I25.118
Coronary artery disease with stable angina pectoris	414.00, 413.9	I25.118
Atherosclerosis of coronary artery of native heart with stable angina pectoris	414.01, 413.9	I25.118
Atherosclerosis of coronary artery with stable angina pectoris	414.00, 413.9	I25.118
Coronary artery disease of native artery with stable angina pectoris	414.01, 413.9	I25.118
Coronary artery disease of native heart with stable angina pectoris	414.01, 413.9	I25.118
Atherosclerosis of native coronary artery with stable angina pectoris	414.01, 413.9	I25.118
Coronary artery disease of native artery of native heart with stable angina pectoris	414.01, 413.9	I25.118
Atherosclerosis of native coronary artery of native heart with stable angina pectoris	414.01, 413.9	I25.118
Atherosclerotic heart disease of native coronary artery with other forms of angina pectoris	414.01, 413.9	I25.118
Coronary artery disease with other form of angina pectoris	414.00, 413.9	I25.118
Atherosclerosis of coronary artery of native heart with stable angina pectoris, unspecified vessel or lesion type	414.01, 413.9	I25.118

© 2021 Eberly LA et al. *JAMA Network Open*.

Coronary artery disease with stable angina pectoris, unspecified vessel or lesion type, unspecified whether native or transplanted heart	414.00, 413.9	I25.118
Coronary artery disease of native heart with stable angina pectoris, unspecified vessel or lesion type	414.01, 413.9	I25.118
Coronary artery disease of native artery with stable angina pectoris, unspecified whether native or transplanted heart	414.01, 413.9	I25.118
Atherosclerosis of native coronary artery with stable angina pectoris, unspecified whether native or transplanted heart	414.01, 413.9	I25.118
Atherosclerosis of coronary artery with stable angina pectoris, unspecified vessel or lesion type, unspecified whether native or transplanted heart	414.00, 413.9	I25.118
Coronary artery disease involving native coronary artery with other form of angina pectoris, unspecified whether native or transplanted heart	414.01, 413.9	I25.118
Atherosclerosis of coronary artery of native heart with other form of angina pectoris, unspecified vessel or lesion type	414.01, 413.9	I25.118
Atherosclerosis of coronary artery with other form of angina pectoris, unspecified vessel or lesion type, unspecified whether native or transplanted heart	414.00, 413.9	I25.118
Coronary artery disease with other form of angina pectoris, unspecified vessel or lesion type, unspecified whether native or transplanted heart	414.00, 413.9	I25.118
Coronary artery disease involving native heart with other form of angina pectoris, unspecified vessel or lesion type	414.01, 413.9	I25.118
Coronary artery disease with exertional angina	414.00, 413.9	I25.118

© 2021 Eberly LA et al. *JAMA Network Open*.

Coronary artery disease involving native coronary artery with other forms of angina pectoris, unspecified whether native or transplanted heart	414.01, 413.9	I25.118
Atherosclerotic heart disease of native coronary artery with other forms of angina pectoris		I25.118
Atherosclerotic heart disease native coronary artery w/angina pectoris	414.01, 413.9	I25.119
Atherosclerotic heart disease of native coronary artery with angina pectoris	414.01, 413.9	I25.119
Atherosclerosis of native coronary artery with angina pectoris	414.01, 413.9	I25.119
Coronary artery disease involving native coronary artery with angina pectoris	414.01, 413.9	I25.119
Atherosclerosis of native coronary artery of native heart with angina pectoris	414.01, 413.9	I25.119
Coronary artery disease involving native coronary artery of native heart with angina pectoris	414.01, 413.9	I25.119
Atherosclerosis of coronary artery of native heart with angina pectoris	414.01, 413.9	I25.119
Atherosclerosis of coronary artery with angina pectoris	414.00, 413.9	I25.119
Coronary artery disease involving native heart with angina pectoris	414.01, 413.9	I25.119
Coronary artery disease with unspecified angina pectoris	414.00, 413.9	I25.119
Atherosclerosis of native coronary artery with angina pectoris, unspecified whether native or transplanted heart	414.01, 413.9	I25.119
Coronary artery disease with angina pectoris	414.00, 413.9	I25.119
Atherosclerotic heart disease of native coronary artery with unspecified angina pectoris	414.01, 413.9	I25.119

© 2021 Eberly LA et al. *JAMA Network Open*.

Coronary artery disease involving native coronary artery with angina pectoris, unspecified whether native or transplanted heart	414.01, 413.9	I25.119
Coronary artery disease with angina pectoris, unspecified vessel or lesion type, unspecified whether native or transplanted heart	414.00, 413.9	I25.119
Coronary artery disease involving native heart with angina pectoris, unspecified vessel or lesion type	414.01, 413.9	I25.119
Atherosclerosis of coronary artery with angina pectoris, unspecified vessel or lesion type, unspecified whether native or transplanted heart	414.00, 413.9	I25.119
Atherosclerosis of coronary artery of native heart with angina pectoris, unspecified vessel or lesion type	414.01, 413.9	I25.119
Angina concurrent with and due to arteriosclerosis of coronary artery	413.9, 414.01	I25.119
Athscl heart disease of native cor art w unsp ang pctrs	414.01, 413.9	I25.119
Atherosclerotic heart disease of native coronary artery with unspecified angina pectoris		I25.119
Old myocardial infarction	412	I25.2
Scarring of papillary muscle	429.81	I25.2
Healed myocardial infarction	412	I25.2
Papillary muscle scarring	429.81	I25.2
Healed myocardial infarct	412	I25.2
Past heart attack	412	I25.2
Past myocardial infarction	412	I25.2
Old myocardial infarct	412	I25.2
ECG: old myocardial infarction	412	I25.2
Healed coronary	412	I25.2
Scar, papillary muscle	429.81	I25.2
Scarring, papillary muscle	429.81	I25.2

© 2021 Eberly LA et al. *JAMA Network Open*.

Old MI (myocardial infarction)	412	I25.2
Personal history of MI (myocardial infarction)	412	I25.2
Myocardial infarction with symptoms after 8 weeks from infarction date	414.8	I25.2
Past myocardial infarction ECG/special investigatn diagnos, no symptom	412	I25.2
Asymptomatic old MI (myocardial infarction)	412	I25.2
Myocardial infarct, old	412	I25.2
Past history of myocardial infarction	412	I25.2
History of heart attack	412	I25.2
History of MI (myocardial infarction)	412	I25.2
Myocardial infarction, old	412	I25.2
Apical myocardial infarction greater than eight weeks ago	412	I25.2
History of myocardial infarction, greater than 8 weeks	412	I25.2
Myocardial infarction of anterior wall greater than eight weeks ago	412	I25.2
Anterior myocardial infarction greater than eight weeks ago	412	I25.2
Anterolateral myocardial infarction greater than eight weeks ago	412	I25.2
Old myocardial infarction of inferior wall, greater than 8 weeks	412	I25.2
Previous inferior myocardial infarction older than 8 weeks	412	I25.2
Old myocardial infarction, greater than 8 weeks	412	I25.2
Previous myocardial infarction older than 8 weeks	412	I25.2
Old non-Q wave myocardial infarction	412	I25.2
Old non-ST elevation myocardial infarction (NSTEMI)	412	I25.2
Asymptomatic old myocardial infarction	412	I25.2
History of myocardial infarction	412	I25.2

© 2021 Eberly LA et al. *JAMA Network Open*.

History of acute myocardial infarction of septum	412	I25.2
History of acute myocardial infarction of anterior wall	412	I25.2
History of acute myocardial infarction of anterolateral wall	412	I25.2
History of acute myocardial infarction of lateral wall	412	I25.2
History of acute myocardial infarction	412	I25.2
History of acute myocardial infarction of inferior wall	412	I25.2
History of acute anterior wall myocardial infarction	412	I25.2
History of acute inferior wall myocardial infarction	412	I25.2
History of acute lateral wall myocardial infarction	412	I25.2
History of acute anterior wall MI	412	I25.2
History of acute anterolateral myocardial infarction	412	I25.2
History of acute anterolateral wall MI	412	I25.2
History of acute inferior wall MI	412	I25.2
History of acute lateral wall MI	412	I25.2
MI, old	412	I25.2
History of ST elevation myocardial infarction	412	I25.2
History of non-ST elevation myocardial infarction (NSTEMI)	412	I25.2
History of ST elevation myocardial infarction (STEMI)	412	I25.2
Hx of myocardial infarction	412	I25.2
H/O ST elevation myocardial infarction	412	I25.2
Hx of ST elevation myocardial infarction	412	I25.2
Hx of non-ST elevation myocardial infarction (NSTEMI)	412	I25.2
H/O non-ST elevation myocardial infarction (NSTEMI)	412	I25.2

© 2021 Eberly LA et al. *JAMA Network Open*.

Hx of myocardial infarction of inferior wall, greater than 8 weeks	412	I25.2
H/O myocardial infarction of inferior wall, greater than 8 weeks	412	I25.2
H/O myocardial infarction, greater than 8 weeks	412	I25.2
Hx of myocardial infarction, greater than 8 weeks	412	I25.2
Hx of acute myocardial infarction of anterolateral wall	412	I25.2
H/O acute myocardial infarction of anterolateral wall	412	I25.2
Hx of acute myocardial infarction of anterior wall	412	I25.2
H/O acute myocardial infarction of anterior wall	412	I25.2
Hx of acute myocardial infarction of inferior wall	412	I25.2
H/O acute myocardial infarction of inferior wall	412	I25.2
H/O acute myocardial infarction	412	I25.2
Hx of acute myocardial infarction	412	I25.2
H/O acute myocardial infarction of lateral wall	412	I25.2
Hx of acute myocardial infarction of lateral wall	412	I25.2
H/O acute myocardial infarction of septum	412	I25.2
Hx of acute myocardial infarction of septum	412	I25.2
Old inferior wall myocardial infarction	412	I25.2
Old anterior myocardial infarction	412	I25.2
Old inferior myocardial infarction	412	I25.2
Old anteroseptal myocardial infarction	412	I25.2
Old anterolateral wall myocardial infarction	412	I25.2
Old anterior wall myocardial infarction	412	I25.2

© 2021 Eberly LA et al. *JAMA Network Open*.

Old inferolateral myocardial infarction	412	I25.2
Old inferoposterior myocardial infarction	412	I25.2
Old lateral wall myocardial infarction	412	I25.2
Old true posterior myocardial infarction	412	I25.2
Anteroapical myocardial infarction greater than eight weeks ago	412	I25.2
Anteroseptal myocardial infarction greater than eight weeks ago	412	I25.2
Myocardial infarction of lateral wall greater than eight weeks ago	412	I25.2
Non-Q wave myocardial infarction greater than eight weeks ago	412	I25.2
Myocardial infarction with symptoms after 8 weeks from date of infarction	414.8	I25.2
History of myocardial infarction of inferior wall, greater than 8 weeks	412	I25.2
Myocardial infarction of anterolateral wall greater than eight weeks ago	412	I25.2
Myocardial infarction of inferolateral wall greater than eight weeks ago	412	I25.2
Non-ST elevation myocardial infarction (NSTEMI) greater than eight weeks ago	412	I25.2
Old posterior myocardial infarction	412	I25.2
Old lateral myocardial infarction	412	I25.2
History of myocardial infarction in last year	412	I25.2
History of myocardial infarct at age greater than 60 years	412	I25.2
History of myocardial infarct at age less than 60 years	412	I25.2
Old subendocardial infarction	412	I25.2
History of anterior wall myocardial infarction	412	I25.2
History of anterolateral myocardial infarction	412	I25.2
History of inferior wall myocardial infarction	412	I25.2

© 2021 Eberly LA et al. *JAMA Network Open*.

History of lateral wall myocardial infarction	412	I25.2
History of myocardial infarction of septum	412	I25.2
Old anterolateral myocardial infarction	412	I25.2
Status post myocardial infarction	412	I25.2
Acute myocardial infarct greater than 3 months ago	412	I25.2
History of anteroapical myocardial infarction	412	I25.2
Status post myocardial infarction of anterolateral wall	412	I25.2
Status post non-Q wave myocardial infarction	412	I25.2
Status post myocardial infarction of anterior wall	412	I25.2
Status post anteroapical myocardial infarction	412	I25.2
History of myocardial infarction of inferoposterior wall	412	I25.2
Status post myocardial infarction of inferior wall	412	I25.2
Status post myocardial infarction of lateral wall	412	I25.2
Status post non-ST elevation myocardial infarction (NSTEMI)	412	I25.2
Evidence of prior myocardial infarction on electrocardiogram	412	I25.2
Myocardial infarction, greater than 8 weeks old	412	I25.2
History of myocardial infarction, greater than 8 weeks ago	412	I25.2
History of myocardial infarction in adulthood	412	I25.2
Myocardial infarction of inferior wall greater than 8 weeks ago	412	I25.2
Myocardial infarction of anterior wall greater than 8 weeks ago	412	I25.2

© 2021 Eberly LA et al. *JAMA Network Open*.

Myocardial infarction of inferolateral wall greater than 8 weeks ago	412	I25.2
Anteroseptal myocardial infarction greater than 8 weeks ago	412	I25.2
Non-Q wave myocardial infarction greater than 8 weeks ago	412	I25.2
Anteroapical myocardial infarction greater than 8 weeks ago	412	I25.2
Non-ST elevation myocardial infarction (NSTEMI) greater than 8 weeks ago	412	I25.2
Apical myocardial infarction greater than 8 weeks ago	412	I25.2
Myocardial infarction greater than 8 weeks ago	412	I25.2
Myocardial infarction of anterolateral wall greater than 8 weeks ago	412	I25.2
Myocardial infarction of lateral wall greater than 8 weeks ago	412	I25.2
Evidence of prior myocardial infarction on electrocardiography	412	I25.2
Myocardial infarction of anterolateral wall greater than 4 weeks ago	IMO0002	I25.2
Myocardial infarction of inferolateral wall greater than 4 weeks ago	IMO0002	I25.2
Myocardial infarction of anterior wall greater than 4 weeks ago	IMO0002	I25.2
Old myocardial infarction		I25.2
Aneurysm of heart (wall)	414.1	I25.3
Other aneurysm of heart	414.19	I25.3
Aneurysm of heart wall	414.1	I25.3
Mural aneurysm of heart	414.1	I25.3
Ventricular aneurysm	414.1	I25.3
Atrial aneurysm	414.1	I25.3
Cardiac aneurysm	414.1	I25.3
Heart aneurysm	414.1	I25.3
Mural aneurysm	414.1	I25.3
Aneurysm of heart NEC	414.19	I25.3

© 2021 Eberly LA et al. *JAMA Network Open*.

Aneurysm of heart	414.1	I25.3
Aneurysm, mural heart	414.1	I25.3
Coronary artery fistula	414.19	I25.3
Aneurysm and dissection of heart	414.1	I25.3
Aneurysm of right ventricle of heart	414.1	I25.3
Aneurysm of left ventricle of heart	414.1	I25.3
Pseudoaneurysm of left ventricle of heart	414.1	I25.3
Pseudoaneurysm of right ventricle of heart	414.1	I25.3
Atrial septal aneurysm	414.1	I25.3
Aneurysm, other cardiac	414.19	I25.3
Left ventricular aneurysm	414.1	I25.3
Right ventricular aneurysm	414.1	I25.3
Cardiac pseudoaneurysm	414.1	I25.3
Left ventricular pseudoaneurysm	414.1	I25.3
Aneurysm, heart wall	414.1	I25.3
Fistula, coronary artery	414.19	I25.3
Fossa ovalis aneurysm	414.1	I25.3
RV aneurysm	414.1	I25.3
Ischemic cardiomyopathy	414.8	I25.5
Cardiomyopathy, ischemic	414.8	I25.5
Generalized ischemic myocardial dysfunction	414.8	I25.5
Ischemic myocardial dysfunction	414.9	I25.5
Ischemic cardiomyopathy		I25.5
Ischemic dilated cardiomyopathy	414.8	I25.5, I42.0
Silent myocardial ischemia	414.8	I25.6
Asymptomatic myocardial ischemia	414.8	I25.6
Silent myocardial ischemia		I25.6
Atherosclerosis of coronary artery bypass graft(s) and coronary artery of transplanted heart with angina pectoris		I25.7
Atherosclerosis of coronary artery bypass graft(s), unspecified, with angina pectoris		I25.70
Atheroscler of coronary artery bypass graft w/unstable angina pectoris	414.05, 411.1	I25.700

© 2021 Eberly LA et al. *JAMA Network Open*.

Atherosclerosis of coronary artery bypass graft with unstable angina pectoris	414.05, 411.1	I25.700
Atherosclerosis of CABG w unstable angina pectoris	414.05, 411.1	I25.700
Coronary artery disease involving coronary bypass graft with unstable angina pectoris	414.05, 411.1	I25.700
Coronary artery disease involving coronary bypass graft of native heart with unstable angina pectoris	414.05, 411.1	I25.700
Atherosclerosis of coronary artery bypass graft of native heart with unstable angina pectoris	414.05, 411.1	I25.700
Atherosclerosis of coronary artery bypass graft with unstable angina pectoris, unspecified whether native or transplanted heart	414.05, 411.1	I25.700
Atherosclerosis of coronary artery bypass graft(s), unspecified, with unstable angina pectoris	414.05, 411.1	I25.700
Coronary artery disease involving coronary bypass graft with unstable angina pectoris, unspecified whether native or transplanted heart	414.05, 411.1	I25.700
Atherosclerosis of CABG, unsp, w unstable angina pectoris	414.05, 411.1	I25.700
Atherosclerosis of coronary artery bypass graft(s), unspecified, with unstable angina pectoris		I25.700
Atherosclerosis of coronary artery bypass graft with angina pectoris with documented spasm	414.05, 413.9	I25.701
Atherosclerosis of CABG w angina pectoris w documented spasm	414.05, 413.9	I25.701
Coronary artery disease involving coronary bypass graft with angina pectoris with documented spasm	414.05, 413.9	I25.701

© 2021 Eberly LA et al. *JAMA Network Open*.

Coronary artery disease involving coronary bypass graft of native heart with angina pectoris with documented spasm	414.05, 413.9	I25.701
Atherosclerosis of coronary artery bypass graft of native heart with angina pectoris with documented spasm	414.05, 413.9	I25.701
Atherosclerosis of coronary artery bypass graft with angina pectoris with documented spasm, unspecified whether native or transplanted heart	414.05, 413.9	I25.701
Atherosclerosis of coronary artery bypass graft(s), unspecified, with angina pectoris with documented spasm	414.05, 413.9	I25.701
Coronary artery disease involving coronary bypass graft with angina pectoris with documented spasm, unspecified whether native or transplanted heart	414.05, 413.9	I25.701
Athscl CABG, unsp, w angina pectoris w documented spasm	414.05, 413.9	I25.701
Atherosclerosis of coronary artery bypass graft(s), unspecified, with angina pectoris with documented spasm		I25.701
Atherosclerosis of CABG, unsp, w oth angina pectoris	414.05, 413.9	I25.708
Atherosclerosis of CABG w oth angina pectoris	414.05, 413.9	I25.708
Atherosclerosis of coronary artery bypass graft with other forms of angina pectoris	414.05, 413.9	I25.708
Coronary artery disease involving coronary bypass graft with other forms of angina pectoris	414.05, 413.9	I25.708
Coronary artery disease involving coronary bypass graft of native heart with other forms of angina pectoris	414.05, 413.9	I25.708
Atherosclerosis of coronary artery bypass graft of native heart with other forms of angina pectoris	414.05, 413.9	I25.708

© 2021 Eberly LA et al. *JAMA Network Open*.

Atherosclerosis of coronary artery bypass graft with other forms of angina pectoris, unspecified whether native or transplanted heart	414.05, 413.9	I25.708
Coronary artery disease of bypass graft with stable angina pectoris	414.05, 413.9	I25.708
Atherosclerosis of coronary artery bypass graft of native heart with stable angina pectoris	414.04, 413.9	I25.708
Atherosclerosis of coronary artery bypass graft with stable angina pectoris	414.04, 413.9	I25.708
Coronary artery disease of bypass graft of native heart with stable angina pectoris	414.05, 413.9	I25.708
Atherosclerosis of coronary artery bypass graft(s), unspecified, with other forms of angina pectoris	414.05, 413.9	I25.708
Atherosclerosis of coronary artery bypass graft with stable angina pectoris, unspecified whether native or transplanted heart	414.04, 413.9	I25.708
Coronary artery disease of bypass graft with stable angina pectoris, unspecified whether native or transplanted heart	414.05, 413.9	I25.708
Coronary artery disease involving coronary bypass graft with other forms of angina pectoris, unspecified whether native or transplanted heart	414.05, 413.9	I25.708
Atherosclerosis of coronary artery bypass graft(s), unspecified, with other forms of angina pectoris		I25.708
Atherosclerosis of coronary artery bypass graft with angina pectoris	414.05, 413.9	I25.709
Atherosclerosis of CABG w unsp angina pectoris	414.05, 413.9	I25.709
Atherosclerosis of coronary artery bypass graft with angina pectoris, unspecified whether native or transplanted heart	414.05, 413.9	I25.709

© 2021 Eberly LA et al. *JAMA Network Open*.

Coronary artery disease involving coronary bypass graft with unspecified angina pectoris	414.05, 413.9	I25.709
Coronary artery disease involving coronary bypass graft of native heart with unspecified angina pectoris	414.05, 413.9	I25.709
Atherosclerosis of coronary artery bypass graft of native heart with unspecified angina pectoris	414.05, 413.9	I25.709
Coronary artery disease involving coronary bypass graft with angina pectoris	414.05, 413.9	I25.709
Coronary artery disease involving coronary bypass graft of native heart with angina pectoris	414.05, 413.9	I25.709
Atherosclerosis of coronary artery bypass graft of native heart with angina pectoris	414.05, 413.9	I25.709
Atherosclerosis of coronary artery bypass graft(s), unspecified, with unspecified angina pectoris	414.05, 413.9	I25.709
Coronary artery disease involving coronary bypass graft with angina pectoris, unspecified whether native or transplanted heart	414.05, 413.9	I25.709
Angina concurrent with and due to arteriosclerosis of coronary artery bypass graft	413.9, 414.05	I25.709
Angina concurrent with and due to arteriosclerosis of CABG	413.9, 414.05	I25.709
Atherosclerosis of CABG, unsp, w unsp angina pectoris	414.05, 413.9	I25.709
Atherosclerosis of coronary artery bypass graft(s), unspecified, with unspecified angina pectoris		I25.709
Atherosclerosis of autologous vein coronary artery bypass graft(s) with angina pectoris		I25.71

© 2021 Eberly LA et al. *JAMA Network Open*.

Atherosclerosis of autologous vein coronary artery bypass graft with unstable angina pectoris	414.02, 411.1	I25.710
Coronary artery disease involving autologous vein coronary bypass graft with unstable angina pectoris	414.02, 411.1	I25.710
Atherosclerosis of autologous vein coronary artery bypass graft(s) with unstable angina pectoris	414.02, 411.1	I25.710
Athscl autologous vein CABG w unstable angina pectoris	414.02, 411.1	I25.710
Atherosclerosis of autologous vein coronary artery bypass graft(s) with unstable angina pectoris		I25.710
Atherosclerosis of autologous vein coronary artery bypass graft with angina pectoris with documented spasm	414.02, 413.9	I25.711
Coronary artery disease involving autologous vein coronary bypass graft with angina pectoris with documented spasm	414.02, 413.9	I25.711
Atherosclerosis of autologous vein coronary artery bypass graft(s) with angina pectoris with documented spasm	414.02, 413.9	I25.711
Athscl autologous vein CABG w ang pctrs w documented spasm	414.02, 413.9	I25.711
Atherosclerosis of autologous vein coronary artery bypass graft(s) with angina pectoris with documented spasm		I25.711
Athscl autologous vein CABG w oth angina pectoris	414.02, 413.9	I25.718
Atherosclerosis of autologous vein coronary artery bypass graft with other forms of angina pectoris	414.02, 413.9	I25.718
Coronary artery disease involving autologous vein coronary bypass graft with other forms of angina pectoris	414.02, 413.9	I25.718

© 2021 Eberly LA et al. *JAMA Network Open*.

Atherosclerosis of autologous vein coronary artery bypass graft with stable angina pectoris	414.02, 413.9	I25.718
Coronary artery disease of autologous vein bypass graft with stable angina pectoris	414.02, 413.9	I25.718
Atherosclerosis of autologous vein coronary artery bypass graft(s) with other forms of angina pectoris	414.02, 413.9	I25.718
Atherosclerosis of autologous vein coronary artery bypass graft(s) with other forms of angina pectoris		I25.718
Atherosclerosis of autologous vein coronary artery bypass graft with angina pectoris	414.02, 413.9	I25.719
Coronary artery disease involving autologous vein coronary bypass graft with unspecified angina pectoris	414.02, 413.9	I25.719
Coronary artery disease involving autologous vein coronary bypass graft with angina pectoris	414.02, 413.9	I25.719
Atherosclerosis of autologous vein coronary artery bypass graft(s) with unspecified angina pectoris	414.02, 413.9	I25.719
Athscl autologous vein CABG w unsp angina pectoris	414.02, 413.9	I25.719
Atherosclerosis of autologous vein coronary artery bypass graft(s) with unspecified angina pectoris		I25.719
Atherosclerosis of autologous artery coronary artery bypass graft(s) with angina pectoris		I25.72
Atherosclerosis of autologous artery coronary artery bypass graft with unstable angina pectoris	414.04, 411.1	I25.720
Coronary artery disease involving autologous artery coronary bypass graft with unstable angina pectoris	414.04, 411.1	I25.720

© 2021 Eberly LA et al. *JAMA Network Open*.

Atherosclerosis of autologous artery coronary artery bypass graft(s) with unstable angina pectoris	414.04, 411.1	I25.720
Athscl autologous artery CABG w unstable angina pectoris	414.04, 411.1	I25.720
Atherosclerosis of autologous artery coronary artery bypass graft(s) with unstable angina pectoris		I25.720
Atherosclerosis of autologous artery coronary artery bypass graft with angina pectoris with documented spasm	414.04, 413.9	I25.721
Coronary artery disease involving autologous artery coronary bypass graft with angina pectoris with documented spasm	414.04, 413.9	I25.721
Atherosclerosis of autologous artery coronary artery bypass graft(s) with angina pectoris with documented spasm	414.04, 413.9	I25.721
Athscl autologous artery CABG w ang pctrs w documented spasm	414.04, 413.9	I25.721
Atherosclerosis of autologous artery coronary artery bypass graft(s) with angina pectoris with documented spasm		I25.721
Athscl autologous artery CABG w oth angina pectoris	414.04, 413.9	I25.728
Atherosclerosis of autologous artery coronary artery bypass graft with other forms of angina pectoris	414.04, 413.9	I25.728
Coronary artery disease involving autologous artery coronary bypass graft with other forms of angina pectoris	414.04, 413.9	I25.728
Coronary artery disease of autologous bypass graft with stable angina pectoris	414.04, 413.9	I25.728
Atherosclerosis of autologous artery coronary artery bypass graft with stable angina	414.04, 413.9	I25.728

© 2021 Eberly LA et al. *JAMA Network Open*.

Atherosclerosis of autologous artery coronary artery bypass graft(s) with other forms of angina pectoris	414.04, 413.9	I25.728
Atherosclerosis of autologous artery coronary artery bypass graft(s) with other forms of angina pectoris		I25.728
Atherosclerosis of autologous artery coronary artery bypass graft with angina pectoris	414.04, 413.9	I25.729
Coronary artery disease involving autologous artery coronary bypass graft with unspecified angina pectoris	414.04, 413.9	I25.729
Coronary artery disease involving autologous artery coronary bypass graft with angina pectoris	414.04, 413.9	I25.729
Atherosclerosis of autologous artery coronary artery bypass graft(s) with unspecified angina pectoris	414.04, 413.9	I25.729
Athscl autologous artery CABG w unsp angina pectoris	414.04, 413.9	I25.729
Atherosclerosis of autologous artery coronary artery bypass graft(s) with unspecified angina pectoris		I25.729
Atherosclerosis of nonautologous biological coronary artery bypass graft(s) with angina pectoris		I25.73
Atherosclerosis of nonautologous biological coronary artery bypass graft with unstable angina pectoris	414.03, 411.1	I25.730
Coronary artery disease involving nonautologous biological coronary bypass graft with unstable angina pectoris	414.03, 411.1	I25.730
Atherosclerosis of nonautologous biological coronary artery bypass graft(s) with unstable angina pectoris	414.03, 411.1	I25.730
Athscl nonautologous biological CABG w unstable ang pctrs	414.03, 411.1	I25.730

© 2021 Eberly LA et al. *JAMA Network Open*.

Atherosclerosis of nonautologous biological coronary artery bypass graft(s) with unstable angina pectoris		I25.730
Atherosclerosis of nonautologous biological coronary artery bypass graft with angina pectoris with documented spasm	414.03, 413.9	I25.731
Coronary artery disease involving nonautologous biological coronary bypass graft with angina pectoris with documented spasm	414.03, 413.9	I25.731
Atherosclerosis of nonautologous biological coronary artery bypass graft(s) with angina pectoris with documented spasm	414.03, 413.9	I25.731
Athscl nonaut biological CABG w ang pctrs w documented spasm	414.03, 413.9	I25.731
Atherosclerosis of nonautologous biological coronary artery bypass graft(s) with angina pectoris with documented spasm		I25.731
Athscl nonautologous biological CABG w oth angina pectoris	414.03, 413.9	I25.738
Atherosclerosis of nonautologous biological coronary artery bypass graft with other forms of angina pectoris	414.03, 413.9	I25.738
Coronary artery disease involving nonautologous biological coronary bypass graft with other forms of angina pectoris	414.03, 413.9	I25.738
Atherosclerosis of non-autologous biological coronary artery bypass graft with stable angina pectoris	414.03, 413.9	I25.738
Coronary artery disease of non-autologous biological bypass graft with stable angina pectoris	414.03, 413.9	I25.738
Atherosclerosis of nonautologous biological coronary artery bypass graft(s) with other forms of angina pectoris	414.03, 413.9	I25.738

© 2021 Eberly LA et al. *JAMA Network Open*.

Atherosclerosis of other coronary artery bypass graft with stable angina pectoris	414.05, 413.9	I25.738
Atherosclerosis of nonautologous biological coronary artery bypass graft(s) with other forms of angina pectoris		I25.738
Atherosclerosis of nonautologous biological coronary artery bypass graft with angina pectoris	414.03, 413.9	I25.739
Coronary artery disease involving nonautologous biological coronary bypass graft with unspecified angina pectoris	414.03, 413.9	I25.739
Coronary artery disease involving nonautologous biological coronary bypass graft with angina pectoris	414.03, 413.9	I25.739
Atherosclerosis of nonautologous biological coronary artery bypass graft(s) with unspecified angina pectoris	414.03, 413.9	I25.739
Athscl nonautologous biological CABG w unsp angina pectoris	414.03, 413.9	I25.739
Atherosclerosis of nonautologous biological coronary artery bypass graft(s) with unspecified angina pectoris		I25.739
Atherosclerosis of native coronary artery of transplanted heart with angina pectoris		I25.75
Atherosclerosis of native coronary artery of transplanted heart with unstable angina	414.06, 411.1	I25.750
Coronary artery disease involving native artery of transplanted heart with unstable angina pectoris	414.06, 411.1	I25.750
Coronary artery disease involving transplanted heart with unstable angina pectoris	414.06, 413.9	I25.750
Atherosclerosis of coronary artery of transplanted heart with unstable angina pectoris	414.06, 413.9	I25.750

© 2021 Eberly LA et al. *JAMA Network Open*.

Coronary artery disease involving transplanted heart with unstable angina pectoris, unspecified vessel or lesion type	414.06, 413.9	I25.750
Atherosclerosis of coronary artery of transplanted heart with unstable angina pectoris, unspecified vessel or lesion type	414.06, 413.9	I25.750
Athscl native cor art of txplt heart w unstable angina	414.06, 411.1	I25.750
Atherosclerosis of native coronary artery of transplanted heart with unstable angina		I25.750
Atherosclerosis of native coronary artery of transplanted heart with angina pectoris with documented spasm	414.06, 413.9	I25.751
Coronary artery disease involving native artery of transplanted heart with angina pectoris with documented spasm	414.06, 413.9	I25.751
Coronary artery disease involving transplanted heart with angina pectoris and documented spasm	414.06, 413.9	I25.751
Atherosclerosis of coronary artery of transplanted heart with angina pectoris and documented spasm	414.06, 413.9	I25.751
Coronary artery disease involving transplanted heart with angina pectoris and documented spasm, unspecified vessel or lesion type	414.06, 413.9	I25.751
Atherosclerosis of coronary artery of transplanted heart with angina pectoris and documented spasm, unspecified vessel or lesion type	414.06, 413.9	I25.751
Athscl native cor art of txplt heart w ang pctrs w spasm	414.06, 413.9	I25.751
Atherosclerosis of native coronary artery of transplanted heart with angina pectoris with documented spasm		I25.751
Athscl native cor art of transplanted heart w oth ang pctrs	414.06, 413.9	I25.758

© 2021 Eberly LA et al. *JAMA Network Open*.

Atherosclerosis of native coronary artery of transplanted heart with other forms of angina pectoris	414.06, 413.9	I25.758
Coronary artery disease involving native artery of transplanted heart with other forms of angina pectoris	414.06, 413.9	I25.758
Atherosclerosis of coronary artery of transplanted heart with other form of angina pectoris	414.06, 413.9	I25.758
Coronary artery disease involving transplanted heart with other form of angina pectoris	414.06, 413.9	I25.758
Coronary artery disease of native artery of transplanted heart with stable angina pectoris	414.06, 413.9	I25.758
Atherosclerosis of native coronary artery of transplanted heart with stable angina pectoris	414.06, 413.9	I25.758
Atherosclerosis of coronary artery of transplanted heart with stable angina pectoris	414.06, 413.9	I25.758
Atherosclerosis of coronary artery of transplanted heart with stable angina pectoris, unspecified vessel or lesion type	414.06, 413.9	I25.758
Atherosclerosis of coronary artery of transplanted heart with other form of angina pectoris, unspecified vessel or lesion type	414.06, 413.9	I25.758
Coronary artery disease involving transplanted heart with other form of angina pectoris, unspecified vessel or lesion type	414.06, 413.9	I25.758
Atherosclerosis of native coronary artery of transplanted heart with other forms of angina pectoris		I25.758
Coronary artery disease of transplanted heart with stable angina pectoris	414.07, 413.9	I25.758

© 2021 Eberly LA et al. *JAMA Network Open*.

Coronary artery disease of transplanted heart with stable angina pectoris, unspecified vessel or lesion type	414.07, 413.9	I25.758
Atherosclerosis of native coronary artery of transplanted heart with angina pectoris	414.06, 413.9	I25.759
Coronary artery disease involving transplanted heart with angina pectoris	414.06, 413.9	I25.759
Atherosclerosis of coronary artery of transplanted heart with angina pectoris	414.06, 413.9	I25.759
Coronary artery disease involving native artery of transplanted heart with unspecified angina pectoris	414.06, 413.9	I25.759
Coronary artery disease involving native artery of transplanted heart with angina pectoris	414.06, 413.9	I25.759
Atherosclerosis of native coronary artery of transplanted heart with unspecified angina pectoris	414.06, 413.9	I25.759
Coronary artery disease involving transplanted heart with angina pectoris, unspecified vessel or lesion type	414.06, 413.9	I25.759
Atherosclerosis of coronary artery of transplanted heart with angina pectoris, unspecified vessel or lesion type	414.06, 413.9	I25.759
Athscl native cor art of transplanted heart w unsp ang pctrs	414.06, 413.9	I25.759
Atherosclerosis of native coronary artery of transplanted heart with unspecified angina pectoris		I25.759
Atherosclerosis of bypass graft of coronary artery of transplanted heart with angina pectoris		I25.76
Atherosclerosis of bypass graft of coronary artery of transplanted heart with unstable angina	414.07, 411.1	I25.760

© 2021 Eberly LA et al. *JAMA Network Open*.

Coronary artery disease involving bypass graft of transplanted heart with unstable angina pectoris	414.07, 411.1	I25.760
Athscl bypass of cor art of txplt heart w unstable angina	414.07, 411.1	I25.760
Atherosclerosis of bypass graft of coronary artery of transplanted heart with unstable angina		I25.760
Atherosclerosis of bypass graft of coronary artery of transplanted heart with angina pectoris with documented spasm	414.07, 413.9	I25.761
Coronary artery disease involving bypass graft of transplanted heart with angina pectoris with documented spasm	414.07, 413.9	I25.761
Athscl bypass of cor art of txplt heart w ang pctrs w spasm	414.07, 413.9	I25.761
Atherosclerosis of bypass graft of coronary artery of transplanted heart with angina pectoris with documented spasm		I25.761
Athscl bypass of cor art of txplt heart w oth ang pctrs	414.07, 413.9	I25.768
Atherosclerosis of bypass graft of coronary artery of transplanted heart with other forms of angina pectoris	414.07, 413.9	I25.768
Coronary artery disease involving bypass graft of transplanted heart with other forms of angina pectoris	414.07, 413.9	I25.768
Coronary artery disease of bypass graft of transplanted heart with stable angina pectoris	414.07, 413.9	I25.768
Atherosclerosis of coronary artery bypass graft of transplanted heart with stable angina pectoris	414.07, 413.9	I25.768
Atherosclerosis of bypass graft of coronary artery of transplanted heart with other forms of angina pectoris		I25.768

© 2021 Eberly LA et al. *JAMA Network Open*.

Atherosclerosis of bypass graft of coronary artery of transplanted heart with angina pectoris	414.07, 413.9	I25.769
Coronary artery disease involving bypass graft of transplanted heart with unspecified angina pectoris	414.07, 413.9	I25.769
Coronary artery disease involving bypass graft of transplanted heart with angina pectoris	414.07, 413.9	I25.769
Atherosclerosis of bypass graft of coronary artery of transplanted heart with unspecified angina pectoris	414.07, 413.9	I25.769
Athscl bypass of cor art of txplt heart w unsp ang pctrs	414.07, 413.9	I25.769
Atherosclerosis of bypass graft of coronary artery of transplanted heart with unspecified angina pectoris		I25.769
Atherosclerosis of other coronary artery bypass graft(s) with angina pectoris		I25.79
Atherosclerosis of other coronary artery bypass graft with unstable angina pectoris	414.04, 411.1	I25.790
Coronary artery disease involving other coronary artery bypass graft with unstable angina pectoris	414.05, 411.1	I25.790
Atherosclerosis of other coronary artery bypass graft(s) with unstable angina pectoris	414.04, 411.1	I25.790
Atherosclerosis of other coronary artery bypass graft(s) with unstable angina pectoris		I25.790
Atherosclerosis of other coronary artery bypass graft with angina pectoris with documented spasm	414.04, 413.9	I25.791
Coronary artery disease involving other coronary artery bypass graft with angina pectoris with documented spasm	414.05, 413.9	I25.791

© 2021 Eberly LA et al. *JAMA Network Open*.

Atherosclerosis of other coronary artery bypass graft(s) with angina pectoris with documented spasm	414.04, 413.9	I25.791
Atherosclerosis of other coronary artery bypass graft(s) with angina pectoris with documented spasm		I25.791
Atherosclerosis of other coronary artery bypass graft with other form of angina pectoris	414.04, 413.9	I25.798
Coronary artery disease involving other coronary artery bypass graft with other forms of angina pectoris	414.05, 413.9	I25.798
Atherosclerosis of other coronary artery bypass graft(s) with other forms of angina pectoris	414.04, 413.9	I25.798
Coronary artery disease of other bypass graft with stable angina pectoris	414.05, 413.9	I25.798
Atherosclerosis of other coronary artery bypass graft(s) with other forms of angina pectoris		I25.798
Atherosclerosis of other coronary artery bypass graft with angina pectoris	414.04, 413.9	I25.799
Coronary artery disease involving other coronary artery bypass graft with unspecified angina pectoris	414.05, 413.9	I25.799
Atherosclerosis of other coronary artery bypass graft(s) with unspecified angina pectoris	414.04, 413.9	I25.799
Coronary artery disease involving other coronary artery bypass graft with angina pectoris	414.05, 413.9	I25.799
Atherosclerosis of other coronary artery bypass graft(s) with unspecified angina pectoris		I25.799
Other forms of chronic ischemic heart disease		I25.8

© 2021 Eberly LA et al. *JAMA Network Open*.

Atherosclerosis of other coronary vessels without angina pectoris		I25.81
Coronary atherosclerosis of autologous vein bypass graft	414.02	I25.810
Coronary atherosclerosis of nonautologous biological bypass graft	414.03	I25.810
Coronary atherosclerosis of artery bypass graft	414.04	I25.810
Coronary atherosclerosis of unspecified type of bypass graft(414.05)	414.05	I25.810
Coronary atherosclerosis of internal mammary artery	414.04	I25.810
Coronary atherosclerosis of bypass graft	414.04	I25.810
CAD (coronary artery disease), autologous vein bypass graft	414.02	I25.810
CAD (coronary artery disease), nonautologous biological bypass graft	414.03	I25.810
CAD (coronary artery disease) of bypass graft	414.05	I25.810
CAD (coronary artery disease) of artery bypass graft	414.05	I25.810
Atherosclerosis of coronary artery bypass graft	414.04	I25.810
Coronary atherosclerosis of autologous artery bypass graft	414.04	I25.810
CAD of autologous arterial graft	414.04	I25.810
Coronary atherosclerosis of autologous bypass graft	414.02	I25.810
CAD of autologous bypass graft	414.02	I25.810
Hardening of bypass graft of coronary artery	414.04	I25.810
Coronary atherosclerosis of vein bypass graft	414.05	I25.810
Atherosclerosis of coronary artery bypass graft w/o angina pectoris	414.05	I25.810
Atherosclerosis of coronary artery bypass graft without angina pectoris	414.05	I25.810

© 2021 Eberly LA et al. *JAMA Network Open*.

Arteriosclerosis of internal mammary artery coronary artery bypass graft	414.04	I25.810
Arteriosclerosis of nonautologous coronary artery bypass graft	414.03	I25.810
Arteriosclerosis of autologous vein coronary artery bypass graft	414.02	I25.810
Atherosclerotic heart disease of artery bypass graft	414.04	I25.810
Arteriosclerosis of coronary artery bypass graft	414.04	I25.810
Atherosclerosis of nonbiological coronary artery bypass graft	414.05	I25.810
Coronary atherosclerosis of unspecified type of bypass graft	414.04	I25.810
Arteriosclerosis of arterial coronary artery bypass graft	414.05	I25.810
Arteriosclerosis of autologous arterial coronary artery bypass graft	414.04	I25.810
Arteriosclerosis of autologous coronary artery bypass graft	414.05	I25.810
Arteriosclerosis of bypass graft of coronary artery	414.04	I25.810
Coronary atherosclerosis of internal mammary artery bypass graft	414.04	I25.810
Coronary atherosclerosis of autologous artery bypass graft without angina	414.04	I25.810
CAD of autologous artery bypass graft without angina	414.04	I25.810
Coronary atherosclerosis of autologous vein bypass graft without angina	414.02	I25.810
CAD of autologous vein bypass graft without angina	414.02	I25.810
Coronary artery disease involving autologous vein bypass graft	414.02	I25.810
Coronary artery disease involving nonautologous biological coronary bypass graft	414.03	I25.810

© 2021 Eberly LA et al. *JAMA Network Open*.

Coronary artery disease involving coronary bypass graft	414.05	I25.810
Coronary artery disease involving autologous artery coronary bypass graft without angina pectoris	414.04	I25.810
Coronary artery disease involving coronary bypass graft without angina pectoris	414.05	I25.810
Coronary artery disease involving autologous artery coronary bypass graft	414.04	I25.810
Coronary artery disease involving nonautologous biological coronary bypass graft without angina pectoris	414.03	I25.810
Coronary artery disease involving autologous vein coronary bypass graft without angina pectoris	414.02	I25.810
Coronary artery disease involving coronary bypass graft of native heart	414.04	I25.810
Coronary artery disease involving coronary bypass graft of native heart without angina pectoris	414.05	I25.810
Atherosclerosis of coronary artery bypass graft of native heart	414.04	I25.810
Atherosclerosis of coronary artery bypass graft of native heart without angina pectoris	414.05	I25.810
Arteriosclerosis of nonautologous coronary artery bypass graft without angina pectoris	414.03	I25.810
Coronary arteriosclerosis after coronary artery bypass grafting	414.05	I25.810
Atherosclerosis of coronary artery bypass graft without angina pectoris, unspecified whether native or transplanted heart	414.05	I25.810
Coronary artery disease involving other coronary artery bypass graft	414.05	I25.810

© 2021 Eberly LA et al. *JAMA Network Open*.

Coronary artery disease involving other coronary artery bypass graft without angina pectoris	414.05	I25.810
Atherosclerosis of other coronary artery bypass graft without angina pectoris	414.04	I25.810
Atherosclerotic heart disease of nonautologous biological bypass graft	414.03	I25.810
Atherosclerosis of autologous vein coronary artery bypass graft	414.02	I25.810
Atherosclerosis of nonautologous biological coronary artery bypass graft	414.03	I25.810
Atherosclerosis of coronary artery bypass graft(s) without angina pectoris	414.05	I25.810
Atherosclerosis of other coronary artery bypass graft	414.04	I25.810
Coronary artery disease involving coronary bypass graft without angina pectoris, unspecified whether native or transplanted heart	414.05	I25.810
Atherosclerosis of coronary artery bypass graft, angina presence unspecified, unspecified whether native or transplanted heart	414.04	I25.810
Atherosclerosis of nonautologous biological coronary artery bypass graft, angina presence unspecified	414.03	I25.810
Coronary artery disease involving coronary bypass graft, angina presence unspecified, unspecified whether native or transplanted heart	414.05	I25.810
Coronary artery disease involving autologous vein bypass graft, angina presence unspecified	414.02	I25.810
Coronary artery disease involving coronary bypass graft of native heart, angina presence unspecified	414.04	I25.810

© 2021 Eberly LA et al. *JAMA Network Open*.

Atherosclerosis of other coronary artery bypass graft, angina presence unspecified	414.04	I25.810
Coronary artery disease involving nonautologous biological coronary bypass graft, angina presence unspecified	414.03	I25.810
Atherosclerosis of autologous vein coronary artery bypass graft, angina presence unspecified	414.02	I25.810
Atherosclerosis of coronary artery bypass graft of native heart, angina presence unspecified	414.04	I25.810
Coronary artery disease involving autologous artery coronary bypass graft, angina presence unspecified	414.04	I25.810
Arteriosclerosis of autologous arterial coronary artery bypass graft, angina presence unspecified	414.04	I25.810
Coronary artery disease involving other coronary artery bypass graft, angina presence unspecified	414.05	I25.810
Atherosclerosis of CABG w/o angina pectoris	414.05	I25.810
Atherosclerosis of coronary artery bypass graft(s) without angina pectoris		I25.810
Coronary atherosclerosis of native coronary artery of transplanted heart	414.06	I25.811
Coronary atherosclerosis of coronary artery of transplanted heart	414.06	I25.811
Coronary arteriosclerosis of transplanted heart	414.06	I25.811
Coronary atherosclerosis transplanted heart	414.06	I25.811
CAD (coronary artery disease), native artery transplanted heart	414.06	I25.811
Accelerated coronary artery disease in transplanted heart	414.06	I25.811

© 2021 Eberly LA et al. *JAMA Network Open*.

Coronary artery disease of transplanted heart	414.06	I25.811
Coronary atherosclerosis of artery of transplanted heart	414.06	I25.811
Coronary atherosclerosis of native artery of transplanted heart	414.06	I25.811
Coronary arteriosclerosis of native coronary artery of transplanted heart	414.06	I25.811
Atherosclerosis of native coronary artery of transplanted heart without angina pectoris	414.06	I25.811
Coronary atherosclerosis of transplanted heart	414.06	I25.811
Atherosclerotic heart disease of coronary artery of transplanted heart	414.06	I25.811
Atherosclerosis of coronary artery of transplanted heart	414.06	I25.811
Coronary artery disease involving native artery of transplanted heart	414.06	I25.811
Coronary artery disease involving native artery of transplanted heart without angina pectoris	414.06	I25.811
Atherosclerosis of native coronary artery of transplanted heart	414.06	I25.811
Coronary artery disease involving transplanted heart	414.06	I25.811
Atherosclerosis of coronary artery of transplanted heart without angina pectoris	414.06	I25.811
Coronary artery disease involving transplanted heart without angina pectoris	414.06	I25.811
Artscl ntv coron trnspl heart	414.06	I25.811
Atherosclerosis of coronary artery of transplanted heart, angina presence unspecified, unspecified vessel or lesion type	414.06	I25.811

© 2021 Eberly LA et al. *JAMA Network Open*.

Coronary artery disease involving transplanted heart, angina presence unspecified, unspecified vessel or lesion type	414.06	I25.811
Atherosclerosis of native coronary artery of transplanted heart, angina presence unspecified	414.06	I25.811
Coronary artery disease involving native artery of transplanted heart, angina presence unspecified	414.06	I25.811
Atherosclerosis of coronary artery of transplanted heart without angina pectoris, unspecified vessel or lesion type	414.06	I25.811
Coronary artery disease involving transplanted heart without angina pectoris, unspecified vessel or lesion type	414.06	I25.811
Athscl native cor art of transplanted heart w/o ang pctrs	414.06	I25.811
Atherosclerosis of native coronary artery of transplanted heart without angina pectoris		I25.811
Coronary atherosclerosis of bypass graft of transplanted heart	414.07	I25.812
CAD (coronary artery disease), bypass graft transplanted heart	414.07	I25.812
Atherosclerosis of bypass graft of coronary artery of transplanted heart without angina pectoris	414.07	I25.812
Arteriosclerosis of coronary artery bypass graft of transplanted heart	414.07	I25.812
Coronary artery disease involving bypass graft of transplanted heart	414.07	I25.812
Coronary artery disease involving bypass graft of transplanted heart without angina pectoris	414.07	I25.812
Atherosclerosis of coronary artery bypass graft of transplanted heart	414.07	I25.812

© 2021 Eberly LA et al. *JAMA Network Open*.

Coronary artery disease involving bypass graft of transplanted heart, angina presence unspecified	414.07	I25.812
Atherosclerosis of coronary artery bypass graft of transplanted heart, angina presence unspecified	414.07	I25.812
Athscl bypass of cor art of transplanted heart w/o ang pctrs	414.07	I25.812
Atherosclerosis of bypass graft of coronary artery of transplanted heart without angina pectoris		I25.812
Chronic total occlusion of coronary artery(414.2)	414.2	I25.82
Complete occlusion of coronary artery, chronic	414.00, 414.2	I25.82
Total occlusion of coronary artery, chronic	414.00, 414.2	I25.82
Coronary artery chronic total occlusion	414.00, 414.2	I25.82
Chronic total occlusion of coronary artery	414.00, 414.2	I25.82
Chronic total occlusion of coronary artery		I25.82
Coronary atherosclerosis due to lipid rich plaque (CODE)	414.3	I25.83
Coronary atherosclerosis due to lipid rich plaque		I25.83
Coronary atherosclerosis due to calcified coronary lesion(414.4)	414.4	I25.84
Coronary atherosclerosis due to severely calcified coronary lesion	414.00, 414.4	I25.84
Coronary atherosclerosis due to calcified coronary lesion (CODE)	414.00, 414.4	I25.84
Coronary atherosclerosis due to calcified coronary lesion		I25.84
Cardiac microvascular disease	414.8	I25.89
Other specified forms of chronic ischemic heart disease	414.8	I25.89
Chronic coronary insufficiency	414.8	I25.89
CCI (chronic coronary insufficiency)	414.8	I25.89

© 2021 Eberly LA et al. *JAMA Network Open*.

Symptomatic old MI (myocardial infarction)	414.8	I25.89
Chronotropic incompetence with ischemic heart disease	414.9	I25.89
Symptomatic old myocardial infarction	414.8	I25.89
Other forms of chronic ischemic heart disease	414.8	I25.89
Ischemic heart disease with chronotropic incompetence	414.9	I25.89
Other forms of chronic ischemic heart disease (CODE)	414.8	I25.89
Other forms of chronic ischemic heart disease		I25.89
Ischemic chest pain	786.5	I25.9
Chest pain due to myocardial ischemia	786.5	I25.9
Chest pain due to myocardial ischemia, unspecified ischemic chest pain type	786.5	I25.9
Chronic ischemic heart disease, unspecified	414.9	I25.9
Chronic ischemic heart disease	414.9	I25.9
Ischemic heart disease	414.9	I25.9
Myocardial ischemia	414.8	I25.9
Chronic ischemia, myocardial	414.8	I25.9
Ischemic heart disease or syndrome, chronic	414.9	I25.9
Cardiac ischemia	414.9	I25.9
IHD (ischemic heart disease)	414.9	I25.9
Ischemic heart disease or syndrome	414.9	I25.9
Myocardial ischemia or hypoxia	414.8	I25.9
Chronic myocardial ischemia	414.8	I25.9
Poor blood flow to the heart muscle	414.9	I25.9
Ischemia of heart, chronic	414.9	I25.9
Ischemic heart disease, chronic	414.9	I25.9
Ischemia, myocardial, chronic	414.8	I25.9
Chronic myocardial infarction	414.9	I25.9
Myocardial infarction, chronic	414.9	I25.9
Supply ischemia of myocardium	414.9	I25.9
Sleep related coronary artery ischemia	414.9	I25.9

© 2021 Eberly LA et al. *JAMA Network Open*.

Asymptomatic coronary heart disease	414.9	I25.9
Sleep related myocardial ischemia	414.9	I25.9
Myocardial ischemia due to inadequate myocardial oxygen supply	414.9	I25.9
Subacute ischemic heart disease	414.9	I25.9
Resting ischemia due to ischemic heart disease	443.9, 414.9	I25.9
Chronic ischemic heart disease, unspecified		I25.9
Mixed myocardial ischemia and ST elevation myocardial infarction (STEMI) involving left main coronary artery	414.9, 410.10	I25.9, I21.01
Mixed myocardial ischemia and ST elevation myocardial infarction (STEMI) involving left anterior descending (LAD) coronary artery	414.9, 410.10	I25.9, I21.02
Mixed myocardial ischemia and ST elevation myocardial infarction (STEMI) involving right coronary artery	414.9, 410.10	I25.9, I21.11
Mixed myocardial ischemia and ST elevation myocardial infarction (STEMI) involving left circumflex coronary artery	414.9, 410.80	I25.9, I21.21
Mixed myocardial ischemia and ST elevation myocardial infarction (STEMI)	414.9, 410.90	I25.9, I21.3
Mixed myocardial ischemia and non-ST elevation myocardial infarction	414.9, 410.70	I25.9, I21.4
Mixed myocardial ischemia and non-ST elevation myocardial infarction (NSTEMI)	414.9, 410.70	I25.9, I21.4
Mixed myocardial ischemia and infarction	410.9	I25.9, I21.9
Cardiomyopathy with implantable cardioverter-defibrillator	425.4, V45.02	I42.9, Z95.810
Systolic heart failure secondary to coronary artery disease	428.20, 414.00	I50.20, I25.10
Heart failure, systolic, due to CAD	428.20, 414.00	I50.20, I25.10
ACC/AHA stage C systolic heart failure due to ischemic cardiomyopathy	428.9, 414.8	I50.20, I25.5

© 2021 Eberly LA et al. *JAMA Network Open*.

ACC/AHA stage B systolic heart failure due to ischemic cardiomyopathy	428.9, 414.8	I50.20, I25.5
Diastolic heart failure secondary to coronary artery disease	428.30, 414.00	I50.30, I25.10
Heart failure, diastolic, due to CAD	428.30, 414.00	I50.30, I25.10
Heart failure, diastolic, due to CAD, unspecified failure chronicity	428.30, 414.00	I50.30, I25.10
Diastolic heart failure secondary to coronary artery disease, unspecified failure chronicity	428.30, 414.00	I50.30, I25.10
Heart failure, diastolic, due to CAD, acute	428.31	I50.31, I25.10
Diastolic heart failure secondary to coronary artery disease, acute	428.31	I50.31, I25.10
Acute diastolic heart failure secondary to coronary artery disease	428.31	I50.31, I25.10
Diastolic heart failure secondary to coronary artery disease, chronic	428.32	I50.32, I25.10
Heart failure, diastolic, due to CAD, chronic	428.32	I50.32, I25.10
Chronic diastolic heart failure secondary to coronary artery disease	428.32	I50.32, I25.10
Heart failure, diastolic, due to CAD, acute on chronic	428.33, 414.00	I50.33, I25.10
Diastolic heart failure secondary to coronary artery disease, acute on chronic	428.33, 414.00	I50.33, I25.10
Acute on chronic diastolic heart failure secondary to coronary artery disease	428.33, 414.00	I50.33, I25.10
Acute on chronic diastolic heart failure due to coronary artery disease	428.33, 414.00	I50.33, I25.10
ACC/AHA stage B congestive heart failure due to ischemic cardiomyopathy	428.0, 414.8	I50.9, I25.5
ACC/AHA stage C congestive heart failure due to ischemic cardiomyopathy	428.0, 414.8	I50.9, I25.5
Cerebral infarction due to unspecified occlusion or stenosis of precerebral arteries		I63.2

© 2021 Eberly LA et al. *JAMA Network Open*.

Occlusion and stenosis of multiple and bilateral precerebral arteries with cerebral infarction	433.31	I63.20
Occlusion and stenosis of unspecified precerebral artery with cerebral infarction	433.91	I63.20
Occlusion and stenosis of precerebral artery with cerebral infarction	433.91	I63.20
Multiple precerebral artery occlusions with cerebral infarction	433.31	I63.20
Precerebral artery stenosis/occlusion with infarction	433.91	I63.20
Precerebral artery stenosis/occlusion, multiple/bilater, with infarct	433.31	I63.20
Precerebral occlusion with cerebral infarction	433.91	I63.20
Stenosis of precerebral artery with cerebral infarction	433.91	I63.20
Extracranial artery stenosis with infarction	433.91	I63.20
Precerebral artery occlusion with infarction	433.91	I63.20
Occlusion of precerebral artery with infarction	433.91	I63.20
Occlusion and stenosis of multiple and bilateral arteries, with cerebral infarction	433.31	I63.20
Cerebral infarction due to occlusion or stenosis of precerebral arteries	433.91	I63.20
Multiple and bilateral precerebral artery stenosis with infarction	433.31	I63.20
Occlusion of multiple and bilateral precerebral arteries with cerebral infarction	433.31	I63.20
Cerebral infarction due to occlusion or stenosis of precerebral artery	433.91	I63.20
Cerebral infarction due to stenosis of precerebral artery	433.91	I63.20

© 2021 Eberly LA et al. *JAMA Network Open*.

Infarction due to disorder of precerebral artery	433.91	I63.20
Mult precerebral occ w/ infarc	433.31	I63.20
Precerebral occl w/ infarct	433.91	I63.20
Cerebrovascular accident (CVA) due to occlusion of precerebral artery	433.91	I63.20
Stroke due to stenosis of precerebral artery	433.91	I63.20
Stroke due to occlusion of precerebral artery	433.91	I63.20
Cerebrovascular accident (CVA) due to stenosis of precerebral artery	433.91	I63.20
Cerebral infarction due to occlusion of precerebral artery	434.91	I63.20
Cerebral infarction due to unspecified occlusion or stenosis of unspecified precerebral arteries	433.91	I63.20
Bilateral cerebral infarction due to occlusion of precererbral artery	434.91	I63.20
Cereb infarction due to unspecified occls or stenos of unsp precerb art	433.91	I63.20
Cerebral infarction due to unspecified occlusion or stenosis of unspecified precerebral arteries		I63.20
Cerebral infarction due to unspecified occlusion or stenosis of vertebral arteries		I63.21
Cerebral infarction due to unspecified occlusion or stenosis of right vertebral artery	433.21	I63.211
Cerebral infarction involving right vertebral artery	433.21	I63.211
Cerebral infarction due to occlusion of right vertebral artery	433.21	I63.211
Cerebral infarction due to stenosis of right vertebral artery	433.21	I63.211
Cerebral infarction involving vertebral artery, right	433.21	I63.211

© 2021 Eberly LA et al. *JAMA Network Open*.

Cerebrovascular accident (CVA) due to occlusion of right vertebral artery	433.21	I63.211
Stroke due to stenosis of right vertebral artery	433.21	I63.211
Stroke due to occlusion of right vertebral artery	433.21	I63.211
Cerebrovascular accident (CVA) due to stenosis of right vertebral artery	433.21	I63.211
Cerebral infarction due to unspecified occlusion or stenosis of right vertebral arteries	433.21	I63.211
Cerebral infarction due to unspecified occlusion or stenosis of right vertebral artery	433.21	I63.211
Cerebral infarction due to unspecified occlusion or stenosis of right vertebral artery (CODE)	433.21	I63.211
Cerebral infarction due to unspecified occlusion or stenosis of right vertebral artery		I63.211
Acute arterial ischemic stroke, vertebrobasilar, brainstem, right	434.91	I63.211, I63.22
Arterial ischemic stroke, vertebrobasilar, brainstem, acute, right	434.91	I63.211, I63.22
Acute ischemic VBA thalamic stroke, right	434.91	I63.211, I63.22
Acute arterial ischemic stroke, vertebrobasilar, thalamic, right	434.91	I63.211, I63.22
Acute ischemic vertebrobasilar artery thalamic stroke, right	434.91	I63.211, I63.22
Arterial ischemic stroke, vertebrobasilar, thalamic, acute, right	434.91	I63.211, I63.22
Acute ischemic vertebrobasilar artery brainstem stroke, right	434.91	I63.211, I63.22
Acute ischemic VBA brainstem stroke, right	434.91	I63.211, I63.22

© 2021 Eberly LA et al. *JAMA Network Open*.

Acute ischemic vertebrobasilar artery brainstem stroke involving right-sided vessel	434.91	I63.211, I63.22
Acute ischemic vertebrobasilar artery thalamic stroke involving right-sided vessel	434.91	I63.211, I63.22
Cerebral infarction due to unspecified occlusion or stenosis of left vertebral artery	433.21	I63.212
Cerebral infarction involving left vertebral artery	433.21	I63.212
Cerebral infarction due to occlusion of left vertebral artery	433.21	I63.212
Cerebral infarction due to stenosis of left vertebral artery	433.21	I63.212
Cerebral infarction involving vertebral artery, left	433.21	I63.212
Cerebrovascular accident (CVA) due to occlusion of left vertebral artery	433.21	I63.212
Stroke due to occlusion of left vertebral artery	433.21	I63.212
Stroke due to stenosis of left vertebral artery	433.21	I63.212
Cerebrovascular accident (CVA) due to stenosis of left vertebral artery	433.21	I63.212
Cerebral infarction due to unspecified occlusion or stenosis of left vertebral arteries	433.21	I63.212
Cerebral infarction due to unspecified occlusion or stenosis of left vertebral artery	433.21	I63.212
Cerebral infarction due to unspecified occlusion or stenosis of left vertebral artery (CODE)	433.21	I63.212
Cerebral infarction due to unspecified occlusion or stenosis of left vertebral artery		I63.212
Acute ischemic VBA brainstem stroke, left	434.91	I63.212, I63.22

© 2021 Eberly LA et al. *JAMA Network Open*.

Arterial ischemic stroke, vertebrobasilar, thalamic, acute, left	434.91	I63.212, I63.22
Acute ischemic VBA thalamic stroke, left	434.91	I63.212, I63.22
Acute arterial ischemic stroke, vertebrobasilar, thalamic, left	434.91	I63.212, I63.22
Acute ischemic vertebrobasilar artery thalamic stroke, left	434.91	I63.212, I63.22
Arterial ischemic stroke, vertebrobasilar, brainstem, acute, left	434.91	I63.212, I63.22
Acute arterial ischemic stroke, vertebrobasilar, brainstem, left	434.91	I63.212, I63.22
Acute ischemic vertebrobasilar artery brainstem stroke, left	434.91	I63.212, I63.22
Acute ischemic vertebrobasilar artery brainstem stroke involving left-sided vessel	434.91	I63.212, I63.22
Acute ischemic vertebrobasilar artery thalamic stroke involving left-sided vessel	434.91	I63.212, I63.22
Cerebral infarction due to bilateral stenosis of vertebral arteries	433.31	I63.213
Cerebral infarction due to bilateral occlusion of vertebral arteries	433.31	I63.213
Cerebrovascular accident (CVA) due to bilateral stenosis of vertebral arteries	433.31	I63.213
Cerebrovascular accident (CVA) due to bilateral occlusion of vertebral arteries	433.31	I63.213
Cerebral infarction due to unspecified occlusion or stenosis of bilateral vertebral arteries	433.21	I63.213
Cerebral infarction due to unspecified occlusion or stenosis of bilateral vertebral arteries		I63.213
Occlusion and stenosis of vertebral artery with cerebral infarction	433.21	I63.219
Vertebral artery stroke	433.21	I63.219
Stroke, vertebral artery	433.21	I63.219
Vertebral artery stenosis/occlusion with infarction	433.21	I63.219

© 2021 Eberly LA et al. *JAMA Network Open*.

Vertebral artery stenosis with cerebral infarction	433.21	I63.219
Cerebral infarction due to unspecified occlusion or stenosis of unspecified vertebral art	433.21	I63.219
Cerebral infarction involving vertebral artery	433.21	I63.219
Cerebral infarction associated with stenosis of vertebral artery	433.21	I63.219
Cerebral infarction due to occlusion of vertebral artery	433.21	I63.219
Cerebral infarction due to stenosis of vertebral artery	433.21	I63.219
Cerebral infarction involving vertebral artery, unspecified laterality	433.21	I63.219
Cerebrovascular accident (CVA) due to occlusion of vertebral artery	433.21	I63.219
Cerebrovascular accident (CVA) due to stenosis of vertebral artery	433.21	I63.219
Stroke due to occlusion of vertebral artery	433.21	I63.219
Stroke due to stenosis of vertebral artery	433.21	I63.219
Cerebral infarction due to unspecified occlusion or stenosis of unspecified vertebral arteries	433.21	I63.219
Cerebral infarction due to stenosis of vertebral artery, unspecified blood vessel laterality	433.21	I63.219
Cerebral infarction due to occlusion of vertebral artery, unspecified blood vessel laterality	433.21	I63.219
Cerebrovascular accident (CVA) due to stenosis of vertebral artery, unspecified blood vessel laterality	433.21	I63.219
Cerebrovascular accident (CVA) due to occlusion of vertebral artery, unspecified blood vessel laterality	433.21	I63.219

© 2021 Eberly LA et al. *JAMA Network Open*.

Cerebral infarction due to unspecified occlusion or stenosis of unspecified vertebral artery		I63.219
Arterial ischemic stroke, vertebrobasilar, brainstem, acute	434.91	I63.219, I63.22
Acute arterial ischemic stroke, vertebrobasilar, brainstem	434.91	I63.219, I63.22
Arterial ischemic stroke, vertebrobasilar, thalamic, acute	434.91	I63.219, I63.22
Acute arterial ischemic stroke, vertebrobasilar, thalamic	434.91	I63.219, I63.22
Acute ischemic vertebrobasilar artery brainstem stroke	434.91	I63.219, I63.22
Acute ischemic vertebrobasilar artery thalamic stroke	434.91	I63.219, I63.22
Acute ischemic VBA brainstem stroke	434.91	I63.219, I63.22
Acute ischemic VBA thalamic stroke	434.91	I63.219, I63.22
Arterial ischemic stroke, vertebrobasilar, thalamic, acute, unspecified laterality	434.91	I63.219, I63.22
Acute arterial ischemic stroke, vertebrobasilar, thalamic, unspecified laterality	434.91	I63.219, I63.22
Arterial ischemic stroke, vertebrobasilar, brainstem, acute, unspecified laterality	434.91	I63.219, I63.22
Acute ischemic vertebrobasilar artery thalamic stroke, unspecified laterality	434.91	I63.219, I63.22
Acute ischemic VBA brainstem stroke, unspecified laterality	434.91	I63.219, I63.22
Acute arterial ischemic stroke, vertebrobasilar, brainstem, unspecified laterality	434.91	I63.219, I63.22
Acute ischemic VBA thalamic stroke, unspecified laterality	434.91	I63.219, I63.22
Acute ischemic vertebrobasilar artery brainstem stroke, unspecified laterality	434.91	I63.219, I63.22
Occlusion and stenosis of basilar artery with cerebral infarction	433.01	I63.22

© 2021 Eberly LA et al. *JAMA Network Open*.

Occlusion and stenosis of basilar artery, with cerebral infarction	433.01	I63.22
Basilar artery stenosis/occlusion with infarction	433.01	I63.22
Basilar artery stenosis with infarction	433.01	I63.22
Cerebral infarction due to unspecified occlusion or stenosis of basilar artery	433.01	I63.22
Basilar artery occlusion with cerebral infarction	433.01	I63.22
Cerebral infarction due to basilar artery occlusion	433.01	I63.22
Cerebral infarction due to stenosis of basilar artery	433.01	I63.22
Cerebral infarction involving basilar artery	433.01	I63.22
Stroke due to occlusion of basilar artery	433.01	I63.22
Cerebrovascular accident (CVA) due to occlusion of basilar artery	433.01	I63.22
Stroke due to stenosis of basilar artery	433.01	I63.22
Cerebrovascular accident (CVA) due to stenosis of basilar artery	433.01	I63.22
Cerebral infarction due to unspecified occlusion or stenosis of basilar arteries	433.01	I63.22
Cerebral infarction due to unspecified occlusion or stenosis of basilar artery	433.01	I63.22
Cerebral infarction due to unspecified occlusion or stenosis of basilar artery (CODE)	433.01	I63.22
Cerebral infarction due to unspecified occlusion or stenosis of basilar artery		I63.22
Cerebral infarction due to unspecified occlusion or stenosis of carotid arteries		I63.23
Arterial ischemic stroke, ICA (internal carotid artery), right, acute	434.91	I63.231
Acute right arterial ischemic stroke, ICA (internal carotid artery)	434.91	I63.231
Acute ischemic right ICA stroke	434.91	I63.231
Arterial ischemic stroke, ICA, right, acute	434.91	I63.231

© 2021 Eberly LA et al. *JAMA Network Open*.

Acute right arterial ischemic stroke, internal carotid artery (ICA)	434.91	I63.231
Acute ischemic right internal carotid artery (ICA) stroke	434.91	I63.231
Cerebral infarction due to internal carotid artery occlusion, right	433.11	I63.231
Stenosis of internal carotid artery with cerebral infarction, right	433.11	I63.231
Cerebral infarction due to occlusion of right internal carotid artery	433.11	I63.231
Stenosis of right internal carotid artery with cerebral infarction	433.11	I63.231
Cerebral infarction involving right carotid artery	433.11	I63.231
Cerebral infarction due to stenosis of right carotid artery	433.11	I63.231
Cerebral infarction due to occlusion of right carotid artery	433.11	I63.231
Cerebral infarction due to vascular stenosis, right	433.11	I63.231
Cerebral infarction due to vascular occlusion, right	433.11	I63.231
Cerebral infarction involving carotid artery, right	433.11	I63.231
Stroke due to stenosis of right carotid artery	433.11	I63.231
Cerebrovascular accident (CVA) due to stenosis of right carotid artery	433.11	I63.231
Cerebrovascular accident (CVA) due to occlusion of right carotid artery	433.11	I63.231
Stroke due to occlusion of right carotid artery	433.11	I63.231
Cerebral infarction due to unspecified occlusion or stenosis of right carotid arteries	433.11	I63.231
Cerebral infarction due to unspecified occlusion or stenosis of right carotid artery	433.11	I63.231

© 2021 Eberly LA et al. *JAMA Network Open*.

Cerebral infarction due to unspecified occlusion or stenosis of right carotid arteries		I63.231
Arterial ischemic stroke, ICA (internal carotid artery), left, acute	434.91	I63.232
Acute left arterial ischemic stroke, ICA (internal carotid artery)	434.91	I63.232
Acute ischemic left ICA stroke	434.91	I63.232
Arterial ischemic stroke, ICA, left, acute	434.91	I63.232
Acute left ICA ischemic stroke	434.91	I63.232
Acute ischemic left internal carotid artery (ICA) stroke	434.91	I63.232
Cerebral infarction due to internal carotid artery occlusion, left	433.11	I63.232
Stenosis of internal carotid artery with cerebral infarction, left	433.11	I63.232
Cerebral infarction due to occlusion of left internal carotid artery	433.11	I63.232
Stenosis of left internal carotid artery with cerebral infarction	433.11	I63.232
Cerebral infarction involving left carotid artery	433.11	I63.232
Cerebral infarction due to stenosis of left carotid artery	433.11	I63.232
Cerebral infarction due to occlusion of left carotid artery	433.11	I63.232
Cerebral infarction due to vascular stenosis, left	433.11	I63.232
Cerebral infarction involving carotid artery, left	433.11	I63.232
Cerebral infarction due to vascular occlusion, left	433.11	I63.232
Stroke due to occlusion of left carotid artery	433.11	I63.232
Cerebrovascular accident (CVA) due to occlusion of left carotid artery	433.11	I63.232

© 2021 Eberly LA et al. *JAMA Network Open*.

Stroke due to stenosis of left carotid artery	433.11	I63.232
Cerebrovascular accident (CVA) due to stenosis of left carotid artery	433.11	I63.232
Cerebral infarction due to unspecified occlusion or stenosis of left carotid arteries	433.11	I63.232
Cerebral infarction due to unspecified occlusion or stenosis of left carotid arteries		I63.232
Cerebral infarction due to bilateral stenosis of carotid arteries	433.31	I63.233
Cerebral infarction due to bilateral occlusion of carotid arteries	433.31	I63.233
Cerebrovascular accident (CVA) due to bilateral occlusion of carotid arteries	433.31	I63.233
Cerebrovascular accident (CVA) due to bilateral stenosis of carotid arteries	433.31	I63.233
Cerebral infarction due to unspecified occlusion or stenosis of bilateral carotid arteries	433.11	I63.233
Cerebral infarction due to unspecified occlusion or stenosis of bilateral carotid arteries		I63.233
Occlusion and stenosis of carotid artery with cerebral infarction	433.11	I63.239
Occlusion and stenosis of carotid artery, with cerebral infarction	433.11	I63.239
Symptomatic carotid artery stenosis with infarction	433.11	I63.239
Asymptomatic carotid artery stenosis with infarction	433.11	I63.239
Carotid artery stenosis/occlusion with infarction	433.11	I63.239
Carotid artery occlusion with infarction	433.11	I63.239
Carotid stenosis, symptomatic, with infarction	433.11	I63.239

© 2021 Eberly LA et al. *JAMA Network Open*.

Carotid artery occlusion with cerebral infarction	433.11	I63.239
Cerebral infarction due to unspecified occlusion or stenosis of unspecified carotid artery	433.11	I63.239
Cerebral infarction due to internal carotid artery occlusion	433.11	I63.239
Cerebral infarction due to internal carotid artery occlusion, unspecified laterality	433.11	I63.239
Stenosis of internal carotid artery with cerebral infarction	433.11	I63.239
Stenosis of internal carotid artery with cerebral infarction, unspecified laterality	433.11	I63.239
Cerebral infarction involving carotid artery	433.11	I63.239
Cerebral infarction due to carotid artery occlusion	433.11	I63.239
Cerebral infarction due to stenosis of carotid artery	433.11	I63.239
Cerebral infarction due to carotid artery stenosis	433.11	I63.239
Carotid artery stenosis with cerebral infarction	433.11	I63.239
Cerebral infarction due to vascular stenosis, unspecified laterality	433.11	I63.239
Cerebral infarction due to vascular occlusion, unspecified laterality	433.11	I63.239
Cerebral infarction involving carotid artery, unspecified laterality	433.11	I63.239
Cerebral infarction due to occlusion of carotid artery	433.11	I63.239
Stroke due to stenosis of carotid artery	433.11	I63.239
Cerebrovascular accident (CVA) due to occlusion of carotid artery	433.11	I63.239
Stroke due to occlusion of carotid artery	433.11	I63.239
Cerebrovascular accident (CVA) due to stenosis of carotid artery	433.11	I63.239

© 2021 Eberly LA et al. *JAMA Network Open*.

Cerebral infarction due to unspecified occlusion or stenosis of unspecified carotid arteries	433.11	I63.239
Cerebrovascular accident (CVA) due to occlusion of carotid artery, unspecified blood vessel laterality	433.11	I63.239
Cerebral infarction due to occlusion of carotid artery, unspecified blood vessel laterality	433.11	I63.239
Cerebral infarction due to carotid artery stenosis, unspecified blood vessel laterality	433.11	I63.239
Cerebrovascular accident (CVA) due to stenosis of carotid artery, unspecified blood vessel laterality	433.11	I63.239
Cerebral infarction due to unspecified occlusion or stenosis of unspecified carotid artery		I63.239
Arterial ischemic stroke, vertebrobasilar, cerebellar, acute	434.91	I63.29
Acute arterial ischemic stroke, vertebrobasilar, cerebellar	434.91	I63.29
Acute ischemic vertebrobasilar artery cerebellar stroke	434.91	I63.29
Ac ischemic VBA cerebellar stroke	434.91	I63.29
Occlusion of anterior choroidal artery with cerebral infarction	433.81	I63.29
Cerebral infarction due to stenosis of other precerebral artery	433.91	I63.29
Cerebral infarction due to occlusion of other precerebral artery	433.81	I63.29
Cerebral infarction due to unspecified occlusion or stenosis of other precerebral arteries	433.81	I63.29
Cerebrovascular accident (CVA) due to stenosis of other precerebral artery	433.81	I63.29
Cerebrovascular accident (CVA) due to occlusion of other precerebral artery	433.81	I63.29

© 2021 Eberly LA et al. *JAMA Network Open*.

Cerebral infarction due to unspecified occlusion or stenosis of other precerebral arteries		I63.29
Cerebral infarction due to thrombosis of cerebral arteries		I63.3
Cerebral thrombosis with cerebral infarction	434.01	I63.30
Cerebral infarction due to thrombosis of cerebral artery	434.01	I63.30
Thrombotic stroke involving cerebral artery	434.01	I63.30
Thrombotic cerebral infarction	434.01	I63.30
Cerebral infarction due to cerebral venous thrombosis	434.01	I63.30
Cerebral infarction due to thrombosis of right cerebral artery	434.01	I63.30
Cerebral infarction due to thrombosis of left cerebral artery	434.01	I63.30
Stroke due to thrombosis of cerebral artery	434.01	I63.30
Cerebrovascular accident (CVA) due to thrombosis of cerebral artery	434.01	I63.30
Cerebral infarction due to thrombosis of unspecified cerebral artery	434.01	I63.30
Cerebral infarction due to thrombosis	434.01	I63.30
Cerebral infarction due to thrombosis of unspecified cerebral artery		I63.30
Cerebral infarction due to thrombosis of middle cerebral artery		I63.31
Cerebral infarction due to thrombosis of right middle cerebral artery	434.01	I63.311
Thrombotic stroke involving right middle cerebral artery	434.01	I63.311
Cerebral infarction due to thrombosis of middle cerebral artery, right	434.01	I63.311
Thrombotic stroke involving middle cerebral artery, right	434.01	I63.311

© 2021 Eberly LA et al. *JAMA Network Open*.

Stroke due to thrombosis of right middle cerebral artery	434.01	I63.311
Cerebrovascular accident (CVA) due to thrombosis of right middle cerebral artery	434.01	I63.311
Cerebral infarction due to thrombosis of right middle cerebral artery		I63.311
Cerebral infarction due to thrombosis of left middle cerebral artery	434.01	I63.312
Thrombotic stroke involving left middle cerebral artery	434.01	I63.312
Cerebral infarction due to thrombosis of middle cerebral artery, left	434.01	I63.312
Thrombotic stroke involving middle cerebral artery, left	434.01	I63.312
Stroke due to thrombosis of left middle cerebral artery	434.01	I63.312
Cerebrovascular accident (CVA) due to thrombosis of left middle cerebral artery	434.01	I63.312
Cerebral infarction due to thrombosis of left middle cerebral artery		I63.312
Cerebral infarction due to bilateral thrombosis of middle cerebral arteries	434.01	I63.313
Cerebrovascular accident (CVA) due to bilateral thrombosis of middle cerebral arteries	434.01	I63.313
Cerebral infarction due to thrombosis of bilateral middle cerebral arteries	434.01	I63.313
Cerebral infarction due to thrombosis of bilateral middle cerebral arteries		I63.313
Cerebral infarction due to thrombosis of middle cerebral artery	434.01	I63.319
Thrombotic stroke involving middle cerebral artery	434.01	I63.319
Thrombotic stroke involving middle cerebral artery, unspecified laterality	434.01	I63.319

© 2021 Eberly LA et al. *JAMA Network Open*.

Cerebral infarction due to thrombosis of middle cerebral artery, unspecified laterality	434.01	I63.319
Cerebrovascular accident (CVA) due to thrombosis of middle cerebral artery	434.01	I63.319
Stroke due to thrombosis of middle cerebral artery	434.01	I63.319
Cerebral infarction due to thrombosis of unspecified middle cerebral artery	434.01	I63.319
Cerebral infarction due to thrombosis of middle cerebral artery, unspecified blood vessel laterality	434.01	I63.319
Cerebrovascular accident (CVA) due to thrombosis of middle cerebral artery, unspecified blood vessel laterality	434.01	I63.319
Cerebral infarction due to thrombus unspecified middle cerebral artery	434.01	I63.319
Cerebral infarction due to thrombosis of unspecified middle cerebral artery		I63.319
Cerebral infarction due to thrombosis of anterior cerebral artery		I63.32
Thrombotic stroke involving right anterior cerebral artery	434.01	I63.321
Cerebral infarction due to thrombosis of right anterior cerebral artery	434.01	I63.321
Thrombotic stroke involving anterior cerebral artery, right	434.01	I63.321
Cerebral infarction due to thrombosis of anterior cerebral artery, right	434.01	I63.321
Stroke due to thrombosis of right anterior cerebral artery	434.01	I63.321
Cerebrovascular accident (CVA) due to thrombosis of right anterior cerebral artery	434.01	I63.321
Cerebral infarction due to thrombus of right ant cerebral artery	434.01	I63.321
Cerebral infarction due to thrombosis of right anterior cerebral artery		I63.321

© 2021 Eberly LA et al. *JAMA Network Open*.

Cerebral infarction due to thrombosis of left anterior cerebral artery	434.01	I63.322
Thrombotic stroke involving left anterior cerebral artery	434.01	I63.322
Thrombotic stroke involving anterior cerebral artery, left	434.01	I63.322
Cerebral infarction due to thrombosis of anterior cerebral artery, left	434.01	I63.322
Cerebrovascular accident (CVA) due to thrombosis of left anterior cerebral artery	434.01	I63.322
Stroke due to thrombosis of left anterior cerebral artery	434.01	I63.322
Cerebral infarction due to thrombosis of left anterior cerebral artery		I63.322
Cerebral infarction due to bilateral thrombosis of anterior cerebral arteries	434.01	I63.323
Cerebrovascular accident (CVA) due to bilateral thrombosis of anterior cerebral arteries	434.01	I63.323
Cerebral infarction due to thrombosis of bilateral anterior arteries	434.01	I63.323
Cerebral infarction due to thrombosis of bilateral anterior cerebral arteries	434.01	I63.323
Cerebral infarction due to thrombosis of bilateral anterior cerebral arteries		I63.323
Cerebral infarction due to thrombosis of anterior cerebral artery	434.01	I63.329
Thrombotic stroke involving anterior cerebral artery	434.01	I63.329
Thrombotic stroke involving anterior cerebral artery, unspecified laterality	434.01	I63.329
Cerebral infarction due to thrombosis of anterior cerebral artery, unspecified laterality	434.01	I63.329
Stroke due to thrombosis of anterior cerebral artery	434.01	I63.329

© 2021 Eberly LA et al. *JAMA Network Open*.

Cerebrovascular accident (CVA) due to thrombosis of anterior cerebral artery	434.01	I63.329
Cerebral infarction due to thrombosis of unspecified anterior cerebral artery	434.01	I63.329
Cerebrovascular accident (CVA) due to thrombosis of anterior cerebral artery, unspecified blood vessel laterality	434.01	I63.329
Cerebral infarction due to thrombosis of anterior cerebral artery, unspecified blood vessel laterality	434.01	I63.329
Cerebral infarction due to thrombosis of unspecified anterior cerebral artery		I63.329
Cerebral infarction due to thrombosis of posterior cerebral artery		I63.33
Thrombotic stroke involving right posterior cerebral artery	434.01	I63.331
Cerebral infarction due to thrombosis of right posterior cerebral artery	434.01	I63.331
Cerebral infarction due to thrombosis of posterior cerebral artery, right	434.01	I63.331
Thrombotic stroke involving posterior cerebral artery, right	434.01	I63.331
Stroke due to thrombosis of right posterior cerebral artery	434.01	I63.331
Cerebrovascular accident (CVA) due to thrombosis of right posterior cerebral artery	434.01	I63.331
Cerebral infarction due to thrombus of right post cerebral artery	434.01	I63.331
Cerebral infarction due to thrombosis of right posterior cerebral artery		I63.331
Thrombotic stroke involving left posterior cerebral artery	434.01	I63.332
Cerebral infarction due to thrombosis of left posterior cerebral artery	434.01	I63.332
Thrombotic stroke involving posterior cerebral artery, left	434.01	I63.332

© 2021 Eberly LA et al. *JAMA Network Open*.

Cerebral infarction due to thrombosis of posterior cerebral artery, left	434.01	I63.332
Cerebrovascular accident (CVA) due to thrombosis of left posterior cerebral artery	434.01	I63.332
Stroke due to thrombosis of left posterior cerebral artery	434.01	I63.332
Cerebral infarction due to thrombus of left post cerebral artery	434.01	I63.332
Cerebral infarction due to thrombosis of left posterior cerebral artery		I63.332
Cerebral infarction due to bilateral thrombosis of posterior cerebral arteries	434.01	I63.333
Cerebrovascular accident (CVA) due to bilateral thrombosis of posterior cerebral arteries	434.01	I63.333
Cerebral infarction to thrombosis of bilateral posterior arteries	434.01	I63.333
Cerebral infarction to thrombosis of bilateral posterior cerebral arteries	434.01	I63.333
Cerebral infarction due to thrombosis of bilateral posterior cerebral arteries		I63.333
Cerebral infarction due to thrombosis of posterior cerebral artery	434.01	I63.339
Thrombotic stroke involving posterior cerebral artery	434.01	I63.339
Cerebral infarction due to thrombosis of posterior cerebral artery, unspecified laterality	434.01	I63.339
Thrombotic stroke involving posterior cerebral artery, unspecified laterality	434.01	I63.339
Stroke due to thrombosis of posterior cerebral artery	434.01	I63.339
Cerebrovascular accident (CVA) due to thrombosis of posterior cerebral artery	434.01	I63.339
Cerebral infarction due to thrombosis of unspecified posterior cerebral artery	434.01	I63.339

© 2021 Eberly LA et al. *JAMA Network Open*.

Cerebrovascular accident (CVA) due to thrombosis of posterior cerebral artery, unspecified blood vessel laterality	434.01	I63.339
Cerebral infarction due to thrombosis of posterior cerebral artery, unspecified blood vessel laterality	434.01	I63.339
Cerebral infarction due to thrombus unspecified posterior cerebral artery	434.01	I63.339
Cerebral infarction due to thrombosis of unspecified posterior cerebral artery		I63.339
Cerebral infarction due to thrombosis of cerebellar artery		I63.34
Cerebral infarction due to thrombosis of right cerebellar artery	434.01	I63.341
Thrombotic stroke involving right cerebellar artery	434.01	I63.341
Cerebral infarction due to thrombosis of cerebellar artery, right	434.01	I63.341
Thrombotic stroke involving cerebellar artery, right	434.01	I63.341
Cerebrovascular accident (CVA) due to thrombosis of right cerebellar artery	434.01	I63.341
Stroke due to thrombosis of right cerebellar artery	434.01	I63.341
Cerebral infarction due to thrombosis of right cerebellar artery		I63.341
Cerebral infarction due to thrombosis of left cerebellar artery	434.01	I63.342
Thrombotic stroke involving left cerebellar artery	434.01	I63.342
Cerebral infarction due to thrombosis of cerebellar artery, left	434.01	I63.342
Thrombotic stroke involving cerebellar artery, left	434.01	I63.342
Stroke due to thrombosis of left cerebellar artery	434.01	I63.342

© 2021 Eberly LA et al. *JAMA Network Open*.

Cerebrovascular accident (CVA) due to thrombosis of left cerebellar artery	434.01	I63.342
Cerebral infarction due to thrombosis of left cerebellar artery		I63.342
Cerebrovascular accident (CVA) due to bilateral thrombosis of cerebellar arteries	434.01	I63.343
Infarction of brain due to bilateral thrombosis of cerebellar arteries	434.01	I63.343
Cerebral infarction to thrombosis of bilateral cerebellar arteries	434.01	I63.343
Cerebral infarction due to thrombosis of bilateral cerebellar arteries		I63.343
Cerebral infarction due to thrombosis of cerebellar artery	434.01	I63.349
Thrombotic stroke involving cerebellar artery	434.01	I63.349
Cerebral infarction due to thrombosis of cerebellar artery, unspecified laterality	434.01	I63.349
Thrombotic stroke involving cerebellar artery, unspecified laterality	434.01	I63.349
Stroke due to thrombosis of cerebellar artery	434.01	I63.349
Cerebrovascular accident (CVA) due to thrombosis of cerebellar artery	434.01	I63.349
Cerebral infarction due to thrombosis of unspecified cerebellar artery	434.01	I63.349
Cerebral infarction due to thrombosis of cerebellar artery, unspecified blood vessel laterality	434.01	I63.349
Cerebrovascular accident (CVA) due to thrombosis of cerebellar artery, unspecified blood vessel laterality	434.01	I63.349
Cerebral infarction due to thrombosis of unspecified cerebellar artery		I63.349
Cerebral infarction due to thrombosis of other cerebral artery	434.01	I63.39

© 2021 Eberly LA et al. *JAMA Network Open*.

Cerebrovascular accident (CVA) due to thrombosis of other cerebral artery	434.01	I63.39
Cerebral infarction due to thrombosis of other cerebral artery		I63.39
Cerebral infarction due to embolism of cerebral artery	434.11	I63.40
Cerebral infarction due to unspecified occlusion or stenosis of cerebral arteries		I63.5
Unspecified cerebral artery occlusion with cerebral infarction	434.91	I63.50
Stroke-in-evolution syndrome	434.91	I63.50
Cerebral artery occlusion with cerebral infarction	434.91	I63.50
Encephalomalacia with cerebral infarction	434.91	I63.50
Right pontine cerebrovascular accident	434.91	I63.50
Right pontine stroke	434.91	I63.50
Right pontine CVA	434.91	I63.50
Cerebrovascular accident of right pontine structure	434.91	I63.50
Occlusion or stenosis of multiple cerebral arteries with cerebral infarction	434.91	I63.50
Cerebral infarction due to occlusion or stenosis of multiple cerebral arteries	434.91	I63.50
Cerebrovascular accident due to cerebral artery occlusion	434.91	I63.50
Cerebrovascular accident of left pontine structure	434.91	I63.50
Left pontine CVA	434.91	I63.50
Left pontine cerebrovascular accident	434.91	I63.50
Left pontine stroke	434.91	I63.50
Cerebral infarction due to cerebral artery occlusion	434.91	I63.50
Posterior circulation stroke	434.91	I63.50
Cerebrovascular accident involving posterior circulation	434.91	I63.50
Occlusion of intracranial artery with cerebral infarction	434.91	I63.50

© 2021 Eberly LA et al. *JAMA Network Open*.

Cerebral infarction due to stenosis of cerebral artery	434.91	I63.50
Cerebrovascular accident (CVA) due to occlusion of cerebral artery	434.91	I63.50
Cerebrovascular accident (CVA) due to stenosis of cerebral artery	434.91	I63.50
Stroke due to stenosis of cerebral artery	434.91	I63.50
Cerebrovascular accident (CVA) of left pontine structure	434.91	I63.50
Cerebrovascular accident (CVA) of right pontine structure	434.91	I63.50
Cerebrovascular accident (CVA) involving posterior circulation	434.91	I63.50
Cerebral infarction due to unspecified occlusion or stenosis of unspecified cerebral artery	434.91	I63.50
Cerebral infarction due to unspecified occlusion or stenosis of unspecified cerebral artery		I63.50
Cerebral infarction due to unspecified occlusion or stenosis of middle cerebral artery		I63.51
Arterial ischemic stroke, MCA (middle cerebral artery), right, acute	434.91	I63.511
Acute right arterial ischemic stroke, MCA (middle cerebral artery)	434.91	I63.511
Acute ischemic right MCA stroke	434.91	I63.511
Arterial ischemic stroke, MCA, right, acute	434.91	I63.511
Acute right MCA stroke	434.91	I63.511
Right middle cerebral artery stroke	434.91	I63.511
Cerebral infarct due to occlusion or stenosis middle cerebral artery, right	434.91	I63.511
Cerebral infarction due to occlusion or stenosis of middle cerebral artery, right	434.91	I63.511
Acute right arterial ischemic stroke, middle cerebral artery (MCA)	434.91	I63.511

© 2021 Eberly LA et al. *JAMA Network Open*.

Acute ischemic right middle cerebral artery (MCA) stroke	434.91	I63.511
Cerebral infarction due to occlusion of middle cerebral artery, right	434.91	I63.511
Cerebral infarction due to occlusion of right middle cerebral artery	434.91	I63.511
Cerebrovascular accident involving right middle cerebral artery territory	434.91	I63.511
Cerebral infarction involving right middle cerebral artery	434.01	I63.511
Cerebral infarction due to stenosis of right middle cerebral artery	434.91	I63.511
Cerebral infarction involving middle cerebral artery, right	434.01	I63.511
Cerebrovascular accident (CVA) due to stenosis of right middle cerebral artery	434.91	I63.511
Stroke due to occlusion of right middle cerebral artery	434.91	I63.511
Stroke due to stenosis of right middle cerebral artery	434.91	I63.511
Cerebrovascular accident (CVA) due to occlusion of right middle cerebral artery	434.91	I63.511
Cerebrovascular accident (CVA) involving right middle cerebral artery territory	434.91	I63.511
Cerebral infarction due to unspecified occlusion or stenosis of right middle cerebral artery	434.91	I63.511
Cerebral infarction due to unspecified occlusion or stenosis of right middle cerebral artery		I63.511
Arterial ischemic stroke, MCA (middle cerebral artery), left, acute	434.91	I63.512
Left acute arterial ischemic stroke, MCA (middle cerebral artery)	434.91	I63.512
Acute ischemic left MCA stroke	434.91	I63.512
Arterial ischemic stroke, MCA, left, acute	434.91	I63.512
Left middle cerebral artery stroke	434.91	I63.512

© 2021 Eberly LA et al. *JAMA Network Open*.

Cerebrovascular accident involving left middle cerebral artery territory	434.91	I63.512
Cerebral infarction due to occlusion or stenosis of middle cerebral artery, left	434.91	I63.512
Cerebral infarct due to occlusion or stenosis middle cerebral artery, left	434.91	I63.512
Acute ischemic left middle cerebral artery (MCA) stroke	434.91	I63.512
Cerebral infarction due to occlusion of middle cerebral artery, left	434.91	I63.512
Cerebral infarction due to occlusion of left middle cerebral artery	434.91	I63.512
Cerebral infarction involving left middle cerebral artery	434.01	I63.512
Cerebral infarction due to stenosis of left middle cerebral artery	434.91	I63.512
Cerebral infarction involving middle cerebral artery, left	434.01	I63.512
Cerebrovascular accident (CVA) due to occlusion of left middle cerebral artery	434.91	I63.512
Stroke due to stenosis of left middle cerebral artery	434.91	I63.512
Stroke due to occlusion of left middle cerebral artery	434.91	I63.512
Cerebrovascular accident (CVA) due to stenosis of left middle cerebral artery	434.91	I63.512
Cerebrovascular accident (CVA) involving left middle cerebral artery territory	434.91	I63.512
Cerebral infarction due to unspecified occlusion or stenosis of left middle cerebral artery	434.91	I63.512
Cerebral infarction d/t unspecified occlusion or stenos of left mid cerebral artery	434.91	I63.512
Cerebral infarction due to unspecified occlusion or stenosis of left middle cerebral artery		I63.512

© 2021 Eberly LA et al. *JAMA Network Open*.

Cerebral infarction due to bilateral stenosis of middle cerebral arteries	434.91	I63.513
Cerebral infarction due to bilateral occlusion of middle cerebral arteries	434.91	I63.513
Cerebrovascular accident (CVA) due to bilateral stenosis of middle cerebral arteries	434.91	I63.513
Cerebrovascular accident (CVA) due to bilateral occlusion of middle cerebral arteries	434.91	I63.513
Cerebral infarction due to unspecified occlusion or stenosis of bilateral middle arteries	434.91	I63.513
Cerebral infarction due to unspecified occlusion or stenosis of bilateral middle cerebral arteries	434.91	I63.513
Cerebral infarction due to unspecified occlusion or stenosis of bilateral middle cerebral arteries (CODE)	434.91	I63.513
Cerebral infarction due to unspecified occlusion or stenosis of bilateral middle cerebral arteries		I63.513
Cerebral infarct due to occlusion or stenosis middle cerebral artery	434.91	I63.519
Cerebral infarction due to occlusion or stenosis of middle cerebral artery	434.91	I63.519
Cerebral infarct due to occlusion or stenosis middle cerebral artery, unspecified laterality	434.91	I63.519
Cerebral infarction due to occlusion or stenosis of middle cerebral artery, unspecified laterality	434.91	I63.519
Cerebral infarction due to occlusion of middle cerebral artery	434.91	I63.519
Cerebral infarction due to occlusion of middle cerebral artery, unspecified laterality	434.91	I63.519

© 2021 Eberly LA et al. *JAMA Network Open*.

Cerebral infarction involving middle cerebral artery	434.01	I63.519
Cerebral infarction due to stenosis of middle cerebral artery	434.91	I63.519
Cerebral infarction involving middle cerebral artery, unspecified laterality	434.01	I63.519
Cerebrovascular accident (CVA) due to occlusion of middle cerebral artery	434.91	I63.519
Stroke due to stenosis of middle cerebral artery	434.91	I63.519
Stroke due to occlusion of middle cerebral artery	434.91	I63.519
Cerebrovascular accident (CVA) due to stenosis of middle cerebral artery	434.91	I63.519
Cerebral infarction due to unspecified occlusion or stenosis of unspecified middle cerebral artery	434.91	I63.519
Cerebrovascular accident (CVA) due to stenosis of middle cerebral artery, unspecified blood vessel laterality	434.91	I63.519
Cerebral infarction due to stenosis of middle cerebral artery, unspecified blood vessel laterality	434.91	I63.519
Cerebral infarction due to occlusion of middle cerebral artery, unspecified blood vessel laterality	434.91	I63.519
Cerebrovascular accident (CVA) due to occlusion of middle cerebral artery, unspecified blood vessel laterality	434.91	I63.519
Cerebral infarction due to unspecified occlusion or stenosis of unspecified middle cerebral artery		I63.519
Cerebral infarction due to unspecified occlusion or stenosis of anterior cerebral artery		I63.52
Arterial ischemic stroke, ACA (anterior cerebral artery), right, acute	434.91	I63.521

© 2021 Eberly LA et al. *JAMA Network Open*.

Acute right arterial ischemic stroke, ACA (anterior cerebral artery)	434.91	I63.521
Acute ischemic right ACA stroke	434.91	I63.521
Arterial ischemic stroke, ACA, right, acute	434.91	I63.521
Acute right ACA stroke	434.91	I63.521
Cerebral infarction due to occlusion or stenosis of anterior cerebral artery, right	434.91	I63.521
Acute ischemic multifocal anterior circulation stroke, right	434.91	I63.521
Acute ischemia multifocal anterior circulation stroke, right	434.91	I63.521
Arterial ischemic stroke, multifocal, anterior circulation, acute, right	434.91	I63.521
Acute arterial ischemic stroke, multifocal, anterior circulation, right	434.91	I63.521
Acute right arterial ischemic stroke, anterior cerebral artery (ACA)	434.91	I63.521
Acute ischemic right anterior cerebral artery (ACA) stroke	434.91	I63.521
Anterior cerebral circulation infarction, right	434.91	I63.521
Cerebral infarction due to anterior cerebral artery occlusion, right	434.91	I63.521
Cerebrovascular accident involving anterior circulation, right	434.91	I63.521
Total anterior cerebral circulation infarction, right	434.91	I63.521
Anterior circulation stroke, right	434.91	I63.521
Partial anterior cerebral circulation infarction, right	434.91	I63.521
Occlusion of recurrent artery of Huebner with cerebral infarction, right	434.91	I63.521
Cerebral infarction due to occlusion of right anterior cerebral artery	434.91	I63.521
Occlusion of right recurrent artery of Huebner with cerebral infarction	434.91	I63.521

© 2021 Eberly LA et al. *JAMA Network Open*.

Cerebrovascular accident involving anterior circulation of right side	434.91	I63.521
Total anterior cerebral circulation infarction of right side	434.91	I63.521
Anterior cerebral circulation infarction involving right-sided vessel	434.91	I63.521
Right-sided partial anterior cerebral circulation infarction	434.91	I63.521
Acute ischemic multifocal anterior circulation stroke involving right-sided vessel	434.91	I63.521
Cerebral infarction involving right anterior cerebral artery	434.91	I63.521
Cerebral infarction due to stenosis of right anterior cerebral artery	434.91	I63.521
Cerebral infarction involving anterior cerebral artery, right	434.91	I63.521
Cerebrovascular accident (CVA) due to occlusion of right anterior cerebral artery	434.91	I63.521
Stroke due to occlusion of right anterior cerebral artery	434.91	I63.521
Cerebrovascular accident (CVA) due to stenosis of right anterior cerebral artery	434.91	I63.521
Stroke due to stenosis of right anterior cerebral artery	434.91	I63.521
Cerebrovascular accident (CVA) involving anterior circulation of right side	434.91	I63.521
Cerebral infarction due to unspecified occlusion or stenosis of right anterior cerebral artery	434.91	I63.521
Right-sided anterior cerebral circulation infarction	434.91	I63.521
Acute ischemic multifocal right-sided anterior circulation stroke	434.91	I63.521
Right-sided total anterior cerebral circulation infarction	434.91	I63.521

© 2021 Eberly LA et al. *JAMA Network Open*.

Cerebral infarction d/t unspecified occlusion or stenos of right anterior cerebral artery	434.91	I63.521
Cerebral infarction due to unspecified occlusion or stenosis of right anterior cerebral artery		I63.521
Arterial ischemic stroke, ACA (anterior cerebral artery), left, acute	434.91	I63.522
Acute left arterial ischemic stroke, ACA (anterior cerebral artery)	434.91	I63.522
Acute ischemic left ACA stroke	434.91	I63.522
Arterial ischemic stroke, ACA, left, acute	434.91	I63.522
Acute left ACA ischemic stroke	434.91	I63.522
Acute ischemic multifocal anterior circulation stroke, left	434.91	I63.522
Arterial ischemic stroke, multifocal, anterior circulation, acute, left	434.91	I63.522
Acute arterial ischemic stroke, multifocal, anterior circulation, left	434.91	I63.522
Cerebral infarction due to occlusion or stenosis of anterior cerebral artery, left	434.91	I63.522
Acute ischemic left anterior cerebral artery (ACA) stroke	434.91	I63.522
Total anterior cerebral circulation infarction, left	434.91	I63.522
Partial anterior cerebral circulation infarction, left	434.91	I63.522
Cerebral infarction due to anterior cerebral artery occlusion, left	434.91	I63.522
Anterior circulation stroke, left	434.91	I63.522
Anterior cerebral circulation infarction, left	434.91	I63.522
Cerebrovascular accident involving anterior circulation, left	434.91	I63.522
Occlusion of recurrent artery of Huebner with cerebral infarction, left	434.91	I63.522
Cerebral infarction due to occlusion of left anterior cerebral artery	434.91	I63.522

© 2021 Eberly LA et al. *JAMA Network Open*.

Occlusion of left recurrent artery of Huebner with cerebral infarction	434.91	I63.522
Left-sided partial anterior cerebral circulation infarction	434.91	I63.522
Total anterior cerebral circulation infarction of left side	434.91	I63.522
Cerebrovascular accident involving anterior circulation of left side	434.91	I63.522
Acute ischemic multifocal anterior circulation stroke involving left-sided vessel	434.91	I63.522
Anterior cerebral circulation infarction involving left-sided vessel	434.91	I63.522
Cerebral infarction involving left anterior cerebral artery	434.91	I63.522
Cerebral infarction due to stenosis of left anterior cerebral artery	434.91	I63.522
Cerebral infarction involving anterior cerebral artery, left	434.91	I63.522
Cerebrovascular accident (CVA) due to stenosis of left anterior cerebral artery	434.91	I63.522
Stroke due to stenosis of left anterior cerebral artery	434.91	I63.522
Cerebrovascular accident (CVA) due to occlusion of left anterior cerebral artery	434.91	I63.522
Stroke due to occlusion of left anterior cerebral artery	434.91	I63.522
Cerebrovascular accident (CVA) involving anterior circulation of left side	434.91	I63.522
Cerebral infarction due to unspecified occlusion or stenosis of left anterior cerebral artery	434.91	I63.522
Acute ischemic multifocal left-sided anterior circulation stroke	434.91	I63.522
Left-sided total anterior cerebral circulation infarction	434.91	I63.522
Left-sided anterior cerebral circulation infarction	434.91	I63.522

© 2021 Eberly LA et al. *JAMA Network Open*.

Cerebral infarction d/t unspecified occlusion or stenos of left anterior cerebral artery	434.91	I63.522
Cerebral infarction due to unspecified occlusion or stenosis of left anterior cerebral artery		I63.522
Cerebral infarction due to bilateral occlusion of anterior cerebral arteries	434.91	I63.523
Cerebral infarction due to bilateral stenosis of anterior cerebral arteries	434.91	I63.523
Cerebrovascular accident (CVA) due to bilateral stenosis of anterior cerebral arteries	434.91	I63.523
Cerebral infarction due to unspecified occlusion or stenosis of bilateral anterior arteries	434.91	I63.523
Cerebral infarction due to unspecified occlusion or stenosis of bilateral anterior cerebral arteries	434.91	I63.523
Cerebral infarction due to unspecified occlusion or stenosis of bilateral anterior cerebral arteries (CODE)	434.91	I63.523
Cerebral infarction due to unspecified occlusion or stenosis of bilateral anterior cerebral arteries		I63.523
Arterial ischemic stroke, multifocal, anterior circulation, acute	434.91	I63.529
Acute arterial ischemic stroke, multifocal, anterior circulation	434.91	I63.529
Acute ischemic multifocal anterior circulation stroke	434.91	I63.529
Acute ischemic multifocal anterior circ stroke	434.91	I63.529
Cerebral infarct due to occlusion or stenosis anterior cerebral artery	434.91	I63.529
Cerebral infarction due to occlusion or stenosis of anterior cerebral artery	434.91	I63.529

© 2021 Eberly LA et al. *JAMA Network Open*.

Acute ischemic multifocal anterior circ stroke, unspecified laterality	434.91	I63.529
Arterial ischemic stroke, multifocal, anterior circulation, acute, unspecified laterality	434.91	I63.529
Acute ischemic multifocal anterior circulation stroke, unspecified laterality	434.91	I63.529
Cerebral infarction due to occlusion or stenosis of anterior cerebral artery, unspecified laterality	434.91	I63.529
Acute arterial ischemic stroke, multifocal, anterior circulation, unspecified laterality	434.91	I63.529
Partial anterior cerebral circulation infarction	434.91	I63.529
Anterior cerebral circulation infarction	434.91	I63.529
Total anterior cerebral circulation infarction	434.91	I63.529
Cerebral infarction due to anterior cerebral artery occlusion	434.91	I63.529
Anterior circulation stroke	434.91	I63.529
Cerebrovascular accident involving anterior circulation	434.91	I63.529
Cerebrovascular accident involving anterior circulation, unspecified laterality	IMO0001	I63.529
Partial anterior cerebral circulation infarction, unspecified laterality	434.91	I63.529
Total anterior cerebral circulation infarction, unspecified laterality	434.91	I63.529
Cerebral infarction due to anterior cerebral artery occlusion, unspecified laterality	434.91	I63.529
Anterior cerebral circulation infarction, unspecified laterality	434.91	I63.529
Anterior circulation stroke, unspecified laterality	IMO0001	I63.529
Occlusion of recurrent artery of Huebner with cerebral infarction	434.91	I63.529

© 2021 Eberly LA et al. *JAMA Network Open*.

Occlusion of recurrent artery of Huebner with cerebral infarction, unspecified laterality	434.91	I63.529
Anterior circulation stroke of uncertain pathology	434.91	I63.529
Cerebral infarction involving anterior cerebral artery	434.91	I63.529
Cerebrovascular accident of uncertain pathology involving anterior circulation	434.91	I63.529
Cerebral infarction due to stenosis of anterior cerebral artery	434.91	I63.529
Cerebral infarction involving anterior cerebral artery, unspecified laterality	434.91	I63.529
Stroke due to stenosis of anterior cerebral artery	434.91	I63.529
Cerebrovascular accident (CVA) due to stenosis of anterior cerebral artery	434.91	I63.529
Cerebrovascular accident (CVA) due to occlusion of anterior cerebral artery	434.91	I63.529
Stroke due to occlusion of anterior cerebral artery	434.91	I63.529
Cerebrovascular accident (CVA) involving anterior circulation	434.91	I63.529
Cerebrovascular accident (CVA) of uncertain pathology involving anterior circulation	434.91	I63.529
Cerebral infarction due to unspecified occlusion or stenosis of unspecified anterior cerebral artery	434.91	I63.529
Cerebrovascular accident (CVA) due to stenosis of anterior cerebral artery, unspecified blood vessel laterality	434.91	I63.529
Cerebral infarction due to stenosis of anterior cerebral artery, unspecified blood vessel laterality	434.91	I63.529

© 2021 Eberly LA et al. *JAMA Network Open*.

Cerebrovascular accident (CVA) due to occlusion of anterior cerebral artery, unspecified blood vessel laterality	434.91	I63.529
Cerebral infarction due to anterior cerebral artery occlusion, unspecified blood vessel laterality	434.91	I63.529
Cerebral infarction d/t unspecified occlusion or stenos of unspecified anterior cerebral art	434.91	I63.529
Cerebral infarction due to unspecified occlusion or stenosis of unspecified anterior cerebral artery		I63.529
Cerebral infarction due to unspecified occlusion or stenosis of posterior cerebral artery		I63.53
Acute right arterial ischemic stroke, PCA (posterior cerebral artery)	434.91	I63.531
Acute ischemic right PCA stroke	434.91	I63.531
Arterial ischemic stroke, PCA, right, acute	434.91	I63.531
Acute right PCA stroke	434.91	I63.531
Arterial ischemic stroke, PCA (posterior cerebral artery), right, acute	434.91	I63.531
Acute arterial ischemic stroke, multifocal, posterior circulation, right	434.91	I63.531
Arterial ischemic stroke, multifocal, posterior circulation, acute, right	434.91	I63.531
Acute ischemic multifocal posterior circulation stroke, right	434.91	I63.531
Acute ischemic right posterior cerebral artery (PCA) stroke	434.91	I63.531
Cerebral infarction due to posterior cerebral artery occlusion, right	434.91	I63.531
Posterior cerebral circulation hemorrhagic infarction, right	434.91	I63.531
Cerebral infarction due to occlusion of right posterior cerebral artery	434.91	I63.531
Hemorrhagic infarction involving posterior cerebral circulation of right side	434.91	I63.531

© 2021 Eberly LA et al. *JAMA Network Open*.

Acute ischemic multifocal posterior circulation stroke involving right-sided vessel	434.91	I63.531
Cerebral infarction involving right posterior cerebral artery	434.91	I63.531
Cerebral infarction due to stenosis of right posterior cerebral artery	434.91	I63.531
Cerebral infarction involving posterior cerebral artery, right	434.91	I63.531
Cerebrovascular accident (CVA) due to occlusion of right posterior cerebral artery	434.91	I63.531
Stroke due to stenosis of right posterior cerebral artery	434.91	I63.531
Cerebrovascular accident (CVA) due to stenosis of right posterior cerebral artery	434.91	I63.531
Stroke due to occlusion of right posterior cerebral artery	434.91	I63.531
Cerebral infarction due to unspecified occlusion or stenosis of right posterior cerebral artery	434.91	I63.531
Acute ischemic multifocal right-sided posterior circulation stroke	434.91	I63.531
Right-sided hemorrhagic posterior cerebral circulation infarction	434.91	I63.531
Cerebral infarction due to unspecified occlusion or stenosis of right posterior cerebral artery		I63.531
Arterial ischemic stroke, PCA (posterior cerebral artery), left, acute	434.91	I63.532
Left acute arterial ischemic stroke, PCA (posterior cerebral artery)	434.91	I63.532
Acute ischemic left PCA stroke	434.91	I63.532
Arterial ischemic stroke, PCA, left, acute	434.91	I63.532
Acute left PCA stroke	434.91	I63.532
Acute ischemic left posterior cerebral artery stroke	434.91	I63.532

© 2021 Eberly LA et al. *JAMA Network Open*.

Acute ischemic left posterior cerebral artery (PCA) stroke	434.91	I63.532
Acute ischemic multifocal posterior circulation stroke, left	434.91	I63.532
Arterial ischemic stroke, multifocal, posterior circulation, acute, left	434.91	I63.532
Acute arterial ischemic stroke, multifocal, posterior circulation, left	434.91	I63.532
Cerebral infarction due to posterior cerebral artery occlusion, left	434.91	I63.532
Posterior cerebral circulation hemorrhagic infarction, left	434.91	I63.532
Cerebral infarction due to occlusion of left posterior cerebral artery	434.91	I63.532
Hemorrhagic infarction involving posterior cerebral circulation of left side	434.91	I63.532
Acute ischemic multifocal posterior circulation stroke involving left-sided vessel	434.91	I63.532
Cerebral infarction involving left posterior cerebral artery	434.91	I63.532
Cerebral infarction due to stenosis of left posterior cerebral artery	434.91	I63.532
Cerebral infarction involving posterior cerebral artery, left	434.91	I63.532
Cerebrovascular accident (CVA) due to occlusion of left posterior cerebral artery	434.91	I63.532
Stroke due to occlusion of left posterior cerebral artery	434.91	I63.532
Cerebrovascular accident (CVA) due to stenosis of left posterior cerebral artery	434.91	I63.532
Stroke due to stenosis of left posterior cerebral artery	434.91	I63.532
Cerebral infarction due to unspecified occlusion or stenosis of left posterior cerebral artery	434.91	I63.532
Acute ischemic multifocal left-sided posterior circulation stroke	434.91	I63.532

© 2021 Eberly LA et al. *JAMA Network Open*.

Left-sided hemorrhagic posterior cerebral circulation infarction	434.91	I63.532
Cerebral infarction due to unspecified occlusion or stenosis of left posterior cerebral artery		I63.532
Cerebral infarction due to bilateral occlusion of posterior cerebral arteries	434.91	I63.533
Cerebral infarction due to bilateral stenosis of posterior cerebral arteries	434.91	I63.533
Cerebrovascular accident (CVA) due to bilateral stenosis of posterior cerebral arteries	434.91	I63.533
Cerebrovascular accident (CVA) due to bilateral occlusion of posterior cerebral arteries	434.91	I63.533
Cerebral infarction due to unspecified occlusion or stenosis of bilateral posterior arteries	434.91	I63.533
Cerebral infarction due to unspecified occlusion or stenosis of bilateral posterior cerebral arteries	434.91	I63.533
Cerebral infarction due to unspecified occlusion or stenosis of bilateral posterior cerebral arteries (CODE)	434.91	I63.533
Cerebral infarction due to unspecified occlusion or stenosis of bilateral posterior cerebral arteries		I63.533
Posterior circulation stroke of uncertain pathology	434.91	I63.539
Cerebrovascular accident of uncertain pathology involving posterior circulation	434.91	I63.539
Cerebrovascular accident (CVA) of uncertain pathology involving posterior circulation	434.91	I63.539
Arterial ischemic stroke, multifocal, posterior circulation, acute	434.91	I63.539

© 2021 Eberly LA et al. *JAMA Network Open*.

Acute arterial ischemic stroke, multifocal, posterior circulation	434.91	I63.539
Acute ischemic multifocal posterior circulation stroke	434.91	I63.539
Occlusion or stenosis of posterior cerebral artery with infarction	434.91	I63.539
Arterial ischemic stroke, multifocal, posterior circulation, acute, unspecified laterality	434.91	I63.539
Acute arterial ischemic stroke, multifocal, posterior circulation, unspecified laterality	434.91	I63.539
Acute ischemic multifocal posterior circulation stroke, unspecified laterality	434.91	I63.539
Posterior cerebral circulation hemorrhagic infarction	434.91	I63.539
Cerebral infarction due to posterior cerebral artery occlusion	434.91	I63.539
Cerebral infarction due to posterior cerebral artery occlusion, unspecified laterality	434.91	I63.539
Posterior cerebral circulation hemorrhagic infarction, unspecified laterality	434.91	I63.539
Cerebral infarction involving posterior cerebral artery	434.91	I63.539
Cerebral infarction due to stenosis of posterior cerebral artery	434.91	I63.539
Cerebral infarction involving posterior cerebral artery, unspecified laterality	434.91	I63.539
Cerebrovascular accident (CVA) due to occlusion of posterior cerebral artery	434.91	I63.539
Stroke due to occlusion of posterior cerebral artery	434.91	I63.539
Stroke due to stenosis of posterior cerebral artery	434.91	I63.539
Cerebrovascular accident (CVA) due to stenosis of posterior cerebral artery	434.91	I63.539

© 2021 Eberly LA et al. *JAMA Network Open*.

Cerebral infarction due to unspecified occlusion or stenosis of unspecified posterior cerebral artery	434.91	I63.539
Cerebral infarction due to stenosis of posterior cerebral artery, unspecified blood vessel laterality	434.91	I63.539
Cerebrovascular accident (CVA) due to occlusion of posterior cerebral artery, unspecified blood vessel laterality	434.91	I63.539
Cerebrovascular accident (CVA) due to stenosis of posterior cerebral artery, unspecified blood vessel laterality	434.91	I63.539
Cerebral infarction due to posterior cerebral artery occlusion, unspecified blood vessel laterality	434.91	I63.539
Cerebral infarction due to unspecified occlusion or stenosis of unspecified posterior cerebral artery		I63.539
Cerebral infarction due to unspecified occlusion or stenosis of cerebellar artery		I63.54
Cerebral infarction involving right cerebellar artery	434.01	I63.541
Cerebral infarction involving cerebellar artery, right	434.01	I63.541
Occlusion of posterior inferior cerebellar artery with cerebral infarction, right	433.81	I63.541
Occlusion of anterior inferior cerebellar artery with cerebral infarction, right	433.81	I63.541
Occlusion of right posterior inferior cerebellar artery with infarction	433.81	I63.541
Occlusion of right anterior inferior cerebellar artery with infarction	433.81	I63.541
Cerebral infarction due to occlusion of right cerebellar artery	434.91	I63.541
Cerebral infarction due to stenosis of right cerebellar artery	434.91	I63.541

© 2021 Eberly LA et al. *JAMA Network Open*.

Occlusion of posterior inferior cerebellar artery with infarction, right	433.81	I63.541
Occlusion of anterior inferior cerebellar artery with infarction, right	433.81	I63.541
Stroke in pediatric patient due to aneurysm, right	434.91	I63.541
Stroke due to occlusion of right cerebellar artery	434.91	I63.541
Cerebrovascular accident (CVA) due to occlusion of right cerebellar artery	434.91	I63.541
Stroke due to stenosis of right cerebellar artery	434.91	I63.541
Cerebrovascular accident (CVA) due to stenosis of right cerebellar artery	434.91	I63.541
Cerebral infarction due to unspecified occlusion or stenosis of right cerebellar artery	434.91	I63.541
Cerebral infarction due to unspecified occlusion or stenos of right cerebral art	434.91	I63.541
Cerebral infarction due to unspecified occlusion or stenosis of right cerebellar artery		I63.541
Cerebral infarction involving left cerebellar artery	434.01	I63.542
Cerebral infarction involving cerebellar artery, left	434.01	I63.542
Stroke in child due to aneurysm, left	434.91	I63.542
Occlusion of posterior inferior cerebellar artery with cerebral infarction, left	433.81	I63.542
Occlusion of anterior inferior cerebellar artery with cerebral infarction, left	433.81	I63.542
Occlusion of left posterior inferior cerebellar artery with infarction	433.81	I63.542
Occlusion of left anterior inferior cerebellar artery with infarction	433.81	I63.542

© 2021 Eberly LA et al. *JAMA Network Open*.

Cerebral infarction due to occlusion of left cerebellar artery	434.91	I63.542
Cerebral infarction due to stenosis of left cerebellar artery	434.91	I63.542
Occlusion of posterior inferior cerebellar artery with infarction, left	433.81	I63.542
Occlusion of anterior inferior cerebellar artery with infarction, left	433.81	I63.542
Stroke in pediatric patient due to aneurysm, left	434.91	I63.542
Cerebrovascular accident (CVA) due to occlusion of left cerebellar artery	434.91	I63.542
Cerebrovascular accident (CVA) due to stenosis of left cerebellar artery	434.91	I63.542
Stroke due to occlusion of left cerebellar artery	434.91	I63.542
Stroke due to stenosis of left cerebellar artery	434.91	I63.542
Cerebral infarction due to unspecified occlusion or stenosis of left cerebellar artery	434.91	I63.542
Cerebral infarction due to unspecified occlusion or stenosis of left cerebellar artery		I63.542
Cerebrovascular accident (CVA) due to bilateral occlusion of cerebellar arteries	434.91	I63.543
Cerebrovascular accident (CVA) due to bilateral stenosis of cerebellar arteries	434.91	I63.543
Infarction of brain due to bilateral occlusion of cerebellar arteries	434.91	I63.543
Infarction of brain due to bilateral stenosis of cerebellar arteries	434.91	I63.543
Cerebral infarction due to unspecified occlusion or stenosis of bilateral cerebellar arteries	434.91	I63.543

© 2021 Eberly LA et al. *JAMA Network Open*.

Cerebral infarction due to unspecified occlusion or stenosis of bilateral cerebellar arteries		I63.543
Cerebellar infarction with occlusion or stenosis of cerebellar artery	434.91	I63.549
Cerebral infarction involving cerebellar artery	434.01	I63.549
Cerebral infarction involving cerebellar artery, unspecified laterality	434.01	I63.549
Stroke in child due to aneurysm, unspecified laterality	434.91	I63.549
Occlusion of posterior inferior cerebellar artery with cerebral infarction	433.81	I63.549
Occlusion of anterior inferior cerebellar artery with cerebral infarction	433.81	I63.549
Occlusion of posterior inferior cerebellar artery with cerebral infarction, unspecified laterality	433.81	I63.549
Occlusion of anterior inferior cerebellar artery with cerebral infarction, unspecified laterality	433.81	I63.549
Occlusion of posterior inferior cerebellar artery with infarction	433.81	I63.549
Occlusion of anterior inferior cerebellar artery with infarction	433.81	I63.549
Stroke in pediatric patient due to aneurysm	434.91	I63.549
Cerebral infarction due to occlusion of cerebellar artery	433.81	I63.549
Cerebral infarction due to stenosis of cerebellar artery	433.81	I63.549
Occlusion of posterior inferior cerebellar artery with infarction, unspecified laterality	433.81	I63.549
Occlusion of anterior inferior cerebellar artery with infarction, unspecified laterality	433.81	I63.549

© 2021 Eberly LA et al. *JAMA Network Open*.

Stroke in pediatric patient due to aneurysm, unspecified laterality	434.91	I63.549
Stroke due to occlusion of cerebellar artery	434.91	I63.549
Cerebrovascular accident (CVA) due to occlusion of cerebellar artery	434.91	I63.549
Stroke due to stenosis of cerebellar artery	434.91	I63.549
Cerebrovascular accident (CVA) due to stenosis of cerebellar artery	434.91	I63.549
Cerebral infarction due to unspecified occlusion or stenosis of unspecified cerebellar artery	433.81	I63.549
Cerebrovascular accident (CVA) due to occlusion of cerebellar artery, unspecified blood vessel laterality	434.91	I63.549
Cerebrovascular accident (CVA) due to stenosis of cerebellar artery, unspecified blood vessel laterality	434.91	I63.549
Cerebral infarction due to stenosis of cerebellar artery, unspecified blood vessel laterality	433.81	I63.549
Cerebral infarction due to occlusion of cerebellar artery, unspecified blood vessel laterality	433.81	I63.549
Cerebral infarction due to unspecified occlusion or stenosis of unspecified cerebellar artery		I63.549
Occlusion and stenosis of other specified precerebral artery with cerebral infarction	433.81	I63.59
Other precerebral occlusion w/ infarction	433.81	I63.59
Cerebral infarction due to stenosis of other cerebral artery	434.91	I63.59
Cerebral infarction due to occlusion of other cerebral artery	434.91	I63.59

© 2021 Eberly LA et al. *JAMA Network Open*.

Cerebral infarction due to unspecified occlusion or stenosis of other cerebral artery	434.91	I63.59
Cerebrovascular accident (CVA) due to occlusion of other cerebral artery	434.91	I63.59
Cerebrovascular accident (CVA) due to stenosis of other cerebral artery	434.91	I63.59
Cerebral infarction due to unspecified occlusion or stenosis of other cerebral artery		I63.59
Cerebral venous infarction, associated with CSVT, acute	434.01	I63.6
Acute cerebral venous infarction associated with CSVT	434.01	I63.6
Cerebral infarction due to cerebral venous thrombosis, nonpyogenic	434.01	I63.6
Acute cerebral venous infarction associated with cerebral sinovenous thrombosis	434.01	I63.6
Cerebral venous infarction, associated with cerebral sinovenous thrombosis, acute	434.01	I63.6
Cerebral infarction due to nonpyogenic cerebral venous thrombosis	434.01	I63.6
Cerebral venous thrombosis of cortical vein with infarction	434.01	I63.6
Stroke due to nonpyogenic cerebral venous thrombosis	434.91	I63.6
Cerebrovascular accident (CVA) due to nonpyogenic cerebral venous thrombosis	434.91	I63.6
Cerebral infarction due to cerebral venous thrombosis, nonpyogenic		I63.6
Other cerebral infarction		I63.8
Lacunar infarction	434.91	I63.81
Lacunar stroke	434.91	I63.81
Stroke, lacunar	434.91	I63.81

© 2021 Eberly LA et al. *JAMA Network Open*.

Acute lacunar infarction	434.91	I63.81
Lacunar infarct, acute	434.91	I63.81
Left sided lacunar infarction	434.91	I63.81
Left sided lacunar stroke	434.91	I63.81
Pure sensorimotor lacunar infarction	434.91	I63.81
Multiple lacunar infarcts	434.91	I63.81
Right-sided lacunar stroke	434.91	I63.81
Right-sided lacunar infarction	434.91	I63.81
Left temporal lobe infarction	434.91	I63.89
Infarction of left temporal lobe	434.91	I63.89
Infarction of right temporal lobe	434.91	I63.89
Right temporal lobe infarction	434.91	I63.89
Brain stem infarction	434.91	I63.89
Brainstem infarction	434.91	I63.89
Acute arterial ischemic stroke, multifocal, multi vascular territories	434.91	I63.89
Acute idiopathic cerebral venous infarction	434.91	I63.89
Cerebral venous infarction, idiopathic, acute	434.91	I63.89
Acute bilateral cerebral infarction in a watershed distribution	434.91	I63.89
Cerebral infarction, watershed distribution, bilateral, acute	434.91	I63.89
Acute cerebral infarction associated with systemic hypoxia or ischemia	434.91	I63.89
Acute global diffuse cerebral infarction	434.91	I63.89
Cerebral infarction, global diffuse, acute	434.91	I63.89
Acute ischemic multifocal multiple vascular territories stroke	434.91	I63.89
Acute unilateral cerebral infarction in a watershed distribution	434.91	I63.89
Cerebral infarction, watershed distribution, unilateral, acute	434.91	I63.89
Cerebral venous infarction, acute	434.91	I63.89
Acute cerebral venous infarction	434.91	I63.89

© 2021 Eberly LA et al. *JAMA Network Open*.

Acute bilat watershed infarction	434.91	I63.89
Ac cerebral infarction w/ ischemia	434.91	I63.89
Acute global cerebral infarction	434.91	I63.89
Acute ischemic multi vascular territories stroke	434.91	I63.89
Acute brainstem infarction	434.91	I63.89
Brainstem infarct, acute	434.91	I63.89
Infarction of parietal lobe	434.91	I63.89
Parietal lobe infarction	434.91	I63.89
Arterial ischemic stroke, multifocal, multiple vascular territories, acute	434.91	I63.89
Acute arterial ischemic stroke, multifocal, multiple vascular territories	434.91	I63.89
Cerebral infarction, associated with systemic hypoxia or ischemia, acute	434.91	I63.89
Acute hemorrhagic infarction of brain	434.91	I63.89
Infarction of visual cortex	434.91	I63.89
Anterior cerebral circulation hemorrhagic infarction	431	I63.89
Cerebral infarction due to other mechanism	434.91	I63.89
Other cerebral infarction	434.91	I63.89
Cerebrovascular accident (CVA) due to other mechanism	434.91	I63.89
Right subthalamic lacunar stroke	434.91	I63.9
Lacunar stroke of right subthalamic region	434.91	I63.9
Lacunar stroke of left subthalamic region	434.91	I63.9
Left subthalamic lacunar stroke	434.91	I63.9
Impending cerebrovascular accident	435.9	I63.9
Progressing stroke	435.9	I63.9
RIND (reversible ischemic neurologic deficit) syndrome	434.91	I63.9
Reversible ischemic neurologic deficit syndrome	434.91	I63.9
Cerebellar infarction	434.91	I63.9

© 2021 Eberly LA et al. *JAMA Network Open*.

R.I.N.D. syndrome	434.91	I63.9
Anterior choroidal artery infarction	434.91	I63.9
Posterior choroidal artery infarction	434.91	I63.9
Mini stroke	434.91	I63.9
Brainstem stroke	434.91	I63.9
Basal ganglia infarction	434.91	I63.9
Brain attack	434.91	I63.9
Acute cerebrovascular accident	434.91	I63.9
Brain vascular accident	436	I63.9
Cerebral infarction	434.91	I63.9
Cerebral infarction, left hemisphere	434.91	I63.9
Cerebral vascular accident	434.91	I63.9
Cerebrovascular accident	434.91	I63.9
Cerebrovascular accident (stroke)	434.91	I63.9
CI (cerebral infarction)	434.91	I63.9
Completed stroke	434.91	I63.9
CVA (cerebral infarction)	434.91	I63.9
CVA (cerebral vascular accident)	434.91	I63.9
CVA (cerebrovascular accident)	434.91	I63.9
Nonparalytic stroke	434.91	I63.9
Paralytic stroke	434.91	I63.9
Right hemisphere, cerebral infarction	434.91	I63.9
Small vessel stroke	434.91	I63.9
Stroke	434.91	I63.9
Stroke (cerebrum)	434.91	I63.9
Stroke, paralytic	434.91	I63.9
Stroke/cerebrovascular accident	434.91	I63.9
Subcortical infarction	434.91	I63.9
Thalamic infarction	434.91	I63.9
Thrombotic stroke	434.01	I63.9
Right sided cerebral infarction	434.91	I63.9
Nonparalytic stroke syndrome	434.91	I63.9
Cerebral infarct	434.91	I63.9
Occipital cerebral infarction	434.91	I63.9
Occipital cortex infarction	434.91	I63.9
Occipital infarction	434.91	I63.9
Occipital stroke	434.91	I63.9

© 2021 Eberly LA et al. *JAMA Network Open*.

White matter periventricular infarction	434.91	I63.9
Cerebrovascular accident involving large vessel	434.91	I63.9
Cerebrovascular accident, large vessel	434.91	I63.9
Stroke, large vessel	434.91	I63.9
Large vessel stroke	434.91	I63.9
Congenital stroke	434.91	I63.9
Newborn stroke	434.91	I63.9
Arterial ischemic stroke	434.91	I63.9
Acute ischemic stroke	434.91	I63.9
Cerebral infarction, acute	434.91	I63.9
Acute cerebral infarction	434.91	I63.9
Chronic idiopathic cerebral venous infarction	434.91	I63.9
RIND syndrome	434.91	I63.9
RIND (reversible ischemic neurologic deficit)	434.91	I63.9
Stroke, thrombotic	434.01	I63.9
Cerebral venous infarction, chronic	434.91	I63.9
Chronic cerebral venous infarction	434.91	I63.9
Chronic global diffuse cerebral infarction	434.91	I63.9
Chronic cerebral infarction associated w/ with systemic hypoxia or ischemia	434.91	I63.9
Stroke, small vessel	434.91	I63.9
Cerebellar infarct	434.91	I63.9
Reversible ischemic neurologic deficit	434.91	I63.9
Cerebrovascular accident due to occlusion	434.91	I63.9
Cerebrovascular accident, impending	435.9	I63.9
Acute cerebrovascular accident of cerebellum	434.91	I63.9
Cerebellar stroke, acute	434.91	I63.9
Acute lacunar stroke	434.91	I63.9
Lacunar stroke, acute	434.91	I63.9

© 2021 Eberly LA et al. *JAMA Network Open*.

Acute thalamic infarction	434.91	I63.9
Thalamic infarct, acute	434.91	I63.9
New infarction of cerebellum	434.91	I63.9
New cerebellar infarct	434.91	I63.9
Acute occipital temporal infarction	434.91	I63.9
Occipitotemporal infarct, acute	434.91	I63.9
Pediatric stroke	434.91	I63.9
Pediatric cerebrovascular accident	434.91	I63.9
Stroke of unusual cause	434.91	I63.9
Reversible ischemic neurological deficit	434.91	I63.9
Stroke with cerebral ischemia	434.91	I63.9
Multiple cerebral infarctions	434.91	I63.9
Chronic cerebral infarction associated with systemic hypoxia or ischemia	434.91	I63.9
Cerebral infarction, associated with systemic hypoxia or ischemia, chronic	434.91	I63.9
Stroke-like episode	434.91	I63.9
Recent cerebral infarction in basilar artery distribution	434.91	I63.9
RIND (reversible ischemic neurologic deficit), acute	434.91	I63.9
Acute reversible ischemic neurologic deficit	434.91	I63.9
Left sided cerebral hemisphere cerebrovascular accident	436	I63.9
Right sided cerebral hemisphere cerebrovascular accident	434.91	I63.9
Non-hemorrhagic cerebrovascular accident	434.91	I63.9
Non-hemorrhagic stroke	434.91	I63.9
Cerebrovascular accident with involvement of both sides of body	434.91	I63.9
Cerebrovascular accident with involvement of right side of body	434.91	I63.9
Cerebrovascular accident with involvement of left side of body	434.91	I63.9

© 2021 Eberly LA et al. *JAMA Network Open*.

Cerebrovascular accident without paresis	434.91	I63.9
Cerebrovascular event	434.91	I63.9
Diagnosis of stroke during current admission	434.91	I63.9
Acute CVA (cerebrovascular accident)	434.91	I63.9
Diagnosed with stroke this admission	434.91	I63.9
Ischemic stroke	434.91	I63.9
Cerebellar stroke	434.91	I63.9
Signs of major cerebral infarct	434.91	I63.9
Ischemic stroke diagnosed during current admission	434.91	I63.9
Death due to stroke	434.91	I63.9
Cerebral infarction due to vascular occlusion	433.11	I63.9
Cerebral infarction due to vascular stenosis	433.11	I63.9
Cerebrovascular accident involving cerebellum	434.91	I63.9
Ischemic stroke without coma	434.91	I63.9
Idiopathic ischemic stroke in adult	434.91	I63.9
Cerebrovascular accident determined by clinical assessment	434.91	I63.9
Stroke determined by clinical assessment	434.91	I63.9
Idiopathic ischemic cerebrovascular accident in pediatric patient	434.91	I63.9
Idiopathic ischemic stroke occurring in pediatric patient	434.91	I63.9
Idiopathic ischemic cerebrovascular accident in adult	434.91	I63.9
Cerebral infarction due to unspecified mechanism	434.91	I63.9
Cerebrovascular accident (CVA)	434.91	I63.9
Focal infarction of brain	434.91	I63.9

© 2021 Eberly LA et al. *JAMA Network Open*.

Cerebrovascular accident (CVA) due to vascular stenosis	434.91	I63.9
Stroke due to vascular occlusion	434.91	I63.9
Cerebrovascular accident (CVA) due to vascular occlusion	434.91	I63.9
Stroke due to vascular stenosis	434.91	I63.9
Stroke due to thrombosis	434.01	I63.9
Cerebrovascular accident (CVA) due to thrombosis	434.01	I63.9
Cerebrovascular accident (CVA) with involvement of both sides of body	434.91	I63.9
Cerebrovascular accident (CVA) with involvement of left side of body	434.91	I63.9
Cerebrovascular accident (CVA) with involvement of right side of body	434.91	I63.9
Cerebrovascular accident (CVA) due to occlusion	434.91	I63.9
Acute cerebrovascular accident (CVA)	434.91	I63.9
Acute cerebrovascular accident (CVA) of cerebellum	434.91	I63.9
Impending cerebrovascular accident (CVA)	435.9	I63.9
Idiopathic ischemic cerebrovascular accident (CVA) in adult	434.91	I63.9
Cerebrovascular accident (CVA) without paresis	434.91	I63.9
Cerebrovascular accident (CVA) determined by clinical assessment	434.91	I63.9
Idiopathic ischemic cerebrovascular accident (CVA) in pediatric patient	434.91	I63.9
Cerebrovascular accident (CVA) involving large vessel	434.91	I63.9
Cerebrovascular accident (CVA) involving cerebellum	434.91	I63.9
Non-hemorrhagic cerebrovascular accident (CVA)	434.91	I63.9

© 2021 Eberly LA et al. *JAMA Network Open*.

Left sided cerebral hemisphere cerebrovascular accident (CVA)	436	I63.9
Right sided cerebral hemisphere cerebrovascular accident (CVA)	434.91	I63.9
Right-sided cerebrovascular accident (CVA)	434.91	I63.9
Left-sided cerebrovascular accident (CVA)	436	I63.9
Cerebrovascular accident (CVA) involving right cerebral hemisphere	434.91	I63.9
Cerebrovascular accident (CVA) involving left cerebral hemisphere	436	I63.9
Ischemic cerebrovascular accident (CVA) of frontal lobe	434.91	I63.9
Ischemic stroke of frontal lobe	434.91	I63.9
Cerebrovascular accident aborted by administration of thrombolytic agent	434.91	I63.9
Stroke aborted by administration of thrombolytic agent	434.91	I63.9
Cerebral infarction, unspecified	434.91	I63.9
Cerebrovascular accident (CVA), unspecified mechanism	434.91	I63.9
Cerebral infarction, unspecified mechanism	434.91	I63.9
Nonatherosclerotic cerebrovascular accident (CVA)	434.91	I63.9
Silent cerebral infarction	434.91	I63.9
Cerebral infarction, unspecified		I63.9
Transient ischemic attack (TIA), and cerebral infarction without residual deficits	434.91, 435.9	I63.9, G45.9
Transient ischemic attack (TIA), and cerebral infarction without residual deficits	434.91, 435.9	I63.9, G45.9
Flaccid hemiplegia due to infarction of brain	342.00, 434.91	I63.9, G81.00

© 2021 Eberly LA et al. *JAMA Network Open*.

Flaccid hemiplegia of right dominant side due to infarction of brain	342.01, 434.91	I63.9, G81.01
Flaccid hemiplegia of left dominant side due to infarction of brain	342.01, 434.91	I63.9, G81.02
Flaccid hemiplegia of right nondominant side due to infarction of brain	342.02, 434.91	I63.9, G81.03
Flaccid hemiplegia of left nondominant side due to infarction of brain	342.02, 434.91	I63.9, G81.04
Spastic hemiplegia due to infarction of brain	434.91, 342.10	I63.9, G81.10
Spastic hemiplegia of right dominant side due to infarction of brain	434.91, 342.11	I63.9, G81.11
Spastic hemiplegia of left dominant side due to infarction of brain	434.91, 342.11	I63.9, G81.12
Spastic hemiplegia of left nondominant side due to infarction of brain	434.91, 342.12	I63.9, G81.14
Hemiplegia due to infarction of brain	434.91, 342.90	I63.9, G81.90
Hemiparesis due to cerebral infarction, unspecified laterality	438.2	I63.9, G81.90
Hemiplegia of right dominant side due to infarction of brain	438.21, 429.79	I63.9, G81.91
Hemiplegia of left dominant side due to infarction of brain	438.21	I63.9, G81.92
Hemiplegia of right nondominant side due to infarction of brain	438.22, 429.79	I63.9, G81.93
Hemiplegia of left nondominant side due to infarction of brain	434.91, 438.22	I63.9, G81.94
Ischemic stroke with paralysis	434.91, 344.9	I63.9, G83.9
Presumed perinatal ischemic vertebrobasilar artery brainstem stroke	779.9	I63.9, P91.0
Arterial ischemic stroke, vertebrobasilar, brainstem, presumed perinatal	779.9	I63.9, P91.0
Ischemic stroke with coma	434.91, 780.01	I63.9, R40.20
Seizure with onset of stroke	434.91, 780.39	I63.9, R56.9

© 2021 Eberly LA et al. *JAMA Network Open*.

Contraindication for thrombolytic medication in stroke patient	434.91, V64.1	I63.9, Z53.09
Occlusion and stenosis of precerebral arteries, not resulting in cerebral infarction		I65
Occlusion and stenosis of vertebral artery		I65.0
Occlusion and stenosis of right vertebral artery	433.2	I65.01
Vertebral artery narrowing, right	433.2	I65.01
Thrombosis, arteries, vertebral, right	433.2	I65.01
Occlusion and stenosis of vertebral artery without mention of cerebral infarction, right	433.2	I65.01
Vertebrobasilar artery stenosis, right	433.3	I65.01
Vertebral artery thrombosis, right	433.2	I65.01
Vertebral artery stenosis, symptomatic, without infarction, right	433.2	I65.01
Symptomatic vertebral artery stenosis without infarction, right	433.2	I65.01
Vertebral artery obstruction, right	433.2	I65.01
Vertebral artery stenosis, right	433.2	I65.01
Asymptomatic vertebral artery stenosis, right	433.2	I65.01
Vertebral artery stenosis/occlusion, right	433.2	I65.01
Vertebral artery stenosis, asymptomatic, right	433.2	I65.01
Vertebral artery stenosis, non-symptomatic, right	433.2	I65.01
Occlusion and stenosis of vertebral artery, right	433.2	I65.01
Occlusion of vertebral artery, right	433.2	I65.01
Vertebral artery occlusion, right	433.2	I65.01

© 2021 Eberly LA et al. *JAMA Network Open*.

Stenosis of vertebral artery without cerebral infarction, right	433.2	I65.01
Occlusion of vertebral artery without cerebral infarction, right	433.2	I65.01
Symptomatic stenosis of right vertebral artery without infarction	433.2	I65.01
Thrombosis of right vertebral artery	433.2	I65.01
Occlusion of right vertebral artery	433.2	I65.01
Stenosis of right vertebral artery without cerebral infarction	433.2	I65.01
Stenosis of right vertebral artery	433.2	I65.01
Obstruction of right vertebral artery	433.2	I65.01
Stenosis of right vertebrobasilar artery	433.3	I65.01
Occlusion of right vertebral artery without cerebral infarction	433.2	I65.01
Asymptomatic stenosis of right vertebral artery	433.2	I65.01
Occlusion and stenosis of vertebral artery without cerebral infarction, right	433.2	I65.01
Occlusion or stenosis of right vertebral artery without cerebral infarction	433.2	I65.01
Occlusion and stenosis of right vertebral artery		I65.01
Occlusion and stenosis of left vertebral artery	433.2	I65.02
Vertebral artery stenosis, non-symptomatic, left	433.2	I65.02
Vertebrobasilar artery stenosis, left	433.3	I65.02
Vertebral artery stenosis, left	433.2	I65.02
Vertebral artery stenosis/occlusion, left	433.2	I65.02
Vertebral artery narrowing, left	433.2	I65.02
Vertebral artery stenosis, asymptomatic, left	433.2	I65.02
Vertebral artery stenosis, symptomatic, without infarction, left	433.2	I65.02

© 2021 Eberly LA et al. *JAMA Network Open*.

Occlusion and stenosis of vertebral artery without mention of cerebral infarction, left	433.2	I65.02
Vertebral artery obstruction, left	433.2	I65.02
Vertebral artery thrombosis, left	433.2	I65.02
Asymptomatic vertebral artery stenosis, left	433.2	I65.02
Occlusion and stenosis of vertebral artery, left	433.2	I65.02
Thrombosis, arteries, vertebral, left	433.2	I65.02
Symptomatic vertebral artery stenosis without infarction, left	433.2	I65.02
Vertebral artery occlusion, left	433.2	I65.02
Occlusion of vertebral artery, left	433.2	I65.02
Occlusion of vertebral artery without cerebral infarction, left	433.2	I65.02
Stenosis of vertebral artery without cerebral infarction, left	433.2	I65.02
Occlusion of left vertebral artery without cerebral infarction	433.2	I65.02
Obstruction of left vertebral artery	433.2	I65.02
Asymptomatic stenosis of left vertebral artery	433.2	I65.02
Stenosis of left vertebral artery without cerebral infarction	433.2	I65.02
Stenosis of left vertebral artery	433.2	I65.02
Thrombosis of left vertebral artery	433.2	I65.02
Stenosis of left vertebrobasilar artery	433.3	I65.02
Occlusion of left vertebral artery	433.2	I65.02
Symptomatic stenosis of left vertebral artery without infarction	433.2	I65.02
Occlusion and stenosis of vertebral artery without cerebral infarction, left	433.2	I65.02

© 2021 Eberly LA et al. *JAMA Network Open*.

Occlusion or stenosis of left vertebral artery without cerebral infarction	433.2	I65.02
Occlusion and stenosis of left vertebral artery		I65.02
Occlusion and stenosis of both vertebral arteries	433.2	I65.03
Vertebrobasilar artery stenosis, bilateral	433.3	I65.03
Vertebral artery stenosis, symptomatic, without infarction, bilateral	433.2	I65.03
Vertebral artery thrombosis, bilateral	433.2	I65.03
Vertebral artery obstruction, bilateral	433.2	I65.03
Vertebral artery stenosis, bilateral	433.2	I65.03
Asymptomatic vertebral artery stenosis, bilateral	433.2	I65.03
Vertebral artery stenosis, asymptomatic, bilateral	433.2	I65.03
Vertebral artery stenosis, non-symptomatic, bilateral	433.2	I65.03
Occlusion and stenosis of vertebral artery, bilateral	433.2	I65.03
Vertebral artery narrowing, bilateral	433.2	I65.03
Symptomatic vertebral artery stenosis without infarction, bilateral	433.2	I65.03
Occlusion and stenosis of vertebral artery without mention of cerebral infarction, bilateral	433.2	I65.03
Vertebral artery stenosis/occlusion, bilateral	433.2	I65.03
Thrombosis, arteries, vertebral, bilateral	433.2	I65.03
Vertebral artery occlusion, bilateral	433.2	I65.03
Occlusion of vertebral artery, bilateral	433.2	I65.03
Occlusion of vertebral artery without cerebral infarction, bilateral	433.2	I65.03

© 2021 Eberly LA et al. *JAMA Network Open*.

Stenosis of vertebral artery without cerebral infarction, bilateral	433.2	I65.03
Symptomatic stenosis of both vertebral arteries without infarction	433.2	I65.03
Stenosis of both vertebrobasilar arteries	433.3	I65.03
Thrombosis of both vertebral arteries	433.2	I65.03
Stenosis of both vertebral arteries	433.2	I65.03
Stenosis of both vertebral arteries without cerebral infarction	433.2	I65.03
Occlusion of both vertebral arteries	433.2	I65.03
Obstruction of both vertebral arteries	433.2	I65.03
Occlusion of both vertebral arteries without cerebral infarction	433.2	I65.03
Asymptomatic stenosis of both vertebral arteries	433.2	I65.03
Occlusion and stenosis of vertebral artery without cerebral infarction, bilateral	433.20, 433.30	I65.03
Occlusion and stenosis of bilateral vertebral arteries	433.2	I65.03
Occlusion or stenosis of both vertebral arteries without cerebral infarction	433.20, 433.30	I65.03
Occlusion and stenosis of bilateral vertebral arteries		I65.03
Occlusion and stenosis of vertebral artery without mention of cerebral infarction	433.2	I65.09
Vertebral artery stenosis	433.2	I65.09
Vertebral artery narrowing	433.2	I65.09
Vertebral artery obstruction	433.2	I65.09
Vertebral artery occlusion	433.2	I65.09
Vertebral artery thrombosis	433.2	I65.09
Occlusion and stenosis of vertebral artery	433.2	I65.09
Thrombosis, arteries, vertebral	433.2	I65.09
Vertebral artery stenosis/occlusion	433.2	I65.09
Asymptomatic vertebral artery stenosis	433.2	I65.09

© 2021 Eberly LA et al. *JAMA Network Open*.

Symptomatic vertebral artery stenosis without infarction	433.2	I65.09
Vertebral artery stenosis, symptomatic, without infarction	433.2	I65.09
Vertebral artery stenosis, asymptomatic	433.2	I65.09
Vertebral artery stenosis, non-symptomatic	433.2	I65.09
Asymptomatic vertebral artery stenosis, unspecified laterality	433.2	I65.09
Symptomatic vertebral artery stenosis without infarction, unspecified laterality	433.2	I65.09
Vertebral artery stenosis, asymptomatic, unspecified laterality	433.2	I65.09
Occlusion and stenosis of vertebral artery, unspecified laterality	433.2	I65.09
Vertebral artery thrombosis, unspecified laterality	433.2	I65.09
Thrombosis, arteries, vertebral, unspecified laterality	433.2	I65.09
Occlusion and stenosis of vertebral artery without mention of cerebral infarction, unspecified laterality	433.2	I65.09
Vertebral artery stenosis, non-symptomatic, unspecified laterality	433.2	I65.09
Vertebral artery stenosis, symptomatic, without infarction, unspecified laterality	433.2	I65.09
Vertebral artery narrowing, unspecified laterality	433.2	I65.09
Vertebral artery obstruction, unspecified laterality	433.2	I65.09
Vertebral artery stenosis, unspecified laterality	433.2	I65.09
Vertebral artery stenosis/occlusion, unspecified laterality	433.2	I65.09
Occlusion of vertebral artery	433.2	I65.09

© 2021 Eberly LA et al. *JAMA Network Open*.

Vertebrobasilar artery stenosis, unspecified laterality	433.3	I65.09
Occlusion of vertebral artery, unspecified laterality	433.2	I65.09
Vertebral artery occlusion, unspecified laterality	433.2	I65.09
Stenosis of vertebral artery without cerebral infarction	433.2	I65.09
Occlusion of vertebral artery without cerebral infarction	433.2	I65.09
Occlusion of vertebral artery without cerebral infarction, unspecified laterality	433.2	I65.09
Stenosis of vertebral artery without cerebral infarction, unspecified laterality	433.2	I65.09
Occlusion and stenosis of vertebral artery without cerebral infarction	433.2	I65.09
Anterior spinal artery syndrome associated with thrombosis	433.8	I65.09
Occlusion and stenosis of vertebral artery without cerebral infarction, unspecified laterality	433.2	I65.09
Vertebral art occ w/o infarct	433.2	I65.09
Occlusion and stenosis of unspecified vertebral artery	433.2	I65.09
Bow hunter's stroke	433.2	I65.09
Occlusion and stenosis of unspecified vertebral artery		I65.09
Occlusion and stenosis of basilar artery without mention of cerebral infarction	433	I65.1
Basilar artery stenosis	433	I65.1
Basilar artery narrowing	433	I65.1
Basilar artery obstruction	433	I65.1
Basilar artery occlusion	433	I65.1
Basilar artery thrombosis	433	I65.1

© 2021 Eberly LA et al. *JAMA Network Open*.

Occlusion and stenosis of basilar artery	433	I65.1
Basilar artery stenosis/occlusion	433	I65.1
Arteriosclerosis of basilar artery	433	I65.1
Symptomatic basilar artery stenosis without infarction	433	I65.1
Basilar artery stenosis, symptomatic, without infarction	433	I65.1
Asymptomatic basilar artery stenosis	433	I65.1
Basilar artery stenosis, asymptomatic	433	I65.1
Arteriosclerosis, basilar artery	433	I65.1
Basilar artery stenosis, non-symptomatic	433	I65.1
Occlusion and stenosis basilar artery w/o mention cerebral infarct	433	I65.1
Basilar artery occlusion without cerebral infarction	433	I65.1
Occlusion and stenosis of basilar artery without cerebral infarction	433	I65.1
Basilar artery occlusion w/o infarction	433	I65.1
Occlusion and stenosis of basilar artery		I65.1
Vertebrobasilar artery stenosis	433.3	I65.1, I65.09
Occlusion and stenosis of carotid artery		I65.2
Occlusion of right carotid artery	433.1	I65.21
Carotid occlusion, right	433.1	I65.21
Stenosis of right carotid artery	433.1	I65.21
Carotid stenosis, right	433.1	I65.21
Thrombosis of right carotid artery	433.1	I65.21
Carotid thrombosis, right	433.1	I65.21
Occlusion and stenosis of right carotid artery	433.1	I65.21
Right carotid artery occlusion	433.1	I65.21
Atherosclerosis of right carotid artery	433.1	I65.21
Right internal carotid occlusion	433.1	I65.21
Right cavernous carotid stenosis	433.1	I65.21
Stenosis of cavernous portion of right internal carotid artery	433.1	I65.21

© 2021 Eberly LA et al. *JAMA Network Open*.

Carotid artery stenosis, symptomatic, right	433.1	I65.21
Carotid artery thrombosis, right	433.1	I65.21
Carotid artery obstruction, right	433.1	I65.21
Carotid artery stenosis, asymptomatic, right	433.1	I65.21
Symptomatic carotid artery stenosis, right	433.1	I65.21
Unilateral carotid artery stenosis, right	433.1	I65.21
Common carotid artery stenosis, right	433.1	I65.21
Symptomatic carotid artery stenosis without infarction, right	433.1	I65.21
Carotid artery occlusion and stenosis, right	433.1	I65.21
Carotid atherosclerosis, right	433.1	I65.21
Carotid stenosis, symptomatic w/o infarct, right	433.1	I65.21
Recurrent carotid stenosis, right	433.1	I65.21
Carotid artery occlusion, right	433.1	I65.21
Carotid stenosis, asymptomatic, right	433.1	I65.21
Carotid stenosis, non-symptomatic, right	433.1	I65.21
Carotid artery stenosis, right	433.1	I65.21
Carotid ulcer, right	433.1	I65.21
Carotid artery stenosis, unilateral, right	433.1	I65.21
Neck artery obstruction, right	433.1	I65.21
Asymptomatic carotid artery narrowing without infarction, right	433.1	I65.21
Internal carotid artery occlusion, right	433.1	I65.21
Carotid artery calcification, right	433.1	I65.21
Asymptomatic carotid artery stenosis without infarction, right	433.1	I65.21
Internal carotid artery stenosis, right	433.1	I65.21
ICAO (internal carotid artery occlusion), right	433.1	I65.21

© 2021 Eberly LA et al. *JAMA Network Open*.

Occlusion and stenosis of carotid artery without mention of cerebral infarction, right	433.1	I65.21
Thrombosis of internal carotid, right	433.1	I65.21
Asymptomatic carotid artery stenosis, right	433.1	I65.21
Internal carotid artery thrombosis, right	433.1	I65.21
Common carotid artery thrombosis, right	433.1	I65.21
Carotid artery occlusion without infarction, right	433.1	I65.21
Occlusion of carotid artery, right	433.1	I65.21
Carotid artery stenosis and occlusion, right	433.1	I65.21
Carotid artery narrowing, right	433.1	I65.21
Carotid thromboses, right	433.1	I65.21
Carotid artery plaque, right	433.1	I65.21
External carotid artery stenosis, right	433.1	I65.21
External carotid artery thrombosis, right	433.1	I65.21
Thrombosis of external carotid, right	433.1	I65.21
Carotid artery, internal, occlusion, right	433.1	I65.21
Intracranial carotid stenosis, right	433.1	I65.21
Occlusion and stenosis of carotid artery, right	433.1	I65.21
Symptomatic carotid artery narrowing without infarction, right	433.1	I65.21
Stenosis of carotid artery, right	433.1	I65.21
Arteriosclerosis of carotid artery, right	433.1	I65.21
Carotid artery stenosis without cerebral infarction, right	433.1	I65.21
Obstruction of carotid artery, right	433.1	I65.21
Thrombosis of right common carotid artery	433.1	I65.21
Stenosis of right carotid artery without cerebral infarction	433.1	I65.21

© 2021 Eberly LA et al. *JAMA Network Open*.

Asymptomatic stenosis of right carotid artery	433.1	I65.21
Thrombosis of right external carotid artery	433.1	I65.21
Symptomatic stenosis of right carotid artery	433.1	I65.21
Asymptomatic stenosis of right carotid artery without infarction	433.1	I65.21
Stenosis of right internal carotid artery	433.1	I65.21
Obstruction of neck artery of right side	433.1	I65.21
Occlusion of right internal carotid artery	433.1	I65.21
Symptomatic stenosis of right carotid artery without infarction	433.1	I65.21
Arteriosclerosis of right carotid artery	433.1	I65.21
Thrombosis of right internal carotid artery	433.1	I65.21
Calcification of right carotid artery	433.1	I65.21
Recurrent stenosis of right carotid artery	433.1	I65.21
Stenosis of intracranial portions of right internal carotid artery	433.1	I65.21
Stenosis of right carotid artery without infarction	433.1	I65.21
Mild atherosclerosis of right carotid artery	433.1	I65.21
Obstruction of right carotid artery	433.1	I65.21
Obstruction of right carotid artery without cerebral infarction	433.1	I65.21
Right-sided carotid artery obstruction	433.1	I65.21
Right-sided carotid artery obstruction without cerebral infarction	433.1	I65.21
Right-sided extracranial carotid artery stenosis	433.1	I65.21
Right-sided carotid artery occlusion without cerebral infarction	433.1	I65.21
Right-sided extracranial carotid artery occlusion	433.1	I65.21

© 2021 Eberly LA et al. *JAMA Network Open*.

Mild atherosclerosis of carotid artery, right	433.1	I65.21
Obstruction of carotid artery without cerebral infarction, right	433.1	I65.21
Greater than 50 percent stenosis of carotid artery, right	433.1	I65.21
Stenosis of extracranial carotid artery, right	433.1	I65.21
Occlusion of carotid artery without cerebral infarction, right	433.1	I65.21
Occlusion of extracranial carotid artery, right	433.1	I65.21
Stenosis of right external carotid artery	433.1	I65.21
More than 50 percent stenosis of right internal carotid artery	433.1	I65.21
Stenosis of right carotid artery greater than 50%	433.1	I65.21
Occlusion and stenosis of right carotid artery		I65.21
Occlusion of left carotid artery	433.1	I65.22
Carotid occlusion, left	433.1	I65.22
Stenosis of left carotid artery	433.1	I65.22
Carotid stenosis, left	433.1	I65.22
Thrombosis of left carotid artery	433.1	I65.22
Carotid thrombosis, left	433.1	I65.22
Left carotid artery stenosis	433.1	I65.22
Left carotid stenosis	433.1	I65.22
Occlusion and stenosis of left carotid artery	433.1	I65.22
Left carotid artery occlusion	433.1	I65.22
Atherosclerosis of left carotid artery	433.1	I65.22
Unilateral carotid artery stenosis, left	433.1	I65.22
Occlusion and stenosis of carotid artery, left	433.1	I65.22
Asymptomatic carotid artery stenosis without infarction, left	433.1	I65.22

© 2021 Eberly LA et al. *JAMA Network Open*.

Thrombosis of external carotid, left	433.1	I65.22
Carotid artery thrombosis, left	433.1	I65.22
Carotid artery stenosis and occlusion, left	433.1	I65.22
Asymptomatic carotid artery narrowing without infarction, left	433.1	I65.22
Carotid artery narrowing, left	433.1	I65.22
Internal carotid artery stenosis, left	433.1	I65.22
Symptomatic carotid artery narrowing without infarction, left	433.1	I65.22
Carotid ulcer, left	433.1	I65.22
Carotid artery stenosis, unilateral, left	433.1	I65.22
Symptomatic carotid artery stenosis without infarction, left	433.1	I65.22
Carotid artery occlusion and stenosis, left	433.1	I65.22
Thrombosis of internal carotid, left	433.1	I65.22
Carotid atherosclerosis, left	433.1	I65.22
Carotid stenosis, asymptomatic, left	433.1	I65.22
Carotid artery calcification, left	433.1	I65.22
Carotid artery occlusion, left	433.1	I65.22
External carotid artery stenosis, left	433.1	I65.22
ICAO (internal carotid artery occlusion), left	433.1	I65.22
Carotid stenosis, symptomatic w/o infarct, left	433.1	I65.22
Internal carotid artery occlusion, left	433.1	I65.22
Neck artery obstruction, left	433.1	I65.22
Common carotid artery thrombosis, left	433.1	I65.22
Symptomatic carotid artery stenosis, left	433.1	I65.22
Carotid artery stenosis, symptomatic, left	433.1	I65.22
Carotid thromboses, left	433.1	I65.22
Stenosis of carotid artery, left	433.1	I65.22
Carotid artery narrowing, left	433.1	I65.22
Carotid artery stenosis, asymptomatic, left	433.1	I65.22

© 2021 Eberly LA et al. *JAMA Network Open*.

Carotid artery stenosis, left	433.1	I65.22
Intracranial carotid stenosis, left	433.1	I65.22
External carotid artery thrombosis, left	433.1	I65.22
Carotid artery, internal, occlusion, left	433.1	I65.22
Occlusion of carotid artery, left	433.1	I65.22
Common carotid artery stenosis, left	433.1	I65.22
Carotid artery obstruction, left	433.1	I65.22
Internal carotid artery thrombosis, left	433.1	I65.22
Recurrent carotid stenosis, left	433.1	I65.22
Carotid stenosis, non-symptomatic, left	433.1	I65.22
Carotid artery occlusion without infarction, left	433.1	I65.22
Carotid artery plaque, left	433.1	I65.22
Occlusion and stenosis of carotid artery without mention of cerebral infarction, left	433.1	I65.22
Asymptomatic carotid artery stenosis, left	433.1	I65.22
Arteriosclerosis of carotid artery, left	433.1	I65.22
Carotid artery stenosis without cerebral infarction, left	433.1	I65.22
Obstruction of carotid artery, left	433.1	I65.22
Thrombosis of left internal carotid artery	433.1	I65.22
Recurrent stenosis of left carotid artery	433.1	I65.22
Obstruction of neck artery of left side	433.1	I65.22
Calcification of left carotid artery	433.1	I65.22
Occlusion of left internal carotid artery	433.1	I65.22
Asymptomatic stenosis of left carotid artery	433.1	I65.22
Symptomatic stenosis of left carotid artery without infarction	433.1	I65.22
Asymptomatic stenosis of left carotid artery without infarction	433.1	I65.22
Thrombosis of left common carotid artery	433.1	I65.22
Arteriosclerosis of left carotid artery	433.1	I65.22

© 2021 Eberly LA et al. *JAMA Network Open*.

Stenosis of left carotid artery without cerebral infarction	433.1	I65.22
Thrombosis of left external carotid artery	433.1	I65.22
Stenosis of left internal carotid artery	433.1	I65.22
Symptomatic stenosis of left carotid artery	433.1	I65.22
Stenosis of intracranial portions of left internal carotid artery	433.1	I65.22
Stenosis of left carotid artery without infarction	433.1	I65.22
Stenosis of cavernous portion of left internal carotid artery	433.1	I65.22
Left-sided carotid artery obstruction	433.1	I65.22
Obstruction of left carotid artery without cerebral infarction	433.1	I65.22
Left-sided carotid artery obstruction without cerebral infarction	433.1	I65.22
Mild atherosclerosis of left carotid artery	433.1	I65.22
Obstruction of left carotid artery	433.1	I65.22
Left-sided extracranial carotid artery stenosis	433.1	I65.22
Left-sided carotid artery occlusion without cerebral infarction	433.1	I65.22
Left-sided extracranial carotid artery occlusion	433.1	I65.22
Mild atherosclerosis of carotid artery, left	433.1	I65.22
Occlusion of extracranial carotid artery, left	433.1	I65.22
Stenosis of extracranial carotid artery, left	433.1	I65.22
Greater than 50 percent stenosis of carotid artery, left	433.1	I65.22
Occlusion of carotid artery without cerebral infarction, left	433.1	I65.22
Obstruction of carotid artery without cerebral infarction, left	433.1	I65.22

© 2021 Eberly LA et al. *JAMA Network Open*.

Atherosclerosis of left common carotid artery	433.1	I65.22
Stenosis of left external carotid artery	433.1	I65.22
More than 50 percent stenosis of left internal carotid artery	433.1	I65.22
Stenosis of left carotid artery greater than 50%	433.1	I65.22
Occlusion and stenosis of left carotid artery		I65.22
Bilateral carotid artery stenosis	433.10, 433.30	I65.23
Carotid stenosis, bilateral	433.10, 433.30	I65.23
Narrowing of both carotid arteries	433.10, 433.30	I65.23
Bilateral carotid artery occlusion	433.10, 433.30	I65.23
Carotid occlusion, bilateral	433.10, 433.30	I65.23
Occlusion and stenosis of carotid arteries of both sides	433.10, 433.30	I65.23
Atherosclerosis of both carotid arteries	433.10, 433.30	I65.23
Asymptomatic bilateral carotid artery stenosis	433.10, 433.30	I65.23
Symptomatic carotid artery stenosis without infarction, bilateral	433.10, 433.30	I65.23
Carotid artery occlusion and stenosis, bilateral	433.1	I65.23
Unilateral carotid artery stenosis, bilateral	433.1	I65.23
Internal carotid artery thrombosis, bilateral	433.1	I65.23
Carotid artery narrowing, bilateral	433.10, 433.30	I65.23
Occlusion and stenosis of carotid artery without mention of cerebral infarction, bilateral	433.1	I65.23
Occlusion and stenosis of carotid artery, bilateral	433.1	I65.23
Carotid artery occlusion without infarction, bilateral	433.1	I65.23

© 2021 Eberly LA et al. *JAMA Network Open*.

Asymptomatic carotid artery stenosis, bilateral	433.10, 433.30	I65.23
Intracranial carotid stenosis, bilateral	433.10, 433.30	I65.23
Occlusion of carotid artery, bilateral	433.10, 433.30	I65.23
Recurrent carotid stenosis, bilateral	433.10, 433.30	I65.23
Common carotid artery thrombosis, bilateral	433.1	I65.23
Carotid artery narrowing, bilateral	433.10, 433.30	I65.23
Asymptomatic carotid artery stenosis without infarction, bilateral	433.10, 433.30	I65.23
Carotid stenosis, non-symptomatic, bilateral	433.10, 433.30	I65.23
Stenosis of carotid artery, bilateral	433.10, 433.30	I65.23
Internal carotid artery occlusion, bilateral	433.1	I65.23
Carotid thromboses, bilateral	433.1	I65.23
Thrombosis of internal carotid, bilateral	433.1	I65.23
Carotid artery occlusion, bilateral	433.10, 433.30	I65.23
External carotid artery thrombosis, bilateral	433.1	I65.23
Carotid artery stenosis, symptomatic, bilateral	433.10, 433.30	I65.23
Carotid stenosis, asymptomatic, bilateral	433.10, 433.30	I65.23
External carotid artery stenosis, bilateral	433.10, 433.30	I65.23
Carotid artery calcification, bilateral	433.1	I65.23
Carotid ulcer, bilateral	433.10, 433.30	I65.23
Carotid artery stenosis and occlusion, bilateral	433.1	I65.23
Carotid stenosis, symptomatic w/o infarct, bilateral	433.10, 433.30	I65.23
Common carotid artery stenosis, bilateral	433.10, 433.30	I65.23
Carotid artery stenosis, unilateral, bilateral	433.1	I65.23
Carotid artery obstruction, bilateral	433.10, 433.30	I65.23
Arteriosclerosis of carotid artery, bilateral	433.10, 433.30	I65.23

© 2021 Eberly LA et al. *JAMA Network Open*.

Carotid artery thrombosis, bilateral	433.1	I65.23
Carotid artery stenosis, asymptomatic, bilateral	433.10, 433.30	I65.23
Neck artery obstruction, bilateral	433.1	I65.23
Asymptomatic carotid artery narrowing without infarction, bilateral	433.10, 433.30	I65.23
Thrombosis of external carotid, bilateral	433.1	I65.23
Carotid artery, internal, occlusion, bilateral	433.1	I65.23
Symptomatic carotid artery narrowing without infarction, bilateral	433.10, 433.30	I65.23
Symptomatic carotid artery stenosis, bilateral	433.10, 433.30	I65.23
Carotid thrombosis, bilateral	433.1	I65.23
Carotid atherosclerosis, bilateral	433.10, 433.30	I65.23
ICAO (internal carotid artery occlusion), bilateral	433.1	I65.23
Carotid artery stenosis, bilateral	433.10, 433.30	I65.23
Internal carotid artery stenosis, bilateral	433.10, 433.30	I65.23
Carotid artery plaque, bilateral	433.10, 433.30	I65.23
Carotid artery stenosis without cerebral infarction, bilateral	433.10, 433.30	I65.23
Obstruction of carotid artery, bilateral	433.10, 433.30	I65.23
Thrombosis of both internal carotid arteries	433.1	I65.23
Symptomatic stenosis of both carotid arteries	433.10, 433.30	I65.23
Stenosis of both internal carotid arteries	433.10, 433.30	I65.23
Stenosis of both carotid arteries without cerebral infarction	433.10, 433.30	I65.23
Symptomatic stenosis of both carotid arteries without infarction	433.10, 433.30	I65.23
Thrombosis of both carotid arteries	433.1	I65.23

© 2021 Eberly LA et al. *JAMA Network Open*.

Occlusion of both internal carotid arteries	433.1	I65.23
Thrombosis of both external carotid arteries	433.1	I65.23
Arteriosclerosis of both carotid arteries	433.10, 433.30	I65.23
Thrombosis of both common carotid arteries	433.1	I65.23
Calcification of both carotid arteries	433.1	I65.23
Recurrent stenosis of both carotid arteries	433.10, 433.30	I65.23
Obstruction of neck artery of both sides	433.1	I65.23
Asymptomatic stenosis of both carotid arteries without infarction	433.10, 433.30	I65.23
Stenosis of intracranial portion of both internal carotid arteries	433.10, 433.30	I65.23
Stenosis of both carotid arteries without infarction	433.10, 433.30	I65.23
Bilateral carotid artery stenosis without cerebral infarction	433.10, 433.30	I65.23
Mild atherosclerosis of both carotid arteries	433.1	I65.23
Bilateral carotid artery obstruction without cerebral infarction	433.1	I65.23
Bilateral extracranial carotid artery stenosis	433.1	I65.23
Bilateral carotid artery occlusion without cerebral infarction	433.10, 433.30	I65.23
Bilateral extracranial carotid artery occlusion	433.10, 433.30	I65.23
Obstruction of carotid artery on both sides	433.10, 433.30	I65.23
Mild atherosclerosis of carotid artery, bilateral	433.1	I65.23
Occlusion of extracranial carotid artery, bilateral	433.10, 433.30	I65.23
Stenosis of extracranial carotid artery, bilateral	433.1	I65.23

© 2021 Eberly LA et al. *JAMA Network Open*.

Occlusion of carotid artery without cerebral infarction, bilateral	433.10, 433.30	I65.23
Greater than 50 percent stenosis of carotid artery, bilateral	433.1	I65.23
Obstruction of carotid artery without cerebral infarction, bilateral	433.1	I65.23
Stenosis of both external carotid arteries	433.3	I65.23
Occlusion and stenosis of bilateral carotid arteries	433.10, 433.30	I65.23
Bilateral stenosis of carotid arteries greater than 50%	433.1	I65.23
Occlusion and stenosis of bilateral carotid arteries		I65.23
Occlusion and stenosis of carotid artery without mention of cerebral infarction	433.1	I65.29
Carotid artery stenosis	433.1	I65.29
Carotid artery narrowing	433.1	I65.29
Carotid artery obstruction	433.1	I65.29
Carotid artery occlusion	433.1	I65.29
Carotid artery thrombosis	433.1	I65.29
Occlusion and stenosis of carotid artery	433.1	I65.29
Carotid thrombosis	433.1	I65.29
Internal carotid artery occlusion	433.1	I65.29
ICAO (internal carotid artery occlusion)	433.1	I65.29
Carotid stenosis	433.1	I65.29
External carotid artery thrombosis	433.1	I65.29
Common carotid artery thrombosis	433.1	I65.29
Internal carotid artery thrombosis	433.1	I65.29
Carotid thromboses	433.1	I65.29
External carotid artery stenosis	433.1	I65.29
Internal carotid artery stenosis	433.1	I65.29
Carotid artery narrowing	433.1	I65.29
Common carotid artery stenosis	433.1	I65.29
Carotid artery plaque	433.1	I65.29
Carotid artery, internal, occlusion	433.1	I65.29

© 2021 Eberly LA et al. *JAMA Network Open*.

Carotid atherosclerosis	433.1	I65.29
Symptomatic carotid artery stenosis without infarction	433.1	I65.29
Symptomatic carotid artery narrowing without infarction	433.1	I65.29
Asymptomatic carotid artery stenosis without infarction	433.1	I65.29
Asymptomatic carotid artery narrowing without infarction	433.1	I65.29
Carotid artery stenosis and occlusion	433.1	I65.29
Carotid artery occlusion and stenosis	433.1	I65.29
Neck artery obstruction	433.1	I65.29
Carotid artery occlusion without infarction	433.1	I65.29
Carotid artery stenosis, unilateral	433.1	I65.29
Unilateral carotid artery stenosis	433.1	I65.29
Asymptomatic carotid artery stenosis	433.1	I65.29
Carotid artery stenosis, asymptomatic	433.1	I65.29
Intracranial carotid stenosis	433.1	I65.29
Recurrent carotid stenosis	433.1	I65.29
Carotid artery calcification	433.1	I65.29
Carotid stenosis, non-symptomatic	433.1	I65.29
Carotid stenosis, symptomatic w/o infarct	433.1	I65.29
Thrombosis of external carotid	433.1	I65.29
Thrombosis of internal carotid	433.1	I65.29
Carotid stenosis, asymptomatic	433.1	I65.29
Occlusion of carotid artery	433.1	I65.29
Stenosis of carotid artery	433.1	I65.29
Symptomatic carotid artery stenosis	433.1	I65.29
Carotid artery stenosis, symptomatic	433.1	I65.29
Carotid artery stenosis and occlusion, unspecified laterality	433.1	I65.29
Carotid artery stenosis, unspecified laterality	433.1	I65.29
Carotid artery occlusion without infarction, unspecified laterality	433.1	I65.29

© 2021 Eberly LA et al. *JAMA Network Open*.

Thrombosis of external carotid, unspecified laterality	433.1	I65.29
Occlusion and stenosis of carotid artery without mention of cerebral infarction, unspecified laterality	433.1	I65.29
Symptomatic carotid artery stenosis without infarction, unspecified laterality	433.1	I65.29
Carotid ulcer, unspecified laterality	433.1	I65.29
Carotid artery calcification, unspecified laterality	433.1	I65.29
Stenosis of carotid artery, unspecified laterality	433.1	I65.29
Recurrent carotid stenosis, unspecified laterality	433.1	I65.29
Unilateral carotid artery stenosis, unspecified laterality	433.1	I65.29
Carotid artery, internal, occlusion, unspecified laterality	433.1	I65.29
External carotid artery thrombosis, unspecified laterality	433.1	I65.29
Carotid stenosis, unspecified laterality	433.1	I65.29
Carotid artery occlusion and stenosis, unspecified laterality	433.1	I65.29
Asymptomatic carotid artery narrowing without infarction, unspecified laterality	433.1	I65.29
Internal carotid artery stenosis, unspecified laterality	433.1	I65.29
Occlusion of carotid artery, unspecified laterality	433.1	I65.29
Carotid artery stenosis, symptomatic, unspecified laterality	433.1	I65.29
Carotid artery thrombosis, unspecified laterality	433.1	I65.29
Carotid thrombosis, unspecified laterality	433.1	I65.29

© 2021 Eberly LA et al. *JAMA Network Open*.

Carotid artery plaque, unspecified laterality	433.1	I65.29
Carotid artery narrowing, unspecified laterality	433.1	I65.29
Carotid artery stenosis, unilateral, unspecified laterality	433.1	I65.29
ICAO (internal carotid artery occlusion), unspecified laterality	433.1	I65.29
Symptomatic carotid artery stenosis, unspecified laterality	433.1	I65.29
Carotid artery narrowing, unspecified laterality	433.1	I65.29
Occlusion and stenosis of carotid artery, unspecified laterality	433.1	I65.29
Carotid artery obstruction, unspecified laterality	433.1	I65.29
Intracranial carotid stenosis, unspecified laterality	433.1	I65.29
Neck artery obstruction, unspecified laterality	433.1	I65.29
Carotid thromboses, unspecified laterality	433.1	I65.29
Carotid artery stenosis, asymptomatic, unspecified laterality	433.1	I65.29
Thrombosis of internal carotid, unspecified laterality	433.1	I65.29
Common carotid artery stenosis, unspecified laterality	433.1	I65.29
Carotid stenosis, asymptomatic, unspecified laterality	433.1	I65.29
Common carotid artery thrombosis, unspecified laterality	433.1	I65.29
Carotid stenosis, non-symptomatic, unspecified laterality	433.1	I65.29
Internal carotid artery occlusion, unspecified laterality	433.1	I65.29
Asymptomatic carotid artery stenosis without infarction, unspecified laterality	433.1	I65.29

© 2021 Eberly LA et al. *JAMA Network Open*.

Internal carotid artery thrombosis, unspecified laterality	433.1	I65.29
Asymptomatic carotid artery stenosis, unspecified laterality	433.1	I65.29
Carotid artery occlusion, unspecified laterality	433.1	I65.29
Symptomatic carotid artery narrowing without infarction, unspecified laterality	433.1	I65.29
Carotid stenosis, symptomatic w/o infarct, unspecified laterality	433.1	I65.29
Carotid atherosclerosis, unspecified laterality	433.1	I65.29
External carotid artery stenosis, unspecified laterality	433.1	I65.29
Arteriosclerosis of carotid artery	433.1	I65.29
Arteriosclerosis of carotid artery, unspecified laterality	433.1	I65.29
Carotid artery stenosis without infarction	433.1	I65.29
Carotid artery stenosis without cerebral infarction	433.1	I65.29
Obstruction of carotid artery	433.1	I65.29
Obstruction of carotid artery without cerebral infarction	433.1	I65.29
Occlusion of carotid artery without cerebral infarction	433.1	I65.29
Carotid artery stenosis without cerebral infarction, unspecified laterality	433.1	I65.29
Obstruction of carotid artery, unspecified laterality	433.1	I65.29
Occlusion of extracranial carotid artery	433.1	I65.29
Stenosis of extracranial carotid artery	433.1	I65.29
Greater than 50 percent stenosis of carotid artery	433.1	I65.29

© 2021 Eberly LA et al. *JAMA Network Open*.

Mild atherosclerosis of carotid artery	433.1	I65.29
Mild atherosclerosis of carotid artery, unspecified laterality	433.1	I65.29
Greater than 50 percent stenosis of carotid artery, unspecified laterality	433.1	I65.29
Stenosis of extracranial carotid artery, unspecified laterality	433.1	I65.29
Occlusion of extracranial carotid artery, unspecified laterality	433.1	I65.29
Occlusion of carotid artery without cerebral infarction, unspecified laterality	433.1	I65.29
Obstruction of carotid artery without cerebral infarction, unspecified laterality	433.1	I65.29
Carotid art occlusion w/o infarction	433.1	I65.29
Stenosis of external carotid artery	433.1	I65.29
Occlusion and stenosis of unspecified carotid artery	433.1	I65.29
Occlusion and stenosis of unspecified carotid artery		I65.29
Occlusion and stenosis of multiple and bilateral precerebral arteries without mention of cerebral infarction	433.3	I65.8
Occlusion and stenosis of other specified precerebral artery without mention of cerebral infarction	433.8	I65.8
Multiple and bilateral precerebral artery obstruction	433.3	I65.8
Occlusion and stenosis of multiple and bilateral precerebral arteries	433.3	I65.8
Precerebral artery stenosis/occlusion, multiple/bilateral	433.3	I65.8
Multiple and bilateral precerebral artery stenosis without infarction	433.3	I65.8
Occlusion and stenosis of other specified precerebral artery	433.8	I65.8

© 2021 Eberly LA et al. *JAMA Network Open*.

Other precerebral occ w/o infarction	433.8	I65.8
Occlusion and stenosis of other precerebral arteries	433.8	I65.8
Occlusion and stenosis of other precerebral arteries (CODE)	433.8	I65.8
Occlusion and stenosis of other precerebral arteries		I65.8
Pontine artery thrombosis	433.8	I65.8
Pontine artery occlusion	433.8	I65.8
Occlusion of pontine artery	433.8	I65.8
Multiple and bilateral precerebral artery stenosis	433.3	I65.9
Occlusion and stenosis of unspecified precerebral artery without mention of cerebral infarction	433.9	I65.9
Stenosis of precerebral artery	433.9	I65.9
Narrowing of precerebral artery	433.9	I65.9
Obstruction of precerebral artery	433.9	I65.9
Occlusion of precerebral artery	433.9	I65.9
Thrombosis of precerebral artery	433.9	I65.9
Multiple and bilateral precerebral artery thrombosis	433.3	I65.9
Occlusion and stenosis of precerebral artery	433.9	I65.9
Multiple precerebral artery occlusions without cerebral infarction	433.3	I65.9
Precerebral occlusion	433.9	I65.9
Precerebral artery stenosis/occlusion	433.9	I65.9
Asymptomatic stenosis of precerebral artery	433.9	I65.9
Precerebral artery stenosis, multiple or bilateral, non-symptomatic	433.3	I65.9
Precerebral artery stenosis, asymptomatic	433.9	I65.9

© 2021 Eberly LA et al. *JAMA Network Open*.

Extra-cranial artery stenosis, asymptomatic	433.9	I65.9
Precerebral artery occlusion	433.9	I65.9
Extra-cranial artery stenosis, multiple or bilateral, asymptomatic	433.3	I65.9
Extracranial artery stenosis and occlusion	433.9	I65.9
Occlusion and stenosis of precerebral arteries	433.9	I65.9
Occlusion and stenosis of unspecified precerebral artery	433.9	I65.9
Multiple and bilateral precerebral arterial occlusion	433.3	I65.9
Occlusion and stenosis of precerebral artery without cerebral infarction	433.9	I65.9
Multiple precerebral occ w/o infarction	433.3	I65.9
Precerebral occlusion w/o infarct	433.9	I65.9
Occlusion and stenosis of unspecified precerebral artery		I65.9
Occlusion and stenosis of cerebral arteries, not resulting in cerebral infarction		I66
Occlusion and stenosis of middle cerebral artery		I66.0
Occlusion and stenosis of right middle cerebral artery	434.9	I66.01
Occlusion and stenosis of middle cerebral artery, right	434.9	I66.01
Middle cerebral artery stenosis, right	437	I66.01
Stenosis of middle cerebral artery, right	437	I66.01
Occlusion or stenosis of middle cerebral artery without infarction, right	434.9	I66.01
Thrombosis of middle cerebral artery, right	434	I66.01
Occlusion of middle cerebral artery, right	434.9	I66.01
Stenosis of right middle cerebral artery	437	I66.01

© 2021 Eberly LA et al. *JAMA Network Open*.

Thrombosis of right middle cerebral artery	434	I66.01
Occlusion of right middle cerebral artery	434.9	I66.01
Stenosis of right middle cerebral artery not resulting in cerebral infarction	434.9	I66.01
Occlusion of right middle cerebral artery not resulting in cerebral infarction	434.9	I66.01
Occlusion and stenosis of right middle cerebral artery		I66.01
Occlusion and stenosis of left middle cerebral artery	434.9	I66.02
Middle cerebral artery stenosis, left	437	I66.02
Occlusion or stenosis of middle cerebral artery without infarction, left	434.9	I66.02
Occlusion and stenosis of middle cerebral artery, left	434.9	I66.02
Stenosis of middle cerebral artery, left	437	I66.02
Thrombosis of middle cerebral artery, left	434	I66.02
Occlusion of middle cerebral artery, left	434.9	I66.02
Thrombosis of left middle cerebral artery	434	I66.02
Stenosis of left middle cerebral artery	437	I66.02
Occlusion of left middle cerebral artery	434.9	I66.02
Stenosis of left middle cerebral artery not resulting in cerebral infarction	434.9	I66.02
Occlusion of left middle cerebral artery not resulting in cerebral infarction	434.9	I66.02
Occlusion and stenosis of left middle cerebral artery		I66.02
Occlusion and stenosis of both middle cerebral arteries	434.9	I66.03
Stenosis of middle cerebral artery, bilateral	437	I66.03
Middle cerebral artery stenosis, bilateral	437	I66.03
Occlusion and stenosis of middle cerebral artery, bilateral	434.9	I66.03
Occlusion or stenosis of middle cerebral artery without infarction, bilateral	434.9	I66.03

© 2021 Eberly LA et al. *JAMA Network Open*.

Thrombosis of middle cerebral artery, bilateral	434	I66.03
Occlusion of middle cerebral artery, bilateral	434.9	I66.03
Occlusion of both middle cerebral arteries	434.9	I66.03
Stenosis of both middle cerebral arteries	437	I66.03
Thrombosis of both middle cerebral arteries	434	I66.03
Stenosis of both middle cerebral arteries not resulting in cerebral infarction	434.9	I66.03
Occlusion of both middle cerebral arteries not resulting in cerebral infarction	434.9	I66.03
Bilateral occlusion of middle cerebral arteries not resulting in cerebral infarction	434.9	I66.03
Bilateral stenosis of middle cerebral arteries not resulting in cerebral infarction	434.9	I66.03
Occlusion and stenosis of bilateral middle cerebral arteries	434.9	I66.03
Occlusion and stenosis of bilateral middle cerebral arteries		I66.03
Occlusion and stenosis of middle cerebral artery	434.9	I66.09
Stenosis of middle cerebral artery	437	I66.09
Middle cerebral artery stenosis	437	I66.09
Occlusion or stenosis of middle cerebral artery without infarction	434.9	I66.09
Occlusion and stenosis of middle cerebral artery, unspecified laterality	434.9	I66.09
Middle cerebral artery stenosis, unspecified laterality	437	I66.09
Occlusion or stenosis of middle cerebral artery without infarction, unspecified laterality	434.9	I66.09

© 2021 Eberly LA et al. *JAMA Network Open*.

Stenosis of middle cerebral artery, unspecified laterality	437	I66.09
Thrombosis of middle cerebral artery	434	I66.09
Thrombosis of middle cerebral artery, unspecified laterality	434	I66.09
Occlusion of middle cerebral artery	434.9	I66.09
Occlusion of middle cerebral artery, unspecified laterality	434.9	I66.09
Occlusion and stenosis of unspecified middle cerebral artery	434.9	I66.09
Occlusion and stenosis of unspecified middle cerebral artery		I66.09
Occlusion and stenosis of anterior cerebral artery		I66.1
Occlusion and stenosis of right anterior cerebral artery	434.9	I66.11
Occlusion and stenosis of anterior cerebral artery, right	434.9	I66.11
Thrombosis of anterior cerebral artery, right	434	I66.11
Thrombosis of right anterior cerebral artery	434	I66.11
Occlusion and stenosis of right anterior cerebral artery		I66.11
Occlusion and stenosis of left anterior cerebral artery	434.9	I66.12
Occlusion and stenosis of anterior cerebral artery, left	434.9	I66.12
Thrombosis of anterior cerebral artery, left	434	I66.12
Thrombosis of left anterior cerebral artery	434	I66.12
Occlusion and stenosis of left anterior cerebral artery		I66.12
Occlusion and stenosis of both anterior cerebral arteries	434.9	I66.13

© 2021 Eberly LA et al. *JAMA Network Open*.

Thrombosis of anterior cerebral artery, bilateral	434	I66.13
Occlusion and stenosis of anterior cerebral artery, bilateral	434.9	I66.13
Thrombosis of both anterior cerebral arteries	434	I66.13
Occlusion and stenosis of bilateral anterior cerebral arteries	434.9	I66.13
Occlusion and stenosis of bilateral anterior cerebral arteries		I66.13
Occlusion and stenosis of anterior cerebral artery	434.9	I66.19
Occlusion and stenosis of anterior cerebral artery, unspecified laterality	434.9	I66.19
Thrombosis of anterior cerebral artery	434	I66.19
Thrombosis of anterior cerebral artery, unspecified laterality	434	I66.19
Occlusion and stenosis of unspecified anterior cerebral artery	434.9	I66.19
Occlusion and stenosis of unspecified anterior cerebral artery		I66.19
Occlusion and stenosis of posterior cerebral artery		I66.2
Occlusion and stenosis of right posterior cerebral artery	434.9	I66.21
Occlusion or stenosis of posterior cerebral artery without infarction, right	437	I66.21
Occlusion and stenosis of posterior cerebral artery, right	434.9	I66.21
Thrombosis of posterior cerebral artery, right	434	I66.21
Thrombosis of right posterior cerebral artery	434	I66.21
Occlusion and stenosis of right posterior cerebral artery		I66.21

© 2021 Eberly LA et al. *JAMA Network Open*.

Occlusion and stenosis of left posterior cerebral artery	434.9	I66.22
Occlusion or stenosis of posterior cerebral artery without infarction, left	437	I66.22
Occlusion and stenosis of posterior cerebral artery, left	434.9	I66.22
Thrombosis of posterior cerebral artery, left	434	I66.22
Posterior cerebral artery embolism, left	434.1	I66.22
Thrombosis of left posterior cerebral artery	434	I66.22
Occlusion and stenosis of left posterior cerebral artery		I66.22
Occlusion and stenosis of both posterior cerebral arteries	434.9	I66.23
Thrombosis of posterior cerebral artery, bilateral	434	I66.23
Occlusion or stenosis of posterior cerebral artery without infarction, bilateral	437	I66.23
Occlusion and stenosis of posterior cerebral artery, bilateral	434.9	I66.23
Posterior cerebral artery embolism, bilateral	434.1	I66.23
Thrombosis of both posterior cerebral arteries	434	I66.23
Occlusion and stenosis of bilateral posterior cerebral arteries	434.9	I66.23
Occlusion and stenosis of bilateral posterior cerebral arteries		I66.23
Occlusion and stenosis of posterior cerebral artery	434.9	I66.29
Occlusion or stenosis of posterior cerebral artery without infarction	437	I66.29
Occlusion or stenosis of posterior cerebral artery without infarction, unspecified laterality	437	I66.29

© 2021 Eberly LA et al. *JAMA Network Open*.

Occlusion and stenosis of posterior cerebral artery, unspecified laterality	434.9	I66.29
Thrombosis of posterior cerebral artery	434	I66.29
Thrombosis of posterior cerebral artery, unspecified laterality	434	I66.29
Occlusion and stenosis of unspecified posterior cerebral artery	434.9	I66.29
Occlusion and stenosis of unspecified posterior cerebral artery		I66.29
Occlusion and stenosis of cerebellar arteries	433.8	I66.3
Cerebellar artery occlusion or stenosis	433.8	I66.3
Thrombosis of anterior inferior cerebellar artery	433.8	I66.3
Thrombosis of posterior inferior cerebellar artery	433.8	I66.3
Thrombosis of superior cerebellar artery	433.8	I66.3
Cerebellar artery thrombosis	433.8	I66.3
Cerebellar artery occlusion	433.8	I66.3
Occlusion of cerebellar artery	433.8	I66.3
Thrombosis of cerebellar artery	433.8	I66.3
Occlusion and stenosis of cerebellar arteries		I66.3
Occlusion and stenosis of other cerebral arteries	434.9	I66.8
Occlusion and stenosis of other cerebral arteries (CODE)	434.9	I66.8
Occlusion and stenosis of other cerebral arteries		I66.8
Cerebral thrombosis without mention of cerebral infarction	434	I66.9
Unspecified cerebral artery occlusion without mention of cerebral infarction	434.9	I66.9

© 2021 Eberly LA et al. *JAMA Network Open*.

Cerebral artery occlusion	434.9	I66.9
Thrombosis of cerebral arteries	434	I66.9
Occlusion, artery, cerebral	434.9	I66.9
Occlusion, cerebral artery	434.9	I66.9
Cerebral thrombosis	434	I66.9
Blood clots in brain	434	I66.9
Cerebral arterial thrombosis	434	I66.9
Cerebral artery occlusion syndrome	434.9	I66.9
CT (cerebral thrombosis)	434	I66.9
Cerebrovascular occlusion	434.9	I66.9
Occlusion of cerebral arteries	434.9	I66.9
Asymptomatic cerebral artery occlusion	434.9	I66.9
Cerebral thrombosis with transient symptoms	434	I66.9
Occlusion or stenosis of cerebral artery without infarction	434.9	I66.9
Cerebral artery occlusion, asymptomatic	434.9	I66.9
Occlusion or stenosis of multiple cerebral arteries	434.9	I66.9
Occlusion and stenosis of cerebral artery	434.9	I66.9
Cerebral artery occlusion, non-symptomatic	434.9	I66.9
Cerebral thrombosis with transient ischemic attack (TIA)	434	I66.9
Unspecified cerebral artery occlusion	434.9	I66.9
Cerebral artery occlusion without cerebral infarction	434.9	I66.9
Cerebral thrombosis without cerebral infarction	434	I66.9
Cerebral art occlusion w/o infarct	434.9	I66.9
Stenosis of intracranial vessel	437	I66.9
Occlusion and stenosis of unspecified cerebral artery	434.9	I66.9

© 2021 Eberly LA et al. *JAMA Network Open*.

Occlusion and stenosis of unspecified cerebral artery		I66.9
Other cerebrovascular diseases		I67
Dissection of cerebral arteries, nonruptured	443.29	I67.0
Dissection of intracranial artery	443.29	I67.0
Dissection of cerebral artery	443.29	I67.0
Dissection of cerebral arteries, nonruptured		I67.0
Cerebral atherosclerosis	437	I67.2
Atheroma of cerebral arteries	437	I67.2
Cerebral arteriosclerosis	437	I67.2
Cerebrovascular arteriosclerosis	437	I67.2
Atherosclerotic cerebrovascular disease	437	I67.2
CAS (cerebral atherosclerosis)	437	I67.2
Intracranial arteriosclerosis	437	I67.2
Intracranial atherosclerosis	437	I67.2
Hardening of the arteries of the brain	437	I67.2
Arteriosclerosis of cerebral artery	437	I67.2
Arteriosclerotic leukoencephalopathy	437.0, 323.81	I67.2
Arteriosclerotic cerebrovascular disease	437	I67.2
Cerebral atherosclerosis		I67.2
Dementia due to arteriosclerosis with behavioral disturbance	290.40, 437.0	I67.2, F01.51
Dementia due to atherosclerosis with behavioral disturbance	290.40, 437.0	I67.2, F01.51
Familial arteriosclerotic leukoencephalopathy with alopecia and lumbago, without arterial hypertension	437.0, 704.00, 724.2	I67.2, L65.9, M54.5
Cerebral arteriosclerosis with history of previous stroke	437.0, V12.54	I67.2, Z86.73
Cerebrovascular disease, arteriosclerotic, post-stroke	437.0, V12.54	I67.2, Z86.73
Cerebral arteriosclerosis with history of previous cerebrovascular accident	437.0, V12.54	I67.2, Z86.73
Systolic hypertension with cerebrovascular disease	437.2	I67.4

© 2021 Eberly LA et al. *JAMA Network Open*.

Progressive intracranial arterial occlusion	437.5	I67.5
Progressive intracranial arterial occlusion syndrome	437.5	I67.5
Other specified cerebrovascular diseases		I67.8
Cerebrovascular insufficiency	437.9	I67.81
Acute cerebrovascular insufficiency	437.1	I67.81
Cerebrovascular insufficiency syndrome	437.9	I67.81
Cerebrovascular insufficiency, acute	437.1	I67.81
Cerebral artery insufficiency	437.1	I67.81
Insufficiency, arterial, cerebral	437.1	I67.81
Acute cerebrovascular insufficiency		I67.81
Chronic cerebral ischemia	437.1	I67.82
Cerebral ischemia	437.1	I67.82
Brain ischemia	437.1	I67.82
Ischemic brain injury	437.1	I67.82
Subcortical microvascular ischemic occlusive disease	437.1	I67.82
Ischemic changes on computed tomography of head	437.1	I67.82
Ischemic changes on head CT	437.1	I67.82
Cerebral ischemia		I67.82
Chronic hypoxic-ischemic brain injury	437.1, 348.1	I67.82, G93.1
Acute, but ill-defined, cerebrovascular disease	436	I67.89
Other generalized ischemic cerebrovascular disease	437.1	I67.89
Other ill-defined cerebrovascular disease	437.8	I67.89
Acute ill-defined cerebrovascular disease	436	I67.89
Generalized ischemic cerebrovascular disease	437.1	I67.89
Ischemic encephalopathy	437.1	I67.89
Cerebrovascular disease, acute	436	I67.89
Cerebrovascular disease, ill-defined, acute	436	I67.89

© 2021 Eberly LA et al. *JAMA Network Open*.

Other cerebrovascular disease	437.8	I67.89
Other and ill-defined cerebrovascular disease	437.8	I67.89
Other cerebrovascular disease		I67.89
Acute confusional state of cerebrovascular origin	437.1, 293.0	I67.89, F05
Flaccid hemiplegia of right dominant side due to other cerebrovascular disease	437.8, 342.01	I67.89, G81.01
Flaccid hemiplegia of left dominant side due to other cerebrovascular disease	437.8, 342.01	I67.89, G81.02
Flaccid hemiplegia of right nondominant side due to other cerebrovascular disease	437.8, 342.02	I67.89, G81.03
Flaccid hemiplegia of left nondominant side due to other cerebrovascular disease	437.8, 342.02	I67.89, G81.04
Spastic hemiparesis of right dominant side due to other cerebrovascular disease	438.21	I67.89, G81.11
Spastic hemiplegia of right dominant side due to other cerebrovascular disease	437.8, 342.11	I67.89, G81.11
Spastic hemiparesis of left dominant side due to other cerebrovascular disease	438.21	I67.89, G81.12
Spastic hemiplegia of left dominant side due to other cerebrovascular disease	437.8, 342.11	I67.89, G81.12
Spastic hemiplegia of right nondominant side due to other cerebrovascular disease	437.8, 342.12	I67.89, G81.13
Spastic hemiplegia of left nondominant side due to other cerebrovascular disease	437.8, 342.12	I67.89, G81.14
Hemiparesis due to other cerebrovascular disease, unspecified laterality	438.2	I67.89, G81.90
Hemiparesis of right dominant side due to other cerebrovascular disease	438.21	I67.89, G81.91
Hemiparesis of left dominant side due to other cerebrovascular disease	438.21	I67.89, G81.92
Hemiparesis of right nondominant side due to other cerebrovascular disease	438.22	I67.89, G81.93
Hemiparesis of left nondominant side due to other cerebrovascular disease	438.22	I67.89, G81.94

© 2021 Eberly LA et al. *JAMA Network Open*.

Cerebrovascular disease, unspecified	437.9	I67.9
Cerebrovascular lesion	437.9	I67.9
Rolandic vein occlusion syndrome	437.8	I67.9
Merwarth's vein occlusion syndrome	437.8	I67.9
Artery disease, cerebral	437.9	I67.9
Cerebral artery disease	437.9	I67.9
Cerebral vascular disturbance	437.9	I67.9
Cerebrovascular disease	437.9	I67.9
Cerebrovascular disorder	437.9	I67.9
Cerebral vascular insufficiency	437.9	I67.9
Cerebrovascular disease or lesion	437.9	I67.9
Ill-defined cerebrovascular disease	437.8	I67.9
Cerebral vascular disorder	437.9	I67.9
CVD (cerebrovascular disease)	437.9	I67.9
Brain vascular disorder	437.9	I67.9
Cerebral arterial disease	437.9	I67.9
Intracranial vascular disease	437.9	I67.9
Intracranial vascular disorder	437.9	I67.9
Cerebral microvascular disease	437.9	I67.9
Cerebral microvasculopathy	437.9	I67.9
Cerebral vascular disease	437.9	I67.9
Cortical paralysis of fixation syndrome	437.8	I67.9
Cerebrovascular small vessel disease	437.9	I67.9
Small vessel disease, cerebrovascular	437.9	I67.9
Intracranial vascular stenosis	437.9	I67.9
Diffuse cerebrovascular disease	437.9	I67.9
Disorder of intracranial venous sinus	437.9	I67.9
Cerebrovascular disease, unspecified		I67.9
Cerebrovascular disease in cancer patient	437.9, 199.1	I67.9, C80.1
Facial paresis due to cerebrovascular disease	437.9, 351.0	I67.9, G51.0
Flaccid hemiplegia due to cerebrovascular disease	437.9, 342.00	I67.9, G81.00
Flaccid hemiplegia of right dominant side due to cerebrovascular disease	437.9, 342.01	I67.9, G81.01

© 2021 Eberly LA et al. *JAMA Network Open*.

Flaccid hemiplegia of left dominant side due to cerebrovascular disease	437.9, 342.01	I67.9, G81.02
Flaccid hemiplegia of right nondominant side due to cerebrovascular disease	437.9, 342.02	I67.9, G81.03
Flaccid hemiplegia of left nondominant side due to cerebrovascular disease	437.9, 342.02	I67.9, G81.04
Spastic hemiplegia due to cerebrovascular disease	437.9, 342.10	I67.9, G81.10
Spastic hemiparesis due to cerebrovascular disease	438.2	I67.9, G81.10
Spastic hemiplegia of right dominant side due to cerebrovascular disease	437.9, 342.11	I67.9, G81.11
Spastic hemiparesis of right dominant side due to cerebrovascular disease	438.21	I67.9, G81.11
Spastic hemiplegia of left dominant side due to cerebrovascular disease	437.9, 342.11	I67.9, G81.12
Spastic hemiparesis of left dominant side due to cerebrovascular disease	438.21	I67.9, G81.12
Spastic hemiplegia of right nondominant side due to cerebrovascular disease	437.9, 342.12	I67.9, G81.13
Spastic hemiparesis of right nondominant side due to cerebrovascular disease	438.22	I67.9, G81.13
Spastic hemiplegia of left nondominant side due to cerebrovascular disease	437.9, 342.12	I67.9, G81.14
Spastic hemiparesis of left nondominant side due to cerebrovascular disease	438.22	I67.9, G81.14
Hemiplegia due to cerebrovascular disease	437.9, 342.90	I67.9, G81.90
Hemiparesis due to cerebrovascular disease	438.2	I67.9, G81.90
Hemiparesis due to cerebrovascular disease, unspecified cerebrovascular disease type, unspecified laterality	438.2	I67.9, G81.90
Hemiparesis of right dominant side due to cerebrovascular disease	438.21	I67.9, G81.91

© 2021 Eberly LA et al. *JAMA Network Open*.

Hemiparesis of right dominant side due to cerebrovascular disease, unspecified cerebrovascular disease type	438.21	I67.9, G81.91
Hemiplegia of right dominant side due to cerebrovascular disease	438.21	I67.9, G81.91
Hemiparesis of left dominant side due to cerebrovascular disease	438.21	I67.9, G81.92
Hemiparesis of left dominant side due to cerebrovascular disease, unspecified cerebrovascular disease type	438.21	I67.9, G81.92
Hemiplegia of left dominant side due to cerebrovascular disease	438.21	I67.9, G81.92
Hemiparesis of right nondominant side due to cerebrovascular disease	438.22	I67.9, G81.93
Hemiparesis of right nondominant side due to cerebrovascular disease, unspecified cerebrovascular disease type	438.22	I67.9, G81.93
Hemiplegia of right nondominant side due to cerebrovascular disease	438.22	I67.9, G81.93
Hemiparesis of left nondominant side due to cerebrovascular disease	438.22	I67.9, G81.94
Hemiparesis of left nondominant side due to cerebrovascular disease, unspecified cerebrovascular disease type	438.22	I67.9, G81.94
Hemiplegia of left nondominant side due to cerebrovascular disease	438.22	I67.9, G81.94
Disturbances of vision due to cerebrovascular disease	437.9, 368.9	I67.9, H53.9
Ataxia due to cerebrovascular disease	437.9, 781.3	I67.9, R27.0
Uncoordinated movements due to circulatory disease of brain	437.9, 781.3	I67.9, R27.0
Facial weakness due to cerebrovascular disease	437.9, 781.94	I67.9, R29.810

© 2021 Eberly LA et al. *JAMA Network Open*.

Cerebrovascular disorders in diseases classified elsewhere		I68
Other cerebrovascular disorders in diseases classified elsewhere	437.8	I68.8
Other cerebrovascular disorders in diseases classified elsewhere		I68.8
Sequelae of cerebrovascular disease		I69
Sequelae of cerebral infarction		I69.3
Chronic unilateral cerebral infarction, watershed distribution	434.91	I69.30
Cerebral infarction, watershed distribution, unilateral, chronic	434.91	I69.30
Sequelae of cerebral infarction	438.9	I69.30
Chronic bilateral cerebral infarction in watershed distribution	V12.54	I69.30
Arterial ischemic stroke, ICA (internal carotid artery), left, chronic	V12.54	I69.30
Chronic left arterial ischemic stroke, ICA (internal carotid artery)	V12.54	I69.30
Chronic ischemic left ICA stroke	V12.54	I69.30
Chronic right arterial ischemic stroke, ICA (internal carotid artery)	V12.54	I69.30
Arterial ischemic stroke, ICA (internal carotid artery), right, chronic	V12.54	I69.30
Chronic ischemic right ica stroke	V12.54	I69.30
Cerebral infarction, watershed distribution, bilateral, chronic	V12.54	I69.30
Arterial ischemic stroke, MCA (middle cerebral artery), left, chronic	V12.54	I69.30
Chronic left arterial ischemic stroke, MCA (middle cerebral artery)	V12.54	I69.30
Chronic ischemic left MCA stroke	V12.54	I69.30
Arterial ischemic stroke, MCA (middle cerebral artery), right, chronic	V12.54	I69.30

© 2021 Eberly LA et al. *JAMA Network Open*.

Chronic right arterial ischemic stroke, MCA (middle cerebral artery)	V12.54	I69.30
Chronic ischemic right MCA stroke	V12.54	I69.30
Chronic left arterial ischemic stroke, ACA (anterior cerebral artery)	V12.54	I69.30
Chronic ischemic left ACA stroke	V12.54	I69.30
Chronic right arterial ischemic stroke, ACA (anterior cerebral artery)	438.9	I69.30
Arterial ischemic stroke, ACA (anterior cerebral artery), right, chronic	438.9	I69.30
Chronic ischemic right ACA stroke	438.9	I69.30
Chronic left arterial ischemic stroke, PCA (posterior cerebral artery)	V12.54	I69.30
Arterial ischemic stroke, PCA (posterior cerebral artery), left, chronic	V12.54	I69.30
Chronic ischemic left PCA stroke	V12.54	I69.30
Arterial ischemic stroke, PCA (posterior cerebral artery), right, chronic	V12.54	I69.30
Chronic ischemic right PCA stroke	V12.54	I69.30
Arterial ischemic stroke, vertebrobasilar, brainstem, chronic	V12.54	I69.30
Chronic arterial ischemic stroke, vertebrobasilar, brainstem	V12.54	I69.30
Chronic ischemic vertebrobasilar artery brainstem stroke	V12.54	I69.30
Arterial ischemic stroke, vertebrobasilar, thalamic, chronic	V12.54	I69.30
Chronic ischemic vertebrobasilar artery thalamic stroke	V12.54	I69.30
Arterial ischemic stroke, multifocal, anterior circulation, chronic	V12.54	I69.30
Chronic arterial ischemic stroke, multifocal, anterior circulation	V12.54	I69.30

© 2021 Eberly LA et al. *JAMA Network Open*.

Chronic ischemic multifocal anterior circulation stroke	V12.54	I69.30
Arterial ischemic stroke, multifocal, multi vascular territories, chronic	V12.54	I69.30
Chronic ischemic multifocal multiple vascular territories stroke	V12.54	I69.30
Arterial ischemic stroke, multifocal, posterior circulation, chronic	V12.54	I69.30
Chronic arterial ischemic stroke, multifocal, posterior circulation	V12.54	I69.30
Chronic ischemic multifocal posterior circulation stroke	V12.54	I69.30
Personal history of stroke with residual effects	438.9	I69.30
History of stroke with residual effects	438.9	I69.30
H/O: stroke with residual effects	438.9	I69.30
Arterial ischemic stroke, chronic	V12.54	I69.30
Chronic arterial ischemic stroke	V12.54	I69.30
Sequelae, post-stroke	438.9	I69.30
Late effect of stroke	438.9	I69.30
Sequela, post-stroke	438.9	I69.30
Complications of stroke	438.9	I69.30
Chronic ischemic left anterior cerebral artery stroke	V12.54	I69.30
Chronic ischemic left internal carotid artery stroke	V12.54	I69.30
Chronic ischemic left middle cerebral artery stroke	V12.54	I69.30
Chronic ischemia posterior cerebral artery stroke	438.9	I69.30
Chronic ischemic right anterior cerebral artery stroke	438.9	I69.30
Chronic ischemic right internal carotid artery stroke	V12.54	I69.30
Chronic ischemic right middle cerebral artery stroke	V12.54	I69.30

© 2021 Eberly LA et al. *JAMA Network Open*.

Chronic ischemic right posterior cerebral artery stroke	V12.54	I69.30
Chronic ischemic left anterior cerebral artery (ACA) stroke	V12.54	I69.30
Chronic ischemic left internal carotid artery (ICA) stroke	V12.54	I69.30
Chronic ischemic left middle cerebral artery (MCA) stroke	V12.54	I69.30
Chronic ischemic right anterior cerebral artery (ACA) stroke	438.9	I69.30
Chronic ischemic right internal carotid artery (ICA) stroke	V12.54	I69.30
Chronic ischemic right middle cerebral artery (MCA) stroke	V12.54	I69.30
Chronic ischemic right posterior cerebral artery (PCA) stroke	V12.54	I69.30
Chronic ischemic left posterior cerebral artery (PCA) stroke	V12.54	I69.30
Chronic ischemic left posterior cerebral artery stroke	V12.54	I69.30
Arterial ischemic stroke, ICA (internal carotid artery), right, chronic	V12.54	I69.30
Arterial ischemic stroke, ACA (anterior cerebral artery), left, chronic	V12.54	I69.30
Arterial ischemic stroke, ACA (anterior cerebral artery), right, chronic	438.9	I69.30
Arterial ischemic stroke, PCA (posterior cerebral artery), left, chronic	V12.54	I69.30
Arterial ischemic stroke, PCA (posterior cerebral artery), right, chronic	V12.54	I69.30
Chronic right arterial ischemic stroke, PCA (posterior cerebral artery)	V12.54	I69.30
Arterial ischemic stroke, multifocal, multiple vascular territories, chronic	V12.54	I69.30
Chronic arterial ischemic stroke, multifocal, multiple vascular territories	V12.54	I69.30

© 2021 Eberly LA et al. *JAMA Network Open*.

Multi-infarct state	438.9	I69.30
Chronic cerebrovascular accident	434.91	I69.30
Late effect of lacunar infarction	438.9	I69.30
Sequela of cerebrovascular accident	438.9	I69.30
History of cerebrovascular accident with residual deficit	438.9	I69.30
History of CVA with residual deficit	438.9	I69.30
History of stroke with current residual effects	438.9	I69.30
Personal history of stroke with current residual effects	438.9	I69.30
Late effect of cerebrovascular accident	438.9	I69.30
History of ischemic cerebrovascular accident with residual deficit	438.9	I69.30
History of stroke with residual deficit	438.9	I69.30
Chronic cerebrovascular accident (CVA)	434.91	I69.30
History of cerebrovascular accident (CVA) with residual deficit	438.9	I69.30
History of ischemic cerebrovascular accident (CVA) with residual deficit	438.9	I69.30
Late effect of cerebrovascular accident (CVA)	438.9	I69.30
Cerebral multi-infarct state	438.9	I69.30
Unspecified sequelae of cerebral infarction	438.9	I69.30
Multi infarct state	438.9	I69.30
Late effects of cerebral ischemic stroke	438.9	I69.30
Late effect of ischemic cerebral stroke	438.9	I69.30
Unspecified sequelae of cerebral infarction		I69.30
Cognitive deficits following cerebral infarction		I69.31
Attention and concentration deficit following cerebral infarction	438	I69.310

© 2021 Eberly LA et al. *JAMA Network Open*.

Attention and concentration deficit following cerebral infarction		I69.310
Memory deficit after cerebral infarction	438.0, 780.93	I69.311
Memory deficit following cerebral infarction	438.0, 780.93	I69.311
Memory deficit following cerebral infarction		I69.311
Visuospatial deficit and spatial neglect after cerebral infarction	438.7, 799.53, 781.8	I69.312
Visuospatial deficit and spatial neglect following cerebral infarction	438.7, 799.53, 781.8	I69.312
Visuospatial deficit and spatial neglect following cerebral infarction		I69.312
Psychomotor deficit after cerebral infarction	438.89, 799.54	I69.313
Psychomotor deficit following cerebral infarction	438.89, 799.54	I69.313
Psychomotor deficit following cerebral infarction		I69.313
Frontal lobe and executive function deficit following cerebral infarction	438	I69.314
Frontal lobe and executive function deficit following cerebral infarction		I69.314
Cognitive social or emotional deficit following cerebral infarction	438	I69.315
Cognitive social or emotional deficit following cerebral infarction		I69.315
Other symptoms and signs involving cognitive functions following cerebral infarction	799.59	I69.318
Other symptoms and signs involving cognitive functions following cerebral infarction		I69.318
Residual cognitive deficit as late effect of cerebrovascular accident	438	I69.319

© 2021 Eberly LA et al. *JAMA Network Open*.

Residual cognitive deficit as late effect of stroke	438	I69.319
Cognitive deficit status post cerebrovascular accident	438	I69.319
Cognitive deficit S/P CVA (cerebrovascular accident)	438	I69.319
CVA, old, cognitive deficits	438	I69.319
Cognitive deficit due to old cerebral infarction	438	I69.319
Cognitive deficit due to old lacunar stroke	438	I69.319
Cognitive deficit, post-stroke	438	I69.319
Cognitive deficits following cerebral infarction	438	I69.319
Cognitive deficit due to recent cerebral infarction	438	I69.319
Cognitive deficit due to recent cerebrovascular accident	438	I69.319
Cognitive deficit due to recent stroke	438	I69.319
Multiple old cerebral infarcts with cognitive deficit	438	I69.319
Cognitive deficit due to recent cerebrovascular accident (CVA)	438	I69.319
Cognitive deficit following cerebrovascular accident (CVA)	438	I69.319
Cognitive deficit due to old cerebrovascular accident (CVA)	438	I69.319
Cognitive deficit due to old subcortical infarcts	438	I69.319
Unspecified symptoms and signs involving cognitive functions following cerebral infarction	438	I69.319
Unspecified symptoms and signs involving cognitive functions following cerebral infarction		I69.319

© 2021 Eberly LA et al. *JAMA Network Open*.

Speech and language deficits following cerebral infarction		I69.32
Aphasia S/P CVA	438.11	I69.320
Aphasia, post-stroke	438.11	I69.320
Aphasia, late effect of prenatal or perinatal stroke	438.11	I69.320
CVA, old, aphasia	438.11	I69.320
Aphasia due to old cerebral infarction	438.11	I69.320
Aphasia following cerebral infarction	438.11	I69.320
Aphasia as late effect of cerebrovascular accident	438.11	I69.320
Aphasia as late effect of stroke	438.11	I69.320
Aphasia due to recent cerebrovascular accident	438.11	I69.320
Aphasia due to recent cerebral infarction	438.11	I69.320
Aphasia due to recent stroke	438.11	I69.320
Aphasia due to old brainstem infarction	438.11	I69.320
Aphasia due to recent cerebrovascular accident (CVA)	438.11	I69.320
Aphasia as late effect of cerebrovascular accident (CVA)	438.11	I69.320
Aphasia following cerebral infarction		I69.320
Dysphasia status post cerebrovascular accident	438.12	I69.321
Dysphasia S/P CVA (cerebrovascular accident)	438.12	I69.321
Dysphasia due to old cerebrovascular accident	438.12	I69.321
Dysphasia due to old stroke	438.12	I69.321
Dysphasia, post-stroke	438.12	I69.321
Dysphasia following cerebral infarction	438.12	I69.321

© 2021 Eberly LA et al. *JAMA Network Open*.

Dysphasia due to recent cerebrovascular accident	438.12	I69.321
Dysphasia due to recent stroke	438.12	I69.321
Dysphasia due to recent cerebrovascular accident (CVA)	438.12	I69.321
Dysphasia following cerebrovascular accident (CVA)	438.12	I69.321
Dysphasia as late effect of cerebrovascular accident (CVA)	438.12	I69.321
Dysphasia following cerebral infarction		I69.321
CVA, old, dysarthria	438.13	I69.322
Dysarthria following cerebrovascular accident	438.13	I69.322
Dysarthria due to old brainstem infarction	438.13	I69.322
Dysarthria due to old lacunar stroke	438.13	I69.322
Dysarthria, post-stroke	438.13	I69.322
Dysarthria following cerebral infarction	438.13	I69.322
Dysarthria as late effect of stroke	438.13	I69.322
Dysarthria due to recent cerebral infarction	438.13	I69.322
Dysarthria due to recent stroke	438.13	I69.322
Dysarthria due to recent cerebrovascular accident	438.13	I69.322
Multiple old cerebral infarcts with dysarthria	438.13	I69.322
Dysarthria due to recent cerebrovascular accident (CVA)	438.13	I69.322
Dysarthria as late effect of cerebrovascular accident (CVA)	438.13	I69.322
Dysarthria following cerebral infarction		I69.322
Fluency disorder, post-stroke	438.14	I69.323
Fluency disorder following cerebral infarction	438.14	I69.323
Fluency disorder as late effect of stroke	438.14	I69.323

© 2021 Eberly LA et al. *JAMA Network Open*.

Fluency disorder following cerebral infarction		I69.323
CVA, old, speech/language deficit	438.1	I69.328
Speech or language deficit following cerebrovascular accident	438.1	I69.328
Speech and language deficit due to old cerebral infarction	438.1	I69.328
Speech and language deficit due to old cerebrovascular accident	438.1	I69.328
Speech and language deficit due to old stroke	438.1	I69.328
Speech or language deficit, post-stroke	438.1	I69.328
Speech and language deficit as late effect of stroke	438.1	I69.328
Speech and language deficit as late effect of cerebrovascular accident (CVA)	438.1	I69.328
Other speech and language deficits following cerebral infarction	438.19	I69.328
Other speech and language deficits following cerebral infarction		I69.328
Monoplegia of upper limb following cerebral infarction		I69.33
Monoplegia of arm after cerebral infarct affecting right dominant side	438.31	I69.331
Monoplegia of upper extremity following cerebral infarction affecting right dominant side	438.31	I69.331
Monoplegia of upper limb following cerebral infarction affecting right dominant side	438.31	I69.331
Monoplegia upper limb from cerebral infarction affected right dominant side side	438.31	I69.331
Monoplegia of upper limb following cerebral infarction affecting right dominant side		I69.331

© 2021 Eberly LA et al. *JAMA Network Open*.

Monoplegia of arm after cerebral infarct affecting left dominant side	438.31	I69.332
Monoplegia of upper extremity following cerebral infarction affecting left dominant side	438.31	I69.332
Monoplegia of upper limb following cerebral infarction affecting left dominant side	438.31	I69.332
Monoplegia upper limb from cerebral infarction aff left dominant side	438.31	I69.332
Monoplegia of upper limb following cerebral infarction affecting left dominant side		I69.332
Monoplegia arm after cerebral infarct affect right non-dominant side	438.32	I69.333
Monoplegia of upper extremity following cerebral infarction affecting right non-dominant side	438.32	I69.333
Monoplegia of upper limb following cerebral infarction affecting right non-dominant side	438.32	I69.333
Monoplegia upper limb from cerebral infarction aff right nondominant side	438.32	I69.333
Monoplegia of upper limb following cerebral infarction affecting right non-dominant side		I69.333
Monoplegia of arm after cerebral infarct affect left non-dominant side	438.32	I69.334
Monoplegia of upper extremity following cerebral infarction affecting left non-dominant side	438.32	I69.334
Monoplegia of upper limb following cerebral infarction affecting left non-dominant side	438.32	I69.334

© 2021 Eberly LA et al. *JAMA Network Open*.

Monoplegia upper limb from cerebral infarction aff left nondominant side	438.32	I69.334
Monoplegia of upper limb following cerebral infarction affecting left non-dominant side		I69.334
Monoplegia, upper limb, nondominant side S/P CVA (cerebrovascular acc)	438.32	I69.339
Monoplegia, upper limb, dominant side S/P CVA (cerebrovascular acc)	438.31	I69.339
Monoplegia, upper limb, dominant side S/P CVA	438.31	I69.339
Monoplegia of upper limb due to old cerebral infarction	438.3	I69.339
Monoplegia of upper extremity following cerebral infarction	438.3	I69.339
Upper limb monoplegia of nondominant side status post cerebrovascular accident	438.32	I69.339
Monoplegia, upper limb, nondominant side S/P CVA (cerebrovascular accident)	438.32	I69.339
Upper limb monoplegia of dominant side following cerebrovascular accident	438.31	I69.339
Upper limb monoplegia of dominant side following CVA (cerebrovascular accident)	438.31	I69.339
Monoplegia of upper extremity due to recent cerebral infarction	438.3	I69.339
Monoplegia of upper limb due to recent cerebrovascular accident	438.3	I69.339
Monoplegia of upper extremity of non-dominant side following cerebrovascular accident	438.32	I69.339
Monoplegia of upper extremity due to old cerebral infarction	438.3	I69.339
Monoplegia of upper extremity due to recent cerebrovascular accident	438.3	I69.339

© 2021 Eberly LA et al. *JAMA Network Open*.

Monoplegia of upper extremity of dominant side following cerebrovascular accident	438.31	I69.339
Monoplegia of upper extremity due to recent cerebrovascular accident (CVA)	438.3	I69.339
Monoplegia of upper extremity of dominant side following cerebrovascular accident (CVA)	438.31	I69.339
Monoplegia of upper extremity of non-dominant side following cerebrovascular accident (CVA)	438.32	I69.339
Monoplegia of upper limb following cerebral infarction affecting unspecified side	438.3	I69.339
Monoplegia upper limb following cerebral infarction affecting unspecified side	438.3	I69.339
Monoplegia of upper extremity following cerebral infarction, unspecified laterality	438.3	I69.339
Monoplegia of upper limb following cerebral infarction affecting unspecified side		I69.339
Monoplegia of lower limb following cerebral infarction		I69.34
Monoplegia of leg after cerebral infarct affecting right dominant side	438.41	I69.341
Monoplegia of lower extremity following cerebral infarction affecting right dominant side	438.41	I69.341
Monoplegia of lower limb following cerebral infarction affecting right dominant side	438.41	I69.341
Monoplegia low limb from cerebral infarction aff right dominant side	438.41	I69.341

© 2021 Eberly LA et al. *JAMA Network Open*.

Monoplegia of lower limb following cerebral infarction affecting right dominant side		I69.341
Monoplegia of leg after cerebral infarct affecting left dominant side	438.41	I69.342
Monoplegia of lower extremity following cerebral infarction affecting left dominant side	438.41	I69.342
Monoplegia of lower limb following cerebral infarction affecting left dominant side	438.41	I69.342
Monoplegia low limb from cerebral infarction aff left dominant side	438.41	I69.342
Monoplegia of lower limb following cerebral infarction affecting left dominant side		I69.342
Monoplegia leg after cerebral infarct affect right non-dominant side	438.42	I69.343
Monoplegia of lower extremity following cerebral infarction affecting right non-dominant side	438.42	I69.343
Monoplegia of lower limb following cerebral infarction affecting right non-dominant side	438.42	I69.343
Monoplegia low limb from cerebral infarction aff right nondominant side	438.42	I69.343
Monoplegia of lower limb following cerebral infarction affecting right non-dominant side		I69.343
Monoplegia of leg after cerebral infarct affect left non-dominant side	438.42	I69.344
Monoplegia of lower extremity following cerebral infarction affecting left non-dominant side	438.42	I69.344

© 2021 Eberly LA et al. *JAMA Network Open*.

Monoplegia of lower limb following cerebral infarction affecting left non-dominant side	438.42	I69.344
Monoplegia low limb from cerebral infarction aff left nondominant side	438.42	I69.344
Monoplegia of lower limb following cerebral infarction affecting left non-dominant side		I69.344
Monoplegia, lower extremity, post-stroke	IMO0002	I69.349
Monoplegia, lower limb S/P CVA (cerebrovascular accident)	IMO0002	I69.349
Monoplegia of lower limb following cerebrovascular accident	IMO0002	I69.349
Monoplegia of lower limb following CVA (cerebrovascular accident)	IMO0002	I69.349
Monoplegia, lower limb, dominant side S/P CVA (cerebrovascular acc)	438.41	I69.349
Monoplegia, lower limb, nondominant side S/P CVA (cerebrovascular acc)	438.42	I69.349
Monoplegia, dominant lower extremity, post-stroke	438.41	I69.349
Monoplegia of lower extremity following cerebral infarction	438.4	I69.349
Monoplegia of lower limb due to recent cerebrovascular accident	438.4	I69.349
Monoplegia of lower limb dominant side status post cerebrovascular accident	438.41	I69.349
Monoplegia, lower limb, dominant side S/P CVA (cerebrovascular accident)	438.41	I69.349
Monoplegia of lower limb nondominant side status post cerebrovascular accident	438.42	I69.349
Monoplegia, lower limb, nondominant side S/P CVA (cerebrovascular accident)	438.42	I69.349

© 2021 Eberly LA et al. *JAMA Network Open*.

Monoplegia of lower extremity affecting non-dominant side following cerebrovascular accident	438.42	I69.349
Monoplegia of lower extremity due to recent cerebrovascular accident	438.4	I69.349
Monoplegia of lower extremity following cerebrovascular accident	IMO0002	I69.349
Monoplegia of lower extremity affecting dominant side following cerebrovascular accident	438.41	I69.349
Monoplegia of lower extremity due to recent cerebrovascular accident (CVA)	438.4	I69.349
Monoplegia of lower extremity following cerebrovascular accident (CVA)	IMO0002	I69.349
Monoplegia of lower extremity affecting dominant side following cerebrovascular accident (CVA)	438.41	I69.349
Monoplegia of lower extremity affecting non-dominant side following cerebrovascular accident (CVA)	438.42	I69.349
Monoplegia of lower limb following cerebral infarction affecting unspecified side	438.4	I69.349
Monoplegia of lower extremity affecting dominant side following cerebrovascular accident (CVA)	438.41	I69.349
Monoplegia of lower extremity affecting dominant side following cerebrovascular accident	438.41	I69.349
Monoplegia low limb following cerebral infarction affecting unspecified side	438.4	I69.349
Monoplegia of lower extremity following cerebral infarction, unspecified laterality	438.4	I69.349
Monoplegia of lower limb following cerebral infarction affecting unspecified side		I69.349

© 2021 Eberly LA et al. *JAMA Network Open*.

Hemiplegia and hemiparesis following cerebral infarction		I69.35
Hemiparesis affecting right side as late effect of stroke	438.2	I69.351
Hemiparesis affecting right side as late effect of cerebrovascular accident	438.2	I69.351
Hemiplegia following cerebral infarction aff right dominant side	438.21	I69.351
Hemiparesis affecting right side as late effect of cerebrovascular accident (CVA)	438.2	I69.351
Hemiplegia and hemiparesis following cerebral infarction affecting right dominant side	438.21	I69.351
Hemiplegia of right dominant side due to infarction of brain, unspecified hemiplegia type	438.21, 429.79	I69.351
Hemiplegia and hemiparesis following cerebral infarction affecting right dominant side		I69.351
Hemiplegia following cerebral infarction aff left dominant side	438.21	I69.352
Hemiplegia and hemiparesis following cerebral infarction affecting left dominant side	438.21	I69.352
Hemiplegia of left dominant side due to infarction of brain, unspecified hemiplegia type	438.21	I69.352
Hemiplegia and hemiparesis following cerebral infarction affecting left dominant side		I69.352
Hemiplegia following cerebral infarction aff right nondominant side	438.22	I69.353
Hemiplegia and hemiparesis following cerebral infarction affecting right non-dominant side	438.22	I69.353

© 2021 Eberly LA et al. *JAMA Network Open*.

Hemiplegia of right nondominant side due to infarction of brain, unspecified hemiplegia type	438.22, 429.79	I69.353
Hemiplegia and hemiparesis following cerebral infarction affecting right non-dominant side		I69.353
Hemiparesis affecting left side as late effect of stroke	438.2	I69.354
Hemiparesis affecting left side as late effect of cerebrovascular accident	438.2	I69.354
Hemiplegia following cerebral infarction affecting left nondominant side	438.22	I69.354
Hemiparesis affecting left side as late effect of cerebrovascular accident (CVA)	438.2	I69.354
Hemiplegia and hemiparesis following cerebral infarction affecting left non-dominant side	438.22	I69.354
Hemiplegia and hemiparesis following cerebral infarction affecting left non-dominant side		I69.354
Hemiplegia, dominant side S/P CVA (cerebrovascular accident)	438.21	I69.359
Hemiplegia S/P CVA (cerebrovascular accident)	438.2	I69.359
Hemiplegia of dominant side following cerebrovascular accident	438.21	I69.359
Hemiplegia of dominant side following CVA (cerebrovascular accident)	438.21	I69.359
Hemiplegia following cerebrovascular accident	438.2	I69.359
Hemiplegia following CVA (cerebrovascular accident)	438.2	I69.359
CVA, old, hemiparesis	438.2	I69.359
Hemiparesis following cerebrovascular accident	438.2	I69.359

© 2021 Eberly LA et al. *JAMA Network Open*.

Hemiplegia due to old stroke	438.2	I69.359
Hemiparesis due to old cerebral infarction	438.2	I69.359
Hemiparesis due to old cerebrovascular accident	438.2	I69.359
Hemiparesis due to old stroke	438.2	I69.359
Hemiparesis due to old lacunar stroke	438.2	I69.359
Hemiplegia, post-stroke	438.2	I69.359
Hemiplegia affecting dominant side, post-stroke	438.21	I69.359
Hemiplegia as late effect of cerebrovascular accident	438.2	I69.359
Hemiplegia as late effect of stroke	438.2	I69.359
Dominant hemiplegia complicating stroke	438.21	I69.359
Hemiparesis due to recent cerebrovascular accident	438.2	I69.359
Hemiparesis due to recent stroke	438.2	I69.359
Hemiparesis of dominant side due to recent cerebrovascular accident	438.21	I69.359
Hemiparesis affecting nondominant side as late effect of stroke	438.22	I69.359
Hemiparesis affecting dominant side as late effect of stroke	438.21	I69.359
Hemiparesis affecting dominant side as late effect of cerebrovascular accident	438.21	I69.359
Hemiparesis affecting nondominant side as late effect of cerebrovascular accident	438.22	I69.359
Hemiparesis due to recent cerebral infarction	438.2	I69.359
Multiple old cerebral infarcts with hemiparesis	438.2	I69.359
Hemiplegia due to recent cerebrovascular accident	438.2	I69.359
Hemiplegia due to recent stroke	438.2	I69.359

© 2021 Eberly LA et al. *JAMA Network Open*.

Hemiparesis due to old brainstem infarction	438.2	I69.359
Hemiplegia due to old brainstem infarction	438.2	I69.359
Hemiparesis due to recent cerebrovascular accident (CVA)	438.2	I69.359
Hemiplegia due to recent cerebrovascular accident (CVA)	438.2	I69.359
Hemiparesis of dominant side due to recent cerebrovascular accident (CVA)	438.21	I69.359
Hemiparesis following cerebrovascular accident (CVA)	438.2	I69.359
Hemiplegia following cerebrovascular accident (CVA)	438.2	I69.359
Hemiplegia of dominant side following cerebrovascular accident (CVA)	438.21	I69.359
Hemiplegia as late effect of cerebrovascular accident (CVA)	438.2	I69.359
Hemiparesis as late effect of cerebrovascular accident (CVA)	438.2	I69.359
Hemiparesis affecting dominant side as late effect of cerebrovascular accident (CVA)	438.21	I69.359
Hemiparesis affecting nondominant side as late effect of cerebrovascular accident (CVA)	438.22	I69.359
Hemiplegia and hemiparesis following cerebral infarction affecting unspecified side	438.2	I69.359
Hemiplegia following cerebral infarction affecting unspecified side	438.2	I69.359
Hemiplegia and hemiparesis following cerebral infarction affecting unspecified side		I69.359
Hemiparesis and aphasia as late effects of cerebrovascular accident	438.20, 438.11	I69.359, I69.320

© 2021 Eberly LA et al. *JAMA Network Open*.

Hemiparesis and aphasia as late effect of cerebrovascular accident (CVA)	438.20, 438.11	I69.359, I69.320
Hemiparesis and dysphasia as late effects of cerebrovascular accident	438.20, 438.12	I69.359, I69.321
Hemiparesis and dysphasia as late effect of cerebrovascular accident (CVA)	438.20, 438.12	I69.359, I69.321
Hemiparesis and speech and language deficit as late effects of stroke	438.20, 438.10	I69.359, I69.328
Hemiparesis and speech and language deficit as late effects of cerebrovascular accident	438.20, 438.10	I69.359, I69.328
Hemiparesis and speech and language deficit as late effect of cerebrovascular accident (CVA)	438.20, 438.10	I69.359, I69.328
Hemiparesis, speech and language deficits, and cognitive deficits due to recent cerebral infarction	438.0, 438.10, 438.20	I69.359, I69.328, I69.319
Speech, language, and cognitive deficits, with hemiparesis, due to recent cerebral infarction	438.0, 438.20, 438.10	I69.359, I69.328, I69.319
Hemiparesis w/speech, language, & cognitive deficits from recent cerebral infarct	438.0, 438.20, 438.10	I69.359, I69.328, I69.319
Hemiparesis and other late effects of cerebrovascular accident	438.20, 438.89	I69.359, I69.398
Hemiparesis and alteration of sensations as late effects of stroke	438.20, 438.6	I69.359, I69.398
Hemiparesis and alteration of sensations as late effects of cerebrovascular accident	438.20, 438.6	I69.359, I69.398
Hemiparesis and alteration of sensations as late effect of cerebrovascular accident (CVA)	438.20, 438.6	I69.359, I69.398
Other paralytic syndrome following cerebral infarction		I69.36
Other parlytic syndrome from cerebral infarction aff right dominant side	438.51	I69.361

© 2021 Eberly LA et al. *JAMA Network Open*.

Other paralytic syndrome following cerebral infarction affecting right dominant side	438.51	I69.361
Other paralytic syndrome following cerebral infarction affecting right dominant side		I69.361
Oth parlyt syndrome fol cereb infrc aff left dominant side	438.51	I69.362
Other paralytic syndrome following cerebral infarction affecting left dominant side	438.51	I69.362
Other paralytic syndrome following cerebral infarction affecting left dominant side		I69.362
Oth parlyt syndrome fol cerebral infrc aff right nondom side	438.52	I69.363
Other paralytic syndrome following cerebral infarction affecting right non-dominant side	438.52	I69.363
Other paralytic syndrome following cerebral infarction affecting right non-dominant side		I69.363
Oth parlyt syndrome fol cerebral infrc aff left nondom side	438.52	I69.364
Other paralytic syndrome following cerebral infarction affecting left non-dominant side	438.52	I69.364
Other paralytic syndrome following cerebral infarction affecting left non-dominant side		I69.364
Paralytic syndrome, bilateral S/P CVA (cerebrovascular accident)	438.53	I69.365
Bilateral paralytic syndrome following cerebrovascular accident	438.53	I69.365

© 2021 Eberly LA et al. *JAMA Network Open*.

Bilateral paralytic syndrome following CVA (cerebrovascular accident)	438.53	I69.365
Paralytic syndrome, bilateral, post-stroke	438.53	I69.365
Bilateral paralytic syndrome following stroke	438.53	I69.365
Bilateral paralytic syndrome following cerebrovascular accident (CVA)	438.53	I69.365
Other paralytic syndrome following cerebral infarction, bilateral	438.53	I69.365
Bilateral paralytic syndrome as late effect of stroke	438.53	I69.365
Bilateral paralytic syndrome as late effect of cerebrovascular accident (CVA)	438.53	I69.365
Other paralytic syndrome following cerebral infarction, bilateral		I69.365
Paralytic syndrome, post-stroke	438.5	I69.369
Paralytic syndrome dominant side S/P CVA (cerebrovascular accident)	438.51	I69.369
Paralytic syndrome nondominant side S/P CVA (cerebrovascular accident)	438.52	I69.369
Paralytic syndrome S/P CVA (cerebrovascular accident)	438.5	I69.369
Paralytic syndrome following cerebrovascular accident	438.5	I69.369
Paralytic syndrome following CVA (cerebrovascular accident)	438.5	I69.369
Paralytic syndrome of dominant side following cerebrovascular accident	438.51	I69.369
Paralytic syndrome, dominant side, post-stroke	438.51	I69.369
Paralytic syndrome, non-dominant side, post-stroke	438.52	I69.369
Paralysis of dominant side as complication of stroke	438.51	I69.369
Paralytic syndrome of nondominant side following cerebrovascular accident	438.52	I69.369

© 2021 Eberly LA et al. *JAMA Network Open*.

Paralytic syndrome affecting dominant side following cerebrovascular accident	438.51	I69.369
Paralytic syndrome of nondominant side as late effect of stroke	438.52	I69.369
Paralytic syndrome of dominant side as late effect of stroke	438.51	I69.369
Paralytic syndrome as late effect of stroke	438.5	I69.369
Paralytic syndrome affecting dominant side	438.51	I69.369
Paralytic syndrome affecting dominant side following cerebrovascular accident (CVA)	438.51	I69.369
Paralytic syndrome following cerebrovascular accident (CVA)	438.5	I69.369
Paralytic syndrome of non-dominant side following cerebrovascular accident (CVA)	438.52	I69.369
Other paralytic syndrome following cerebral infarction affecting unspecified side	438.5	I69.369
Other paralytic syndrome following cerebral infarction	438.5	I69.369
Oth paralytic syndrome fol cerebral infrc aff unsp side	438.5	I69.369
Other paralytic syndrome following cerebral infarction affecting unspecified side		I69.369
Other sequelae of cerebral infarction		I69.39
Apraxia S/P CVA (cerebrovascular accident)	438.81	I69.390
Apraxia following cerebrovascular accident	438.81	I69.390
Apraxia following CVA (cerebrovascular accident)	438.81	I69.390
Apraxia due to old lacunar stroke	438.81	I69.390
Apraxia, post-stroke	438.81	I69.390

© 2021 Eberly LA et al. *JAMA Network Open*.

Apraxia following cerebral infarction	438.81	I69.390
Apraxia due to recent cerebral infarction	438.81	I69.390
Apraxia due to recent cerebrovascular accident	438.81	I69.390
Apraxia due to old brainstem infarction	438.81	I69.390
Apraxia due to recent cerebrovascular accident (CVA)	438.81	I69.390
Apraxia following cerebrovascular accident (CVA)	438.81	I69.390
Apraxia as late effect of cerebrovascular accident (CVA)	438.81	I69.390
Apraxia following cerebral infarction		I69.390
Dysphagia status post cerebrovascular accident	438.82	I69.391
Dysphagia S/P CVA (cerebrovascular accident)	438.82	I69.391
CVA, old, dysphagia	438.82	I69.391
Dysphagia following cerebrovascular accident	438.82	I69.391
Dysphagia due to old stroke	438.82	I69.391
Dysphagia, post-stroke	438.82	I69.391
Dysphagia following cerebral infarction	438.82	I69.391
Dysphagia as late effect of stroke	438.82	I69.391
Dysphagia due to recent cerebrovascular accident	438.82	I69.391
Dysphagia due to recent stroke	438.82	I69.391
Dysphagia due to recent cerebral infarction	438.82	I69.391
Dysphagia due to old cerebrovascular accident	438.82	I69.391
Dysphagia due to recent cerebrovascular accident (CVA)	438.82	I69.391
Dysphagia following cerebrovascular accident (CVA)	438.82	I69.391

© 2021 Eberly LA et al. *JAMA Network Open*.

Dysphagia as late effect of cerebrovascular accident (CVA)	438.82	I69.391
Dysphagia following cerebral infarction		I69.391
Hemiparesis, aphasia, and dysphagia as late effect of cerebrovascular accident (CVA)	438.82, 438.20, 438.11	I69.391, I69.320, I69.359
Hemiparesis, aphasia, and dysphagia as late effects of stroke	438.82, 438.20, 438.11	I69.391, I69.359, I69.320
Hemiparesis, aphasia, and dysphagia as late effects of cerebrovascular accident	438.82, 438.20, 438.11	I69.391, I69.359, I69.320
Facial weakness status post cerebrovascular accident	438.83	I69.392
Facial weakness S/P CVA (cerebrovascular accident)	438.83	I69.392
CVA, old, facial weakness	438.83	I69.392
Facial weakness due to old brainstem infarction	438.83	I69.392
Facial weakness due to old cerebrovascular accident	438.83	I69.392
Facial weakness due to old stroke	438.83	I69.392
Facial weakness due to old lacunar stroke	438.83	I69.392
Facial weakness, post-stroke	438.83	I69.392
Facial weakness following cerebral infarction	438.83	I69.392
Facial weakness due to recent cerebrovascular accident	438.83	I69.392
Facial weakness due to recent stroke	438.83	I69.392
Facial weakness due to recent cerebral infarction	438.83	I69.392
Facial weakness due to recent cerebrovascular accident (CVA)	438.83	I69.392
Facial weakness following cerebrovascular accident (CVA)	438.83	I69.392

© 2021 Eberly LA et al. *JAMA Network Open*.

Facial weakness as late effect of cerebrovascular accident (CVA)	438.83	I69.392
Facial weakness following cerebral infarction		I69.392
Ataxia due to old cerebellar infarction	438.84	I69.393
Ataxia due to old cerebral infarction	438.84	I69.393
Ataxia due to old cerebrovascular accident	438.84	I69.393
Ataxia due to old stroke	438.84	I69.393
Ataxia due to old lacunar stroke	438.84	I69.393
Ataxia, post-stroke	438.84	I69.393
Ataxia following cerebral infarction	438.84	I69.393
Muscular incoordination due to old stroke	438.84	I69.393
Ataxia due to recent stroke	438.84	I69.393
Ataxia due to recent cerebrovascular accident	438.84	I69.393
Ataxia due to recent cerebral infarction	438.84	I69.393
Multiple old cerebral infarcts with ataxia	438.84	I69.393
Ataxia due to old cerebrovascular accident (CVA)	438.84	I69.393
Ataxia due to recent cerebrovascular accident (CVA)	438.84	I69.393
Ataxia following cerebral infarction		I69.393
Other late effect of cerebrovascular accident	438.89	I69.398
Other late effect of recent cerebral infarction	438.89	I69.398
Other sequelae of cerebral infarction	438.89	I69.398
Other sequelae of cerebral infarction		I69.398
Depression due to old stroke	438.89, 293.83	I69.398, F06.31
Depression as late effect of cerebrovascular accident (CVA)	438.89, 293.83	I69.398, F06.31
Neurobehavioral disorder following cerebrovascular accident	438.89, 310.9	I69.398, F09

© 2021 Eberly LA et al. *JAMA Network Open*.

Neurobehavioral disorder following stroke	438.89, 310.9	I69.398, F09
Neurobehavioral disorder following cerebrovascular accident (CVA)	438.89, 310.9	I69.398, F09
Seizure disorder as sequela of cerebrovascular accident	438.89, 345.90	I69.398, G40.909
Central sleep apnea secondary to cerebrovascular accident	438.89, 327.27	I69.398, G47.37
Central sleep apnea secondary to cerebrovascular accident (CVA)	438.89, 327.27	I69.398, G47.37
CVA, old, homonymous hemianopsia	438.7, 368.46	I69.398, H53.469
Homonymous hemianopsia following cerebrovascular accident	438.7, 368.46	I69.398, H53.469
Homonymous hemianopsia due to old cerebral infarction	438.7, 368.46	I69.398, H53.469
Multiple old cerebral infarcts with homonymous hemianopsia	438.7, 368.46	I69.398, H53.469
Homonymous hemianopsia due to recent cerebrovascular accident	438.7, 368.46	I69.398, H53.469
Homonymous hemianopsia due to recent stroke	438.7, 368.46	I69.398, H53.469
Homonymous hemianopsia due to recent cerebral infarction	438.7, 368.46	I69.398, H53.469
Homonymous hemianopsia due to recent cerebrovascular accident (CVA)	438.7, 368.46	I69.398, H53.469
Homonymous hemianopsia following cerebrovascular accident (CVA)	438.7, 368.46	I69.398, H53.469
Vision disturbance S/P CVA (cerebrovascular accident)	438.7	I69.398, H53.9
Vision disturbance following cerebrovascular accident	438.7	I69.398, H53.9
Vision disturbance following CVA (cerebrovascular accident)	438.7	I69.398, H53.9
CVA, old, disturbances of vision	438.7	I69.398, H53.9
Visual disturbance due to old lacunar stroke	438.7	I69.398, H53.9

© 2021 Eberly LA et al. *JAMA Network Open*.

Visual disturbance as complication of stroke	438.7	I69.398, H53.9
Visual disturbance due to recent cerebrovascular accident	438.7	I69.398, H53.9
Visual disturbance due to recent stroke	438.7	I69.398, H53.9
Disturbances of vision, late effect of stroke	438.7	I69.398, H53.9
Visual disturbance due to recent cerebral infarction	438.7	I69.398, H53.9
Visual disturbance due to recent cerebrovascular accident (CVA)	438.7	I69.398, H53.9
Visual disturbance following cerebrovascular accident (CVA)	438.7	I69.398, H53.9
Visual disturbance as late effect of cerebrovascular accident (CVA)	438.7	I69.398, H53.9
Visual field loss following cerebrovascular accident	438.7	I69.398, H54.7
Visual field loss, post-stroke	438.7	I69.398, H54.7
Visual field loss following stroke	438.7	I69.398, H54.7
Visual field loss following cerebrovascular accident (CVA)	438.7	I69.398, H54.7
Neurogenic pain due to central nervous system abnormality following stroke	438.89, 729.2	I69.398, M79.2
Alterations of sensations S/P CVA (cerebrovascular accident)	438.6	I69.398, R20.9
Alterations of sensations following cerebrovascular accident	438.6	I69.398, R20.9
Alterations of sensations following CVA (cerebrovascular accident)	438.6	I69.398, R20.9
CVA, old, alterations of sensations	438.6	I69.398, R20.9
Altered sensation due to old lacunar stroke	438.6	I69.398, R20.9
Alteration of sensations, post-stroke	438.6	I69.398, R20.9
Altered sensation due to recent cerebral infarction	438.6	I69.398, R20.9

© 2021 Eberly LA et al. *JAMA Network Open*.

Altered sensation due to recent stroke	438.6	I69.398, R20.9
Alteration of sensation as late effect of stroke	438.6	I69.398, R20.9
Alteration of sensation as late effect of cerebrovascular accident	438.6	I69.398, R20.9
Alterations of sensations following cerebrovascular accident (CVA)	438.6	I69.398, R20.9
Alteration of sensation as late effect of cerebrovascular accident (CVA)	438.6	I69.398, R20.9
Impaired balance as late effect of cerebrovascular accident	438.89, 781.2	I69.398, R26.89
Imbalance due to old stroke	438.89, 781.2	I69.398, R26.89
Impaired balance as late effect of cerebrovascular accident (CVA)	438.89, 781.2	I69.398, R26.89
Abnormality of gait following cerebrovascular accident	438.89, 781.2	I69.398, R26.9
Gait disturbance, post-stroke	438.89, 781.2	I69.398, R26.9
Abnormality of gait following cerebrovascular accident (CVA)	438.89, 781.2	I69.398, R26.9
Abnormality of gait as late effect of cerebrovascular accident (CVA)	438.89	I69.398, R26.9
Abnormality of gait as late effect of stroke	438.89	I69.398, R26.9
Vertigo as late effect of stroke	438.85	I69.398, R42
Vertigo S/P CVA (cerebrovascular accident)	438.85	I69.398, R42
Vertigo following cerebrovascular accident	438.85	I69.398, R42
Vertigo following CVA (cerebrovascular accident)	438.85	I69.398, R42
Vertigo due to previous cerebellar infarction	438.85	I69.398, R42
Vertigo, post-stroke	438.85	I69.398, R42
Vertigo following cerebrovascular accident (CVA)	438.85	I69.398, R42

© 2021 Eberly LA et al. *JAMA Network Open*.

Pain after cerebrovascular accident (CVA)	438.89, 780.96	I69.398, R52
Weakness status post cerebrovascular accident	438.89, 780.79	I69.398, R53.1
Weakness S/P CVA (cerebrovascular accident)	438.89, 780.79	I69.398, R53.1
Weakness following cerebrovascular accident (CVA)	438.89, 780.79	I69.398, R53.1
Seizure as late effect of cerebrovascular accident (CVA)	438.89, 780.39	I69.398, R56.9
Seizure, late effect of stroke	438.89, 780.39	I69.398, R56.9
Sequelae of other cerebrovascular diseases		I69.8
Unspecified sequelae of other cerebrovascular disease	438.9	I69.80
Unspecified sequelae of other cerebrovascular disease		I69.80
Cognitive deficits following other cerebrovascular disease		I69.81
Attention and concentration deficit following other cerebrovascular disease	438	I69.810
Attention and concentration deficit following other cerebrovascular disease		I69.810
Memory deficit following other cerebrovascular disease	438.0, 780.93	I69.811
Memory deficit following other cerebrovascular disease		I69.811
Visuospatial deficit and spatial neglect following other cerebrovascular disease	438.89, 799.53, 781.8	I69.812
Visuospatial deficit and spatial neglect following other cerebrovascular disease		I69.812
Psychomotor deficit following other cerebrovascular disease	438.89, 799.54	I69.813
Psychomotor deficit following other cerebrovascular disease		I69.813

© 2021 Eberly LA et al. *JAMA Network Open*.

Frontal lobe and executive function deficit following other cerebrovascular disease	438	I69.814
Frontal lobe and executive function deficit following other cerebrovascular disease		I69.814
Cognitive social or emotional deficit following other cerebrovascular disease	438	I69.815
Cognitive social or emotional deficit following other cerebrovascular disease		I69.815
Other symptoms and signs involving cognitive functions following other cerebrovascular disease	799.59	I69.818
Other symptoms and signs involving cognitive functions following other cerebrovascular disease		I69.818
Cognitive deficits following other cerebrovascular disease	438	I69.819
Unspecified symptoms and signs involving cognitive functions following other cerebrovascular disease	438	I69.819
Unspecified symptoms and signs involving cognitive functions following other cerebrovascular disease		I69.819
Speech and language deficits following other cerebrovascular disease		I69.82
Aphasia following other cerebrovascular disease	438.11	I69.820
Aphasia following other cerebrovascular disease		I69.820
Dysphasia following other cerebrovascular disease	438.12	I69.821
Dysphasia following other cerebrovascular disease		I69.821
Dysarthria following other cerebrovascular disease	438.13	I69.822

© 2021 Eberly LA et al. *JAMA Network Open*.

Dysarthria following other cerebrovascular disease		I69.822
Fluency disorder following other cerebrovascular disease	438.14	I69.823
Fluency disorder following other cerebrovascular disease		I69.823
Oth speech/lang deficits following oth cerebvasc disease	438.19	I69.828
Other speech and language deficits following other cerebrovascular disease	438.19	I69.828
Other speech and language deficits following other cerebrovascular disease		I69.828
Monoplegia of upper limb following other cerebrovascular disease		I69.83
Monoplg upr lmb fol oth cerebvasc disease aff right dom side	438.31	I69.831
Monoplegia of upper limb following other cerebrovascular disease affecting right dominant side	438.31	I69.831
Monoplegia of upper extremity following other cerebrovascular disease affecting right dominant side	438.31	I69.831
Monoplegia of upper limb following other cerebrovascular disease affecting right dominant side		I69.831
Monoplg upr lmb fol oth cerebvasc disease aff left dom side	438.31	I69.832
Monoplegia of upper limb following other cerebrovascular disease affecting left dominant side	438.31	I69.832
Monoplegia of upper extremity following other cerebrovascular disease affecting left dominant side	438.31	I69.832
Monoplegia of upper limb following other cerebrovascular disease affecting left dominant side		I69.832

© 2021 Eberly LA et al. *JAMA Network Open*.

Monoplg upr lmb fol oth cerebvasc dis aff right nondom side	438.32	I69.833
Monoplegia of upper limb following other cerebrovascular disease affecting right non-dominant side	438.32	I69.833
Monoplegia of upper extremity following other cerebrovascular disease affecting right non-dominant side	438.32	I69.833
Monoplegia of upper limb following other cerebrovascular disease affecting right non-dominant side		I69.833
Monoplg upr lmb fol oth cerebvasc dis aff left nondom side	438.32	I69.834
Monoplegia of upper limb following other cerebrovascular disease affecting left non-dominant side	438.32	I69.834
Monoplegia of upper extremity following other cerebrovascular disease affecting left non-dominant side	438.32	I69.834
Monoplegia of upper limb following other cerebrovascular disease affecting left non-dominant side		I69.834
Monoplg upr lmb fol oth cerebvasc disease aff unsp side	438.3	I69.839
Monoplegia of upper limb following other cerebrovascular disease affecting unspecified side	438.3	I69.839
Monoplegia of upper extremity following other cerebrovascular disease, unspecified laterality	438.3	I69.839
Monoplegia of upper limb following other cerebrovascular disease affecting unspecified side		I69.839

© 2021 Eberly LA et al. *JAMA Network Open*.

Monoplegia of lower limb following other cerebrovascular disease		I69.84
Monoplg low lmb fol oth cerebvasc disease aff right dom side	438.41	I69.841
Monoplegia of lower limb following other cerebrovascular disease affecting right dominant side	438.41	I69.841
Monoplegia of lower extremity following other cerebrovascular disease affecting right dominant side	438.41	I69.841
Monoplegia of lower limb following other cerebrovascular disease affecting right dominant side		I69.841
Monoplg low lmb fol oth cerebvasc disease aff left dom side	438.41	I69.842
Monoplegia of lower limb following other cerebrovascular disease affecting left dominant side	438.41	I69.842
Monoplegia of lower extremity following other cerebrovascular disease affecting left dominant side	438.41	I69.842
Monoplegia of lower limb following other cerebrovascular disease affecting left dominant side		I69.842
Monoplg low lmb fol oth cerebvasc dis aff right nondom side	438.42	I69.843
Monoplegia of lower limb following other cerebrovascular disease affecting right non-dominant side	438.42	I69.843
Monoplegia of lower extremity following other cerebrovascular disease affecting right non-dominant side	438.42	I69.843
Monoplegia of lower limb following other cerebrovascular disease affecting right non-dominant side		I69.843

© 2021 Eberly LA et al. *JAMA Network Open*.

Monoplg low lmb fol oth cerebvasc dis aff left nondom side	438.42	I69.844
Monoplegia of lower limb following other cerebrovascular disease affecting left non-dominant side	438.42	I69.844
Monoplegia of lower extremity following other cerebrovascular disease affecting left non-dominant side	438.42	I69.844
Monoplegia of lower limb following other cerebrovascular disease affecting left non-dominant side		I69.844
Monoplg low lmb fol oth cerebvasc disease aff unsp side	438.4	I69.849
Monoplegia of lower limb following other cerebrovascular disease affecting unspecified side	438.4	I69.849
Monoplegia of lower extremity following other cerebrovascular disease, unspecified laterality	438.4	I69.849
Monoplegia of lower limb following other cerebrovascular disease affecting unspecified side		I69.849
Hemiplegia and hemiparesis following other cerebrovascular disease		I69.85
Hemiplegia and hemiparesis following other cerebrovascular disease affecting right dominant side	438.21	I69.851
Hemiplga fol oth cerebvasc disease aff right dominant side	438.21	I69.851
Hemiplegia of right dominant side due to other cerebrovascular disease, unspecified hemiplegia type	438.21	I69.851
Hemiplegia and hemiparesis following other cerebrovascular disease affecting right dominant side		I69.851

© 2021 Eberly LA et al. *JAMA Network Open*.

Hemiplga fol oth cerebvasc disease aff left dominant side	438.21	I69.852
Hemiplegia and hemiparesis following other cerebrovascular disease affecting left dominant side	438.21	I69.852
Hemiplegia of left dominant side due to other cerebrovascular disease, unspecified hemiplegia type	438.21	I69.852
Hemiplegia and hemiparesis following other cerebrovascular disease affecting left dominant side		I69.852
Hemiplga fol oth cerebvasc disease aff right nondom side	438.22	I69.853
Hemiplegia and hemiparesis following other cerebrovascular disease affecting right non-dominant side	438.22	I69.853
Hemiplegia of right nondominant side due to other cerebrovascular disease, unspecified hemiplegia type	438.22	I69.853
Hemiplegia and hemiparesis following other cerebrovascular disease affecting right non-dominant side		I69.853
Hemiplegia and hemiparesis following other cerebrovascular disease affecting left non-dominant side	438.22	I69.854
Hemiplga fol oth cerebvasc disease aff left nondom side	438.22	I69.854
Hemiplegia of left nondominant side due to other cerebrovascular disease, unspecified hemiplegia type	438.22	I69.854
Hemiplegia and hemiparesis following other cerebrovascular disease affecting left non-dominant side		I69.854

© 2021 Eberly LA et al. *JAMA Network Open*.

Hemiparesis as late effect of cerebral aneurysm	438.2	I69.859
Hemiplegia and hemiparesis following other cerebrovascular disease affecting unspecified side	438.2	I69.859
Hemiplga following oth cerebvasc disease affecting unsp side	438.2	I69.859
Spastic hemiplegia due to other cerebrovascular disease, unspecified hemiplegia laterality	438.20, 436	I69.859
Hemiplegia and hemiparesis following other cerebrovascular disease affecting unspecified side		I69.859
Other paralytic syndrome following other cerebrovascular disease		I69.86
Oth parlyt synd fol oth cerebvasc disease aff right dom side	438.51	I69.861
Other paralytic syndrome following other cerebrovascular disease affecting right dominant side	438.51	I69.861
Other paralytic syndrome following other cerebrovascular disease affecting right dominant side		I69.861
Oth parlyt synd fol oth cerebvasc disease aff left dom side	438.51	I69.862
Other paralytic syndrome following other cerebrovascular disease affecting left dominant side	438.51	I69.862
Other paralytic syndrome following other cerebrovascular disease affecting left dominant side		I69.862
Oth parlyt synd fol oth cerebvasc dis aff right nondom side	438.52	I69.863

© 2021 Eberly LA et al. *JAMA Network Open*.

Other paralytic syndrome following other cerebrovascular disease affecting right non-dominant side	438.52	I69.863
Other paralytic syndrome following other cerebrovascular disease affecting right non-dominant side		I69.863
Oth parlyt synd fol oth cerebvasc dis aff left nondom side	438.52	I69.864
Other paralytic syndrome following other cerebrovascular disease affecting left non-dominant side	438.52	I69.864
Other paralytic syndrome following other cerebrovascular disease affecting left non-dominant side		I69.864
Oth paralytic syndrome following oth cerebvasc disease, bi	438.53	I69.865
Other paralytic syndrome following other cerebrovascular disease, bilateral	438.53	I69.865
Other paralytic syndrome following other cerebrovascular disease, bilateral		I69.865
Oth parlyt syndrome fol oth cerebvasc disease aff unsp side	438.5	I69.869
Other paralytic syndrome following other cerebrovascular disease affecting unspecified side	438.5	I69.869
Other paralytic syndrome following other cerebrovascular disease	438.5	I69.869
Other paralytic syndrome following other cerebrovascular disease affecting unspecified side		I69.869
Other sequelae of other cerebrovascular disease		I69.89
Apraxia following other cerebrovascular disease	438.81	I69.890

© 2021 Eberly LA et al. *JAMA Network Open*.

Apraxia following other cerebrovascular disease		I69.890
Dysphagia following other cerebrovascular disease	438.82	I69.891
Dysphagia following other cerebrovascular disease		I69.891
Facial weakness following other cerebrovascular disease	438.83	I69.892
Facial weakness following other cerebrovascular disease		I69.892
Ataxia following other cerebrovascular disease	438.84	I69.893
Ataxia following other cerebrovascular disease		I69.893
History of lateralizing motor deficit following cerebrovascular accident	438.89	I69.898
History of lateralizing motor deficit following cerebrovascular accident (CVA)	438.89	I69.898
Other sequelae of other cerebrovascular disease	438.89	I69.898
Other sequelae of other cerebrovascular disease		I69.898
Alterations of sensations, late effect of cerebrovascular disease	438.6, 782.0	I69.898, R20.8
Multiple old cerebral infarcts with alterations of sensation	438.6, 782.0	I69.898, R20.9
Sequelae of unspecified cerebrovascular diseases		I69.9
Unspecified late effects of cerebrovascular disease	438.9	I69.90
Late effects of cerebrovascular disease	438.9	I69.90
Late, effect, cerebrovascular disease	438.9	I69.90
Sequelae of cerebrovascular disease	438.9	I69.90
Late effects of CVA (cerebrovascular accident)	438.9	I69.90

© 2021 Eberly LA et al. *JAMA Network Open*.

Personal history of cerebrovascular accident with residual effects	438.9	I69.90
History of cerebrovascular accident with residual effects	438.9	I69.90
Cerebrovascular accident, late effects	438.9	I69.90
Late effects of cerebrovascular accident	438.9	I69.90
History of cerebrovascular accident with current residual effects	438.9	I69.90
Personal history of cerebrovascular accident with current residual effects	438.9	I69.90
Unspecified sequelae of unspecified cerebrovascular disease	438.9	I69.90
Unspecified sequelae of unspecified cerebrovascular disease		I69.90
Cognitive deficits following unspecified cerebrovascular disease		I69.91
Attention and concentration deficit following unspecified cerebrovascular disease	438	I69.910
Attention and concentration deficit following unspecified cerebrovascular disease		I69.910
Memory deficit after cerebrovascular disease	438.0, 780.93	I69.911
Memory deficit following unspecified cerebrovascular disease	438.0, 780.93	I69.911
Memory deficit following unspecified cerebrovascular disease		I69.911
Visuospatial deficit and spatial neglect after cerebrovascular disease	438.7, 799.53, 781.8	I69.912
Visuospatial deficit and spatial neglect following unspecified cerebrovascular disease	438.7, 799.53, 781.8	I69.912
Visuospatial deficit and spatial neglect following unspecified cerebrovascular disease		I69.912

© 2021 Eberly LA et al. *JAMA Network Open*.

Psychomotor deficit after cerebrovascular disease	438.89, 799.54	I69.913
Psychomotor deficit following unspecified cerebrovascular disease	438.89, 799.54	I69.913
Psychomotor deficit following unspecified cerebrovascular disease		I69.913
Frontal lobe and executive function deficit following unspecified cerebrovascular disease	438	I69.914
Frontal lobe and executive function deficit following unspecified cerebrovascular disease		I69.914
Cognitive social or emotional deficit following unspecified cerebrovascular disease	438	I69.915
Cognitive social or emotional deficit following unspecified cerebrovascular disease		I69.915
Other symptoms and signs involving cognitive functions following unspecified cerebrovascular disease	799.59	I69.918
Other symptoms and signs involving cognitive functions following unspecified cerebrovascular disease		I69.918
Cognitive deficits, late effect of cerebrovascular disease	438	I69.919
Cognitive deficits following cerebrovascular disease	438	I69.919
Cognitive deficits as late effect of cerebrovascular disease	438	I69.919
Cognitive deficits following unspecified cerebrovascular disease	438	I69.919
Unspecified symptoms and signs involving cognitive functions following unspecified cerebrovascular disease	438	I69.919

© 2021 Eberly LA et al. *JAMA Network Open*.

Unspecified symptoms and signs involving cognitive functions following unspecified cerebrovascular disease		I69.919
Speech and language deficits following unspecified cerebrovascular disease		I69.92
Aphasia, late effect of cerebrovascular disease	438.11	I69.920
Aphasia due to late effects of cerebrovascular disease	438.11	I69.920
Aphasia following cerebrovascular disease	438.11	I69.920
Aphasia following unspecified cerebrovascular disease	438.11	I69.920
Aphasia following unspecified cerebrovascular disease		I69.920
Dysphasia, late effect of cerebrovascular disease	438.12	I69.921
Dysphasia due to cerebrovascular disease	438.12	I69.921
Dysphasia following cerebrovascular disease	438.12	I69.921
Dysphasia as late effect of cerebrovascular disease	438.12	I69.921
Dysphasia following unspecified cerebrovascular disease	438.12	I69.921
Dysphasia following unspecified cerebrovascular disease		I69.921
Dysarthria as late effect of cerebrovascular disease	438.13	I69.922
Late effects of cerebrovascular disease, dysarthria	438.13	I69.922
Dysarthria following cerebrovascular disease	438.13	I69.922
Dysarthria following unspecified cerebrovascular disease	438.13	I69.922
Dysarthria following unspecified cerebrovascular disease		I69.922

© 2021 Eberly LA et al. *JAMA Network Open*.

Fluency disorder as late effect of cerebrovascular disease	438.14	I69.923
Stuttering as late effect of cerebrovascular disease	438.14	I69.923
Late effects of cerebrovascular disease, fluency disorder	438.14	I69.923
Stuttering due to late effect of cerebrovascular disease	438.14	I69.923
Fluency disorder due to late effect of cerebrovascular accident	438.14	I69.923
Fluency disorder following cerebrovascular accident	438.14	I69.923
Fluency disorder following cerebrovascular disease	438.14	I69.923
Fluency disorder following unspecified cerebrovascular disease	438.14	I69.923
Fluency disorder following unspecified cerebrovascular disease		I69.923
Speech and language deficit, unspecified, late effect of cerebrovascular disease	438.1	I69.928
Other speech and language deficits, late effect of cerebrovascular disease(438.19)	438.19	I69.928
Speech and language deficits, late effect of cerebrovascular disease	438.1	I69.928
Speech and language deficit, late effect of cerebrovascular disease	438.1	I69.928
Late ef-spch/lng def NOS	438.1	I69.928
Other speech and language deficits following unspecified cerebrovascular disease	438.19	I69.928
Other speech and language deficits, late effect of cerebrovascular disease	438.19	I69.928
Oth speech/lang deficits following unsp cerebvasc disease	438.19	I69.928

© 2021 Eberly LA et al. *JAMA Network Open*.

Other speech and language deficits following unspecified cerebrovascular disease		I69.928
Monoplegia of upper limb following unspecified cerebrovascular disease		I69.93
Monoplegia of arm after cerebrovasc disease affect right dominant side	438.31	I69.931
Monoplegia of upper extremity following cerebrovascular disease affecting right dominant side	438.31	I69.931
Monoplegia of upper limb following unspecified cerebrovascular disease affecting right dominant side	438.31	I69.931
Monoplg upr lmb fol unsp cerebvasc dis aff right dom side	438.31	I69.931
Monoplegia of upper extremity following cerebrovascular disease affecting right dominant side, unspecified cerebrovascular disease type	438.31	I69.931
Monoplegia of upper limb following unspecified cerebrovascular disease affecting right dominant side		I69.931
Monoplegia of arm after cerebrovasc disease affect left dominant side	438.31	I69.932
Monoplegia of upper extremity following cerebrovascular disease affecting left dominant side	438.31	I69.932
Monoplegia of upper limb following unspecified cerebrovascular disease affecting left dominant side	438.31	I69.932
Monoplg upr lmb fol unsp cerebvasc disease aff left dom side	438.31	I69.932
Monoplegia of upper extremity following cerebrovascular disease affecting left	438.31	I69.932

dominant side, unspecified cerebrovascular disease type		
Monoplegia of upper limb following unspecified cerebrovascular disease affecting left dominant side		I69.932
Monoplegia of upper extremity following cerebrovascular disease affecting right non-dominant side	438.32	I69.933
Monoplegia of upper limb following unspecified cerebrovascular disease affecting right non-dominant side	438.32	I69.933
Monoplg upr lmb fol unsp cerebvasc dis aff right nondom side	438.32	I69.933
Monoplegia of upper extremity following cerebrovascular disease affecting right non-dominant side, unspecified cerebrovascular disease type	438.32	I69.933
Monoplegia of upper limb following unspecified cerebrovascular disease affecting right non-dominant side		I69.933
Monoplegia arm after cerebrovasc disease affect left non-dominant side	438.32	I69.934
Monoplegia of upper extremity following cerebrovascular disease affecting left non-dominant side	438.32	I69.934
Monoplegia of upper limb following unspecified cerebrovascular disease affecting left non-dominant side	438.32	I69.934
Monoplg upr lmb fol unsp cerebvasc dis aff left nondom side	438.32	I69.934
Monoplegia of upper extremity following cerebrovascular disease affecting left non-dominant side, unspecified cerebrovascular disease type	438.32	I69.934

© 2021 Eberly LA et al. *JAMA Network Open*.

Monoplegia of upper limb following unspecified cerebrovascular disease affecting left non-dominant side		I69.934
Monoplegia of upper limb affecting unspecified side, late effect of cerebrovascular disease	438.3	I69.939
Monoplegia of upper limb affecting dominant side, late effect of cerebrovascular disease	438.31	I69.939
Monoplegia of upper limb affecting nondominant side, late effect of cerebrovascular disease	438.32	I69.939
Monoplegia of upper limb, late effect of cerebrovascular disease	438.3	I69.939
Monoplegia of upper extremity following cerebrovascular disease	438.3	I69.939
Monoplegia arm affect dominant side, late effect cerebrovasc disease	438.31	I69.939
Monoplegia of upper extremity affecting dominant side, late effect of cerebrovascular disease	438.31	I69.939
Monoplegia of upper extremity, late effect of cerebrovascular disease	438.3	I69.939
Monoplegia of upper extremity affecting nondominant side, late effect of cerebrovascular disease	438.32	I69.939
Late eff,CVD,monopleg ulimb	438.3	I69.939
Late eff,CVD,monopleg ulimb, dom	438.31	I69.939
Late eff,CVD,monopleg ulimb,nond	438.32	I69.939
Monoplegia of upper limb following unspecified cerebrovascular disease affecting unspecified side	438.3	I69.939

© 2021 Eberly LA et al. *JAMA Network Open*.

Monoplegia of upper extremity following cerebrovascular disease, unspecified cerebrovascular disease type, unspecified laterality	438.3	I69.939
Monoplegia of upper limb following unspecified cerebrovascular disease affecting unspecified side		I69.939
Monoplegia of lower limb following unspecified cerebrovascular disease		I69.94
Monoplegia of leg after cerebrovasc disease affect right dominant side	438.41	I69.941
Monoplegia of lower extremity following cerebrovascular disease affecting right dominant side	438.41	I69.941
Monoplegia of lower limb following unspecified cerebrovascular disease affecting right dominant side	438.41	I69.941
Monoplg low lmb fol unsp cerebvasc dis aff right dom side	438.41	I69.941
Monoplegia of lower extremity following cerebrovascular disease affecting right dominant side, unspecified cerebrovascular disease type	438.41	I69.941
Monoplegia of lower limb following unspecified cerebrovascular disease affecting right dominant side		I69.941
Monoplegia of leg after cerebrovasc disease affect left dominant side	438.41	I69.942
Monoplegia of lower extremity following cerebrovascular disease affecting left dominant side	438.41	I69.942
Monoplegia of lower limb following unspecified cerebrovascular disease affecting left dominant side	438.41	I69.942

© 2021 Eberly LA et al. *JAMA Network Open*.

Monoplg low lmb fol unsp cerebvasc disease aff left dom side	438.41	I69.942
Monoplegia of lower extremity following cerebrovascular disease affecting left dominant side, unspecified cerebrovascular disease type	438.41	I69.942
Monoplegia of lower limb following unspecified cerebrovascular disease affecting left dominant side		I69.942
Monoplegia of lower extremity following cerebrovascular disease affecting right non-dominant side	438.42	I69.943
Monoplegia of lower limb following unspecified cerebrovascular disease affecting right non-dominant side	438.42	I69.943
Monoplg low lmb fol unsp cerebvasc dis aff right nondom side	438.42	I69.943
Monoplegia of lower extremity following cerebrovascular disease affecting right non-dominant side, unspecified cerebrovascular disease type	438.42	I69.943
Monoplegia of lower limb following unspecified cerebrovascular disease affecting right non-dominant side		I69.943
Monoplegia leg after cerebrovasc disease affect left non-dominant side	438.42	I69.944
Monoplegia of lower extremity following cerebrovascular disease affecting left non-dominant side	438.42	I69.944
Monoplegia of lower limb following unspecified cerebrovascular disease affecting left non-dominant side	438.42	I69.944

© 2021 Eberly LA et al. *JAMA Network Open*.

Monoplg low lmb fol unsp cerebvasc dis aff left nondom side	438.42	I69.944
Monoplegia of lower extremity following cerebrovascular disease affecting left non-dominant side, unspecified cerebrovascular disease type	438.42	I69.944
Monoplegia of lower limb following unspecified cerebrovascular disease affecting left non-dominant side		I69.944
Monoplegia of lower limb affecting unspecified side, late effect of cerebrovascular disease	438.4	I69.949
Monoplegia of lower limb affecting dominant side, late effect of cerebrovascular disease	438.41	I69.949
Monoplegia of lower limb affecting nondominant side, late effect of cerebrovascular disease	438.42	I69.949
Monoplegia of lower limb due to cerebrovascular disease	438.4	I69.949
Monoplegia of lower limb, late effect of cerebrovascular disease	438.4	I69.949
Monoplegia of lower limb, nondominant, due to cerebrovascular disease	438.42	I69.949
Monoplegia of lower extremity following cerebrovascular disease	438.4	I69.949
Monoplegia leg affect dominant side, late effect cerebrovasc disease	438.41	I69.949
Monoplegia of lower extremity affecting nondominant side, late effect of cerebrovascular disease	438.42	I69.949
Monoplegia of lower extremity affecting dominant side, late effect of cerebrovascular disease	438.41	I69.949

© 2021 Eberly LA et al. *JAMA Network Open*.

Monoplegia of lower extremity, late effect of cerebrovascular disease	438.4	I69.949
Late eff,CVD,monopleg llimb	438.4	I69.949
Late eff,CVD,monopleg llimb, dom	438.41	I69.949
Late eff,CVD,monopleg llimb,nond	438.42	I69.949
Monoplegia of lower limb following unspecified cerebrovascular disease affecting unspecified side	438.4	I69.949
Monoplg low lmb fol unsp cerebvasc disease aff unsp side	438.4	I69.949
Monoplegia of lower extremity following cerebrovascular disease, unspecified cerebrovascular disease type, unspecified laterality	438.4	I69.949
Monoplegia of lower limb following unspecified cerebrovascular disease affecting unspecified side		I69.949
Hemiplegia and hemiparesis following unspecified cerebrovascular disease		I69.95
Hemiplga fol unsp cerebvasc disease aff right dominant side	438.21	I69.951
Hemiplegia affecting right side in right-dominant patient as late effect of cerebrovascular disease	438.21	I69.951
Hemiplegia and hemiparesis following unspecified cerebrovascular disease affecting right dominant side	438.21	I69.951
Hemiplegia of right dominant side due to cerebrovascular disease, unspecified cerebrovascular disease type, unspecified hemiplegia type	438.21	I69.951

© 2021 Eberly LA et al. *JAMA Network Open*.

Hemiplegia and hemiparesis following unspecified cerebrovascular disease affecting right dominant side		I69.951
Hemiplga fol unsp cerebvasc disease aff left dominant side	438.21	I69.952
Hemiplegia affecting left side in left-dominant patient as late effect of cerebrovascular disease	438.21	I69.952
Hemiplegia and hemiparesis following unspecified cerebrovascular disease affecting left dominant side	438.21	I69.952
Hemiplegia of left dominant side due to cerebrovascular disease, unspecified cerebrovascular disease type, unspecified hemiplegia type	438.21	I69.952
Hemiplegia and hemiparesis following unspecified cerebrovascular disease affecting left dominant side		I69.952
Hemiplga fol unsp cerebvasc disease aff right nondom side	438.22	I69.953
Hemiplegia and hemiparesis following unspecified cerebrovascular disease affecting right non-dominant side	438.22	I69.953
Hemiplegia of right nondominant side due to cerebrovascular disease, unspecified cerebrovascular disease type, unspecified hemiplegia type	438.22	I69.953
Hemiplegia and hemiparesis following unspecified cerebrovascular disease affecting right non-dominant side		I69.953
Hemiplga fol unsp cerebvasc disease aff left nondom side	438.22	I69.954

© 2021 Eberly LA et al. *JAMA Network Open*.

Hemiplegia and hemiparesis following unspecified cerebrovascular disease affecting left non-dominant side	438.22	I69.954
Hemiplegia of left nondominant side due to cerebrovascular disease, unspecified cerebrovascular disease type, unspecified hemiplegia type	438.22	I69.954
Hemiplegia and hemiparesis following unspecified cerebrovascular disease affecting left non-dominant side		I69.954
Hemiplegia affecting unspecified side, late effect of cerebrovascular disease	438.2	I69.959
Hemiplegia affecting dominant side, late effect of cerebrovascular disease	438.21	I69.959
Hemiplegia affecting nondominant side, late effect of cerebrovascular disease	438.22	I69.959
Hemiplegia of dominant side, late effect of cerebrovascular disease	438.21	I69.959
Hemiplegia, late effect of cerebrovascular disease	438.2	I69.959
Hemiplegia due to cerebrovascular disease, late effect	438.2	I69.959
Hemiplegia or hemiparesis as late effect of cerebrovascular disease	438.2	I69.959
Hemiplegia of dominant side as late effect of cerebrovascular disease	438.21	I69.959
Hemiplegia of nondominant side, late effect of cerebrovascular disease	438.22	I69.959
Hemiplegia as late effect of cerebrovascular disease	438.2	I69.959
Hemiplegia of nondominant side as late effect of cerebrovascular disease	438.22	I69.959
Hemiplegia/hemiparesis, late effect of cerebrovascular disease	438.2	I69.959
Hemiplegia of dominant side as late effect following cerebrovascular disease	438.21	I69.959

© 2021 Eberly LA et al. *JAMA Network Open*.

Hemiplegia and hemiparesis following unspecified cerebrovascular disease affecting unspecified side	438.2	I69.959
Hemiplga following unsp cerebvasc disease aff unsp side	438.2	I69.959
Hemiplegia and hemiparesis following unspecified cerebrovascular disease affecting unspecified side		I69.959
Other paralytic syndrome following unspecified cerebrovascular disease		I69.96
Oth parlyt synd fol unsp cerebvasc dis aff right dom side	438.51	I69.961
Other paralytic syndrome following unspecified cerebrovascular disease affecting right dominant side	438.51	I69.961
Other paralytic syndrome following unspecified cerebrovascular disease affecting right dominant side		I69.961
Oth parlyt synd fol unsp cerebvasc disease aff left dom side	438.51	I69.962
Other paralytic syndrome following unspecified cerebrovascular disease affecting left dominant side	438.51	I69.962
Other paralytic syndrome following unspecified cerebrovascular disease affecting left dominant side		I69.962
Oth parlyt synd fol unsp cerebvasc dis aff right nondom side	438.52	I69.963
Other paralytic syndrome following unspecified cerebrovascular disease affecting right non-dominant side	438.52	I69.963
Other paralytic syndrome following unspecified cerebrovascular disease affecting right non-dominant side		I69.963

© 2021 Eberly LA et al. *JAMA Network Open*.

Oth parlyt synd fol unsp cerebvasc dis aff left nondom side	438.52	I69.964
Other paralytic syndrome following unspecified cerebrovascular disease affecting left non-dominant side	438.52	I69.964
Other paralytic syndrome following unspecified cerebrovascular disease affecting left non-dominant side		I69.964
Other paralytic syndrome, bilateral, late effect of cerebrovascular disease	438.53	I69.965
Other paralytic syndrome, bilateral, due to cerebrovascular disease	438.53	I69.965
Paralytic syndrome, bilateral, due to cerebrovascular disease	438.53	I69.965
Bilateral paralytic syndrome as late effect of cerebrovascular disease	438.53	I69.965
Other paralytic syndrome following unspecified cerebrovascular disease, bilateral	438.53	I69.965
Oth paralytic syndrome following unsp cerebvasc disease, bi	438.53	I69.965
Other paralytic syndrome following unspecified cerebrovascular disease, bilateral		I69.965
Other paralytic syndrome affecting unspecified side, late effect of cerebrovascular disease	438.5	I69.969
Other paralytic syndrome affecting dominant side, late effect of cerebrovascular disease	438.51	I69.969
Other paralytic syndrome affecting nondominant side, late effect of cerebrovascular disease	438.52	I69.969

© 2021 Eberly LA et al. *JAMA Network Open*.

Paralytic syndrome, late effect of cerebrovascular disease	438.5	I69.969
Paralytic syndrome affecting side	438.5	I69.969
Paralytic syndrome affecting dominant side, late effect of cerebrovascular disease	438.51	I69.969
Paralytic syndrome affecting nondominant side, late effect of cerebrovascular disease	438.52	I69.969
Other paralytic syndrome, late effect of cerebrovascular disease	438.5	I69.969
Paralytic syndrome of nondominant side as late effect of cerebrovascular disease	438.52	I69.969
Paralytic syndrome affecting dominant side as late effect of cerebrovascular disease	438.51	I69.969
Paralytic syndrome as late effect of cerebrovascular disease	438.5	I69.969
Other paralytic syndrome following cerebrovascular disease	438.5	I69.969
Other paralytic syndrome following unspecified cerebrovascular disease affecting unspecified side	438.5	I69.969
Oth parlyt syndrome fol unsp cerebvasc disease aff unsp side	438.5	I69.969
Other paralytic syndrome following unspecified cerebrovascular disease affecting unspecified side		I69.969
Other sequelae of unspecified cerebrovascular disease		I69.99
Apraxia, late effect of cerebrovascular disease	438.81	I69.990
Apraxia following cerebrovascular disease	438.81	I69.990
Apraxia as late effect of cerebrovascular disease	438.81	I69.990

© 2021 Eberly LA et al. *JAMA Network Open*.

Apraxia following unspecified cerebrovascular disease	438.81	I69.990
Apraxia following unspecified cerebrovascular disease		I69.990
Dysphagia, late effect of cerebrovascular disease	438.82	I69.991
Dysphagia following cerebrovascular disease	438.82	I69.991
Dysphagia as late effect of cerebrovascular disease	438.82	I69.991
Dysphagia following unspecified cerebrovascular disease	438.82	I69.991
Dysphagia following unspecified cerebrovascular disease		I69.991
Facial weakness due to cerebrovascular disease(438.83)	438.83	I69.992
Facial weakness following cerebrovascular disease	438.83	I69.992
Facial weakness following unspecified cerebrovascular disease	438.83	I69.992
Facial weakness following unspecified cerebrovascular disease		I69.992
Ataxia, late effect of cerebrovascular disease	438.84	I69.993
Ataxia S/P CVA	438.84	I69.993
CVA, old, ataxia	438.84	I69.993
Ataxia following cerebrovascular disease	438.84	I69.993
Ataxia following unspecified cerebrovascular disease	438.84	I69.993
Ataxia following unspecified cerebrovascular disease		I69.993
Other late effects of cerebrovascular disease(438.89)	438.89	I69.998
Other late effects of cerebrovascular disease	438.89	I69.998

© 2021 Eberly LA et al. *JAMA Network Open*.

Other sequelae following unspecified cerebrovascular disease	438.89	I69.998
Other sequelae following unspecified cerebrovascular disease		I69.998
Disturbances of vision, late effect of cerebrovascular disease	438.7	I69.998, H53.9
Vision disturbance, late effect of cerebrovascular disease	438.7	I69.998, H53.9
Abnormal vision as late effect of cerebrovascular disease	438.7	I69.998, H53.9
Alterations of sensations, late effect of cerebrovascular disease(438.6)	438.6	I69.998, R20.9
Sensation alteration, late effect of cerebrovascular disease	438.6	I69.998, R20.9
Alterations of sensations, late effect of cerebrovascular disease	438.6	I69.998, R20.9
Vertigo, late effect of cerebrovascular disease	438.85	I69.998, R42
Atherosclerosis		I70
Atherosclerosis of aorta	440	I70.0
Aortic atherosclerosis	440	I70.0
Hardening of the aorta (main artery of the heart)	440	I70.0
Intra-aortic calcification	440	I70.0
Intraaortic calcification	440	I70.0
Thoracic aortic atherosclerosis	440	I70.0
Thoracic aorta atherosclerosis	440	I70.0
Abdominal aortic atherosclerosis	440	I70.0
Atherosclerosis of abdominal aorta	440	I70.0
Atherosclerosis of aortic arch	440	I70.0
Aortic arch atherosclerosis	440	I70.0
Aortoiliac stenosis	440	I70.0
Left aortoiliac stenosis	440	I70.0
Aortoiliac stenosis, left	440	I70.0
Aortoiliac stenosis, right	440	I70.0

© 2021 Eberly LA et al. *JAMA Network Open*.

Right aortoiliac stenosis	440	I70.0
Calcification of aorta	440	I70.0
Aortic calcification	440	I70.0
Mild aortic sclerosis	440	I70.0
Stenosis of infrarenal abdominal aorta due to arteriosclerosis	440	I70.0
Stenosis of infrarenal abdominal aorta due to atherosclerosis	440	I70.0
Arteriosclerosis of abdominal aorta	440	I70.0
Arteriosclerosis of aorta	440	I70.0
Arteriosclerosis of thoracic aorta	440	I70.0
Non-rheumatic aortic sclerosis	440	I70.0
Atherosclerotic ulcer of aorta	440	I70.0
Penetrating atherosclerotic ulcer of aorta	440	I70.0
Atherosclerosis of aorta without gangrene	440	I70.0
Calcification of abdominal aorta	440	I70.0
Medial degeneration of aorta determined by biopsy	440	I70.0
Atherosclerosis of aorta		I70.0
Aorto-iliac atherosclerosis	440.0, 440.20	I70.0, I70.299
Atherosclerosis of aortic bifurcation and common iliac arteries	440.0, 440.8	I70.0, I70.8
Shaggy aorta syndrome	440.0, 444.9	I70.0, I74.9
Atherosclerosis of aorta with gangrene	440.0, 785.4	I70.0, I96
Atherosclerosis of renal artery	440.1	I70.1
Goldblatt hypertension	440.1	I70.1
Goldblatt's, hypertension	440.1	I70.1
Hypertension, Goldblatt	440.1	I70.1
Renal artery arteriosclerosis	440.1	I70.1
Renal artery atheroma	440.1	I70.1
Renal artery atherosclerosis	440.1	I70.1
Renal artery stenosis of unknown cause	440.1	I70.1
Kidney artery constriction	593.81	I70.1
Renal artery stenosis	440.1	I70.1

© 2021 Eberly LA et al. *JAMA Network Open*.

RAS (renal artery stenosis)	440.1	I70.1
Atherosclerotic renal artery stenosis	440.1	I70.1
Unilateral atherosclerotic renal artery stenosis	440.1	I70.1
Atherosclerotic renal artery stenosis, unilateral	440.1	I70.1
Atherosclerotic RAS (renal artery stenosis), unilateral	440.1	I70.1
Renal artery atherosclerosis, unilateral	440.1	I70.1
Bilateral atherosclerotic renal artery stenosis	440.1	I70.1
Bilateral renal artery stenosis	440.1	I70.1
Atherosclerotic renal artery stenosis, bilateral	440.1	I70.1
Renal artery atherosclerosis, bilateral	440.1	I70.1
Atherosclerotic RAS (renal artery stenosis), bilateral	440.1	I70.1
Non-flow-limiting renal artery stenosis	440.1	I70.1
Renal artery stenosis, non-flow-limiting	440.1	I70.1
Native stenosis of both renal arteries	440.1	I70.1
Renal artery stenosis, native, bilateral	440.1	I70.1
Native stenosis of renal artery	440.1	I70.1
Renal artery stenosis, native	440.1	I70.1
Stenosis of one of two renal arteries	440.1	I70.1
Renal artery stenosis in 1 of 2 vessels	440.1	I70.1
Stenosis of renal artery involving single vessel	440.1	I70.1
Left renal artery stenosis	440.1	I70.1
Right renal artery stenosis	440.1	I70.1
Stenosis of right renal artery	440.1	I70.1
Ischemic nephropathy with atherosclerotic renal artery stenosis	440.1	I70.1
Stenosis of both renal arteries	440.1	I70.1

© 2021 Eberly LA et al. *JAMA Network Open*.

Acquired renal artery stenosis	440.1	I70.1
Atherosclerosis of renal artery without gangrene	440.1	I70.1
Atherosclerosis of renal artery		I70.1
Atherosclerosis of renal artery with gangrene	440.1, 785.4	I70.1, I96
Atherosclerosis of native arteries of the extremities		I70.2
Unspecified atherosclerosis of native arteries of extremities		I70.20
Stenosis of right peroneal artery	440.29	I70.201
Peroneal artery stenosis, right	440.29	I70.201
Stenosis of right popliteal artery	440.2	I70.201
Popliteal artery stenosis, right	440.2	I70.201
Stenosis of right femoral artery	440.2	I70.201
Femoral artery stenosis, right	440.2	I70.201
Stenosis of right tibial artery	440.2	I70.201
Tibial artery stenosis, right	440.2	I70.201
Stenosis of right popliteal-tibial artery	440.2	I70.201
Tibial popliteal stenosis, right	440.2	I70.201
Atherosclerosis of native artery of right lower extremity	440.2	I70.201
Atherosclerosis of right leg	440.2	I70.201
Atherosclerosis of artery of right lower extremity	440.2	I70.201
Atherosclerosis of right lower extremity	440.2	I70.201
Unspecified atherosclerosis of native arteries of extremities, right leg	440.2	I70.201
Atherosclerosis of native artery of right lower extremity, with unspecified presence of clinical manifestation	440.2	I70.201
Unsp athscl native arteries of extremities, right leg	440.2	I70.201

© 2021 Eberly LA et al. *JAMA Network Open*.

Unspecified atherosclerosis of native arteries of extremities, right leg		I70.201
Stenosis of left peroneal artery	440.29	I70.202
Peroneal artery stenosis, left	440.29	I70.202
Stenosis of left popliteal artery	440.2	I70.202
Popliteal artery stenosis, left	440.2	I70.202
Femoral artery stenosis, left	440.2	I70.202
Stenosis of left femoral artery	440.2	I70.202
Stenosis of left tibial artery	440.2	I70.202
Tibial artery stenosis, left	440.2	I70.202
Stenosis of left popliteal-tibial artery	440.2	I70.202
Tibial popliteal stenosis, left	440.2	I70.202
Atherosclerosis of native artery of left lower extremity	440.2	I70.202
Atherosclerosis of left leg	440.2	I70.202
Atherosclerosis of artery of left lower extremity	440.2	I70.202
Atherosclerosis of left lower extremity	440.2	I70.202
Unspecified atherosclerosis of native arteries of extremities, left leg	440.2	I70.202
Atherosclerosis of native artery of left lower extremity, with unspecified presence of clinical manifestation	440.2	I70.202
Unsp athscl native arteries of extremities, left leg	440.2	I70.202
Unspecified atherosclerosis of native arteries of extremities, left leg		I70.202
Bilateral femoral artery stenosis	440.2	I70.203
Bilateral peroneal artery stenosis	440.29	I70.203
Peroneal artery stenosis, bilateral	440.29	I70.203
Narrowing of peroneal artery in both legs	440.29	I70.203
Atherosclerosis of native artery of both lower extremities	440.2	I70.203

© 2021 Eberly LA et al. *JAMA Network Open*.

Atherosclerosis of artery of both lower extremities	440.2	I70.203
Bilateral atherosclerosis of legs	440.2	I70.203
Atherosclerosis of both lower extremities	440.2	I70.203
Unspecified atherosclerosis of native arteries of extremities, bilateral legs	440.2	I70.203
Atherosclerosis of native artery of both lower extremities, with unspecified presence of clinical manifestation	440.2	I70.203
Unsp athscl native arteries of extremities, bilateral legs	440.2	I70.203
Unspecified atherosclerosis of native arteries of extremities, bilateral legs		I70.203
Ulnar artery stenosis	440.2	I70.208
Stenosis of ulnar artery	440.2	I70.208
Stenosis of left brachial artery	440.2	I70.208
Brachial artery stenosis, left	440.2	I70.208
Stenosis of right brachial artery	440.2	I70.208
Brachial artery stenosis, right	440.2	I70.208
Stenosis of artery of upper extremity	440.2	I70.208
Atherosclerosis of native artery of other extremity	440.2	I70.208
Unspecified atherosclerosis of native arteries of extremities, other extremity	440.2	I70.208
Atherosclerosis of other extremity	440.2	I70.208
Atherosclerosis of native artery of other extremity, with unspecified presence of clinical manifestation	440.2	I70.208
Unsp athscl native arteries of extremities, oth extremity	440.2	I70.208
Unspecified atherosclerosis of native arteries of extremities, other extremity		I70.208
Atherosclerosis of native arteries of the extremities, unspecified	440.2	I70.209

© 2021 Eberly LA et al. *JAMA Network Open*.

Monckeberg's medial sclerosis	440.2	I70.209
Monckeberg's medial calcinosis	440.2	I70.209
Peripheral arteriosclerosis	440.2	I70.209
Atherosclerosis of arteries of extremities	440.2	I70.209
Femoral-popliteal artery atherosclerosis	440.2	I70.209
Stenosis of femoral artery	440.2	I70.209
Femoral artery stenosis	440.2	I70.209
Stenosis of popliteal artery	440.2	I70.209
Stenosis of lower extremity artery	440.2	I70.209
Stenosis of tibial artery	440.2	I70.209
Tibial artery stenosis	440.2	I70.209
Arteriosclerosis of arteries of extremities	440.2	I70.209
Stenosis of popliteal-tibial artery	440.2	I70.209
Tibial popliteal stenosis	440.2	I70.209
Popliteal artery stenosis	440.2	I70.209
Atherosclerosis of native artery of extremity	440.2	I70.209
Atherosclerotic peripheral vascular disease	440.2	I70.209
Femoral-popliteal atherosclerosis	440.2	I70.209
Other atherosclerotic peripheral vascular disease	440.2	I70.209
Occlusion of artery of lower extremity due to arteriosclerosis	440.2	I70.209
Atherosclerosis of native arteries of the extremities	440.2	I70.209
Arteriosclerosis of artery of extremity	440.2	I70.209
Occlusive disease of artery of upper extremity	440.2	I70.209
Occlusive disease of artery of lower extremity	440.2	I70.209
Atheroma of artery of extremity	440.2	I70.209
Occlusion of artery of extremity	440.2	I70.209
Atherosclerosis of artery of extremity without gangrene	440.2	I70.209

© 2021 Eberly LA et al. *JAMA Network Open*.

Atherosclerotic peripheral vascular disease of extremity	440.2	I70.209
Atherosclerosis of lower extremity	440.2	I70.209
Atherosclerosis of native artery of lower extremity	440.2	I70.209
Unspecified atherosclerosis of native arteries of extremities, unspecified extremity	440.2	I70.209
Atherosclerosis of native artery of lower extremity, unspecified laterality, with unspecified presence of clinical manifestation	440.2	I70.209
Atherosclerosis of native artery of extremity, unspecified extremity, with unspecified presence of clinical manifestation	440.2	I70.209
Unsp athscl native arteries of extremities, unsp extremity	440.2	I70.209
Unspecified atherosclerosis of native arteries of extremities, unspecified extremity		I70.209
Atherosclerotic peripheral vascular disease with ulceration	440.23, 707.9	I70.209, L98.499
Atherosclerotic PVD with ulceration	440.23, 707.9	I70.209, L98.499
Atherosclerosis of extremity with ulceration	440.23, 707.9	I70.209, L98.499
Atherosclerosis of native arteries of extremities with intermittent claudication		I70.21
Atheroscler of native artery of right leg with intermit claudication	440.21	I70.211
Atherosclerosis of native artery of right lower extremity with intermittent claudication	440.21	I70.211

© 2021 Eberly LA et al. *JAMA Network Open*.

Atherosclerosis of right lower extremity with intermittent claudication	440.21	I70.211
Atherosclerosis of native arteries of extremities with intermittent claudication, right leg	440.21	I70.211
Intermittent claudication of right lower extremity due to atherosclerosis	440.21	I70.211
Athscl native arteries of extrm w intrmt claud, right leg	440.21	I70.211
Atherosclerosis of native arteries of extremities with intermittent claudication, right leg		I70.211
Atheroscler of native artery of left leg with intermit claudication	440.21	I70.212
Atherosclerosis of native artery of left lower extremity with intermittent claudication	440.21	I70.212
Atherosclerosis of left lower extremity with intermittent claudication	440.21	I70.212
Atherosclerosis of native arteries of extremities with intermittent claudication, left leg	440.21	I70.212
Intermittent claudication of left lower extremity due to atherosclerosis	440.21	I70.212
Athscl native arteries of extrm w intrmt claud, left leg	440.21	I70.212
Atherosclerosis of native arteries of extremities with intermittent claudication, left leg		I70.212
Atheroscler of native artery of both legs with intermit claudication	440.21	I70.213
Atherosclerosis of native artery of both lower extremities with intermittent claudication	440.21	I70.213

© 2021 Eberly LA et al. *JAMA Network Open*.

Atherosclerosis of both lower extremities with intermittent claudication	440.21	I70.213
Atherosclerosis of native arteries of extremities with intermittent claudication, bilateral legs	440.21	I70.213
Intermittent claudication of both lower extremities due to atherosclerosis	440.21	I70.213
Athscl native arteries of extrm w intrmt claud, bi legs	440.21	I70.213
Atherosclerosis of native arteries of extremities with intermittent claudication, bilateral legs		I70.213
Athscl native arteries of extrm w intrmt claud, oth extrm	440.21	I70.218
Atherosclerosis of native artery of other extremity with intermittent claudication	440.21	I70.218
Atherosclerosis of native arteries of extremities with intermittent claudication, other extremity	440.21	I70.218
Atherosclerosis of other extremity with intermittent claudication	440.21	I70.218
Atherosclerosis of native arteries of extremities with intermittent claudication, other extremity		I70.218
Atherosclerosis of native arteries of the extremities with intermittent claudication	440.21	I70.219
Extremity atherosclerosis with intermittent claudication	440.21	I70.219
Atherosclerotic femoro-popliteal artery disease with claudication	440.21	I70.219
Atherosclerosis of leg with intermittent claudication	440.21	I70.219
Atherosclerotic PVD with intermittent claudication	440.21	I70.219

© 2021 Eberly LA et al. *JAMA Network Open*.

Atheroscler native arteries the extremities w/intermit claudication	440.21	I70.219
Atherosclerotic peripheral vascular disease with intermittent claudication	440.21	I70.219
Atherosclerosis of native arteries of extremity with intermittent claudication	440.21	I70.219
Atherosclerosis of lower extremity with claudication	440.21	I70.219
Atherosclerosis of artery of extremity with intermittent claudication	440.21	I70.219
Atherosclerosis of lower extremity with intermittent claudication	440.21	I70.219
Atherosclerosis with limb claudication	440.21	I70.219
Atherosclerosis with claudication of extremity	440.21	I70.219
Atheroscler-limb&claudic	440.21	I70.219
Atherosclerosis of native artery of extremity with intermittent claudication	440.21	I70.219
Atherosclerosis of native artery of lower extremity with intermittent claudication	440.21	I70.219
Atherosclerosis of extremity with intermittent claudication	440.21	I70.219
Atherosclerosis of native arteries of extremities with intermittent claudication, unspecified extremity	440.21	I70.219
Atherosclerosis of native artery of lower extremity with intermittent claudication, unspecified laterality	440.21	I70.219
Atherosclerosis of native artery of extremity with intermittent claudication, unspecified extremity	440.21	I70.219
Athscl native arteries of extrm w intrmt claud, unsp extrm	440.21	I70.219
Atherosclerosis of native arteries of extremities with intermittent claudication, unspecified extremity		I70.219

© 2021 Eberly LA et al. *JAMA Network Open*.

Atherosclerosis of native arteries of extremities with rest pain		I70.22
Atherosclerosis of native artery of right leg with rest pain	440.22	I70.221
Atherosclerosis of native artery of right lower extremity with rest pain	440.22	I70.221
Atherosclerosis of right lower extremity with rest pain	440.22	I70.221
Atherosclerosis of native arteries of extremities with rest pain, right leg	440.22	I70.221
Athscl native arteries of extremities w rest pain, right leg	440.22	I70.221
Atherosclerosis of native arteries of extremities with rest pain, right leg		I70.221
Atherosclerosis of native artery of left leg with rest pain	440.22	I70.222
Atherosclerosis of native artery of left lower extremity with rest pain	440.22	I70.222
Atherosclerosis of left lower extremity with rest pain	440.22	I70.222
Atherosclerosis of native arteries of extremities with rest pain, left leg	440.22	I70.222
Athscl native arteries of extremities w rest pain, left leg	440.22	I70.222
Atherosclerosis of native arteries of extremities with rest pain, left leg		I70.222
Atherosclerosis of native artery of both legs with rest pain	440.22	I70.223
Atherosclerosis of native artery of both lower extremities with rest pain	440.22	I70.223
Atherosclerosis of both lower extremities with rest pain	440.22	I70.223
Atherosclerosis of native arteries of extremities with rest pain, bilateral legs	440.22	I70.223
Athscl native arteries of extrm w rest pain, bilateral legs	440.22	I70.223

© 2021 Eberly LA et al. *JAMA Network Open*.

Atherosclerosis of native arteries of extremities with rest pain, bilateral legs		I70.223
Athscl native arteries of extrm w rest pain, oth extremity	440.22	I70.228
Atherosclerosis of native artery of other extremity with rest pain	440.22	I70.228
Atherosclerosis of native arteries of extremities with rest pain, other extremity	440.22	I70.228
Atherosclerosis of other extremity with rest pain	440.22	I70.228
Atherosclerosis of native arteries of extremities with rest pain, other extremity		I70.228
Atherosclerosis of native arteries of the extremities with rest pain	440.22	I70.229
Extremity atherosclerosis with resting pain	440.22	I70.229
Atherosclerotic femoro-popliteal artery disease with rest pain	440.22	I70.229
Atherosclerotic peripheral vascular disease with rest pain	440.22	I70.229
Atherosclerosis of native arteries of extremity with rest pain	440.22	I70.229
Atherosclerosis of artery of extremity with rest pain	440.22	I70.229
Atherosclerosis of native artery of extremity with rest pain	440.22	I70.229
Atherosclerosis of native artery of lower extremity with rest pain	440.22	I70.229
Atherosclerosis of lower extremity with rest pain	440.22	I70.229
Atherosclerosis of extremity with rest pain	440.22	I70.229
Atherosclerosis of native arteries of extremities with rest pain, unspecified extremity	440.22	I70.229

© 2021 Eberly LA et al. *JAMA Network Open*.

Atherosclerosis of native artery of lower extremity with rest pain, unspecified laterality	440.22	I70.229
Atherosclerosis of native artery of extremity with rest pain, unspecified extremity	440.22	I70.229
Athscl native arteries of extrm w rest pain, unsp extremity	440.22	I70.229
Atherosclerosis of native arteries of extremities with rest pain, unspecified extremity		I70.229
Atherosclerosis of native arteries of right leg with ulceration		I70.23
Atherosclerosis of native artery of right leg with ulceration of thigh	440.23, 707.11	I70.231
Atherosclerosis of native artery of right lower extremity with ulceration of thigh	440.23, 707.11	I70.231
Atherosclerosis of right lower extremity with ulceration of thigh	440.23, 707.11	I70.231
Atherosclerosis of native arteries of right leg with ulceration of thigh	440.23, 707.11	I70.231
Athscl native arteries of right leg w ulceration of thigh	440.23, 707.11	I70.231
Atherosclerosis of native arteries of right leg with ulceration of thigh		I70.231
Atherosclerosis of native artery of both lower extremities with bilateral ulceration of thighs	440.23, 707.11	I70.231, I70.241
Atherosclerosis of both lower extremities with bilateral ulceration of thighs	440.23, 707.11	I70.231, I70.241
Atherosclerosis of native artery of right leg with ulceration of calf	440.23, 707.12	I70.232
Atherosclerosis of native artery of right lower extremity with ulceration of calf	440.23, 707.12	I70.232

© 2021 Eberly LA et al. *JAMA Network Open*.

Atherosclerosis of right lower extremity with ulceration of calf	440.23, 707.12	I70.232
Atherosclerosis of native arteries of right leg with ulceration of calf	440.23, 707.12	I70.232
Athscl native arteries of right leg w ulceration of calf	440.23, 707.12	I70.232
Atherosclerosis of native arteries of right leg with ulceration of calf		I70.232
Atherosclerosis of native artery of both lower extremities with bilateral ulceration of calf	440.23, 707.12	I70.232, I70.242
Atherosclerosis of native artery of both lower extremities with bilateral ulceration of calves	440.23, 707.12	I70.232, I70.242
Atherosclerosis of both lower extremities with bilateral ulceration of calves	440.23, 707.12	I70.232, I70.242
Atherosclerosis of native artery of right leg with ulceration of ankle	440.23, 707.13	I70.233
Atherosclerosis of native artery of right lower extremity with ulceration of ankle	440.23, 707.13	I70.233
Atherosclerosis of right lower extremity with ulceration of ankle	440.23, 707.13	I70.233
Atherosclerosis of native arteries of right leg with ulceration of ankle	440.23, 707.13	I70.233
Athscl native arteries of right leg w ulceration of ankle	440.23, 707.13	I70.233
Atherosclerosis of native arteries of right leg with ulceration of ankle		I70.233
Atherosclerosis of native artery of both lower extremities with bilateral ulceration of ankles	440.23, 707.13	I70.233, I70.243
Atherosclerosis of both lower extremities with bilateral ulceration of ankles	440.23, 707.13	I70.233, I70.243
Athscl native art of right leg w ulcer of heel and midfoot	440.23, 707.9	I70.234

© 2021 Eberly LA et al. *JAMA Network Open*.

Atherosclerosis of right lower extremity with ulceration of heel	440.23, 707.14	I70.234
Atherosclerosis of right lower extremity with ulceration of midfoot	440.23, 707.14	I70.234
Atherosclerosis of native artery of right lower extremity with ulceration of heel	440.23, 707.14	I70.234
Atherosclerosis of native artery of right lower extremity with ulceration of midfoot	440.23, 707.14	I70.234
Atherosclerosis of native arteries of right leg with ulceration of heel and midfoot	440.23, 707.9	I70.234
Atherosclerosis of native arteries of right leg with ulceration of heel and midfoot		I70.234
Atherosclerosis of native artery of both lower extremities with bilateral ulceration of midfeet	440.23, 707.14	I70.234, I70.244
Atherosclerosis of both lower extremities with bilateral ulceration of midfeet	440.23, 707.14	I70.234, I70.244
Atherosclerosis of native artery of both lower extremities with bilateral ulceration of heels	440.23, 707.14	I70.234, I70.244
Atherosclerosis of both lower extremities with bilateral ulceration of heels	440.23, 707.14	I70.234, I70.244
Athscl native arteries of right leg w ulcer oth prt foot	440.23, 707.9	I70.235
Atherosclerosis of native artery of right lower extremity with ulceration of other part of foot	440.23, 707.9	I70.235
Atherosclerosis of native arteries of right leg with ulceration of other part of foot	440.23, 707.9	I70.235
Atherosclerosis of right lower extremity with ulceration of other part of foot	440.23, 707.15	I70.235
Atherosclerosis of native arteries of right leg with ulceration of other part of foot		I70.235

© 2021 Eberly LA et al. *JAMA Network Open*.

Atherosclerosis of native artery of both lower extremities with bilateral ulceration of other part of feet	440.23, 707.15	I70.235, I70.245
Atherosclerosis of both lower extremities with bilateral ulceration of other part of feet	440.23, 707.15	I70.235, I70.245
Athscl natv art of right leg w ulcer oth prt lower right leg	440.23, 707.9	I70.238
Atherosclerosis of native artery of right lower extremity with ulceration of other part of lower leg	440.23, 707.9	I70.238
Atherosclerosis of native arteries of right leg with ulceration of other part of lower right leg	440.23, 707.9	I70.238
Atherosclerosis of right lower extremity with ulceration of other part of lower leg	440.23, 707.19	I70.238
Atherosclerosis of native arteries of right leg with ulceration of other part of lower right leg		I70.238
Atherosclerosis of both lower extremities with bilateral ulceration of other part of lower legs	440.23, 707.19	I70.238, I70.248
Atherosclerosis of native artery of both lower extremities with bilateral ulceration of other part of lower legs	440.23, 707.19	I70.238, I70.248
Atherosclerosis of native artery of right leg with ulceration	440.23, 707.9	I70.239
Atherosclerosis of native artery of right lower extremity with ulceration	440.23, 707.9	I70.239
Atherosclerosis of right lower extremity with ulceration	440.23, 707.10	I70.239
Atherosclerosis of native arteries of right leg with ulceration of unspecified site	440.23, 707.9	I70.239
Atherosclerosis of native artery of right lower extremity with ulceration, unspecified ulceration site	440.23, 707.9	I70.239

© 2021 Eberly LA et al. *JAMA Network Open*.

Athscl native arteries of right leg w ulcer of unsp site	440.23, 707.9	I70.239
Atherosclerosis of native arteries of right leg with ulceration of unspecified site		I70.239
Atherosclerosis of both lower extremities with bilateral ulceration	440.23, 707.10	I70.239, I70.249
Atherosclerosis of native artery of both lower extremities with bilateral ulceration	440.23, 707.10	I70.239, I70.249
Atherosclerosis of native artery of both lower extremities with bilateral ulceration, unspecified ulceration site	440.23, 707.10	I70.239, I70.249
Atherosclerosis of native arteries of left leg with ulceration		I70.24
Atherosclerosis of native artery of left leg with ulceration of thigh	440.23, 707.11	I70.241
Atherosclerosis of native artery of left lower extremity with ulceration of thigh	440.23, 707.11	I70.241
Atherosclerosis of left lower extremity with ulceration of thigh	440.23, 707.11	I70.241
Atherosclerosis of native arteries of left leg with ulceration of thigh	440.23, 707.11	I70.241
Athscl native arteries of left leg w ulceration of thigh	440.23, 707.11	I70.241
Atherosclerosis of native arteries of left leg with ulceration of thigh		I70.241
Atherosclerosis of native artery of left leg with ulceration of calf	440.23, 707.12	I70.242
Atherosclerosis of native artery of left lower extremity with ulceration of calf	440.23, 707.12	I70.242
Atherosclerosis of left lower extremity with ulceration of calf	440.23, 707.12	I70.242
Atherosclerosis of native arteries of left leg with ulceration of calf	440.23, 707.12	I70.242
Atherosclerosis of native arteries of left leg with ulceration of calf		I70.242

© 2021 Eberly LA et al. *JAMA Network Open*.

Atherosclerosis of native artery of left leg with ulceration of ankle	440.23, 707.13	I70.243
Atherosclerosis of native artery of left lower extremity with ulceration of ankle	440.23, 707.13	I70.243
Atherosclerosis of left lower extremity with ulceration of ankle	440.23, 707.13	I70.243
Atherosclerosis of native arteries of left leg with ulceration of ankle	440.23, 707.13	I70.243
Athscl native arteries of left leg w ulceration of ankle	440.23, 707.13	I70.243
Atherosclerosis of native arteries of left leg with ulceration of ankle		I70.243
Athscl native art of left leg w ulcer of heel and midfoot	440.23, 707.9	I70.244
Atherosclerosis of native artery of left lower extremity with ulceration of midfoot	440.23, 707.14	I70.244
Atherosclerosis of left lower extremity with ulceration of midfoot	440.23, 707.14	I70.244
Atherosclerosis of native artery of left lower extremity with ulceration of heel	440.23, 707.14	I70.244
Atherosclerosis of left lower extremity with ulceration of heel	440.23, 707.14	I70.244
Atherosclerosis of native arteries of left leg with ulceration of heel and midfoot	440.23, 707.9	I70.244
Atherosclerosis of native arteries of left leg with ulceration of heel and midfoot		I70.244
Athscl native arteries of left leg w ulceration oth prt foot	440.23, 707.9	I70.245
Atherosclerosis of native artery of left lower extremity with ulceration of other part of foot	440.23, 707.9	I70.245
Atherosclerosis of native arteries of left leg with ulceration of other part of foot	440.23, 707.9	I70.245
Atherosclerosis of left lower extremity with ulceration of other part of foot	440.23, 707.15	I70.245

© 2021 Eberly LA et al. *JAMA Network Open*.

Atherosclerosis of native arteries of left leg with ulceration of other part of foot		I70.245
Athscl native art of left leg w ulcer oth prt lower left leg	440.23, 707.9	I70.248
Atherosclerosis of native artery of left lower extremity with ulceration of other part of lower leg	440.23, 707.9	I70.248
Atherosclerosis of native arteries of left leg with ulceration of other part of lower left leg	440.23, 707.9	I70.248
Atherosclerosis of left lower extremity with ulceration of other part of lower leg	440.23, 707.19	I70.248
Atherosclerosis of native arteries of left leg with ulceration of other part of lower left leg		I70.248
Atherosclerosis of native artery of left leg with ulceration	440.23, 707.9	I70.249
Atherosclerosis of native artery of left lower extremity with ulceration	440.23, 707.9	I70.249
Atherosclerosis of left lower extremity with ulceration	440.23, 707.10	I70.249
Atherosclerosis of native arteries of left leg with ulceration of unspecified site	440.23, 707.9	I70.249
Atherosclerosis of native artery of left lower extremity with ulceration, unspecified ulceration site	440.23, 707.9	I70.249
Athscl native arteries of left leg w ulceration of unsp site	440.23, 707.9	I70.249
Atherosclerosis of native arteries of left leg with ulceration of unspecified site		I70.249
Atherosclerotic femoro-popliteal artery disease with ulceration	440.23	I70.25
Atherosclerosis of native artery of leg with ulceration of ankle	440.23, 707.13	I70.25

© 2021 Eberly LA et al. *JAMA Network Open*.

Atherosclerosis of native artery of leg with ulceration of midfoot	440.23, 707.14	I70.25
Atherosclerosis of native artery of leg with ulceration of calf	440.23, 707.12	I70.25
Atherosclerosis of native artery of leg with ulceration of heel	440.23, 707.14	I70.25
Atherosclerosis of native artery of lower extremity with ulceration of calf	440.23, 707.12	I70.25
Atherosclerosis of native artery of lower extremity with ulceration of ankle	440.23, 707.13	I70.25
Atherosclerosis of native artery of lower extremity with ulceration of heel	440.23, 707.14	I70.25
Atherosclerosis of native artery of lower extremity with ulceration of midfoot	440.23, 707.14	I70.25
Atherosclerosis of native arteries of the extremities with ulceration	440.23, 707.9	I70.25
Atherosclerosis of lower extremity with ulceration of thigh	440.23, 707.11	I70.25
Atherosclerosis of lower extremity with ulceration	440.23, 707.10	I70.25
Atherosclerosis of native artery of lower extremity with ulceration	440.23, 707.10	I70.25
Atherosclerosis of lower extremity with ulceration of heel	440.23, 707.14	I70.25
Atherosclerosis of native artery of lower extremity with ulceration of thigh	440.23, 707.11	I70.25
Atherosclerosis of lower extremity with ulceration of ankle	440.23, 707.13	I70.25
Atherosclerosis of lower extremity with ulceration of midfoot	440.23, 707.14	I70.25
Atherosclerosis of lower extremity with ulceration of calf	440.23, 707.12	I70.25
Atherosclerosis of native artery of extremity with ulceration	440.23, 707.9	I70.25
Atherosclerosis of native artery of other extremity with ulceration	440.23, 707.9	I70.25

© 2021 Eberly LA et al. *JAMA Network Open*.

Atherosclerosis of native arteries of other extremities with ulceration	440.23, 707.9	I70.25
Atherosclerosis of other extremity with ulceration	440.23, 707.9	I70.25
Atherosclerosis of native artery of lower extremity with ulceration of other part of foot	440.23, 707.15	I70.25
Atherosclerosis of native artery of lower extremity with ulceration of other part of lower leg	440.23, 707.19	I70.25
Atherosclerosis of lower extremity with ulceration of other part of foot	440.23, 707.15	I70.25
Atherosclerosis of lower extremity with ulceration of other part of lower leg	440.23, 707.19	I70.25
Atherosclerosis of native artery of lower extremity with ulceration of midfoot, unspecified laterality	440.23, 707.14	I70.25
Atherosclerosis of native artery of lower extremity with ulceration of calf, unspecified laterality	440.23, 707.12	I70.25
Atherosclerosis of native artery of lower extremity with ulceration of ankle, unspecified laterality	440.23, 707.13	I70.25
Atherosclerosis of native artery of lower extremity with ulceration, unspecified laterality, unspecified ulceration site	440.23, 707.10	I70.25
Atherosclerosis of native artery of lower extremity with ulceration of other part of lower leg, unspecified laterality	440.23, 707.19	I70.25
Atherosclerosis of native artery of lower extremity with ulceration of heel, unspecified laterality	440.23, 707.14	I70.25
Atherosclerosis of native artery of lower extremity with ulceration of thigh, unspecified laterality	440.23, 707.11	I70.25

© 2021 Eberly LA et al. *JAMA Network Open*.

Atherosclerosis of native artery of lower extremity with ulceration of other part of foot, unspecified laterality	440.23, 707.15	I70.25
Atherosclerosis of native artery of extremity with ulceration, unspecified extremity	440.23, 707.9	I70.25
Athscl native arteries of extremities w ulceration	440.23, 707.9	I70.25
Atherosclerosis of native arteries of other extremities with ulceration		I70.25
Ischemic ulcer of lower leg due to atherosclerotic disease	440.23, 707.12	I70.25, L97.209
Ischemic ulcer of lower leg due to atherosclerosis	440.23, 707.12	I70.25, L97.209
Ischemic ulcer of midfoot due to atherosclerosis of native artery of extremity	440.23, 707.14	I70.25, L97.409
Ischemic midfoot ulcer due to atherosclerosis of native artery of limb	440.23, 707.14	I70.25, L97.409
Ischemic ulcer of foot due to atherosclerosis of native artery of extremity	440.23, 707.15	I70.25, L97.509
Ischemic foot ulcer due to atherosclerosis of native artery of limb	440.23, 707.15	I70.25, L97.509
Atherosclerosis of native arteries of extremities with gangrene		I70.26
Atherosclerosis of native artery of right leg with gangrene	440.24	I70.261
Atherosclerosis of native artery of right lower extremity with gangrene	440.24	I70.261
Atherosclerosis of right lower extremity with gangrene	440.24	I70.261
Atherosclerosis of native arteries of extremities with gangrene, right leg	440.24	I70.261

© 2021 Eberly LA et al. *JAMA Network Open*.

Athscl native arteries of extremities w gangrene, right leg	440.24	I70.261
Atherosclerosis of native arteries of extremities with gangrene, right leg		I70.261
Atherosclerosis of native artery of left lower extremity with gangrene	440.24	I70.262
Atherosclerosis of left lower extremity with gangrene	440.24	I70.262
Atherosclerosis of native arteries of extremities with gangrene, left leg	440.24	I70.262
Athscl native arteries of extremities w gangrene, left leg	440.24	I70.262
Atherosclerosis of native arteries of extremities with gangrene, left leg		I70.262
Atherosclerosis of native artery of both legs with gangrene	440.24	I70.263
Atherosclerosis of native artery of both lower extremities with gangrene	440.24	I70.263
Atherosclerosis of both lower extremities with gangrene	440.24	I70.263
Atherosclerosis of native arteries of extremities with gangrene, bilateral legs	440.24	I70.263
Athscl native arteries of extrm w gangrene, bilateral legs	440.24	I70.263
Atherosclerosis of native arteries of extremities with gangrene, bilateral legs		I70.263
Athscl native arteries of extrm w gangrene, oth extremity	440.24	I70.268
Atherosclerosis of native artery of other extremity with gangrene	440.24	I70.268
Atherosclerosis of native arteries of extremities with gangrene, other extremity	440.24	I70.268
Atherosclerosis of other extremity with gangrene	440.24	I70.268
Atherosclerosis of native arteries of extremities with gangrene, other extremity		I70.268

© 2021 Eberly LA et al. *JAMA Network Open*.

Atherosclerosis of native arteries of the extremities with gangrene	440.24	I70.269
Extremity atherosclerosis with gangrene	440.24	I70.269
Gangrene from atherosclerosis, extremities	440.24	I70.269
Atherosclerosis of native arteries of extremity with gangrene	440.24	I70.269
Atherosclerotic peripheral vascular disease with gangrene	440.24	I70.269
Atherosclerotic femoro-popliteal artery disease with gangrene	440.24	I70.269
Arteriosclerotic gangrene	440.24	I70.269
Generalized atherosclerosis with gangrene	440.24	I70.269
Atherosclerosis of artery of extremity with gangrene	440.24	I70.269
Atherosclerosis of native artery of extremity with gangrene	440.24	I70.269
Atherosclerosis of native artery of lower extremity with gangrene	440.24	I70.269
Atherosclerosis of extremity with gangrene	440.24	I70.269
Atherosclerosis of lower extremity with gangrene	440.24	I70.269
Atherosclerosis of native arteries of extremities with gangrene, unspecified extremity	440.24	I70.269
Atherosclerosis of native artery of lower extremity with gangrene, unspecified laterality	440.24	I70.269
Atherosclerosis of native artery of extremity with gangrene, unspecified extremity	440.24	I70.269
Athscl native arteries of extrm w gangrene, unsp extremity	440.24	I70.269

© 2021 Eberly LA et al. *JAMA Network Open*.

Atherosclerosis of native arteries of extremities with gangrene, unspecified extremity		I70.269
Other atherosclerosis of native arteries of extremities		I70.29
Other atherosclerosis of native arteries of extremities, right leg	440.29	I70.291
Atherosclerosis of native artery of right lower extremity with other clinical manifestation	440.29	I70.291
Atherosclerosis of right lower extremity with other clinical manifestation	440.29	I70.291
Other atherosclerosis of native arteries of extremities, right leg		I70.291
Other atherosclerosis of native arteries of extremities, left leg	440.29	I70.292
Atherosclerosis of left lower extremity with other clinical manifestation	440.29	I70.292
Atherosclerosis of native artery of left lower extremity with other clinical manifestation	440.29	I70.292
Other atherosclerosis of native arteries of extremities, left leg		I70.292
Oth athscl native arteries of extremities, bilateral legs	440.29	I70.293
Other atherosclerosis of native arteries of extremities, bilateral legs	440.29	I70.293
Atherosclerosis of native artery of both lower extremities with other clinical manifestation	440.29	I70.293
Atherosclerosis of both lower extremities with other clinical manifestation	440.29	I70.293
Other atherosclerosis of native arteries of extremities, bilateral legs		I70.293

© 2021 Eberly LA et al. *JAMA Network Open*.

Other atherosclerosis of native artery of other extremity	440.29	I70.298
Other atherosclerosis of native arteries of extremities, other extremity	440.29	I70.298
Oth athscl native arteries of extremities, oth extremity	440.29	I70.298
Atherosclerosis of other extremity with other clinical manifestation	440.29	I70.298
Atherosclerosis of native artery of other extremity with other clinical manifestation	440.29	I70.298
Other atherosclerosis of native arteries of extremities, other extremity		I70.298
Other atherosclerosis of native arteries of the extremities	440.29	I70.299
Other atherosclerosis of native artery of extremity	440.29	I70.299
Other atherosclerosis of native arteries of extremities, unspecified extremity	440.29	I70.299
Atherosclerosis of native artery of extremity with other clinical manifestation	440.29	I70.299
Atherosclerosis of extremity with other clinical manifestation	440.29	I70.299
Atherosclerosis of native artery of lower extremity with other clinical manifestation	440.29	I70.299
Atherosclerosis of lower extremity with other clinical manifestation	440.29	I70.299
Atherosclerosis of native artery of lower extremity with other clinical manifestation, unspecified laterality	440.29	I70.299
Atherosclerosis of native artery of extremity with other clinical manifestation, unspecified extremity	440.29	I70.299
Oth athscl native arteries of extremities, unsp extremity	440.29	I70.299

© 2021 Eberly LA et al. *JAMA Network Open*.

Other atherosclerosis of native arteries of extremities, unspecified extremity		I70.299
Atherosclerosis of native artery of leg with ulceration of foot	440.23, 707.15	I70.299, L97.509
Atherosclerosis of native artery of lower extremity with ulceration of foot	440.23, 707.15	I70.299, L97.509
Atherosclerosis of artery of extremity with ulceration	440.23	I70.299, L97.909
Atherosclerosis of unspecified type of bypass graft(s) of the extremities		I70.3
Unspecified atherosclerosis of unspecified type of bypass graft(s) of the extremities		I70.30
Atherosclerosis of bypass graft of right lower extremity	440.3	I70.301
Unspecified atherosclerosis of unspecified type of bypass graft(s) of the extremities, right leg	440.3	I70.301
Atherosclerosis of bypass graft of right lower extremity, with unspecified presence of clinical manifestation	440.3	I70.301
Unsp athscl unsp type bypass of the extremities, right leg	440.3	I70.301
Unspecified atherosclerosis of unspecified type of bypass graft(s) of the extremities, right leg		I70.301
Atherosclerosis of bypass graft of left lower extremity	440.3	I70.302
Unspecified atherosclerosis of unspecified type of bypass graft(s) of the extremities, left leg	440.3	I70.302
Atherosclerosis of bypass graft of left lower extremity, with unspecified presence of clinical manifestation	440.3	I70.302
Unsp athscl unsp type bypass of the extremities, left leg	440.3	I70.302

© 2021 Eberly LA et al. *JAMA Network Open*.

Unspecified atherosclerosis of unspecified type of bypass graft(s) of the extremities, left leg		I70.302
Atherosclerosis of bypass graft of both lower extremities	440.3	I70.303
Unspecified atherosclerosis of unspecified type of bypass graft(s) of the extremities, bilateral legs	440.3	I70.303
Atherosclerosis of bypass graft of both lower extremities, with unspecified presence of clinical manifestation	440.3	I70.303
Unsp athscl unsp type bypass of the extrm, bilateral legs	440.3	I70.303
Unspecified atherosclerosis of unspecified type of bypass graft(s) of the extremities, bilateral legs		I70.303
Unsp athscl unsp type bypass of the extrm, oth extremity	440.3	I70.308
Atherosclerosis of bypass graft of other extremity	440.3	I70.308
Unspecified atherosclerosis of unspecified type of bypass graft(s) of the extremities, other extremity	440.3	I70.308
Atherosclerosis of bypass graft of other extremity, with unspecified presence of clinical manifestation	440.3	I70.308
Unspecified atherosclerosis of unspecified type of bypass graft(s) of the extremities, other extremity		I70.308
Atherosclerosis of unspecified bypass graft of extremities	440.3	I70.309
Atherosclerosis of bypass graft of extremity	440.3	I70.309
Atherosclerosis of bypass graft of limb	440.3	I70.309
Atherosclerosis of aorto-iliac bypass graft	440.3	I70.309

© 2021 Eberly LA et al. *JAMA Network Open*.

Hardening of bypass graft of limb	440.3	I70.309
Atherosclerosis of bypass graft of extremities	440.3	I70.309
Atherosclerosis of bypass graft of the extremities	440.3	I70.309
Atherosclerosis of bypass graft of lower extremity	440.3	I70.309
Unspecified atherosclerosis of unspecified type of bypass graft(s) of the extremities, unspecified extremity	440.3	I70.309
Atherosclerosis of bypass graft of lower extremity, unspecified laterality, with unspecified presence of clinical manifestation	440.3	I70.309
Atherosclerosis of bypass graft of extremity, unspecified extremity, with unspecified presence of clinical manifestation	440.3	I70.309
Hardening of bypass graft of extremity	440.3	I70.309
Unsp athscl unsp type bypass of the extrm, unsp extremity	440.3	I70.309
Unspecified atherosclerosis of unspecified type of bypass graft(s) of the extremities, unspecified extremity		I70.309
Atherosclerosis of unspecified type of bypass graft(s) of the extremities with intermittent claudication		I70.31
Atheroscler of bypass graft of right leg with intermit claudication	440.3	I70.311
Atherosclerosis of bypass graft of right lower extremity with intermittent claudication	440.3	I70.311
Atherosclerosis of unspecified type of bypass graft(s) of the extremities with intermittent claudication, right leg	440.3	I70.311

© 2021 Eberly LA et al. *JAMA Network Open*.

Athscl unsp type bypass of extrm w intrmt claud, right leg	440.3	I70.311
Atherosclerosis of unspecified type of bypass graft(s) of the extremities with intermittent claudication, right leg		I70.311
Atheroscler of bypass graft of left leg with intermittent claudication	440.3	I70.312
Atherosclerosis of bypass graft of left lower extremity with intermittent claudication	440.3	I70.312
Atherosclerosis of unspecified type of bypass graft(s) of the extremities with intermittent claudication, left leg	440.3	I70.312
Athscl unsp type bypass of extrm w intrmt claud, left leg	440.3	I70.312
Atherosclerosis of unspecified type of bypass graft(s) of the extremities with intermittent claudication, left leg		I70.312
Atheroscler of bypass graft of both legs with intermit claudication	440.3	I70.313
Atherosclerosis of bypass graft of both lower extremities with intermittent claudication	440.3	I70.313
Atherosclerosis of unspecified type of bypass graft(s) of the extremities with intermittent claudication, bilateral legs	440.3	I70.313
Athscl unsp type bypass of the extrm w intrmt claud, bi legs	440.3	I70.313
Atherosclerosis of unspecified type of bypass graft(s) of the extremities with intermittent claudication, bilateral legs		I70.313
Athscl unsp type bypass of extrm w intrmt claud, oth extrm	440.3	I70.318

© 2021 Eberly LA et al. *JAMA Network Open*.

Atherosclerosis of bypass graft of other extremity with intermittent claudication	440.3	I70.318
Atherosclerosis of unspecified type of bypass graft(s) of the extremities with intermittent claudication, other extremity	440.3	I70.318
Atherosclerosis of unspecified type of bypass graft(s) of the extremities with intermittent claudication, other extremity		I70.318
Atheroscler of bypass graft of extremity with intermit claudication	440.3	I70.319
Atherosclerosis of bypass graft of extremity with intermittent claudication	440.3	I70.319
Atherosclerosis of bypass graft of lower extremity with intermittent claudication	440.30, 443.9	I70.319
Atherosclerosis of unspecified type of bypass graft(s) of the extremities with intermittent claudication, unspecified extremity	440.3	I70.319
Atherosclerosis of bypass graft of lower extremity with intermittent claudication, unspecified laterality	440.30, 443.9	I70.319
Atherosclerosis of bypass graft of extremity with intermittent claudication, unspecified extremity	440.3	I70.319
Athscl unsp type bypass of extrm w intrmt claud, unsp extrm	440.3	I70.319
Atherosclerosis of unspecified type of bypass graft(s) of the extremities with intermittent claudication, unspecified extremity		I70.319
Atherosclerosis of unspecified type of bypass graft(s) of the extremities with rest pain		I70.32

© 2021 Eberly LA et al. *JAMA Network Open*.

Atherosclerosis of bypass graft of right leg with rest pain	440.3	I70.321
Atherosclerosis of bypass graft of right lower extremity with rest pain	440.3	I70.321
Atherosclerosis of unspecified type of bypass graft(s) of the extremities with rest pain, right leg	440.3	I70.321
Athscl unsp type bypass of the extrm w rest pain, right leg	440.3	I70.321
Atherosclerosis of unspecified type of bypass graft(s) of the extremities with rest pain, right leg		I70.321
Atherosclerosis of bypass graft of left lower extremity with rest pain	440.3	I70.322
Atherosclerosis of unspecified type of bypass graft(s) of the extremities with rest pain, left leg	440.3	I70.322
Athscl unsp type bypass of the extrm w rest pain, left leg	440.3	I70.322
Atherosclerosis of unspecified type of bypass graft(s) of the extremities with rest pain, left leg		I70.322
Atherosclerosis of bypass graft of both legs with rest pain	440.3	I70.323
Atherosclerosis of bypass graft of both lower extremities with rest pain	440.3	I70.323
Atherosclerosis of unspecified type of bypass graft(s) of the extremities with rest pain, bilateral legs	440.3	I70.323
Athscl unsp type bypass of the extrm w rest pain, bi legs	440.3	I70.323
Atherosclerosis of unspecified type of bypass graft(s) of the extremities with rest pain, bilateral legs		I70.323

© 2021 Eberly LA et al. *JAMA Network Open*.

Athscl unsp type bypass of the extrm w rest pain, oth extrm	440.3	I70.328
Atherosclerosis of bypass graft of other extremity with rest pain	440.3	I70.328
Atherosclerosis of unspecified type of bypass graft(s) of the extremities with rest pain, other extremity	440.3	I70.328
Atherosclerosis of unspecified type of bypass graft(s) of the extremities with rest pain, other extremity		I70.328
Atherosclerosis of bypass graft of extremity with rest pain	440.3	I70.329
Atherosclerosis of bypass graft of lower extremity with rest pain	440.3	I70.329
Atherosclerosis of unspecified type of bypass graft(s) of the extremities with rest pain, unspecified extremity	440.3	I70.329
Atherosclerosis of bypass graft of lower extremity with rest pain, unspecified laterality	440.3	I70.329
Atherosclerosis of bypass graft of extremity with rest pain, unspecified extremity	440.3	I70.329
Athscl unsp type bypass of the extrm w rest pain, unsp extrm	440.3	I70.329
Atherosclerosis of unspecified type of bypass graft(s) of the extremities with rest pain, unspecified extremity		I70.329
Atherosclerosis of unspecified type of bypass graft(s) of the right leg with ulceration		I70.33
Atherosclerosis of bypass graft of right leg with ulceration of thigh	440.3	I70.331
Atherosclerosis of bypass graft of right lower extremity with ulceration of thigh	440.3	I70.331

© 2021 Eberly LA et al. *JAMA Network Open*.

Atherosclerosis of unspecified type of bypass graft(s) of the right leg with ulceration of thigh	440.3	I70.331
Athscl unsp type bypass of the right leg w ulcer of thigh	440.3	I70.331
Atherosclerosis of unspecified type of bypass graft(s) of the right leg with ulceration of thigh		I70.331
Atherosclerosis of bypass graft of both lower extremities with bilateral ulceration of thighs	440.30, 707.11	I70.331, I70.341
Atherosclerosis of bypass graft of right leg with ulceration of calf	440.3	I70.332
Atherosclerosis of bypass graft of right lower extremity with ulceration of calf	440.3	I70.332
Atherosclerosis of unspecified type of bypass graft(s) of the right leg with ulceration of calf	440.3	I70.332
Athscl unsp type bypass of the right leg w ulcer of calf	440.3	I70.332
Atherosclerosis of unspecified type of bypass graft(s) of the right leg with ulceration of calf		I70.332
Atherosclerosis of bypass graft of both lower extremities with bilateral ulceration of calves	440.30, 707.12	I70.332, I70.342
Atherosclerosis of bypass graft of right leg with ulceration of ankle	440.3	I70.333
Atherosclerosis of bypass graft of right lower extremity with ulceration of ankle	440.3	I70.333
Atherosclerosis of unspecified type of bypass graft(s) of the right leg with ulceration of ankle	440.3	I70.333

© 2021 Eberly LA et al. *JAMA Network Open*.

Athscl unsp type bypass of the right leg w ulcer of ankle	440.3	I70.333
Atherosclerosis of unspecified type of bypass graft(s) of the right leg with ulceration of ankle		I70.333
Atherosclerosis of bypass graft of both lower extremities with bilateral ulceration of ankles	440.30, 707.13	I70.333, I70.343
Athscl unsp type bypass of r leg w ulcer of heel and midft	440.3	I70.334
Atherosclerosis of bypass graft of right lower extremity with ulceration of heel	440.30, 707.14	I70.334
Atherosclerosis of bypass graft of right lower extremity with ulceration of midfoot	440.30, 707.14	I70.334
Atherosclerosis of unspecified type of bypass graft(s) of the right leg with ulceration of heel and midfoot	440.3	I70.334
Atherosclerosis of unspecified type of bypass graft(s) of the right leg with ulceration of heel and midfoot		I70.334
Atherosclerosis of bypass graft of both lower extremities with bilateral ulceration of heels	440.30, 707.14	I70.334, I70.344
Atherosclerosis of bypass graft of both lower extremities with bilateral ulceration of midfeet	440.30, 707.14	I70.334, I70.344
Athscl unsp type bypass of right leg w ulcer oth prt foot	440.3	I70.335
Atherosclerosis of bypass graft of right lower extremity with ulceration of other part of foot	440.3	I70.335
Atherosclerosis of unspecified type of bypass graft(s) of the right leg with ulceration of other part of foot	440.3	I70.335

© 2021 Eberly LA et al. *JAMA Network Open*.

Atherosclerosis of unspecified type of bypass graft(s) of the right leg with ulceration of other part of foot		I70.335
Atherosclerosis of bypass graft of both lower extremities with bilateral ulceration of other part of feet	440.30, 707.15	I70.335, I70.345
Athscl unsp type bypass of right leg w ulcer oth prt low leg	440.3	I70.338
Atherosclerosis of bypass graft of right lower extremity with ulceration of other part of lower leg	440.3	I70.338
Atherosclerosis of unspecified type of bypass graft(s) of the right leg with ulceration of other part of lower leg	440.3	I70.338
Atherosclerosis of unspecified type of bypass graft(s) of the right leg with ulceration of other part of lower leg		I70.338
Atherosclerosis of bypass graft of both lower extremities with bilateral ulceration of other part of lower legs	440.30, 707.19	I70.338, I70.348
Atherosclerosis of bypass graft of right leg with ulceration	440.30, 707.10	I70.339
Atherosclerosis of bypass graft of right lower extremity with ulceration	440.30, 707.10	I70.339
Atherosclerosis of unspecified type of bypass graft(s) of the right leg with ulceration of unspecified site	440.30, 707.10	I70.339
Atherosclerosis of bypass graft of right lower extremity with ulceration, unspecified ulceration site	440.30, 707.10	I70.339
Athscl unsp type bypass of right leg w ulcer of unsp site	440.30, 707.10	I70.339
Atherosclerosis of unspecified type of bypass graft(s) of the right leg with ulceration of unspecified site		I70.339

© 2021 Eberly LA et al. *JAMA Network Open*.

Atherosclerosis of bypass graft of both lower extremities with bilateral ulceration	440.30, 707.10	I70.339, I70.349
Atherosclerosis of bypass graft of both lower extremities with bilateral ulceration, unspecified ulceration site	440.30, 707.10	I70.339, I70.349
Atherosclerosis of unspecified type of bypass graft(s) of the left leg with ulceration		I70.34
Atherosclerosis of bypass graft of left leg with ulceration of thigh	440.30, 707.11	I70.341
Atherosclerosis of bypass graft of left lower extremity with ulceration of thigh	440.30, 707.11	I70.341
Atherosclerosis of unspecified type of bypass graft(s) of the left leg with ulceration of thigh	440.30, 707.11	I70.341
Athscl unsp type bypass of the left leg w ulcer of thigh	440.30, 707.11	I70.341
Atherosclerosis of unspecified type of bypass graft(s) of the left leg with ulceration of thigh		I70.341
Atherosclerosis of bypass graft of left leg with ulceration of calf	440.30, 707.12	I70.342
Atherosclerosis of bypass graft of left lower extremity with ulceration of calf	440.30, 707.12	I70.342
Atherosclerosis of unspecified type of bypass graft(s) of the left leg with ulceration of calf	440.30, 707.12	I70.342
Athscl unsp type bypass of the left leg w ulceration of calf	440.30, 707.12	I70.342
Atherosclerosis of unspecified type of bypass graft(s) of the left leg with ulceration of calf		I70.342

© 2021 Eberly LA et al. *JAMA Network Open*.

Atherosclerosis of bypass graft of left leg with ulceration of ankle	440.3	I70.343
Atherosclerosis of bypass graft of left lower extremity with ulceration of ankle	440.3	I70.343
Atherosclerosis of unspecified type of bypass graft(s) of the left leg with ulceration of ankle	440.3	I70.343
Athscl unsp type bypass of the left leg w ulcer of ankle	440.3	I70.343
Atherosclerosis of unspecified type of bypass graft(s) of the left leg with ulceration of ankle		I70.343
Athscl unsp type bypass of left leg w ulc of heel and midft	440.3	I70.344
Atherosclerosis of bypass graft of left lower extremity with ulceration of heel	440.30, 707.14	I70.344
Atherosclerosis of bypass graft of left lower extremity with ulceration of midfoot	440.30, 707.14	I70.344
Atherosclerosis of unspecified type of bypass graft(s) of the left leg with ulceration of heel and midfoot	440.3	I70.344
Atherosclerosis of unspecified type of bypass graft(s) of the left leg with ulceration of heel and midfoot		I70.344
Athscl unsp type bypass of the left leg w ulcer oth prt foot	440.3	I70.345
Atherosclerosis of bypass graft of left lower extremity with ulceration of other part of foot	440.3	I70.345
Atherosclerosis of unspecified type of bypass graft(s) of the left leg with ulceration of other part of foot	440.3	I70.345

© 2021 Eberly LA et al. *JAMA Network Open*.

Atherosclerosis of unspecified type of bypass graft(s) of the left leg with ulceration of other part of foot		I70.345
Athscl unsp type bypass of left leg w ulcer oth prt low leg	440.3	I70.348
Atherosclerosis of bypass graft of left lower extremity with ulceration of other part of lower leg	440.3	I70.348
Atherosclerosis of unspecified type of bypass graft(s) of the left leg with ulceration of other part of lower leg	440.3	I70.348
Atherosclerosis of unspecified type of bypass graft(s) of the left leg with ulceration of other part of lower leg		I70.348
Atherosclerosis of bypass graft of left leg with ulceration	440.3	I70.349
Atherosclerosis of bypass graft of left lower extremity with ulceration	440.3	I70.349
Atherosclerosis of unspecified type of bypass graft(s) of the left leg with ulceration of unspecified site	440.3	I70.349
Atherosclerosis of bypass graft of left lower extremity with ulceration, unspecified ulceration site	440.3	I70.349
Athscl unsp type bypass of the left leg w ulcer of unsp site	440.3	I70.349
Atherosclerosis of unspecified type of bypass graft(s) of the left leg with ulceration of unspecified site		I70.349
Athscl unsp type bypass graft(s) of extremity w ulceration	440.30, 707.9	I70.35
Atherosclerosis of bypass graft of lower extremity with ulceration	443.9, 707.10	I70.35

© 2021 Eberly LA et al. *JAMA Network Open*.

Atherosclerosis of bypass graft of lower extremity with ulceration of calf	443.9, 707.12	I70.35
Atherosclerosis of bypass graft of lower extremity with ulceration of thigh	443.9, 707.11	I70.35
Atherosclerosis of bypass graft of lower extremity with ulceration of heel	443.9, 707.14	I70.35
Atherosclerosis of bypass graft of lower extremity with ulceration of midfoot	443.9, 707.14	I70.35
Atherosclerosis of bypass graft of lower extremity with ulceration of ankle	443.9, 707.13	I70.35
Atherosclerosis of bypass graft of extremity with ulceration	440.30, 707.9	I70.35
Atherosclerosis of bypass graft of other extremity with ulceration	440.30, 707.9	I70.35
Atherosclerosis of unspecified type of bypass graft(s) of other extremity with ulceration	440.30, 707.9	I70.35
Atherosclerosis of bypass graft of lower extremity with ulceration of other part of lower leg	443.9, 707.19	I70.35
Atherosclerosis of bypass graft of lower extremity with ulceration of other part of foot	443.9, 707.15	I70.35
Atherosclerosis of bypass graft of lower extremity with ulceration of other part of foot, unspecified laterality	443.9, 707.15	I70.35
Atherosclerosis of bypass graft of lower extremity with ulceration of ankle, unspecified laterality	443.9, 707.13	I70.35
Atherosclerosis of bypass graft of lower extremity with ulceration of thigh, unspecified laterality	443.9, 707.11	I70.35
Atherosclerosis of bypass graft of lower extremity with ulceration of other part of lower leg, unspecified laterality	443.9, 707.19	I70.35
Atherosclerosis of bypass graft of lower extremity with ulceration of calf, unspecified laterality	443.9, 707.12	I70.35

© 2021 Eberly LA et al. *JAMA Network Open*.

Atherosclerosis of bypass graft of lower extremity with ulceration of midfoot, unspecified laterality	443.9, 707.14	I70.35
Atherosclerosis of bypass graft of lower extremity with ulceration, unspecified laterality, unspecified ulceration site	443.9, 707.10	I70.35
Atherosclerosis of bypass graft of lower extremity with ulceration of heel, unspecified laterality	443.9, 707.14	I70.35
Atherosclerosis of bypass graft of extremity with ulceration, unspecified extremity	440.30, 707.9	I70.35
Atherosclerosis of unspecified type of bypass graft(s) of other extremity with ulceration		I70.35
Atherosclerosis of unspecified type of bypass graft(s) of the extremities with gangrene		I70.36
Atherosclerosis of bypass graft of right lower extremity with gangrene	440.3	I70.361
Atherosclerosis of unspecified type of bypass graft(s) of the extremities with gangrene, right leg	440.3	I70.361
Athscl unsp type bypass of the extrm w gangrene, right leg	440.3	I70.361
Atherosclerosis of unspecified type of bypass graft(s) of the extremities with gangrene, right leg		I70.361
Atherosclerosis of bypass graft of left lower extremity with gangrene	440.3	I70.362
Atherosclerosis of unspecified type of bypass graft(s) of the extremities with gangrene, left leg	440.3	I70.362
Athscl unsp type bypass of the extrm w gangrene, left leg	440.3	I70.362

© 2021 Eberly LA et al. *JAMA Network Open*.

Atherosclerosis of unspecified type of bypass graft(s) of the extremities with gangrene, left leg		I70.362
Atherosclerosis of bypass graft of both legs with gangrene	440.30, 785.4	I70.363
Atherosclerosis of bypass graft of both lower extremities with gangrene	440.30, 785.4	I70.363
Atherosclerosis of unspecified type of bypass graft(s) of the extremities with gangrene, bilateral legs	440.30, 785.4	I70.363
Athscl unsp type bypass of the extrm w gangrene, bi legs	440.30, 785.4	I70.363
Atherosclerosis of unspecified type of bypass graft(s) of the extremities with gangrene, bilateral legs		I70.363
Athscl unsp type bypass of the extrm w gangrene, oth extrm	440.3	I70.368
Atherosclerosis of bypass graft of other extremity with gangrene	440.3	I70.368
Atherosclerosis of unspecified type of bypass graft(s) of the extremities with gangrene, other extremity	440.3	I70.368
Atherosclerosis of unspecified type of bypass graft(s) of the extremities with gangrene, other extremity		I70.368
Atherosclerosis of bypass graft of extremity with gangrene	440.3	I70.369
Atherosclerosis of bypass graft of lower extremity with gangrene	440.30, 785.4	I70.369
Atherosclerosis of unspecified type of bypass graft(s) of the extremities with gangrene, unspecified extremity	440.3	I70.369
Atherosclerosis of bypass graft of lower extremity with gangrene, unspecified laterality	440.30, 785.4	I70.369

© 2021 Eberly LA et al. *JAMA Network Open*.

Atherosclerosis of bypass graft of extremity with gangrene, unspecified extremity	440.3	I70.369
Athscl unsp type bypass of the extrm w gangrene, unsp extrm	440.3	I70.369
Atherosclerosis of unspecified type of bypass graft(s) of the extremities with gangrene, unspecified extremity		I70.369
Other atherosclerosis of unspecified type of bypass graft(s) of the extremities		I70.39
Oth athscl unsp type bypass of the extremities, right leg	440.3	I70.391
Other atherosclerosis of unspecified type of bypass graft(s) of the extremities, right leg	440.3	I70.391
Atherosclerosis of bypass graft of right lower extremity with other clinical manifestation	440.3	I70.391
Other atherosclerosis of unspecified type of bypass graft(s) of the extremities, right leg		I70.391
Oth athscl unsp type bypass of the extremities, left leg	440.3	I70.392
Other atherosclerosis of unspecified type of bypass graft(s) of the extremities, left leg	440.3	I70.392
Atherosclerosis of bypass graft of left lower extremity with other clinical manifestation	440.3	I70.392
Other atherosclerosis of unspecified type of bypass graft(s) of the extremities, left leg		I70.392
Oth athscl unsp type bypass of the extrm, bilateral legs	440.3	I70.393
Other atherosclerosis of unspecified type of bypass graft(s) of the extremities, bilateral legs	440.3	I70.393

© 2021 Eberly LA et al. *JAMA Network Open*.

Atherosclerosis of bypass graft of both lower extremities with other clinical manifestation	440.3	I70.393
Other atherosclerosis of unspecified type of bypass graft(s) of the extremities, bilateral legs		I70.393
Oth athscl unsp type bypass of the extrm, oth extremity	440.3	I70.398
Other atherosclerosis of unspecified type of bypass graft(s) of the extremities, other extremity	440.3	I70.398
Atherosclerosis of bypass graft of other extremity with other clinical manifestation	440.3	I70.398
Other atherosclerosis of unspecified type of bypass graft(s) of the extremities, other extremity		I70.398
Oth athscl unsp type bypass of the extrm, unsp extremity	440.3	I70.399
Other atherosclerosis of unspecified type of bypass graft(s) of the extremities, unspecified extremity	440.3	I70.399
Atherosclerosis of bypass graft of extremity with other clinical manifestation	440.3	I70.399
Atherosclerosis of bypass graft of lower extremity with other clinical manifestation	440.3	I70.399
Atherosclerosis of bypass graft of lower extremity with other clinical manifestation, unspecified laterality	440.3	I70.399
Atherosclerosis of bypass graft of extremity with other clinical manifestation, unspecified extremity	440.3	I70.399
Other atherosclerosis of unspecified type of bypass graft(s) of the extremities, unspecified extremity		I70.399

© 2021 Eberly LA et al. *JAMA Network Open*.

Atherosclerosis of autologous vein bypass graft(s) of the extremities		I70.4
Unspecified atherosclerosis of autologous vein bypass graft(s) of the extremities		I70.40
Atherosclerosis of autologous vein bypass graft of right leg	440.31	I70.401
Atherosclerosis of autologous vein bypass graft of right lower extremity	440.31	I70.401
Unspecified atherosclerosis of autologous vein bypass graft(s) of the extremities, right leg	440.31	I70.401
Atherosclerosis of autologous vein bypass graft of right lower extremity, with unspecified presence of clinical manifestation	440.31	I70.401
Atherosclerosis of autologous bypass graft of right lower extremity	440.31	I70.401
Unsp athscl autologous vein bypass of the extrm, right leg	440.31	I70.401
Unspecified atherosclerosis of autologous vein bypass graft(s) of the extremities, right leg		I70.401
Atherosclerosis of autologous vein bypass graft of left leg	440.31	I70.402
Atherosclerosis of autologous vein bypass graft of left lower extremity	440.31	I70.402
Unspecified atherosclerosis of autologous vein bypass graft(s) of the extremities, left leg	440.31	I70.402
Atherosclerosis of autologous vein bypass graft of left lower extremity, with unspecified presence of clinical manifestation	440.31	I70.402

© 2021 Eberly LA et al. *JAMA Network Open*.

Atherosclerosis of autologous bypass graft of left lower extremity	440.31	I70.402
Unsp athscl autologous vein bypass of the extrm, left leg	440.31	I70.402
Unspecified atherosclerosis of autologous vein bypass graft(s) of the extremities, left leg		I70.402
Atherosclerosis of autologous vein bypass graft of both legs	440.31	I70.403
Atherosclerosis of autologous vein bypass graft of both lower extremities	440.31	I70.403
Unspecified atherosclerosis of autologous vein bypass graft(s) of the extremities, bilateral legs	440.31	I70.403
Atherosclerosis of autologous vein bypass graft of both lower extremities, with unspecified presence of clinical manifestation	440.31	I70.403
Atherosclerosis of autologous bypass graft of both lower extremities	440.31	I70.403
Unsp athscl autol vein bypass of the extrm, bilateral legs	440.31	I70.403
Unspecified atherosclerosis of autologous vein bypass graft(s) of the extremities, bilateral legs		I70.403
Unsp athscl autol vein bypass of the extrm, oth extremity	440.31	I70.408
Atherosclerosis of autologous vein bypass graft of other extremity	440.31	I70.408
Unspecified atherosclerosis of autologous vein bypass graft(s) of the extremities, other extremity	440.31	I70.408

© 2021 Eberly LA et al. *JAMA Network Open*.

Atherosclerosis of autologous vein bypass graft of other extremity, with unspecified presence of clinical manifestation	440.31	I70.408
Unspecified atherosclerosis of autologous vein bypass graft(s) of the extremities, other extremity		I70.408
Atherosclerosis of autologous vein bypass graft of extremities	440.31	I70.409
Atherosclerosis of autologous vein bypass graft of extremity	440.31	I70.409
Atherosclerosis of autologous vein bypass graft of limb	440.31	I70.409
Atherosclerosis of autologous vein bypass graft of lower extremity	440.31	I70.409
Unspecified atherosclerosis of autologous vein bypass graft(s) of the extremities, unspecified extremity	440.31	I70.409
Atherosclerosis of autologous vein bypass graft of lower extremity, unspecified laterality, with unspecified presence of clinical manifestation	440.31	I70.409
Atherosclerosis of autologous vein bypass graft of extremity, unspecified extremity, with unspecified presence of clinical manifestation	440.31	I70.409
Unsp athscl autol vein bypass of the extrm, unsp extremity	440.31	I70.409
Unspecified atherosclerosis of autologous vein bypass graft(s) of the extremities, unspecified extremity		I70.409
Atherosclerosis of autologous vein bypass graft(s) of the extremities with intermittent claudication		I70.41
Atherosclerosis of autologous vein bypass graft of right lower extremity with intermittent claudication	440.31	I70.411

© 2021 Eberly LA et al. *JAMA Network Open*.

Atherosclerosis of autologous vein bypass graft(s) of the extremities with intermittent claudication, right leg	440.31	I70.411
Athscl autol vein bypass of extrm w intrmt claud, right leg	440.31	I70.411
Atherosclerosis of autologous vein bypass graft(s) of the extremities with intermittent claudication, right leg		I70.411
Atherosclerosis of autologous vein bypass graft of left lower extremity with intermittent claudication	440.31	I70.412
Atherosclerosis of autologous vein bypass graft(s) of the extremities with intermittent claudication, left leg	440.31	I70.412
Athscl autol vein bypass of extrm w intrmt claud, left leg	440.31	I70.412
Atherosclerosis of autologous vein bypass graft(s) of the extremities with intermittent claudication, left leg		I70.412
Atherosclerosis of autologous vein bypass graft of both lower extremities with intermittent claudication	440.31	I70.413
Atherosclerosis of autologous vein bypass graft(s) of the extremities with intermittent claudication, bilateral legs	440.31	I70.413
Athscl autol vein bypass of extrm w intrmt claud, bi legs	440.31	I70.413
Atherosclerosis of autologous vein bypass graft(s) of the extremities with intermittent claudication, bilateral legs		I70.413
Athscl autol vein bypass of extrm w intrmt claud, oth extrm	440.31	I70.418
Atherosclerosis of autologous vein bypass graft of other extremity with intermittent claudication	440.31	I70.418

© 2021 Eberly LA et al. *JAMA Network Open*.

Atherosclerosis of autologous vein bypass graft(s) of the extremities with intermittent claudication, other extremity	440.31	I70.418
Atherosclerosis of autologous vein bypass graft(s) of the extremities with intermittent claudication, other extremity		I70.418
Atherosclerosis of autologous vein bypass graft of extremity with intermittent claudication	440.31	I70.419
Atherosclerosis of autologous vein bypass graft of lower extremity with intermittent claudication	440.31, 443.9	I70.419
Atherosclerosis of autologous vein bypass graft(s) of the extremities with intermittent claudication, unspecified extremity	440.31	I70.419
Atherosclerosis of autologous vein bypass graft of lower extremity with intermittent claudication, unspecified laterality	440.31, 443.9	I70.419
Atherosclerosis of autologous vein bypass graft of extremity with intermittent claudication, unspecified extremity	440.31	I70.419
Athscl autol vein bypass of extrm w intrmt claud, unsp extrm	440.31	I70.419
Atherosclerosis of autologous vein bypass graft(s) of the extremities with intermittent claudication, unspecified extremity		I70.419
Atherosclerosis of autologous vein bypass graft(s) of the extremities with rest pain		I70.42
Atheroscler of autologous vein bypass graft of right leg w/rest pain	440.31	I70.421
Atherosclerosis of autologous vein bypass graft of right lower extremity with rest pain	440.31	I70.421

© 2021 Eberly LA et al. *JAMA Network Open*.

Atherosclerosis of autologous vein bypass graft(s) of the extremities with rest pain, right leg	440.31	I70.421
Athscl autol vein bypass of the extrm w rest pain, right leg	440.31	I70.421
Atherosclerosis of autologous vein bypass graft(s) of the extremities with rest pain, right leg		I70.421
Atheroscler of autologous vein bypass graft of left leg with rest pain	440.31	I70.422
Atherosclerosis of autologous vein bypass graft of left lower extremity with rest pain	440.31	I70.422
Atherosclerosis of autologous vein bypass graft(s) of the extremities with rest pain, left leg	440.31	I70.422
Athscl autol vein bypass of the extrm w rest pain, left leg	440.31	I70.422
Atherosclerosis of autologous vein bypass graft(s) of the extremities with rest pain, left leg		I70.422
Atheroscler of autologous vein bypass graft of both legs w/rest pain	440.31	I70.423
Atherosclerosis of autologous vein bypass graft of both lower extremities with rest pain	440.31	I70.423
Atherosclerosis of autologous vein bypass graft(s) of the extremities with rest pain, bilateral legs	440.31	I70.423
Athscl autol vein bypass of the extrm w rest pain, bi legs	440.31	I70.423
Atherosclerosis of autologous vein bypass graft(s) of the extremities with rest pain, bilateral legs		I70.423

© 2021 Eberly LA et al. *JAMA Network Open*.

Athscl autol vein bypass of the extrm w rest pain, oth extrm	440.31	I70.428
Atherosclerosis of autologous vein bypass graft of other extremity with rest pain	440.31	I70.428
Atherosclerosis of autologous vein bypass graft(s) of the extremities with rest pain, other extremity	440.31	I70.428
Atherosclerosis of autologous vein bypass graft(s) of the extremities with rest pain, other extremity		I70.428
Atheroscler of autologous vein bypass graft of extremity w/rest pain	440.31	I70.429
Atherosclerosis of autologous vein bypass graft of extremity with rest pain	440.31	I70.429
Atherosclerosis of autologous vein bypass graft of lower extremity with rest pain	440.31	I70.429
Atherosclerosis of autologous vein bypass graft(s) of the extremities with rest pain, unspecified extremity	440.31	I70.429
Atherosclerosis of autologous vein bypass graft of lower extremity with rest pain, unspecified laterality	440.31	I70.429
Atherosclerosis of autologous vein bypass graft of extremity with rest pain, unspecified extremity	440.31	I70.429
Athscl autol vein bypass of extrm w rest pain, unsp extrm	440.31	I70.429
Atherosclerosis of autologous vein bypass graft(s) of the extremities with rest pain, unspecified extremity		I70.429
Atherosclerosis of autologous vein bypass graft(s) of the right leg with ulceration		I70.43
Atheroscler autologous vein bypass graft right leg w/ulceration thigh	440.31, 707.11	I70.431

© 2021 Eberly LA et al. *JAMA Network Open*.

Atherosclerosis of autologous vein bypass graft of right lower extremity with ulceration of thigh	440.31, 707.11	I70.431
Atherosclerosis of autologous vein bypass graft(s) of the right leg with ulceration of thigh	440.31, 707.11	I70.431
Athscl autol vein bypass of the right leg w ulcer of thigh	440.31, 707.11	I70.431
Atherosclerosis of autologous vein bypass graft(s) of the right leg with ulceration of thigh		I70.431
Atherosclerosis of autologous vein bypass graft of both lower extremities with bilateral ulceration of thighs	440.31, 707.11	I70.431, I70.441
Atheroscler autologous vein bypass graft right leg w/ulceration calf	440.31, 707.12	I70.432
Atherosclerosis of autologous vein bypass graft of right lower extremity with ulceration of calf	440.31, 707.12	I70.432
Atherosclerosis of autologous vein bypass graft(s) of the right leg with ulceration of calf	440.31, 707.12	I70.432
Athscl autol vein bypass of the right leg w ulcer of calf	440.31, 707.12	I70.432
Atherosclerosis of autologous vein bypass graft(s) of the right leg with ulceration of calf		I70.432
Atherosclerosis of autologous vein bypass graft of both lower extremities with bilateral ulceration of calves	440.31, 707.12	I70.432, I70.442
Atheroscler autologous vein bypass graft right leg w/ulceration ankle	440.31	I70.433
Atherosclerosis of autologous vein bypass graft of right lower extremity with ulceration of ankle	440.31	I70.433

© 2021 Eberly LA et al. *JAMA Network Open*.

Atherosclerosis of autologous vein bypass graft(s) of the right leg with ulceration of ankle	440.31	I70.433
Athscl autol vein bypass of the right leg w ulcer of ankle	440.31	I70.433
Atherosclerosis of autologous vein bypass graft(s) of the right leg with ulceration of ankle		I70.433
Atherosclerosis of autologous vein bypass graft of both lower extremities with bilateral ulceration of ankles	440.31, 707.13	I70.433, I70.443
Athscl autol vein bypass of r leg w ulcer of heel and midft	440.31	I70.434
Atherosclerosis of autologous vein bypass graft of right lower extremity with ulceration of midfoot	440.31, 707.14	I70.434
Atherosclerosis of autologous vein bypass graft of right lower extremity with ulceration of heel	440.31, 707.14	I70.434
Atherosclerosis of autologous vein bypass graft(s) of the right leg with ulceration of heel and midfoot	440.31	I70.434
Atherosclerosis of autologous vein bypass graft(s) of the right leg with ulceration of heel and midfoot		I70.434
Atherosclerosis of autologous vein bypass graft of both lower extremities with bilateral ulceration of heels	440.31, 707.14	I70.434, I70.444
Atherosclerosis of autologous vein bypass graft of both lower extremities with bilateral ulceration of midfeet	440.31, 707.14	I70.434, I70.444
Athscl autol vein bypass of right leg w ulcer oth prt foot	440.31	I70.435

© 2021 Eberly LA et al. *JAMA Network Open*.

Atherosclerosis of autologous vein bypass graft of right lower extremity with ulceration of other part of foot	440.31	I70.435
Atherosclerosis of autologous vein bypass graft(s) of the right leg with ulceration of other part of foot	440.31	I70.435
Atherosclerosis of autologous vein bypass graft(s) of the right leg with ulceration of other part of foot		I70.435
Atherosclerosis of autologous vein bypass graft of both lower extremities with bilateral ulceration of other part of feet	440.31, 707.15	I70.435, I70.445
Athscl autol vein bypass of r leg w ulcer oth prt low leg	440.31	I70.438
Atherosclerosis of autologous vein bypass graft of right lower extremity with ulceration of other part of lower leg	440.31	I70.438
Atherosclerosis of autologous vein bypass graft(s) of the right leg with ulceration of other part of lower leg	440.31	I70.438
Atherosclerosis of autologous vein bypass graft(s) of the right leg with ulceration of other part of lower leg		I70.438
Atherosclerosis of autologous vein bypass graft of both lower extremities with bilateral ulceration of other part of lower legs	440.31, 707.19	I70.438, I70.448
Atheroscler of autologous vein bypass graft of right leg w/ulceration	440.31, 707.10	I70.439
Atherosclerosis of autologous vein bypass graft of right lower extremity with ulceration	440.31, 707.10	I70.439
Atherosclerosis of autologous vein bypass graft(s) of the right leg with ulceration of unspecified site	440.31, 707.10	I70.439

© 2021 Eberly LA et al. *JAMA Network Open*.

Atherosclerosis of autologous vein bypass graft of right lower extremity with ulceration, unspecified ulceration site	440.31, 707.10	I70.439
Athscl autol vein bypass of right leg w ulcer of unsp site	440.31, 707.10	I70.439
Atherosclerosis of autologous vein bypass graft(s) of the right leg with ulceration of unspecified site		I70.439
Atherosclerosis of autologous vein bypass graft of both lower extremities with bilateral ulceration	440.23, 707.10	I70.439, I70.449
Atherosclerosis of autologous vein bypass graft of both lower extremities with bilateral ulceration, unspecified ulceration site	440.23, 707.10	I70.439, I70.449
Atherosclerosis of autologous vein bypass graft(s) of the left leg with ulceration		I70.44
Atheroscler autologous vein bypass graft left leg w/ulceration thigh	440.31, 707.11	I70.441
Atherosclerosis of autologous vein bypass graft of left lower extremity with ulceration of thigh	440.31, 707.11	I70.441
Atherosclerosis of autologous vein bypass graft(s) of the left leg with ulceration of thigh	440.31, 707.11	I70.441
Athscl autol vein bypass of the left leg w ulcer of thigh	440.31, 707.11	I70.441
Atherosclerosis of autologous vein bypass graft(s) of the left leg with ulceration of thigh		I70.441
Atheroscler autologous vein bypass graft left leg w/ulceration calf	440.31, 707.12	I70.442

Atherosclerosis of autologous vein bypass graft of left lower extremity with ulceration of calf	440.31, 707.12	I70.442
Atherosclerosis of autologous vein bypass graft(s) of the left leg with ulceration of calf	440.31, 707.12	I70.442
Athscl autol vein bypass of the left leg w ulcer of calf	440.31, 707.12	I70.442
Atherosclerosis of autologous vein bypass graft(s) of the left leg with ulceration of calf		I70.442
Atheroscler autologous vein bypass graft left leg w/ulceration ankle	440.31, 707.13	I70.443
Atherosclerosis of autologous vein bypass graft of left lower extremity with ulceration of ankle	440.31, 707.13	I70.443
Atherosclerosis of autologous vein bypass graft(s) of the left leg with ulceration of ankle	440.31, 707.13	I70.443
Athscl autol vein bypass of the left leg w ulcer of ankle	440.31, 707.13	I70.443
Atherosclerosis of autologous vein bypass graft(s) of the left leg with ulceration of ankle		I70.443
Athscl autol vein bypass of left leg w ulc of heel and midft	440.31	I70.444
Atherosclerosis of autologous vein bypass graft of left lower extremity with ulceration of midfoot	440.31, 707.14	I70.444
Atherosclerosis of autologous vein bypass graft of left lower extremity with ulceration of heel	440.31, 707.14	I70.444
Atherosclerosis of autologous vein bypass graft(s) of the left leg with ulceration of heel and midfoot	440.31	I70.444

© 2021 Eberly LA et al. *JAMA Network Open*.

Atherosclerosis of autologous vein bypass graft(s) of the left leg with ulceration of heel and midfoot		I70.444
Athscl autol vein bypass of left leg w ulcer oth prt foot	440.31	I70.445
Atherosclerosis of autologous vein bypass graft of left lower extremity with ulceration of other part of foot	440.31	I70.445
Atherosclerosis of autologous vein bypass graft(s) of the left leg with ulceration of other part of foot	440.31	I70.445
Atherosclerosis of autologous vein bypass graft(s) of the left leg with ulceration of other part of foot		I70.445
Athscl autol vein bypass of left leg w ulcer oth prt low leg	440.31	I70.448
Atherosclerosis of autologous vein bypass graft of left lower extremity with ulceration of other part of lower leg	440.31	I70.448
Atherosclerosis of autologous vein bypass graft(s) of the left leg with ulceration of other part of lower leg	440.31	I70.448
Atherosclerosis of autologous vein bypass graft(s) of the left leg with ulceration of other part of lower leg		I70.448
Atheroscler of autologous vein bypass graft of left leg w/ulceration	440.31, 707.10	I70.449
Atherosclerosis of autologous vein bypass graft of left lower extremity with ulceration	440.31, 707.10	I70.449
Atherosclerosis of autologous vein bypass graft(s) of the left leg with ulceration of unspecified site	440.31, 707.10	I70.449

© 2021 Eberly LA et al. *JAMA Network Open*.

Atherosclerosis of autologous vein bypass graft of left lower extremity with ulceration, unspecified ulceration site	440.31, 707.10	I70.449
Athscl autol vein bypass of left leg w ulcer of unsp site	440.31, 707.10	I70.449
Atherosclerosis of autologous vein bypass graft(s) of the left leg with ulceration of unspecified site		I70.449
Athscl autologous vein bypass of extremity w ulceration	440.31, 707.9	I70.45
Atherosclerosis of autologous vein bypass graft of lower extremity with ulceration	440.31, 707.10	I70.45
Atherosclerosis of autologous vein bypass graft of lower extremity with ulceration of heel	440.31, 707.14	I70.45
Atherosclerosis of autologous vein bypass graft of lower extremity with ulceration of ankle	440.31, 707.13	I70.45
Atherosclerosis of autologous vein bypass graft of lower extremity with ulceration of thigh	440.31, 707.11	I70.45
Atherosclerosis of autologous vein bypass graft of lower extremity with ulceration of midfoot	440.31, 707.14	I70.45
Atherosclerosis of autologous vein bypass graft of lower extremity with ulceration of calf	440.31, 707.12	I70.45
Atherosclerosis of autologous vein bypass graft of extremity with ulceration	440.31, 707.9	I70.45
Atherosclerosis of autologous vein bypass graft of other extremity with ulceration	440.31	I70.45
Atherosclerosis of autologous vein bypass graft(s) of other extremity with ulceration	440.31	I70.45

© 2021 Eberly LA et al. *JAMA Network Open*.

Atherosclerosis of autologous vein bypass graft of lower extremity with ulceration of other part of lower leg	440.31, 707.19	I70.45
Atherosclerosis of autologous vein bypass graft of lower extremity with ulceration of other part of foot	440.31, 707.15	I70.45
Atherosclerosis of autologous vein bypass graft of lower extremity with ulceration, unspecified laterality, unspecified ulceration site	440.31, 707.10	I70.45
Atherosclerosis of autologous vein bypass graft of lower extremity with ulceration of heel, unspecified laterality	440.31, 707.14	I70.45
Atherosclerosis of autologous vein bypass graft of lower extremity with ulceration of other part of foot, unspecified laterality	440.31, 707.15	I70.45
Atherosclerosis of autologous vein bypass graft of lower extremity with ulceration of calf, unspecified laterality	440.31, 707.12	I70.45
Atherosclerosis of autologous vein bypass graft of lower extremity with ulceration of ankle, unspecified laterality	440.31, 707.13	I70.45
Atherosclerosis of autologous vein bypass graft of lower extremity with ulceration of midfoot, unspecified laterality	440.31, 707.14	I70.45
Atherosclerosis of autologous vein bypass graft of lower extremity with ulceration of other part of lower leg, unspecified laterality	440.31, 707.19	I70.45
Atherosclerosis of autologous vein bypass graft of lower extremity with ulceration of thigh, unspecified laterality	440.31, 707.11	I70.45
Atherosclerosis of autologous vein bypass graft of extremity with ulceration, unspecified extremity	440.31, 707.9	I70.45

© 2021 Eberly LA et al. *JAMA Network Open*.

Atherosclerosis of autologous vein bypass graft(s) of other extremity with ulceration		I70.45
Atherosclerosis of autologous vein bypass graft(s) of the extremities with gangrene		I70.46
Atheroscler of autologous vein bypass graft of right leg with gangrene	440.31	I70.461
Atherosclerosis of autologous vein bypass graft of right lower extremity with gangrene	440.31	I70.461
Atherosclerosis of autologous vein bypass graft(s) of the extremities with gangrene, right leg	440.31	I70.461
Athscl autol vein bypass of the extrm w gangrene, right leg	440.31	I70.461
Atherosclerosis of autologous vein bypass graft(s) of the extremities with gangrene, right leg		I70.461
Atheroscler of autologous vein bypass graft of left leg with gangrene	440.31, 785.4	I70.462
Atherosclerosis of autologous vein bypass graft of left lower extremity with gangrene	440.31, 785.4	I70.462
Atherosclerosis of autologous vein bypass graft(s) of the extremities with gangrene, left leg	440.31, 785.4	I70.462
Athscl autol vein bypass of the extrm w gangrene, left leg	440.31, 785.4	I70.462
Atherosclerosis of autologous vein bypass graft(s) of the extremities with gangrene, left leg		I70.462
Atheroscler of autologous vein bypass graft of both legs with gangrene	440.31	I70.463
Atherosclerosis of autologous vein bypass graft of both lower extremities with gangrene	440.31	I70.463

© 2021 Eberly LA et al. *JAMA Network Open*.

Atherosclerosis of autologous vein bypass graft(s) of the extremities with gangrene, bilateral legs	440.31	I70.463
Athscl autol vein bypass of the extrm w gangrene, bi legs	440.31	I70.463
Atherosclerosis of autologous vein bypass graft(s) of the extremities with gangrene, bilateral legs		I70.463
Athscl autol vein bypass of the extrm w gangrene, oth extrm	440.31	I70.468
Atherosclerosis of autologous vein bypass graft of other extremity with gangrene	440.31	I70.468
Atherosclerosis of autologous vein bypass graft(s) of the extremities with gangrene, other extremity	440.31	I70.468
Atherosclerosis of autologous vein bypass graft(s) of the extremities with gangrene, other extremity		I70.468
Atheroscler of autologous vein bypass graft of extremity with gangrene	440.31, 785.4	I70.469
Atherosclerosis of autologous vein bypass graft of extremity with gangrene	440.31, 785.4	I70.469
Atherosclerosis of autologous vein bypass graft of lower extremity with gangrene	440.31, 785.4	I70.469
Atherosclerosis of autologous vein bypass graft(s) of the extremities with gangrene, unspecified extremity	440.31, 785.4	I70.469
Atherosclerosis of autologous vein bypass graft of lower extremity with gangrene, unspecified laterality	440.31, 785.4	I70.469
Atherosclerosis of autologous vein bypass graft of extremity with gangrene, unspecified extremity	440.31, 785.4	I70.469

© 2021 Eberly LA et al. *JAMA Network Open*.

Athscl autol vein bypass of the extrm w gangrene, unsp extrm	440.31, 785.4	I70.469
Atherosclerosis of autologous vein bypass graft(s) of the extremities with gangrene, unspecified extremity		I70.469
Other atherosclerosis of autologous vein bypass graft(s) of the extremities		I70.49
Oth athscl autologous vein bypass of the extrm, right leg	440.31	I70.491
Other atherosclerosis of autologous vein bypass graft(s) of the extremities, right leg	440.31	I70.491
Atherosclerosis of autologous vein bypass graft of right lower extremity with other clinical manifestation	440.31	I70.491
Other atherosclerosis of autologous vein bypass graft(s) of the extremities, right leg		I70.491
Oth athscl autologous vein bypass of the extrm, left leg	440.31	I70.492
Other atherosclerosis of autologous vein bypass graft(s) of the extremities, left leg	440.31	I70.492
Atherosclerosis of autologous vein bypass graft of left lower extremity with other clinical manifestation	440.31	I70.492
Other atherosclerosis of autologous vein bypass graft(s) of the extremities, left leg		I70.492
Oth athscl autol vein bypass of the extrm, bilateral legs	440.31	I70.493
Other atherosclerosis of autologous vein bypass graft(s) of the extremities, bilateral legs	440.31	I70.493
Atherosclerosis of autologous vein bypass graft of both lower extremities with other clinical manifestation	440.31	I70.493

© 2021 Eberly LA et al. *JAMA Network Open*.

Other atherosclerosis of autologous vein bypass graft(s) of the extremities, bilateral legs		I70.493
Oth athscl autol vein bypass of the extrm, oth extremity	440.31	I70.498
Other atherosclerosis of autologous vein bypass graft(s) of the extremities, other extremity	440.31	I70.498
Atherosclerosis of autologous vein bypass graft of other extremity with other clinical manifestation	440.31	I70.498
Other atherosclerosis of autologous vein bypass graft(s) of the extremities, other extremity		I70.498
Oth athscl autol vein bypass of the extrm, unsp extremity	440.31	I70.499
Other atherosclerosis of autologous vein bypass graft(s) of the extremities, unspecified extremity	440.31	I70.499
Atherosclerosis of autologous vein bypass graft of extremity with other clinical manifestation	440.31	I70.499
Atherosclerosis of autologous vein bypass graft of lower extremity with other clinical manifestation	440.31	I70.499
Atherosclerosis of autologous vein bypass graft of lower extremity with other clinical manifestation, unspecified laterality	440.31	I70.499
Atherosclerosis of autologous vein bypass graft of extremity with other clinical manifestation, unspecified extremity	440.31	I70.499
Other atherosclerosis of autologous vein bypass graft(s) of the extremities, unspecified extremity		I70.499

© 2021 Eberly LA et al. *JAMA Network Open*.

Atherosclerosis of nonautologous biological bypass graft(s) of the extremities		I70.5
Unspecified atherosclerosis of nonautologous biological bypass graft(s) of the extremities		I70.50
Atherosclerosis of nonautologous biological bypass graft of right leg	440.32	I70.501
Atherosclerosis of nonautologous biological bypass graft of right lower extremity	440.32	I70.501
Unspecified atherosclerosis of nonautologous biological bypass graft(s) of the extremities, right leg	440.32	I70.501
Atherosclerosis of nonautologous biological bypass graft of right lower extremity, with unspecified presence of clinical manifestation	440.32	I70.501
Unsp athscl nonaut bio bypass of the extremities, right leg	440.32	I70.501
Unspecified atherosclerosis of nonautologous biological bypass graft(s) of the extremities, right leg		I70.501
Atherosclerosis of nonautologous biological bypass graft of left leg	440.32	I70.502
Atherosclerosis of nonautologous biological bypass graft of left lower extremity	440.32	I70.502
Unspecified atherosclerosis of nonautologous biological bypass graft(s) of the extremities, left leg	440.32	I70.502
Atherosclerosis of nonautologous biological bypass graft of left lower extremity, with unspecified presence of clinical manifestation	440.32	I70.502

© 2021 Eberly LA et al. *JAMA Network Open*.

Unsp athscl nonaut bio bypass of the extremities, left leg	440.32	I70.502
Unspecified atherosclerosis of nonautologous biological bypass graft(s) of the extremities, left leg		I70.502
Atherosclerosis of nonautologous biological bypass graft of both legs	440.32	I70.503
Atherosclerosis of nonautologous biological bypass graft of both lower extremities	440.32	I70.503
Unspecified atherosclerosis of nonautologous biological bypass graft(s) of the extremities, bilateral legs	440.32	I70.503
Atherosclerosis of nonautologous biological bypass graft of both lower extremities, with unspecified presence of clinical manifestation	440.32	I70.503
Unsp athscl nonaut bio bypass of the extrm, bilateral legs	440.32	I70.503
Unspecified atherosclerosis of nonautologous biological bypass graft(s) of the extremities, bilateral legs		I70.503
Unsp athscl nonaut bio bypass of the extrm, oth extremity	440.32	I70.508
Atherosclerosis of nonautologous biological bypass graft of other extremity	440.32	I70.508
Unspecified atherosclerosis of nonautologous biological bypass graft(s) of the extremities, other extremity	440.32	I70.508
Atherosclerosis of nonautologous biological bypass graft of other extremity, with unspecified presence of clinical manifestation	440.32	I70.508

© 2021 Eberly LA et al. *JAMA Network Open*.

Unspecified atherosclerosis of nonautologous biological bypass graft(s) of the extremities, other extremity		I70.508
Atherosclerosis of nonautologous biological bypass graft of extremities	440.32	I70.509
Extremity atherosclerosis nonautologous biologic bypass graft	440.32	I70.509
Atherosclerosis of nonautologous biological bypass graft of extremity	440.32	I70.509
Athrscl nonauto gft extr	440.32	I70.509
Atherosclerosis of nonautologous biological bypass graft of lower extremity	440.32	I70.509
Unspecified atherosclerosis of nonautologous biological bypass graft(s) of the extremities, unspecified extremity	440.32	I70.509
Atherosclerosis of nonautologous biological bypass graft of lower extremity, unspecified laterality, with unspecified presence of clinical manifestation	440.32	I70.509
Atherosclerosis of nonautologous biological bypass graft of extremity, unspecified extremity, with unspecified presence of clinical manifestation	440.32	I70.509
Unsp athscl nonaut bio bypass of the extrm, unsp extremity	440.32	I70.509
Unspecified atherosclerosis of nonautologous biological bypass graft(s) of the extremities, unspecified extremity		I70.509
Atherosclerosis of nonautologous biological bypass graft(s) of the extremities intermittent claudication		I70.51
Atherosclerosis of nonautologous biological bypass graft of right lower extremity with intermittent claudication	440.32	I70.511

© 2021 Eberly LA et al. *JAMA Network Open*.

Atherosclerosis of nonautologous biological bypass graft(s) of the extremities with intermittent claudication, right leg	440.32	I70.511
Athscl nonaut bio bypass of extrm w intrmt claud, right leg	440.32	I70.511
Atherosclerosis of nonautologous biological bypass graft(s) of the extremities with intermittent claudication, right leg		I70.511
Atherosclerosis of nonautologous biological bypass graft of left lower extremity with intermittent claudication	440.32	I70.512
Atherosclerosis of nonautologous biological bypass graft(s) of the extremities with intermittent claudication, left leg	440.32	I70.512
Athscl nonaut bio bypass of extrm w intrmt claud, left leg	440.32	I70.512
Atherosclerosis of nonautologous biological bypass graft(s) of the extremities with intermittent claudication, left leg		I70.512
Atherosclerosis of nonautologous biological bypass graft of both lower extremities with intermittent claudication	440.32	I70.513
Atherosclerosis of nonautologous biological bypass graft(s) of the extremities with intermittent claudication, bilateral legs	440.32	I70.513
Athscl nonaut bio bypass of extrm w intrmt claud, bi legs	440.32	I70.513
Atherosclerosis of nonautologous biological bypass graft(s) of the extremities with intermittent claudication, bilateral legs		I70.513

© 2021 Eberly LA et al. *JAMA Network Open*.

Athscl nonaut bio bypass of extrm w intrmt claud, oth extrm	440.32	I70.518
Atherosclerosis of nonautologous biological bypass graft of other extremity with intermittent claudication	440.32	I70.518
Atherosclerosis of nonautologous biological bypass graft(s) of the extremities with intermittent claudication, other extremity	440.32	I70.518
Atherosclerosis of nonautologous biological bypass graft(s) of the extremities with intermittent claudication, other extremity		I70.518
Atherosclerosis of nonautologous biological bypass graft of extremity with intermittent claudication	440.32	I70.519
Atherosclerosis of nonautologous biological bypass graft of lower extremity with intermittent claudication	440.32, 443.9	I70.519
Atherosclerosis of nonautologous biological bypass graft(s) of the extremities with intermittent claudication, unspecified extremity	440.32	I70.519
Atherosclerosis of nonautologous biological bypass graft of lower extremity with intermittent claudication, unspecified laterality	440.32, 443.9	I70.519
Atherosclerosis of nonautologous biological bypass graft of extremity with intermittent claudication, unspecified extremity	440.32	I70.519
Athscl nonaut bio bypass of extrm w intrmt claud, unsp extrm	440.32	I70.519
Atherosclerosis of nonautologous biological bypass graft(s) of the extremities with intermittent claudication, unspecified extremity		I70.519

© 2021 Eberly LA et al. *JAMA Network Open*.

Atherosclerosis of nonautologous biological bypass graft(s) of the extremities with rest pain		I70.52
Atheroscler nonautolg biological bypass graft right leg w/rest pain	440.32	I70.521
Atherosclerosis of nonautologous biological bypass graft of right lower extremity with rest pain	440.32	I70.521
Atherosclerosis of nonautologous biological bypass graft(s) of the extremities with rest pain, right leg	440.32	I70.521
Athscl nonaut bio bypass of the extrm w rest pain, right leg	440.32	I70.521
Atherosclerosis of nonautologous biological bypass graft(s) of the extremities with rest pain, right leg		I70.521
Atheroscler nonautolg biological bypass graft left leg w/rest pain	440.32	I70.522
Atherosclerosis of nonautologous biological bypass graft of left lower extremity with rest pain	440.32	I70.522
Atherosclerosis of nonautologous biological bypass graft(s) of the extremities with rest pain, left leg	440.32	I70.522
Athscl nonaut bio bypass of the extrm w rest pain, left leg	440.32	I70.522
Atherosclerosis of nonautologous biological bypass graft(s) of the extremities with rest pain, left leg		I70.522
Atheroscler nonautolg biological bypass graft both legs w/rest pain	440.32	I70.523

© 2021 Eberly LA et al. *JAMA Network Open*.

Atherosclerosis of nonautologous biological bypass graft of both lower extremities with rest pain	440.32	I70.523
Atherosclerosis of nonautologous biological bypass graft(s) of the extremities with rest pain, bilateral legs	440.32	I70.523
Athscl nonaut bio bypass of the extrm w rest pain, bi legs	440.32	I70.523
Atherosclerosis of nonautologous biological bypass graft(s) of the extremities with rest pain, bilateral legs		I70.523
Athscl nonaut bio bypass of the extrm w rest pain, oth extrm	440.32	I70.528
Atherosclerosis of nonautologous biological bypass graft of other extremity with rest pain	440.32	I70.528
Atherosclerosis of nonautologous biological bypass graft(s) of the extremities with rest pain, other extremity	440.32	I70.528
Atherosclerosis of nonautologous biological bypass graft(s) of the extremities with rest pain, other extremity		I70.528
Atheroscler nonautolg biological bypass graft extremity w/rest pain	440.32	I70.529
Atherosclerosis of nonautologous biological bypass graft of extremity with rest pain	440.32	I70.529
Atherosclerosis of nonautologous biological bypass graft of lower extremity with rest pain	440.32	I70.529
Atherosclerosis of nonautologous biological bypass graft(s) of the extremities with rest pain, unspecified extremity	440.32	I70.529

© 2021 Eberly LA et al. *JAMA Network Open*.

Atherosclerosis of nonautologous biological bypass graft of lower extremity with rest pain, unspecified laterality	440.32	I70.529
Atherosclerosis of nonautologous biological bypass graft of extremity with rest pain, unspecified extremity	440.32	I70.529
Athscl nonaut bio bypass of extrm w rest pain, unsp extrm	440.32	I70.529
Atherosclerosis of nonautologous biological bypass graft(s) of the extremities with rest pain, unspecified extremity		I70.529
Atherosclerosis of nonautologous biological bypass graft(s) of the right leg with ulceration		I70.53
Atherosclerosis of nonautologous biological bypass graft of right lower extremity with ulceration of thigh	440.32	I70.531
Atherosclerosis of nonautologous biological bypass graft(s) of the right leg with ulceration of thigh	440.32	I70.531
Athscl nonaut bio bypass of the right leg w ulcer of thigh	440.32	I70.531
Atherosclerosis of nonautologous biological bypass graft(s) of the right leg with ulceration of thigh		I70.531
Atherosclerosis of nonautologous biological bypass graft of both lower extremities with bilateral ulceration of thighs	440.32, 707.11	I70.531, I70.541
Atherosclerosis of nonautologous biological bypass graft of right lower extremity with ulceration of calf	440.32	I70.532
Atherosclerosis of nonautologous biological bypass graft(s) of the right leg with ulceration of calf	440.32	I70.532

© 2021 Eberly LA et al. *JAMA Network Open*.

Athscl nonaut bio bypass of the right leg w ulcer of calf	440.32	I70.532
Atherosclerosis of nonautologous biological bypass graft(s) of the right leg with ulceration of calf		I70.532
Atherosclerosis of nonautologous biological bypass graft of both lower extremities with bilateral ulceration of calves	440.32, 707.12	I70.532, I70.542
Atherosclerosis of nonautologous biological bypass graft of right lower extremity with ulceration of ankle	440.32	I70.533
Atherosclerosis of nonautologous biological bypass graft(s) of the right leg with ulceration of ankle	440.32	I70.533
Athscl nonaut bio bypass of the right leg w ulcer of ankle	440.32	I70.533
Atherosclerosis of nonautologous biological bypass graft(s) of the right leg with ulceration of ankle		I70.533
Atherosclerosis of nonautologous biological bypass graft of both lower extremities with bilateral ulceration of ankles	440.32, 707.13	I70.533, I70.543
Athscl nonaut bio bypass of r leg w ulcer of heel and midft	440.32	I70.534
Atherosclerosis of nonautologous biological bypass graft of right lower extremity with ulceration of heel	440.32, 707.14	I70.534
Atherosclerosis of nonautologous biological bypass graft of right lower extremity with ulceration of midfoot	440.32, 707.14	I70.534
Atherosclerosis of nonautologous biological bypass graft(s) of the right leg with ulceration of heel and midfoot	440.32	I70.534

© 2021 Eberly LA et al. *JAMA Network Open*.

Atherosclerosis of nonautologous biological bypass graft(s) of the right leg with ulceration of heel and midfoot		I70.534
Atherosclerosis of nonautologous biological bypass graft of both lower extremities with bilateral ulceration of midfeet	440.32, 707.14	I70.534, I70.544
Atherosclerosis of nonautologous biological bypass graft of both lower extremities with bilateral ulceration of heels	440.32, 707.14	I70.534, I70.544
Athscl nonaut bio bypass of right leg w ulcer oth prt foot	440.32	I70.535
Atherosclerosis of nonautologous biological bypass graft of right lower extremity with ulceration of other part of foot	440.32	I70.535
Atherosclerosis of nonautologous biological bypass graft(s) of the right leg with ulceration of other part of foot	440.32	I70.535
Atherosclerosis of nonautologous biological bypass graft(s) of the right leg with ulceration of other part of foot		I70.535
Atherosclerosis of nonautologous biological bypass graft of both lower extremities with bilateral ulceration of other part of feet	440.32, 707.15	I70.535, I70.545
Athscl nonaut bio bypass of r leg w ulcer oth prt low leg	440.32	I70.538
Atherosclerosis of nonautologous biological bypass graft of right lower extremity with ulceration of other part of lower leg	440.32	I70.538
Atherosclerosis of nonautologous biological bypass graft(s) of the right leg with ulceration of other part of lower leg	440.32	I70.538

© 2021 Eberly LA et al. *JAMA Network Open*.

Atherosclerosis of nonautologous biological bypass graft(s) of the right leg with ulceration of other part of lower leg		I70.538
Atherosclerosis of nonautologous biological bypass graft of both lower extremities with bilateral ulceration of other part of lower leg	440.32, 707.19	I70.538, I70.548
Atheroscler nonautolg biological bypass graft right leg w/ulceration	440.32	I70.539
Atherosclerosis of nonautologous biological bypass graft of right lower extremity with ulceration	440.32	I70.539
Atherosclerosis of nonautologous biological bypass graft(s) of the right leg with ulceration of unspecified site	440.32	I70.539
Atherosclerosis of nonautologous biological bypass graft of right lower extremity with ulceration, unspecified ulceration site	440.32	I70.539
Athscl nonaut bio bypass of right leg w ulcer of unsp site	440.32	I70.539
Atherosclerosis of nonautologous biological bypass graft(s) of the right leg with ulceration of unspecified site		I70.539
Atherosclerosis of nonautologous biological bypass graft of both lower extremities with bilateral ulceration	440.32, 707.10	I70.539, I70.549
Atherosclerosis of nonautologous biological bypass graft of both lower extremities with bilateral ulceration, unspecified ulceration site	440.32, 707.10	I70.539, I70.549
Atherosclerosis of nonautologous biological bypass graft(s) of the left leg with ulceration		I70.54

© 2021 Eberly LA et al. *JAMA Network Open*.

Atherosclerosis of nonautologous biological bypass graft of left lower extremity with ulceration of thigh	440.32	I70.541
Atherosclerosis of nonautologous biological bypass graft(s) of the left leg with ulceration of thigh	440.32	I70.541
Athscl nonaut bio bypass of the left leg w ulcer of thigh	440.32	I70.541
Atherosclerosis of nonautologous biological bypass graft(s) of the left leg with ulceration of thigh		I70.541
Atherosclerosis of nonautologous biological bypass graft of left lower extremity with ulceration of calf	440.32	I70.542
Atherosclerosis of nonautologous biological bypass graft(s) of the left leg with ulceration of calf	440.32	I70.542
Athscl nonaut bio bypass of the left leg w ulcer of calf	440.32	I70.542
Atherosclerosis of nonautologous biological bypass graft(s) of the left leg with ulceration of calf		I70.542
Atherosclerosis of nonautologous biological bypass graft of left lower extremity with ulceration of ankle	440.32	I70.543
Atherosclerosis of nonautologous biological bypass graft(s) of the left leg with ulceration of ankle	440.32	I70.543
Athscl nonaut bio bypass of the left leg w ulcer of ankle	440.32	I70.543
Atherosclerosis of nonautologous biological bypass graft(s) of the left leg with ulceration of ankle		I70.543

© 2021 Eberly LA et al. *JAMA Network Open*.

Athscl nonaut bio bypass of left leg w ulc of heel and midft	440.32	I70.544
Atherosclerosis of nonautologous biological bypass graft of left lower extremity with ulceration of midfoot	440.32, 707.14	I70.544
Atherosclerosis of nonautologous biological bypass graft of left lower extremity with ulceration of heel	440.32, 707.14	I70.544
Atherosclerosis of nonautologous biological bypass graft(s) of the left leg with ulceration of heel and midfoot	440.32	I70.544
Atherosclerosis of nonautologous biological bypass graft(s) of the left leg with ulceration of heel and midfoot		I70.544
Athscl nonaut bio bypass of left leg w ulcer oth prt foot	440.32	I70.545
Atherosclerosis of nonautologous biological bypass graft of left lower extremity with ulceration of other part of foot	440.32	I70.545
Atherosclerosis of nonautologous biological bypass graft(s) of the left leg with ulceration of other part of foot	440.32	I70.545
Atherosclerosis of nonautologous biological bypass graft(s) of the left leg with ulceration of other part of foot		I70.545
Athscl nonaut bio bypass of left leg w ulcer oth prt low leg	440.32	I70.548
Atherosclerosis of nonautologous biological bypass graft of left lower extremity with ulceration of other part of lower leg	440.32	I70.548
Atherosclerosis of nonautologous biological bypass graft(s) of the left leg with ulceration of other part of lower leg	440.32	I70.548

© 2021 Eberly LA et al. *JAMA Network Open*.

Atherosclerosis of nonautologous biological bypass graft(s) of the left leg with ulceration of other part of lower leg		I70.548
Atheroscler nonautolg biological bypass graft left leg w/ulceration	440.32, 707.9	I70.549
Atherosclerosis of nonautologous biological bypass graft of left lower extremity with ulceration	440.32, 707.9	I70.549
Atherosclerosis of nonautologous biological bypass graft(s) of the left leg with ulceration of unspecified site	440.32, 707.9	I70.549
Atherosclerosis of nonautologous biological bypass graft of left lower extremity with ulceration, unspecified ulceration site	440.32, 707.9	I70.549
Athscl nonaut bio bypass of left leg w ulcer of unsp site	440.32, 707.9	I70.549
Atherosclerosis of nonautologous biological bypass graft(s) of the left leg with ulceration of unspecified site		I70.549
Athscl nonautologous bio bypass of extremity w ulceration	440.32, 707.9	I70.55
Atherosclerosis of nonautologous biological bypass graft of lower extremity with ulceration of calf	440.32, 707.12	I70.55
Atherosclerosis of nonautologous biological bypass graft of lower extremity with ulceration of ankle	440.32, 707.13	I70.55
Atherosclerosis of nonautologous biological bypass graft of lower extremity with ulceration	440.32, 707.10	I70.55
Atherosclerosis of nonautologous biological bypass graft of lower extremity with ulceration of heel	440.32, 707.14	I70.55

© 2021 Eberly LA et al. *JAMA Network Open*.

Atherosclerosis of nonautologous biological bypass graft of lower extremity with ulceration of midfoot	440.32, 707.14	I70.55
Atherosclerosis of nonautologous biological bypass graft of lower extremity with ulceration of thigh	440.32, 707.11	I70.55
Atherosclerosis of nonautologous biological bypass graft of extremity with ulceration	440.32, 707.9	I70.55
Atherosclerosis of nonautologous biological bypass graft of other extremity with ulceration	440.32	I70.55
Atherosclerosis of nonautologous biological bypass graft(s) of other extremity with ulceration	440.32	I70.55
Atherosclerosis of nonautologous biological bypass graft of lower extremity with ulceration of other part of foot	440.32, 707.15	I70.55
Atherosclerosis of nonautologous biological bypass graft of lower extremity with ulceration of other part of lower leg	440.32, 707.19	I70.55
Atherosclerosis of nonautologous biological bypass graft of lower extremity with ulceration, unspecified laterality, unspecified ulceration site	440.32, 707.10	I70.55
Atherosclerosis of nonautologous biological bypass graft of lower extremity with ulceration of other part of lower leg, unspecified laterality	440.32, 707.19	I70.55
Atherosclerosis of nonautologous biological bypass graft of lower extremity with ulceration of midfoot, unspecified laterality	440.32, 707.14	I70.55
Atherosclerosis of nonautologous biological bypass graft of lower extremity with ulceration of calf, unspecified laterality	440.32, 707.12	I70.55

© 2021 Eberly LA et al. *JAMA Network Open*.

Atherosclerosis of nonautologous biological bypass graft of lower extremity with ulceration of heel, unspecified laterality	440.32, 707.14	I70.55
Atherosclerosis of nonautologous biological bypass graft of lower extremity with ulceration of ankle, unspecified laterality	440.32, 707.13	I70.55
Atherosclerosis of nonautologous biological bypass graft of lower extremity with ulceration of other part of foot, unspecified laterality	440.32, 707.15	I70.55
Atherosclerosis of nonautologous biological bypass graft of lower extremity with ulceration of thigh, unspecified laterality	440.32, 707.11	I70.55
Atherosclerosis of nonautologous biological bypass graft of extremity with ulceration, unspecified extremity	440.32, 707.9	I70.55
Atherosclerosis of nonautologous biological bypass graft(s) of other extremity with ulceration		I70.55
Atherosclerosis of nonautologous biological bypass graft(s) of the extremities with gangrene		I70.56
Atheroscler nonautolg biological bypass graft right leg w/gangrene	440.32	I70.561
Atherosclerosis of nonautologous biological bypass graft of right lower extremity with gangrene	440.32	I70.561
Atherosclerosis of nonautologous biological bypass graft(s) of the extremities with gangrene, right leg	440.32	I70.561
Athscl nonaut bio bypass of the extrm w gangrene, right leg	440.32	I70.561
Atherosclerosis of nonautologous biological bypass graft(s) of the extremities with gangrene, right leg		I70.561

© 2021 Eberly LA et al. *JAMA Network Open*.

Atheroscler nonautolg biological bypass graft left leg w/gangrene	440.32, 785.4	I70.562
Atherosclerosis of nonautologous biological bypass graft of left lower extremity with gangrene	440.32, 785.4	I70.562
Atherosclerosis of nonautologous biological bypass graft(s) of the extremities with gangrene, left leg	440.32, 785.4	I70.562
Athscl nonaut bio bypass of the extrm w gangrene, left leg	440.32, 785.4	I70.562
Atherosclerosis of nonautologous biological bypass graft(s) of the extremities with gangrene, left leg		I70.562
Atheroscler nonautolg biological bypass graft both legs w/gangrene	440.32, 785.4	I70.563
Atherosclerosis of nonautologous biological bypass graft of both lower extremities with gangrene	440.32, 785.4	I70.563
Atherosclerosis of nonautologous biological bypass graft(s) of the extremities with gangrene, bilateral legs	440.32, 785.4	I70.563
Athscl nonaut bio bypass of the extrm w gangrene, bi legs	440.32, 785.4	I70.563
Atherosclerosis of nonautologous biological bypass graft(s) of the extremities with gangrene, bilateral legs		I70.563
Athscl nonaut bio bypass of the extrm w gangrene, oth extrm	440.32	I70.568
Atherosclerosis of nonautologous biological bypass graft of other extremity with gangrene	440.32	I70.568
Atherosclerosis of nonautologous biological bypass graft(s) of the extremities with gangrene, other extremity	440.32	I70.568

© 2021 Eberly LA et al. *JAMA Network Open*.

Atherosclerosis of nonautologous biological bypass graft(s) of the extremities with gangrene, other extremity		I70.568
Atheroscler nonautolg biological bypass graft extremity w/gangrene	440.32	I70.569
Atherosclerosis of nonautologous biological bypass graft of extremity with gangrene	440.32	I70.569
Atherosclerosis of nonautologous biological bypass graft of lower extremity with gangrene	440.32, 785.4	I70.569
Atherosclerosis of nonautologous biological bypass graft(s) of the extremities with gangrene, unspecified extremity	440.32	I70.569
Atherosclerosis of nonautologous biological bypass graft of lower extremity with gangrene, unspecified laterality	440.32, 785.4	I70.569
Atherosclerosis of nonautologous biological bypass graft of extremity with gangrene, unspecified extremity	440.32	I70.569
Athscl nonaut bio bypass of the extrm w gangrene, unsp extrm	440.32	I70.569
Atherosclerosis of nonautologous biological bypass graft(s) of the extremities with gangrene, unspecified extremity		I70.569
Other atherosclerosis of nonautologous biological bypass graft(s) of the extremities		I70.59
Oth athscl nonaut bio bypass of the extremities, right leg	440.32	I70.591
Other atherosclerosis of nonautologous biological bypass graft(s) of the extremities, right leg	440.32	I70.591

© 2021 Eberly LA et al. *JAMA Network Open*.

Atherosclerosis of nonautologous biological bypass graft of right lower extremity with other clinical manifestation	440.32	I70.591
Other atherosclerosis of nonautologous biological bypass graft(s) of the extremities, right leg		I70.591
Oth athscl nonaut bio bypass of the extremities, left leg	440.32	I70.592
Other atherosclerosis of nonautologous biological bypass graft(s) of the extremities, left leg	440.32	I70.592
Atherosclerosis of nonautologous biological bypass graft of left lower extremity with other clinical manifestation	440.32	I70.592
Other atherosclerosis of nonautologous biological bypass graft(s) of the extremities, left leg		I70.592
Oth athscl nonaut bio bypass of the extrm, bilateral legs	440.32	I70.593
Other atherosclerosis of nonautologous biological bypass graft(s) of the extremities, bilateral legs	440.32	I70.593
Atherosclerosis of nonautologous biological bypass graft of both lower extremities with other clinical manifestation	440.32	I70.593
Other atherosclerosis of nonautologous biological bypass graft(s) of the extremities, bilateral legs		I70.593
Oth athscl nonaut bio bypass of the extrm, oth extremity	440.32	I70.598
Other atherosclerosis of nonautologous biological bypass graft(s) of the extremities, other extremity	440.32	I70.598

© 2021 Eberly LA et al. *JAMA Network Open*.

Atherosclerosis of nonautologous biological bypass graft of other extremity with other clinical manifestation	440.32	I70.598
Other atherosclerosis of nonautologous biological bypass graft(s) of the extremities, other extremity		I70.598
Atherosclerotic disease of nonautologous bypass graft of extremity	440.32	I70.599
Oth athscl nonaut bio bypass of the extrm, unsp extremity	440.32	I70.599
Atherosclerosis of nonautologous bypass graft of extremity	440.32	I70.599
Other atherosclerosis of nonautologous biological bypass graft(s) of the extremities, unspecified extremity	440.32	I70.599
Atherosclerosis of nonautologous biological bypass graft of extremity with other clinical manifestation	440.32	I70.599
Atherosclerosis of nonautologous biological bypass graft of lower extremity with other clinical manifestation	440.32	I70.599
Atherosclerosis of nonautologous biological bypass graft of lower extremity with other clinical manifestation, unspecified laterality	440.32	I70.599
Atherosclerosis of nonautologous biological bypass graft of extremity with other clinical manifestation, unspecified extremity	440.32	I70.599
Other atherosclerosis of nonautologous biological bypass graft(s) of the extremities, unspecified extremity		I70.599
Atherosclerosis of nonbiological bypass graft(s) of the extremities		I70.6
Unspecified atherosclerosis of nonbiological bypass graft(s) of the extremities		I70.60

© 2021 Eberly LA et al. *JAMA Network Open*.

Atherosclerosis of nonbiological bypass graft of right lower extremity	440.3	I70.601
Unspecified atherosclerosis of nonbiological bypass graft(s) of the extremities, right leg	440.3	I70.601
Atherosclerosis of nonbiological bypass graft of right lower extremity, with unspecified presence of clinical manifestation	440.3	I70.601
Unsp athscl nonbiol bypass of the extremities, right leg	440.3	I70.601
Unspecified atherosclerosis of nonbiological bypass graft(s) of the extremities, right leg		I70.601
Atherosclerosis of nonbiological bypass graft of left lower extremity	440.3	I70.602
Unspecified atherosclerosis of nonbiological bypass graft(s) of the extremities, left leg	440.3	I70.602
Atherosclerosis of nonbiological bypass graft of left lower extremity, with unspecified presence of clinical manifestation	440.3	I70.602
Unsp athscl nonbiol bypass of the extremities, left leg	440.3	I70.602
Unspecified atherosclerosis of nonbiological bypass graft(s) of the extremities, left leg		I70.602
Atherosclerosis of nonbiological bypass graft of both legs	440.3	I70.603
Atherosclerosis of nonbiological bypass graft of both lower extremities	440.3	I70.603

© 2021 Eberly LA et al. *JAMA Network Open*.

Unspecified atherosclerosis of nonbiological bypass graft(s) of the extremities, bilateral legs	440.3	I70.603
Atherosclerosis of nonbiological bypass graft of both lower extremities, with unspecified presence of clinical manifestation	440.3	I70.603
Unsp athscl nonbiol bypass of the extrm, bilateral legs	440.3	I70.603
Unspecified atherosclerosis of nonbiological bypass graft(s) of the extremities, bilateral legs		I70.603
Unsp athscl nonbiol bypass of the extremities, oth extremity	440.3	I70.608
Atherosclerosis of nonbiological bypass graft of other extremity	440.3	I70.608
Unspecified atherosclerosis of nonbiological bypass graft(s) of the extremities, other extremity	440.3	I70.608
Atherosclerosis of nonbiological bypass graft of other extremity, with unspecified presence of clinical manifestation	440.3	I70.608
Unspecified atherosclerosis of nonbiological bypass graft(s) of the extremities, other extremity		I70.608
Atherosclerosis of nonbiological bypass graft of extremity	440.3	I70.609
Atherosclerosis of nonbiological bypass graft of lower extremity	440.3	I70.609
Unspecified atherosclerosis of nonbiological bypass graft(s) of the extremities, unspecified extremity	440.3	I70.609
Atherosclerosis of nonbiological bypass graft of lower extremity, unspecified laterality, with unspecified presence of clinical manifestation	440.3	I70.609

© 2021 Eberly LA et al. *JAMA Network Open*.

Atherosclerosis of nonbiological bypass graft of extremity, unspecified extremity, with unspecified presence of clinical manifestation	440.3	I70.609
Unsp athscl nonbiol bypass of the extrm, unsp extremity	440.3	I70.609
Unspecified atherosclerosis of nonbiological bypass graft(s) of the extremities, unspecified extremity		I70.609
Atherosclerosis of nonbiological bypass graft(s) of the extremities with intermittent claudication		I70.61
Atheroscler nonbiologic bypass graft right leg w/intermit claudication	440.3	I70.611
Atherosclerosis of nonbiological bypass graft of right lower extremity with intermittent claudication	440.3	I70.611
Atherosclerosis of nonbiological bypass graft(s) of the extremities with intermittent claudication, right leg	440.3	I70.611
Athscl nonbiol bypass of the extrm w intrmt claud, right leg	440.3	I70.611
Atherosclerosis of nonbiological bypass graft(s) of the extremities with intermittent claudication, right leg		I70.611
Atheroscler nonbiologic bypass graft left leg w/intermit claudication	440.3	I70.612
Atherosclerosis of nonbiological bypass graft of left lower extremity with intermittent claudication	440.3	I70.612
Atherosclerosis of nonbiological bypass graft(s) of the extremities with intermittent claudication, left leg	440.3	I70.612

© 2021 Eberly LA et al. *JAMA Network Open*.

Athscl nonbiol bypass of the extrm w intrmt claud, left leg	440.3	I70.612
Atherosclerosis of nonbiological bypass graft(s) of the extremities with intermittent claudication, left leg		I70.612
Atheroscler nonbiologic bypass graft both legs w/intermit claudication	440.3	I70.613
Atherosclerosis of nonbiological bypass graft of both lower extremities with intermittent claudication	440.3	I70.613
Atherosclerosis of nonbiological bypass graft(s) of the extremities with intermittent claudication, bilateral legs	440.3	I70.613
Athscl nonbiol bypass of the extrm w intrmt claud, bi legs	440.3	I70.613
Atherosclerosis of nonbiological bypass graft(s) of the extremities with intermittent claudication, bilateral legs		I70.613
Athscl nonbiol bypass of the extrm w intrmt claud, oth extrm	440.3	I70.618
Atherosclerosis of nonbiological bypass graft of other extremity with intermittent claudication	440.3	I70.618
Atherosclerosis of nonbiological bypass graft(s) of the extremities with intermittent claudication, other extremity	440.3	I70.618
Atherosclerosis of nonbiological bypass graft(s) of the extremities with intermittent claudication, other extremity		I70.618
Atheroscler nonbiologic bypass graft extremity w/intermit claudication	440.3	I70.619
Atherosclerosis of nonbiological bypass graft of extremity with intermittent claudication	440.3	I70.619

© 2021 Eberly LA et al. *JAMA Network Open*.

Atherosclerosis of nonbiological bypass graft of lower extremity with intermittent claudication	440.30, 443.9	I70.619
Atherosclerosis of nonbiological bypass graft(s) of the extremities with intermittent claudication, unspecified extremity	440.3	I70.619
Atherosclerosis of nonbiological bypass graft of lower extremity with intermittent claudication, unspecified laterality	440.30, 443.9	I70.619
Atherosclerosis of nonbiological bypass graft of extremity with intermittent claudication, unspecified extremity	440.3	I70.619
Athscl nonbiol bypass of extrm w intrmt claud, unsp extrm	440.3	I70.619
Atherosclerosis of nonbiological bypass graft(s) of the extremities with intermittent claudication, unspecified extremity		I70.619
Atherosclerosis of nonbiological bypass graft(s) of the extremities with rest pain		I70.62
Atheroscler of nonbiologic bypass graft of right leg with rest pain	440.3	I70.621
Atherosclerosis of nonbiological bypass graft of right lower extremity with rest pain	440.3	I70.621
Atherosclerosis of nonbiological bypass graft(s) of the extremities with rest pain, right leg	440.3	I70.621
Athscl nonbiol bypass of the extrm w rest pain, right leg	440.3	I70.621
Atherosclerosis of nonbiological bypass graft(s) of the extremities with rest pain, right leg		I70.621

© 2021 Eberly LA et al. *JAMA Network Open*.

Atherosclerosis of nonbiologic bypass graft of left leg with rest pain	440.3	I70.622
Atherosclerosis of nonbiological bypass graft of left lower extremity with rest pain	440.3	I70.622
Atherosclerosis of nonbiological bypass graft(s) of the extremities with rest pain, left leg	440.3	I70.622
Athscl nonbiol bypass of the extrm w rest pain, left leg	440.3	I70.622
Atherosclerosis of nonbiological bypass graft(s) of the extremities with rest pain, left leg		I70.622
Atheroscler of nonbiologic bypass graft of both legs with rest pain	440.3	I70.623
Atherosclerosis of nonbiological bypass graft of both lower extremities with rest pain	440.3	I70.623
Atherosclerosis of nonbiological bypass graft(s) of the extremities with rest pain, bilateral legs	440.3	I70.623
Athscl nonbiol bypass of the extrm w rest pain, bi legs	440.3	I70.623
Atherosclerosis of nonbiological bypass graft(s) of the extremities with rest pain, bilateral legs		I70.623
Athscl nonbiol bypass of the extrm w rest pain, oth extrm	440.3	I70.628
Atherosclerosis of nonbiological bypass graft of other extremity with rest pain	440.3	I70.628
Atherosclerosis of nonbiological bypass graft(s) of the extremities with rest pain, other extremity	440.3	I70.628

© 2021 Eberly LA et al. *JAMA Network Open*.

Atherosclerosis of nonbiological bypass graft(s) of the extremities with rest pain, other extremity		I70.628
Atheroscler of nonbiologic bypass graft of extremity with rest pain	440.3	I70.629
Atherosclerosis of nonbiological bypass graft of extremity with rest pain	440.3	I70.629
Atherosclerosis of nonbiological bypass graft of lower extremity with rest pain	440.3	I70.629
Atherosclerosis of nonbiological bypass graft(s) of the extremities with rest pain, unspecified extremity	440.3	I70.629
Atherosclerosis of nonbiological bypass graft of lower extremity with rest pain, unspecified laterality	440.3	I70.629
Atherosclerosis of nonbiological bypass graft of extremity with rest pain, unspecified extremity	440.3	I70.629
Athscl nonbiol bypass of the extrm w rest pain, unsp extrm	440.3	I70.629
Atherosclerosis of nonbiological bypass graft(s) of the extremities with rest pain, unspecified extremity		I70.629
Atherosclerosis of nonbiological bypass graft(s) of the right leg with ulceration		I70.63
Atheroscler nonbiologic bypass graft right leg w/ulceration thigh	440.30, 707.11	I70.631
Atherosclerosis of nonbiological bypass graft of right lower extremity with ulceration of thigh	440.30, 707.11	I70.631
Atherosclerosis of nonbiological bypass graft(s) of the right leg with ulceration of thigh	440.30, 707.11	I70.631

© 2021 Eberly LA et al. *JAMA Network Open*.

Athscl nonbiol bypass of the right leg w ulceration of thigh	440.30, 707.11	I70.631
Atherosclerosis of nonbiological bypass graft(s) of the right leg with ulceration of thigh		I70.631
Atherosclerosis of nonbiological bypass graft of both lower extremities with bilateral ulceration of thighs	440.30, 707.11	I70.631, I70.641
Atheroscler nonbiologic bypass graft right leg w/ulceration calf	440.30, 707.12	I70.632
Atherosclerosis of nonbiological bypass graft of right lower extremity with ulceration of calf	440.30, 707.12	I70.632
Atherosclerosis of nonbiological bypass graft(s) of the right leg with ulceration of calf	440.30, 707.12	I70.632
Athscl nonbiol bypass of the right leg w ulceration of calf	440.30, 707.12	I70.632
Atherosclerosis of nonbiological bypass graft(s) of the right leg with ulceration of calf		I70.632
Atherosclerosis of nonbiological bypass graft of both lower extremities with bilateral ulceration of calves	440.30, 707.12	I70.632, I70.642
Atheroscler nonbiologic bypass graft right leg w/ulceration ankle	440.30, 707.13	I70.633
Atherosclerosis of nonbiological bypass graft of right lower extremity with ulceration of ankle	440.30, 707.13	I70.633
Atherosclerosis of nonbiological bypass graft(s) of the right leg with ulceration of ankle	440.30, 707.13	I70.633
Athscl nonbiol bypass of the right leg w ulceration of ankle	440.30, 707.13	I70.633

© 2021 Eberly LA et al. *JAMA Network Open*.

Atherosclerosis of nonbiological bypass graft(s) of the right leg with ulceration of ankle		I70.633
Atherosclerosis of nonbiological bypass graft of both lower extremities with bilateral ulceration of ankles	440.30, 707.13	I70.633, I70.643
Athscl nonbiol bypass of right leg w ulcer of heel and midft	440.3	I70.634
Atherosclerosis of nonbiological bypass graft of right lower extremity with ulceration of heel	440.30, 707.14	I70.634
Atherosclerosis of nonbiological bypass graft of right lower extremity with ulceration of midfoot	440.30, 707.14	I70.634
Atherosclerosis of nonbiological bypass graft(s) of the right leg with ulceration of heel and midfoot	440.3	I70.634
Atherosclerosis of nonbiological bypass graft(s) of the right leg with ulceration of heel and midfoot		I70.634
Atherosclerosis of nonbiological bypass graft of both lower extremities with bilateral ulceration of midfeet	440.30, 707.14	I70.634, I70.644
Atherosclerosis of nonbiological bypass graft of both lower extremities with bilateral ulceration of heels	440.30, 707.14	I70.634, I70.644
Athscl nonbiol bypass of the right leg w ulcer oth prt foot	440.3	I70.635
Atherosclerosis of nonbiological bypass graft of right lower extremity with ulceration of other part of foot	440.3	I70.635
Atherosclerosis of nonbiological bypass graft(s) of the right leg with ulceration of other part of foot	440.3	I70.635

© 2021 Eberly LA et al. *JAMA Network Open*.

Atherosclerosis of nonbiological bypass graft(s) of the right leg with ulceration of other part of foot		I70.635
Atherosclerosis of nonbiological bypass graft of both lower extremities with bilateral ulceration of other part of feet	440.30, 707.15	I70.635, I70.645
Athscl nonbiol bypass of right leg w ulcer oth prt low leg	440.3	I70.638
Atherosclerosis of nonbiological bypass graft of right lower extremity with ulceration of other part of lower leg	440.3	I70.638
Atherosclerosis of nonbiological bypass graft(s) of the right leg with ulceration of other part of lower leg	440.3	I70.638
Atherosclerosis of nonbiological bypass graft(s) of the right leg with ulceration of other part of lower leg		I70.638
Atherosclerosis of nonbiological bypass graft of both lower extremities with bilateral ulceration of other part of lower legs	440.30, 707.19	I70.638, I70.648
Atheroscler of nonbiologic bypass graft of right leg with ulceration	440.30, 707.9	I70.639
Atherosclerosis of nonbiological bypass graft of right lower extremity with ulceration	440.30, 707.9	I70.639
Atherosclerosis of nonbiological bypass graft(s) of the right leg with ulceration of unspecified site	440.30, 707.9	I70.639
Atherosclerosis of nonbiological bypass graft of right lower extremity with ulceration, unspecified ulceration site	440.30, 707.9	I70.639
Athscl nonbiol bypass of the right leg w ulcer of unsp site	440.30, 707.9	I70.639

© 2021 Eberly LA et al. *JAMA Network Open*.

Atherosclerosis of nonbiological bypass graft(s) of the right leg with ulceration of unspecified site		I70.639
Atherosclerosis of nonbiological bypass graft of both lower extremities with bilateral ulceration	440.30, 707.10	I70.639, I70.649
Atherosclerosis of nonbiological bypass graft of both lower extremities with bilateral ulceration, unspecified ulceration site	440.30, 707.10	I70.639, I70.649
Atherosclerosis of nonbiological bypass graft(s) of the left leg with ulceration		I70.64
Atheroscler nonbiologic bypass graft left leg w/ulceration thigh	440.30, 707.11	I70.641
Atherosclerosis of nonbiological bypass graft of left lower extremity with ulceration of thigh	440.30, 707.11	I70.641
Atherosclerosis of nonbiological bypass graft(s) of the left leg with ulceration of thigh	440.30, 707.11	I70.641
Athscl nonbiol bypass of the left leg w ulceration of thigh	440.30, 707.11	I70.641
Atherosclerosis of nonbiological bypass graft(s) of the left leg with ulceration of thigh		I70.641
Atheroscler nonbiologic bypass graft left leg w/ulceration calf	440.30, 707.12	I70.642
Atherosclerosis of nonbiological bypass graft of left lower extremity with ulceration of calf	440.30, 707.12	I70.642
Atherosclerosis of nonbiological bypass graft(s) of the left leg with ulceration of calf	440.30, 707.12	I70.642

© 2021 Eberly LA et al. *JAMA Network Open*.

Athscl nonbiol bypass of the left leg w ulceration of calf	440.30, 707.12	I70.642
Atherosclerosis of nonbiological bypass graft(s) of the left leg with ulceration of calf		I70.642
Atheroscler nonbiologic bypass graft left leg w/ulceration ankle	440.3	I70.643
Atherosclerosis of nonbiological bypass graft of left lower extremity with ulceration of ankle	440.3	I70.643
Atherosclerosis of nonbiological bypass graft(s) of the left leg with ulceration of ankle	440.3	I70.643
Athscl nonbiol bypass of the left leg w ulceration of ankle	440.3	I70.643
Atherosclerosis of nonbiological bypass graft(s) of the left leg with ulceration of ankle		I70.643
Athscl nonbiol bypass of left leg w ulcer of heel and midft	440.3	I70.644
Atherosclerosis of nonbiological bypass graft of left lower extremity with ulceration of midfoot	440.30, 707.14	I70.644
Atherosclerosis of nonbiological bypass graft of left lower extremity with ulceration of heel	440.30, 707.14	I70.644
Atherosclerosis of nonbiological bypass graft(s) of the left leg with ulceration of heel and midfoot	440.3	I70.644
Atherosclerosis of nonbiological bypass graft(s) of the left leg with ulceration of heel and midfoot		I70.644
Athscl nonbiol bypass of the left leg w ulcer oth prt foot	440.3	I70.645

© 2021 Eberly LA et al. *JAMA Network Open*.

Atherosclerosis of nonbiological bypass graft of left lower extremity with ulceration of other part of foot	440.3	I70.645
Atherosclerosis of nonbiological bypass graft(s) of the left leg with ulceration of other part of foot	440.3	I70.645
Atherosclerosis of nonbiological bypass graft(s) of the left leg with ulceration of other part of foot		I70.645
Athscl nonbiol bypass of left leg w ulcer oth prt low leg	440.3	I70.648
Atherosclerosis of nonbiological bypass graft of left lower extremity with ulceration of other part of lower leg	440.3	I70.648
Atherosclerosis of nonbiological bypass graft(s) of the left leg with ulceration of other part of lower leg	440.3	I70.648
Atherosclerosis of nonbiological bypass graft(s) of the left leg with ulceration of other part of lower leg		I70.648
Atheroscler of nonbiologic bypass graft of left leg with ulceration	440.3	I70.649
Atherosclerosis of nonbiological bypass graft of left lower extremity with ulceration	440.3	I70.649
Atherosclerosis of nonbiological bypass graft(s) of the left leg with ulceration of unspecified site	440.3	I70.649
Atherosclerosis of nonbiological bypass graft of left lower extremity with ulceration, unspecified ulceration site	440.3	I70.649
Athscl nonbiol bypass of the left leg w ulcer of unsp site	440.3	I70.649
Atherosclerosis of nonbiological bypass graft(s) of the left leg with ulceration of unspecified site		I70.649

© 2021 Eberly LA et al. *JAMA Network Open*.

Athscl nonbiological bypass of extremity w ulceration	440.30, 707.9	I70.65
Atherosclerosis of nonbiological bypass graft of lower extremity with ulceration of midfoot	440.30, 707.14	I70.65
Atherosclerosis of nonbiological bypass graft of lower extremity with ulceration of thigh	440.30, 707.11	I70.65
Atherosclerosis of nonbiological bypass graft of lower extremity with ulceration of ankle	440.30, 707.13	I70.65
Atherosclerosis of nonbiological bypass graft of lower extremity with ulceration of calf	440.30, 707.12	I70.65
Atherosclerosis of nonbiological bypass graft of lower extremity with ulceration of heel	440.30, 707.14	I70.65
Atherosclerosis of nonbiological bypass graft of lower extremity with ulceration	440.30, 707.10	I70.65
Atherosclerosis of nonbiological bypass graft of extremity with ulceration	440.30, 707.9	I70.65
Atherosclerosis of nonbiological bypass graft of other extremity with ulceration	440.3	I70.65
Atherosclerosis of nonbiological bypass graft(s) of other extremity with ulceration	440.3	I70.65
Atherosclerosis of nonbiological bypass graft of lower extremity with ulceration of other part of foot	440.30, 707.15	I70.65
Atherosclerosis of nonbiological bypass graft of lower extremity with ulceration of other part of lower leg	440.30, 707.19	I70.65
Atherosclerosis of nonbiological bypass graft of lower extremity with ulceration, unspecified laterality, unspecified ulceration site	440.30, 707.10	I70.65

© 2021 Eberly LA et al. *JAMA Network Open*.

Atherosclerosis of nonbiological bypass graft of lower extremity with ulceration of midfoot, unspecified laterality	440.30, 707.14	I70.65
Atherosclerosis of nonbiological bypass graft of lower extremity with ulceration of heel, unspecified laterality	440.30, 707.14	I70.65
Atherosclerosis of nonbiological bypass graft of lower extremity with ulceration of ankle, unspecified laterality	440.30, 707.13	I70.65
Atherosclerosis of nonbiological bypass graft of lower extremity with ulceration of thigh, unspecified laterality	440.30, 707.11	I70.65
Atherosclerosis of nonbiological bypass graft of lower extremity with ulceration of other part of lower leg, unspecified laterality	440.30, 707.19	I70.65
Atherosclerosis of nonbiological bypass graft of lower extremity with ulceration of other part of foot, unspecified laterality	440.30, 707.15	I70.65
Atherosclerosis of nonbiological bypass graft of lower extremity with ulceration of calf, unspecified laterality	440.30, 707.12	I70.65
Atherosclerosis of nonbiological bypass graft of extremity with ulceration, unspecified extremity	440.30, 707.9	I70.65
Atherosclerosis of nonbiological bypass graft(s) of other extremity with ulceration		I70.65
Atherosclerosis of nonbiological bypass graft(s) of the extremities with gangrene		I70.66
Atherosclerosis of nonbiologic bypass graft of right leg with gangrene	440.30, 785.4	I70.661
Atherosclerosis of nonbiological bypass graft of right lower extremity with gangrene	440.30, 785.4	I70.661

© 2021 Eberly LA et al. *JAMA Network Open*.

Atherosclerosis of nonbiological bypass graft(s) of the extremities with gangrene, right leg	440.30, 785.4	I70.661
Athscl nonbiol bypass of the extrm w gangrene, right leg	440.30, 785.4	I70.661
Atherosclerosis of nonbiological bypass graft(s) of the extremities with gangrene, right leg		I70.661
Atherosclerosis of nonbiologic bypass graft of left leg with gangrene	440.30, 785.4	I70.662
Atherosclerosis of nonbiological bypass graft of left lower extremity with gangrene	440.30, 785.4	I70.662
Atherosclerosis of nonbiological bypass graft(s) of the extremities with gangrene, left leg	440.30, 785.4	I70.662
Athscl nonbiol bypass of the extrm w gangrene, left leg	440.30, 785.4	I70.662
Atherosclerosis of nonbiological bypass graft(s) of the extremities with gangrene, left leg		I70.662
Atheroscler of nonbiologic bypass graft of bilateral legs w/gangrene	440.30, 785.4	I70.663
Atherosclerosis of nonbiological bypass graft of bilateral lower extremities with gangrene	440.30, 785.4	I70.663
Atherosclerosis of nonbiological bypass graft of both lower extremities with gangrene	440.30, 785.4	I70.663
Atherosclerosis of nonbiological bypass graft(s) of the extremities with gangrene, bilateral legs	440.30, 785.4	I70.663
Athscl nonbiol bypass of the extrm w gangrene, bi legs	440.30, 785.4	I70.663

© 2021 Eberly LA et al. *JAMA Network Open*.

Atherosclerosis of nonbiological bypass graft(s) of the extremities with gangrene, bilateral legs		I70.663
Athscl nonbiol bypass of the extrm w gangrene, oth extremity	440.3	I70.668
Atherosclerosis of nonbiological bypass graft of other extremity with gangrene	440.3	I70.668
Atherosclerosis of nonbiological bypass graft(s) of the extremities with gangrene, other extremity	440.3	I70.668
Atherosclerosis of nonbiological bypass graft(s) of the extremities with gangrene, other extremity		I70.668
Atherosclerosis of nonbiologic bypass graft of extremity with gangrene	440.30, 785.4	I70.669
Atherosclerosis of nonbiological bypass graft of extremity with gangrene	440.30, 785.4	I70.669
Atherosclerosis of nonbiological bypass graft of lower extremity with gangrene	440.30, 785.4	I70.669
Atherosclerosis of nonbiological bypass graft(s) of the extremities with gangrene, unspecified extremity	440.30, 785.4	I70.669
Atherosclerosis of nonbiological bypass graft of lower extremity with gangrene, unspecified laterality	440.30, 785.4	I70.669
Atherosclerosis of nonbiological bypass graft of extremity with gangrene, unspecified extremity	440.30, 785.4	I70.669
Athscl nonbiol bypass of the extrm w gangrene, unsp extrm	440.30, 785.4	I70.669
Atherosclerosis of nonbiological bypass graft(s) of the extremities with gangrene, unspecified extremity		I70.669
Other atherosclerosis of nonbiological bypass graft(s) of the extremities		I70.69

© 2021 Eberly LA et al. *JAMA Network Open*.

Oth athscl nonbiol bypass of the extremities, right leg	440.3	I70.691
Other atherosclerosis of nonbiological bypass graft(s) of the extremities, right leg	440.3	I70.691
Atherosclerosis of nonbiological bypass graft of right lower extremity with other clinical manifestation	440.3	I70.691
Other atherosclerosis of nonbiological bypass graft(s) of the extremities, right leg		I70.691
Oth athscl nonbiological bypass of the extremities, left leg	440.3	I70.692
Other atherosclerosis of nonbiological bypass graft(s) of the extremities, left leg	440.3	I70.692
Atherosclerosis of nonbiological bypass graft of left lower extremity with other clinical manifestation	440.3	I70.692
Other atherosclerosis of nonbiological bypass graft(s) of the extremities, left leg		I70.692
Oth athscl nonbiol bypass of the extremities, bilateral legs	440.3	I70.693
Other atherosclerosis of nonbiological bypass graft(s) of the extremities, bilateral legs	440.3	I70.693
Atherosclerosis of nonbiological bypass graft of both lower extremities with other clinical manifestation	440.3	I70.693
Other atherosclerosis of nonbiological bypass graft(s) of the extremities, bilateral legs		I70.693
Oth athscl nonbiol bypass of the extremities, oth extremity	440.3	I70.698
Other atherosclerosis of nonbiological bypass graft(s) of the extremities, other extremity	440.3	I70.698

© 2021 Eberly LA et al. *JAMA Network Open*.

Atherosclerosis of nonbiological bypass graft of other extremity with other clinical manifestation	440.3	I70.698
Other atherosclerosis of nonbiological bypass graft(s) of the extremities, other extremity		I70.698
Oth athscl nonbiol bypass of the extremities, unsp extremity	440.3	I70.699
Other atherosclerosis of nonbiological bypass graft(s) of the extremities, unspecified extremity	440.3	I70.699
Atherosclerosis of nonbiological bypass graft of lower extremity with other clinical manifestation	440.3	I70.699
Atherosclerosis of nonbiological bypass graft of extremity with other clinical manifestation	440.3	I70.699
Atherosclerosis of nonbiological bypass graft of lower extremity with other clinical manifestation, unspecified laterality	440.3	I70.699
Atherosclerosis of nonbiological bypass graft of extremity with other clinical manifestation, unspecified extremity	440.3	I70.699
Other atherosclerosis of nonbiological bypass graft(s) of the extremities, unspecified extremity		I70.699
Atherosclerosis of other type of bypass graft(s) of the extremities		I70.7
Unspecified atherosclerosis of other type of bypass graft(s) of the extremities		I70.70
Unsp athscl type of bypass of the extremities, right leg	440.3	I70.701
Atherosclerosis of other type of bypass graft of right lower extremity	440.3	I70.701

© 2021 Eberly LA et al. *JAMA Network Open*.

Unspecified atherosclerosis of other type of bypass graft(s) of the extremities, right leg	440.3	I70.701
Atherosclerosis of other type of bypass graft of right lower extremity, with unspecified presence of clinical manifestation	440.3	I70.701
Unspecified atherosclerosis of other type of bypass graft(s) of the extremities, right leg		I70.701
Unsp athscl type of bypass of the extremities, left leg	440.3	I70.702
Atherosclerosis of other type of bypass graft of left lower extremity	440.3	I70.702
Unspecified atherosclerosis of other type of bypass graft(s) of the extremities, left leg	440.3	I70.702
Atherosclerosis of other type of bypass graft of left lower extremity, with unspecified presence of clinical manifestation	440.3	I70.702
Unspecified atherosclerosis of other type of bypass graft(s) of the extremities, left leg		I70.702
Unsp athscl type of bypass of the extrm, bilateral legs	440.3	I70.703
Atherosclerosis of other type of bypass graft of both lower extremities	440.3	I70.703
Unspecified atherosclerosis of other type of bypass graft(s) of the extremities, bilateral legs	440.3	I70.703
Atherosclerosis of other type of bypass graft of both lower extremities, with unspecified presence of clinical manifestation	440.3	I70.703

© 2021 Eberly LA et al. *JAMA Network Open*.

Unspecified atherosclerosis of other type of bypass graft(s) of the extremities, bilateral legs		I70.703
Unsp athscl type of bypass of the extremities, oth extremity	440.3	I70.708
Atherosclerosis of other type of bypass graft of other extremity	440.3	I70.708
Unspecified atherosclerosis of other type of bypass graft(s) of the extremities, other extremity	440.3	I70.708
Atherosclerosis of other type of bypass graft of other extremity, with unspecified presence of clinical manifestation	440.3	I70.708
Unspecified atherosclerosis of other type of bypass graft(s) of the extremities, other extremity		I70.708
Unsp athscl type of bypass of the extrm, unsp extremity	440.3	I70.709
Atherosclerosis of other type of bypass graft of extremity	440.3	I70.709
Unspecified atherosclerosis of other type of bypass graft(s) of the extremities, unspecified extremity	440.3	I70.709
Atherosclerosis of other bypass graft of lower extremity	440.3	I70.709
Atherosclerosis of other bypass graft of lower extremity, unspecified laterality, with unspecified presence of clinical manifestation	440.3	I70.709
Atherosclerosis of other type of bypass graft of extremity, unspecified extremity, with unspecified presence of clinical manifestation	440.3	I70.709

© 2021 Eberly LA et al. *JAMA Network Open*.

Unspecified atherosclerosis of other type of bypass graft(s) of the extremities, unspecified extremity		I70.709
Atherosclerosis of other type of bypass graft(s) of the extremities with intermittent claudication		I70.71
Athscl type of bypass of the extrm w intrmt claud, right leg	440.3	I70.711
Atherosclerosis of other type of bypass graft of right lower extremity with intermittent claudication	440.3	I70.711
Atherosclerosis of other type of bypass graft(s) of the extremities with intermittent claudication, right leg	440.3	I70.711
Atherosclerosis of other type of bypass graft(s) of the extremities with intermittent claudication, right leg		I70.711
Athscl type of bypass of the extrm w intrmt claud, left leg	440.3	I70.712
Atherosclerosis of other type of bypass graft of left lower extremity with intermittent claudication	440.3	I70.712
Atherosclerosis of other type of bypass graft(s) of the extremities with intermittent claudication, left leg	440.3	I70.712
Atherosclerosis of other type of bypass graft(s) of the extremities with intermittent claudication, left leg		I70.712
Athscl type of bypass of the extrm w intrmt claud, bi legs	440.3	I70.713
Atherosclerosis of other type of bypass graft of both lower extremities with intermittent claudication	440.3	I70.713

© 2021 Eberly LA et al. *JAMA Network Open*.

Atherosclerosis of other type of bypass graft(s) of the extremities with intermittent claudication, bilateral legs	440.3	I70.713
Atherosclerosis of other type of bypass graft(s) of the extremities with intermittent claudication, bilateral legs		I70.713
Athscl type of bypass of the extrm w intrmt claud, oth extrm	440.3	I70.718
Atherosclerosis of other type of bypass graft of other extremity with intermittent claudication	440.3	I70.718
Atherosclerosis of other type of bypass graft(s) of the extremities with intermittent claudication, other extremity	440.3	I70.718
Atherosclerosis of other type of bypass graft(s) of the extremities with intermittent claudication, other extremity		I70.718
Athscl type of bypass of extrm w intrmt claud, unsp extrm	440.3	I70.719
Atherosclerosis of other type of bypass graft of extremity with intermittent claudication	440.3	I70.719
Atherosclerosis of other type of bypass graft(s) of the extremities with intermittent claudication, unspecified extremity	440.3	I70.719
Atherosclerosis of other bypass graft of lower extremity with intermittent claudication	440.30, 443.9	I70.719
Atherosclerosis of other bypass graft of lower extremity with intermittent claudication, unspecified laterality	440.30, 443.9	I70.719
Atherosclerosis of other type of bypass graft of extremity with intermittent claudication, unspecified extremity	440.3	I70.719

© 2021 Eberly LA et al. *JAMA Network Open*.

Atherosclerosis of other type of bypass graft(s) of the extremities with intermittent claudication, unspecified extremity		I70.719
Atherosclerosis of other type of bypass graft(s) of the extremities with rest pain		I70.72
Athscl type of bypass of the extrm w rest pain, right leg	440.3	I70.721
Atherosclerosis of other type of bypass graft of right lower extremity with rest pain	440.3	I70.721
Atherosclerosis of other type of bypass graft(s) of the extremities with rest pain, right leg	440.3	I70.721
Atherosclerosis of other type of bypass graft(s) of the extremities with rest pain, right leg		I70.721
Athscl type of bypass of the extrm w rest pain, left leg	440.3	I70.722
Atherosclerosis of other type of bypass graft of left lower extremity with rest pain	440.3	I70.722
Atherosclerosis of other type of bypass graft(s) of the extremities with rest pain, left leg	440.3	I70.722
Atherosclerosis of other type of bypass graft(s) of the extremities with rest pain, left leg		I70.722
Athscl type of bypass of the extrm w rest pain, bi legs	440.3	I70.723
Atherosclerosis of other type of bypass graft of both lower extremities with rest pain	440.3	I70.723

© 2021 Eberly LA et al. *JAMA Network Open*.

Atherosclerosis of other type of bypass graft(s) of the extremities with rest pain, bilateral legs	440.3	I70.723
Atherosclerosis of other type of bypass graft(s) of the extremities with rest pain, bilateral legs		I70.723
Athscl type of bypass of the extrm w rest pain, oth extrm	440.3	I70.728
Atherosclerosis of other type of bypass graft of other extremity with rest pain	440.3	I70.728
Atherosclerosis of other type of bypass graft(s) of the extremities with rest pain, other extremity	440.3	I70.728
Atherosclerosis of other type of bypass graft(s) of the extremities with rest pain, other extremity		I70.728
Athscl type of bypass of the extrm w rest pain, unsp extrm	440.3	I70.729
Atherosclerosis of other type of bypass graft of extremity with rest pain	440.3	I70.729
Atherosclerosis of other type of bypass graft(s) of the extremities with rest pain, unspecified extremity	440.3	I70.729
Atherosclerosis of other bypass graft of lower extremity with rest pain	440.3	I70.729
Atherosclerosis of other bypass graft of lower extremity with rest pain, unspecified laterality	440.3	I70.729
Atherosclerosis of other type of bypass graft of extremity with rest pain, unspecified extremity	440.3	I70.729
Atherosclerosis of other type of bypass graft(s) of the extremities with rest pain, unspecified extremity		I70.729

© 2021 Eberly LA et al. *JAMA Network Open*.

Atherosclerosis of other type of bypass graft(s) of the right leg with ulceration		I70.73
Athscl type of bypass of the right leg w ulceration of thigh	440.3	I70.731
Atherosclerosis of other type of bypass graft of right lower extremity with ulceration of thigh	440.3	I70.731
Atherosclerosis of other type of bypass graft(s) of the right leg with ulceration of thigh	440.3	I70.731
Atherosclerosis of other type of bypass graft(s) of the right leg with ulceration of thigh		I70.731
Atherosclerosis of other bypass graft of both lower extremities with bilateral ulceration of thigh	440.30, 707.11	I70.731, I70.741
Athscl type of bypass of the right leg w ulceration of calf	440.3	I70.732
Atherosclerosis of other type of bypass graft of right lower extremity with ulceration of calf	440.3	I70.732
Atherosclerosis of other type of bypass graft(s) of the right leg with ulceration of calf	440.3	I70.732
Atherosclerosis of other type of bypass graft(s) of the right leg with ulceration of calf		I70.732
Atherosclerosis of other bypass graft of both lower extremities with bilateral ulceration of calves	440.30, 707.12	I70.732, I70.742
Athscl type of bypass of the right leg w ulceration of ankle	440.3	I70.733
Atherosclerosis of other type of bypass graft of right lower extremity with ulceration of ankle	440.3	I70.733

© 2021 Eberly LA et al. *JAMA Network Open*.

Atherosclerosis of other type of bypass graft(s) of the right leg with ulceration of ankle	440.3	I70.733
Atherosclerosis of other type of bypass graft(s) of the right leg with ulceration of ankle		I70.733
Atherosclerosis of other bypass graft of both lower extremities with bilateral ulceration of ankles	440.30, 707.13	I70.733, I70.743
Athscl type of bypass of right leg w ulcer of heel and midft	440.3	I70.734
Atherosclerosis of other type of bypass graft(s) of the right leg with ulceration of heel and midfoot	440.3	I70.734
Atherosclerosis of other bypass graft of right lower extremity with ulceration of heel	440.30, 707.14	I70.734
Atherosclerosis of other bypass graft of right lower extremity with ulceration of midfoot	440.30, 707.14	I70.734
Atherosclerosis of other type of bypass graft(s) of the right leg with ulceration of heel and midfoot		I70.734
Atherosclerosis of other bypass graft of both lower extremities with bilateral ulceration of midfeet	440.30, 707.14	I70.734, I70.744
Atherosclerosis of other bypass graft of both lower extremities with bilateral ulceration of heels	440.30, 707.14	I70.734, I70.744
Athscl type of bypass of the right leg w ulcer oth prt foot	440.3	I70.735
Atherosclerosis of other type of bypass graft of right lower extremity with ulceration of other part of foot	440.3	I70.735

© 2021 Eberly LA et al. *JAMA Network Open*.

Atherosclerosis of other type of bypass graft(s) of the right leg with ulceration of other part of foot	440.3	I70.735
Atherosclerosis of other type of bypass graft(s) of the right leg with ulceration of other part of foot		I70.735
Atherosclerosis of other bypass graft of both lower extremities with bilateral ulceration of other part of feet	440.30, 707.15	I70.735, I70.745
Athscl type of bypass of right leg w ulcer oth prt low leg	440.3	I70.738
Atherosclerosis of other type of bypass graft of right lower extremity with ulceration of other part of lower leg	440.3	I70.738
Atherosclerosis of other type of bypass graft(s) of the right leg with ulceration of other part of lower leg	440.3	I70.738
Atherosclerosis of other type of bypass graft(s) of the right leg with ulceration of other part of lower leg		I70.738
Atherosclerosis of other bypass graft of both lower extremities with bilateral ulceration of other part of lower legs	440.30, 707.19	I70.738, I70.748
Athscl type of bypass of the right leg w ulcer of unsp site	440.3	I70.739
Atherosclerosis of other type of bypass graft of right lower extremity with ulceration	440.3	I70.739
Atherosclerosis of other type of bypass graft(s) of the right leg with ulceration of unspecified site	440.3	I70.739
Atherosclerosis of other type of bypass graft of right lower extremity with ulceration, unspecified ulceration site	440.3	I70.739

© 2021 Eberly LA et al. *JAMA Network Open*.

Atherosclerosis of other type of bypass graft(s) of the right leg with ulceration of unspecified site		I70.739
Atherosclerosis of other bypass graft of both lower extremities with bilateral ulceration	440.30, 707.10	I70.739, I70.749
Atherosclerosis of other bypass graft of both lower extremities with bilateral ulceration, unspecified ulceration site	440.30, 707.10	I70.739, I70.749
Atherosclerosis of other type of bypass graft(s) of the left leg with ulceration		I70.74
Athscl type of bypass of the left leg w ulceration of thigh	440.3	I70.741
Atherosclerosis of other type of bypass graft of left lower extremity with ulceration of thigh	440.3	I70.741
Atherosclerosis of other type of bypass graft(s) of the left leg with ulceration of thigh	440.3	I70.741
Atherosclerosis of other type of bypass graft(s) of the left leg with ulceration of thigh		I70.741
Athscl type of bypass of the left leg w ulceration of calf	440.3	I70.742
Atherosclerosis of other type of bypass graft of left lower extremity with ulceration of calf	440.3	I70.742
Atherosclerosis of other type of bypass graft(s) of the left leg with ulceration of calf	440.3	I70.742
Atherosclerosis of other type of bypass graft(s) of the left leg with ulceration of calf		I70.742
Athscl type of bypass of the left leg w ulceration of ankle	440.3	I70.743

© 2021 Eberly LA et al. *JAMA Network Open*.

Atherosclerosis of other type of bypass graft of left lower extremity with ulceration of ankle	440.3	I70.743
Atherosclerosis of other type of bypass graft(s) of the left leg with ulceration of ankle	440.3	I70.743
Atherosclerosis of other type of bypass graft(s) of the left leg with ulceration of ankle		I70.743
Athscl type of bypass of left leg w ulcer of heel and midft	440.3	I70.744
Atherosclerosis of other type of bypass graft(s) of the left leg with ulceration of heel and midfoot	440.3	I70.744
Atherosclerosis of other bypass graft of left lower extremity with ulceration of midfoot	440.30, 707.14	I70.744
Atherosclerosis of other bypass graft of left lower extremity with ulceration of heel	440.30, 707.14	I70.744
Atherosclerosis of other type of bypass graft(s) of the left leg with ulceration of heel and midfoot		I70.744
Athscl type of bypass of the left leg w ulcer oth prt foot	440.3	I70.745
Atherosclerosis of other type of bypass graft of left lower extremity with ulceration of other part of foot	440.3	I70.745
Atherosclerosis of other type of bypass graft(s) of the left leg with ulceration of other part of foot	440.3	I70.745
Atherosclerosis of other type of bypass graft(s) of the left leg with ulceration of other part of foot		I70.745
Athscl type of bypass of left leg w ulcer oth prt low leg	440.3	I70.748

© 2021 Eberly LA et al. *JAMA Network Open*.

Atherosclerosis of other type of bypass graft of left lower extremity with ulceration of other part of lower leg	440.3	I70.748
Atherosclerosis of other type of bypass graft(s) of the left leg with ulceration of other part of lower leg	440.3	I70.748
Atherosclerosis of other type of bypass graft(s) of the left leg with ulceration of other part of lower leg		I70.748
Athscl type of bypass of the left leg w ulcer of unsp site	440.3	I70.749
Atherosclerosis of other type of bypass graft of left lower extremity with ulceration	440.3	I70.749
Atherosclerosis of other type of bypass graft(s) of the left leg with ulceration of unspecified site	440.3	I70.749
Atherosclerosis of other type of bypass graft of left lower extremity with ulceration, unspecified ulceration site	440.3	I70.749
Atherosclerosis of other type of bypass graft(s) of the left leg with ulceration of unspecified site		I70.749
Athscl type of bypass graft(s) of extremity w ulceration	440.30, 707.9	I70.75
Atherosclerosis of other type of bypass graft of other extremity with ulceration	440.3	I70.75
Atherosclerosis of other type of bypass graft(s) of other extremity with ulceration	440.3	I70.75
Atherosclerosis of other bypass graft of lower extremity with ulceration of other part of lower leg	440.30, 707.19	I70.75
Atherosclerosis of other bypass graft of lower extremity with ulceration of calf	440.30, 707.12	I70.75

© 2021 Eberly LA et al. *JAMA Network Open*.

Atherosclerosis of other bypass graft of lower extremity with ulceration of ankle	440.30, 707.13	I70.75
Atherosclerosis of other bypass graft of lower extremity with ulceration of heel	440.30, 707.14	I70.75
Atherosclerosis of other bypass graft of lower extremity with ulceration	440.30, 707.10	I70.75
Atherosclerosis of other bypass graft of lower extremity with ulceration of thigh	440.30, 707.11	I70.75
Atherosclerosis of other bypass graft of lower extremity with ulceration of midfoot	440.30, 707.14	I70.75
Atherosclerosis of other bypass graft of lower extremity with ulceration of other part of foot	440.30, 707.15	I70.75
Atherosclerosis of other bypass graft of extremity with ulceration	440.30, 707.9	I70.75
Atherosclerosis of other bypass graft of lower extremity with ulceration of other part of foot, unspecified laterality	440.30, 707.15	I70.75
Atherosclerosis of other bypass graft of lower extremity with ulceration of calf, unspecified laterality	440.30, 707.12	I70.75
Atherosclerosis of other bypass graft of lower extremity with ulceration of thigh, unspecified laterality	440.30, 707.11	I70.75
Atherosclerosis of other bypass graft of lower extremity with ulceration of ankle, unspecified laterality	440.30, 707.13	I70.75
Atherosclerosis of other bypass graft of lower extremity with ulceration, unspecified laterality, unspecified ulceration site	440.30, 707.10	I70.75
Atherosclerosis of other bypass graft of lower extremity with ulceration of other part of lower leg, unspecified laterality	440.30, 707.19	I70.75

© 2021 Eberly LA et al. *JAMA Network Open*.

Atherosclerosis of other bypass graft of lower extremity with ulceration of midfoot, unspecified laterality	440.30, 707.14	I70.75
Atherosclerosis of other bypass graft of lower extremity with ulceration of heel, unspecified laterality	440.30, 707.14	I70.75
Atherosclerosis of other bypass graft of extremity with ulceration, unspecified extremity	440.30, 707.9	I70.75
Atherosclerosis of other type of bypass graft(s) of other extremity with ulceration		I70.75
Atherosclerosis of other type of bypass graft(s) of the extremities with gangrene		I70.76
Athscl type of bypass of the extrm w gangrene, right leg	440.3	I70.761
Atherosclerosis of other type of bypass graft of right lower extremity with gangrene	440.3	I70.761
Atherosclerosis of other type of bypass graft(s) of the extremities with gangrene, right leg	440.3	I70.761
Atherosclerosis of other type of bypass graft(s) of the extremities with gangrene, right leg		I70.761
Athscl type of bypass of the extrm w gangrene, left leg	440.3	I70.762
Atherosclerosis of other type of bypass graft of left lower extremity with gangrene	440.3	I70.762
Atherosclerosis of other type of bypass graft(s) of the extremities with gangrene, left leg	440.3	I70.762
Atherosclerosis of other type of bypass graft(s) of the extremities with gangrene, left leg		I70.762

© 2021 Eberly LA et al. *JAMA Network Open*.

Athscl type of bypass of the extrm w gangrene, bi legs	440.3	I70.763
Atherosclerosis of other type of bypass graft of both lower extremities with gangrene	440.3	I70.763
Atherosclerosis of other type of bypass graft(s) of the extremities with gangrene, bilateral legs	440.3	I70.763
Atherosclerosis of other type of bypass graft(s) of the extremities with gangrene, bilateral legs		I70.763
Athscl type of bypass of the extrm w gangrene, oth extremity	440.3	I70.768
Atherosclerosis of other type of bypass graft of other extremity with gangrene	440.3	I70.768
Atherosclerosis of other type of bypass graft(s) of the extremities with gangrene, other extremity	440.3	I70.768
Atherosclerosis of other type of bypass graft(s) of the extremities with gangrene, other extremity		I70.768
Athscl type of bypass of the extrm w gangrene, unsp extrm	440.3	I70.769
Atherosclerosis of other type of bypass graft of extremity with gangrene	440.3	I70.769
Atherosclerosis of other type of bypass graft(s) of the extremities with gangrene, unspecified extremity	440.3	I70.769
Atherosclerosis of other bypass graft of lower extremity with gangrene	440.30, 785.4	I70.769
Atherosclerosis of other bypass graft of lower extremity with gangrene, unspecified laterality	440.30, 785.4	I70.769

© 2021 Eberly LA et al. *JAMA Network Open*.

Atherosclerosis of other type of bypass graft of extremity with gangrene, unspecified extremity	440.3	I70.769
Atherosclerosis of other type of bypass graft(s) of the extremities with gangrene, unspecified extremity		I70.769
Other atherosclerosis of other type of bypass graft(s) of the extremities		I70.79
Oth athscl type of bypass of the extremities, right leg	440.3	I70.791
Other atherosclerosis of other type of bypass graft(s) of the extremities, right leg	440.3	I70.791
Atherosclerosis of other bypass graft of right lower extremity with other clinical manifestation	440.3	I70.791
Other atherosclerosis of other type of bypass graft(s) of the extremities, right leg		I70.791
Oth athscl type of bypass of the extremities, left leg	440.3	I70.792
Other atherosclerosis of other type of bypass graft(s) of the extremities, left leg	440.3	I70.792
Atherosclerosis of other bypass graft of left lower extremity with other clinical manifestation	440.3	I70.792
Other atherosclerosis of other type of bypass graft(s) of the extremities, left leg		I70.792
Oth athscl type of bypass of the extremities, bilateral legs	440.3	I70.793
Other atherosclerosis of other type of bypass graft(s) of the extremities, bilateral legs	440.3	I70.793
Atherosclerosis of other bypass graft of both lower extremities with other clinical manifestation	440.3	I70.793

© 2021 Eberly LA et al. *JAMA Network Open*.

Other atherosclerosis of other type of bypass graft(s) of the extremities, bilateral legs		I70.793
Oth athscl type of bypass of the extremities, oth extremity	440.3	I70.798
Other atherosclerosis of other type of bypass graft(s) of the extremities, other extremity	440.3	I70.798
Atherosclerosis of other bypass graft of other extremity with other clinical manifestation	440.3	I70.798
Other atherosclerosis of other type of bypass graft(s) of the extremities, other extremity		I70.798
Oth athscl type of bypass of the extremities, unsp extremity	440.3	I70.799
Other atherosclerosis of other type of bypass graft(s) of the extremities, unspecified extremity	440.3	I70.799
Atherosclerosis of other bypass graft of extremity with other clinical manifestation	440.3	I70.799
Atherosclerosis of other bypass graft of lower extremity with other clinical manifestation	440.3	I70.799
Atherosclerosis of other bypass graft of lower extremity with other clinical manifestation, unspecified laterality	440.3	I70.799
Atherosclerosis of other bypass graft of extremity with other clinical manifestation, unspecified extremity	440.3	I70.799
Other atherosclerosis of other type of bypass graft(s) of the extremities, unspecified extremity		I70.799

© 2021 Eberly LA et al. *JAMA Network Open*.

Atherosclerosis of other specified arteries	440.8	I70.8
Atherosclerosis NEC	440.8	I70.8
Atherosclerosis of arteries	440.8	I70.8
Stenosis of left hepatic artery	440.8	I70.8
Hepatic artery stenosis, left	440.8	I70.8
Stenosis of right hepatic artery	440.8	I70.8
Hepatic artery stenosis, right	440.8	I70.8
Atherosclerosis of hypogastric artery	440.8	I70.8
Hypogastric atherosclerosis	440.8	I70.8
Atherosclerosis of artery	440.8	I70.8
Stenosis of left hepatic artery of transplanted liver	440.8	I70.8
Arterial atherosclerosis	440.8	I70.8
Hepatic artery stenosis, left, transplanted liver	440.8	I70.8
Atherosclerosis of celiac artery	440.8	I70.8
Celiac artery atherosclerosis	440.8	I70.8
Atherosclerotic stenosis of brachiocephalic artery	440.8	I70.8
Atherosclerotic stenosis of innominate artery	440.8	I70.8
Atherosclerosis of other arteries	440.8	I70.8
Atherosclerosis of right iliac artery	440.8	I70.8
Atherosclerosis of both iliac arteries	440.8	I70.8
Atherosclerosis of other arteries (CODE)	440.8	I70.8
Atherosclerosis of other arteries		I70.8
Arteriosclerotic retinopathy	440.8, 362.13	I70.8, H35.09
Retinal arteriosclerosis	440.8, 362.13	I70.8, H35.09
Retinal artery plaque	440.8, 362.13	I70.8, H35.09
Stenosis of right hepatic artery of transplanted liver	440.8, V42.7	I70.8, Z94.4
Other and unspecified atherosclerosis		I70.9

© 2021 Eberly LA et al. *JAMA Network Open*.

Generalized and unspecified atherosclerosis	440.9	I70.90
Arteriosclerotic vascular disease	440.9	I70.90
Arterial degeneration	440.9	I70.90
Arteriovascular degeneration	440.9	I70.90
Vascular degeneration	440.9	I70.90
Atheroma of artery	440.9	I70.90
Endarteritis obliterans	440.9	I70.90
Senile endarteritis	440.9	I70.90
Arteriosclerosis	440.9	I70.90
Atheroma	440.9	I70.90
Atheromatous plaque	440.9	I70.90
Atherosclerotic plaque	440.9	I70.90
ASO (arteriosclerosis obliterans)	440.9	I70.90
Arteriosclerosis obliterans	440.9	I70.90
Senile arteriosclerosis	440.9	I70.90
Atherosclerosis	440.9	I70.90
Arterial vascular disease	440.9	I70.90
Arterioloscleroses	440.9	I70.90
Arteriovascular disease	440.9	I70.90
AS (atherosclerosis)	440.9	I70.90
Atheromatosis	440.9	I70.90
Atheromatous degeneration	440.9	I70.90
Atherosclerotic vascular disease	440.9	I70.90
Endarteritis deformans	440.9	I70.90
Arterial fatty streak	440.9	I70.90
Arterial fatty streaks	440.9	I70.90
Arteriolosclerosis	440.9	I70.90
Arterial occlusion due to arteriosclerosis	440.9	I70.90
ASVD (arteriosclerotic vascular disease)	440.9	I70.90
Class IV atherosclerotic vascular disease	440.9	I70.90
Class III atherosclerotic vascular disease	440.9	I70.90
Class II atherosclerotic vascular disease	440.9	I70.90
Class I atherosclerotic vascular disease	440.9	I70.90
Arteriosclerosis of saphenous vein	440.9	I70.90
Atherosclerotic occlusive disease	440.9	I70.90

© 2021 Eberly LA et al. *JAMA Network Open*.

Hard blood vessel	440.9	I70.90
Unspecified atherosclerosis	440.9	I70.90
Vascular hyalinosis	440.9	I70.90
Unspecified atherosclerosis		I70.90
Calcification of multiple joints and arteries	719.80, 440.9	I70.90, M25.80
Generalized atherosclerosis	440.9	I70.91
Atherosclerosis, generalized	440.9	I70.91
Generalized atherosclerosis without gangrene	440.9	I70.91
Generalized atherosclerosis		I70.91
Chronic total occlusion of artery of the extremities	440.4	I70.92
Complete occlusion of artery of the extremities	440.4	I70.92
Total occlusion of artery of the extremities	440.4	I70.92
Extremity artery chronic total occlusion	440.4	I70.92
Chronic total occlusion of artery of extremity	440.4	I70.92
Chronic total occlusion of artery of the extremities (CODE)	440.4	I70.92
Chronic total occlusion of artery of the extremities		I70.92
Intramural aortic hematoma	441	I71.00
Dissection of aorta, abdominal	441.02	I71.02
Dissecting aortic aneurysm (any part), abdominal	441.02	I71.02
Dissecting aortic aneurysm, abdominal	441.02	I71.02
Dissection of abdominal aorta	441.02	I71.02
Dissecting abdominal aortic aneurysm	441.02	I71.02
Dissecting AAA (abdominal aortic aneurysm)	441.02	I71.02
Aortic dissection, abdominal	441.02	I71.02
Abdominal aortic aneurysm dissection	441.02	I71.02

© 2021 Eberly LA et al. *JAMA Network Open*.

Dissecting abdominal aortic aneurysm (AAA)	441.02	I71.02
Dissection of abdominal aorta		I71.02
Abdominal aneurysm, ruptured	441.3	I71.3
Ruptured abdominal aortic aneurysm	441.3	I71.3
Aneurysm, abdominal aorta, ruptured	441.3	I71.3
Aneurysm, aorta, abdominal, ruptured	441.3	I71.3
AAA (abdominal aortic aneurysm, ruptured)	441.3	I71.3
RAAA (ruptured abdominal aortic aneurysm)	441.3	I71.3
Abdominal aortic aneurysm, ruptured	441.3	I71.3
Abdominal aortic aneurysm rupture	441.3	I71.3
Aneurysm, abdominal aortic, with rupture	441.3	I71.3
Ruptured abdominal aortic aneurysm (AAA)	441.3	I71.3
Abdominal aortic aneurysm, ruptured		I71.3
Abdominal aneurysm without mention of rupture	441.4	I71.4
Abdominal aortic aneurysm without rupture	441.4	I71.4
Abdominal aortic aneurysm	441.4	I71.4
Aneurysm, abdominal aortic	441.4	I71.4
Aortic aneurysm, abdominal	441.4	I71.4
AAA (abdominal aortic aneurysm)	441.4	I71.4
Abdominal aneurysm	441.4	I71.4
AAA (abdominal aortic aneurysm) without rupture	441.4	I71.4
Enlarging abdominal aortic aneurysm	441.4	I71.4
Abdominal aortic aneurysm without mention of rupture	441.4	I71.4
Leaking abdominal aortic aneurysm	441.4	I71.4
Aneurysm of abdominal aorta	441.4	I71.4
Aneurysm of supraceliac aorta	441.4	I71.4

© 2021 Eberly LA et al. *JAMA Network Open*.

Supraceliac aortic aneurysm	441.4	I71.4
Suprarenal aortic aneurysm	441.4	I71.4
Aneurysm of infrarenal abdominal aorta	441.4	I71.4
Inflammatory abdominal aortic aneurysm	441.4	I71.4
Recurrent abdominal aortic aneurysm	441.4	I71.4
Aneurysm of suprarenal aorta	441.4	I71.4
Abdominal aortic aneurysm, not a candidate for repair	441.4	I71.4
AAA (abdominal aortic aneurysm), not a candidate for repair	441.4	I71.4
Aneurysm of abdominal aorta branch vessel	441.4	I71.4
Abdominal aortic aneurysm greater than 39 mm in diameter	441.4	I71.4
Abdominal aortic aneurysm 35 to 39 mm in diameter	441.4	I71.4
Abdominal aortic aneurysm 30 to 34 mm in diameter	441.4	I71.4
Abdominal aortic aneurysm (AAA), 30-34 mm diameter	441.4	I71.4
Abdominal aortic aneurysm (AAA), 35-39 mm diameter	441.4	I71.4
Abdominal aortic aneurysm (AAA) >39 mm diameter	441.4	I71.4
Aneurysm of abdominal vessel	441.4	I71.4
Abdominal aortic aneurysm, without rupture	441.4	I71.4
Abdominal aortic aneurysm (AAA)	441.4	I71.4
Abdominal aortic aneurysm (AAA) without rupture	441.4	I71.4
Abdominal aortic aneurysm (AAA) 30 to 34 mm in diameter	441.4	I71.4
Abdominal aortic aneurysm (AAA), not a candidate for repair	441.4	I71.4
Abdominal aortic aneurysm (AAA) greater than 39 mm in diameter	441.4	I71.4

© 2021 Eberly LA et al. *JAMA Network Open*.

Enlarging abdominal aortic aneurysm (AAA)	441.4	I71.4
Leaking abdominal aortic aneurysm (AAA)	441.4	I71.4
Recurrent abdominal aortic aneurysm (AAA)	441.4	I71.4
Abdominal aortic aneurysm (AAA) 35 to 39 mm in diameter	441.4	I71.4
Abdominal aortic aneurysm (AAA) greater than 5.0 cm in diameter in female	441.4	I71.4
Abdominal aortic aneurysm (AAA) 3.0 cm to 5.5 cm in diameter in male	441.4	I71.4
Abdominal aortic aneurysm (AAA) 3.0 cm to 5.0 cm in diameter in female	441.4	I71.4
Abdominal aortic aneurysm (AAA) greater than 5.5 cm in diameter in male	441.4	I71.4
Abdominal aortic aneurysm, without rupture		I71.4
Penetrating ulcer of aorta	441.9	I71.9
Other specified peripheral vascular diseases		I73.8
Other peripheral vascular disease(443.89)	443.89	I73.89
Other specified peripheral vascular diseases (CODE)	443.89	I73.89
Other specified peripheral vascular diseases		I73.89
Peripheral vascular disease, unspecified	443.9	I73.9
Intermittent claudication	443.9	I73.9
Claudication, intermittent	443.9	I73.9
Peripheral vascular disease	443.9	I73.9
Peripheral vascular disorder	443.9	I73.9
Vascular disease, peripheral	443.9	I73.9
PVD (peripheral vascular disease)	443.9	I73.9
Claudication	443.9	I73.9
IC (intermittent claudication)	443.9	I73.9

© 2021 Eberly LA et al. *JAMA Network Open*.

Asymptomatic peripheral vascular disease	443.9	I73.9
Asymptomatic PVD (peripheral vascular disease)	443.9	I73.9
Peripheral vascular disease with claudication	443.9	I73.9
PVD (peripheral vascular disease) with claudication	443.9	I73.9
Peripheral arterial disease	443.9	I73.9
PAD (peripheral artery disease)	443.9	I73.9
Peripheral vascular occlusive disease	443.9	I73.9
Peripheral vascular obstructive disease	443.9	I73.9
Lower extremity arterial insufficiency, severe, right	443.9	I73.9
Severe arterial insufficiency of left lower extremity	443.9	I73.9
Severe arterial insufficiency of right lower extremity	443.9	I73.9
Lower extremity arterial insufficiency, severe, left	443.9	I73.9
Peripheral artery insufficiency	443.9	I73.9
Insufficiency, arterial, peripheral	443.9	I73.9
Peripheral artery disease	443.9	I73.9
Claudication of gluteal region	443.9	I73.9
Gluteal claudication	443.9	I73.9
Femoro-popliteal artery disease	443.9	I73.9
Peripheral venous engorgement	443.9	I73.9
Vascular claudication	443.9	I73.9
Peripheral vascular disease, asymptomatic	443.9	I73.9
Peripheral artery vasospasm	443.9	I73.9
Vasospasm of peripheral artery	443.9	I73.9
Claudication in peripheral vascular disease	443.9	I73.9
Posterior tibial artery insufficiency	443.9	I73.9
Arterial insufficiency, posterior tibial	443.9	I73.9
Peripheral vascular disease with pain at rest	443.9	I73.9

© 2021 Eberly LA et al. *JAMA Network Open*.

Secondary peripheral vascular disease	443.81	I73.9
Peripheral vascular disease, secondary	443.81	I73.9
Claudication of calf muscles	443.9	I73.9
Bilateral claudication of lower limb	443.9	I73.9
Ischemic rest pain of lower extremity	443.9	I73.9
Class IV claudication	443.9	I73.9
Claudication, class IV	443.9	I73.9
Severe claudication	443.9	I73.9
Claudication, class III	443.9	I73.9
Moderate claudication	443.9	I73.9
Claudication, class II	443.9	I73.9
Class II claudication	443.9	I73.9
Mild claudication	443.9	I73.9
Class I claudication	443.9	I73.9
Claudication, class I	443.9	I73.9
Peripheral vascular insufficiency	443.9	I73.9
Right leg claudication	443.9	I73.9
Claudication of right lower extremity	443.9	I73.9
Cold foot with peripheral vascular disease	443.9	I73.9
Left leg claudication	443.9	I73.9
Claudication of left lower extremity	443.9	I73.9
Dysvascular foot	443.9	I73.9
Peripheral vascular disease of foot	443.9	I73.9
Vascular disorder of extremity	443.9	I73.9
Claudication of lower extremity	443.9	I73.9
Peripheral neurovascular dysfunction	443.9	I73.9
Peripheral vasodilation	443.9	I73.9
Peripheral vascular disease with cramping and inability to walk	443.9	I73.9
Peripheral vascular disease of extremity	443.9	I73.9
Peripheral vascular disease of extremity with claudication	443.9	I73.9
Peripheral vascular disease of lower extremity	443.9	I73.9
Severe peripheral arterial disease	443.9	I73.9

© 2021 Eberly LA et al. *JAMA Network Open*.

Arterial insufficiency of lower extremity	443.9	I73.9
Poor peripheral circulation	443.9	I73.9
Claudication of both lower extremities	443.9	I73.9
Gangrene due to arterial insufficiency	443.9, 785.4	I73.9
Ischemic foot pain at rest	443.9	I73.9
Ischemic foot pain when walking	443.9	I73.9
Ischemic pain of foot at rest	443.9	I73.9
Pain of foot due to ischemia when walking	443.9	I73.9
Claudication of upper extremity	443.9	I73.9
Cutaneous collagenous vasculopathy	443.9	I73.9
Peripheral vascular disease, unspecified		I73.9
Neuropathy due to peripheral vascular disease	357.4	I73.9, G63
Tissue necrosis with gangrene in peripheral vascular disease	443.9, 785.4	I73.9, I96
Peripheral vascular disease of lower extremity with ulceration	443.9, 707.10	I73.9, L97.909
Atherosclerosis of native arteries of the extremities with ulceration(440.23)	440.23	I73.9, L98.499
Loss of protective sensation of skin of deformed foot with peripheral vascular disease of foot	443.9, 782.0, 736.70	I73.9, M21.969, R20.8
Loss of protective sensation of skin of foot with peripheral vascular disease of foot	443.9, 782.0	I73.9, R20.8
Arterial insufficiency	447.1	I77.1
Arterial stenosis	447.1	I77.1
Artery stenosis	447.1	I77.1
Stenosis of artery	447.1	I77.1
Stenosis of hepatic artery	447.1	I77.1
Stenosis of radial artery	447.1	I77.1
Radial artery stenosis	447.1	I77.1
Narrowing of femoral artery in both legs	447.1	I77.1
Stenosis of left subclavian artery	447.1	I77.1
Subclavian artery stenosis, left	447.1	I77.1
Stenosis of right subclavian artery	447.1	I77.1

© 2021 Eberly LA et al. *JAMA Network Open*.

Subclavian artery stenosis, right	447.1	I77.1
Stenosis of pancreatic artery	447.1	I77.1
Pancreas artery stenosis	447.1	I77.1
Diffuse narrowing of hepatic artery	447.1	I77.1
Hepatic artery, diffuse narrowing	447.1	I77.1
Stenosis of brachiocephalic artery	447.1	I77.1
Stenosis of left brachiocephalic artery	447.1	I77.1
Brachiocephalic artery stenosis, left	447.1	I77.1
Stenosis of right brachiocephalic artery	447.1	I77.1
Brachiocephalic artery stenosis, right	447.1	I77.1
Superior mesenteric artery stenosis	557.1	I77.1
Stenosis of iliac artery	447.1	I77.1
Stenosis of left iliac artery	447.1	I77.1
Iliac artery stenosis, left	447.1	I77.1
Stenosis of right iliac artery	447.1	I77.1
Iliac artery stenosis, right	447.1	I77.1
Bilateral iliac artery stenosis	447.1	I77.1
Iliac artery stenosis, bilateral	447.1	I77.1
Bilateral pelvic artery narrowing	447.1	I77.1
SMA stenosis	557.1	I77.1
Subclavian artery stenosis	447.1	I77.1
Subclavian arterial stenosis	447.1	I77.1
Stenosis of subclavian artery	447.1	I77.1
Arterial occlusion due to stenosis	447.1	I77.1
Right iliac artery stenosis	447.1	I77.1
Hepatic artery stenosis	447.1	I77.1
Innominate artery stenosis	447.1	I77.1
Stenosis of artery in neck	447.1	I77.1
Narrowing of artery in neck	447.1	I77.1
Kinking of left iliac artery	447.1	I77.1
Stenosis of artery of abdomen	447.1	I77.1
Stenosis of iliac artery, unspecified laterality	447.1	I77.1
Stricture of artery		I77.1
Arterial insufficiency with ischemic ulcer	447.1, 707.9	I77.1, L98.499
Carotid ulcer	447.2, 433.10	I77.2, I65.29

© 2021 Eberly LA et al. *JAMA Network Open*.

Deep venous thrombosis associated with coronary artery bypass graft, right	453.40, 414.04	I82.401, I25.810
Deep vein thrombosis associated with coronary artery bypass graft, right	453.40, 414.04	I82.401, I25.810
Deep venous thrombosis associated with coronary artery bypass graft, left	453.40, 414.04	I82.402, I25.810
Deep vein thrombosis associated with coronary artery bypass graft, left	453.40, 414.04	I82.402, I25.810
Deep vein thrombosis associated with coronary artery bypass graft, bilateral	453.40, 414.04	I82.403, I25.810
Deep venous thrombosis associated with coronary artery bypass graft, bilateral	453.40, 414.04	I82.403, I25.810
Deep venous thrombosis associated with coronary artery bypass graft, unspecified laterality	453.40, 414.04	I82.409, I25.810
Deep vein thrombosis associated with coronary artery bypass graft, unspecified laterality	453.40, 414.04	I82.409, I25.810
Gangrene of right lower extremity due to atherosclerosis	785.4, 440.20	I96, I70.201
Gangrene of left lower extremity due to atherosclerosis	785.4, 440.20	I96, I70.202
Gangrene due to peripheral vascular disease	785.4, 443.9	I96, I73.9
Acute myocardial infarction, true posterior wall infarction, subsequent episode of care	410.62	IMO0001
Acute coronary occlusion without mycocardial infarction	411.81	IMO0001
Maternal coronary artery disease	IMO0001	IMO0001
Acute non-ST segment elevation myocardial infarction (STEMI) following previous myocardial infarction	IMO0001	IMO0001
Aphasic stroke	IMO0002	IMO0002
Stroke syndrome	IMO0002	IMO0002

© 2021 Eberly LA et al. *JAMA Network Open*.

Apraxia due to cerebrovascular accident	IMO0002	IMO0002
Apraxia due to stroke	IMO0002	IMO0002
Aphasia with stroke	IMO0002	IMO0002
Aphasia due to stroke	IMO0002	IMO0002
Cognitive dysfunction due to stroke	IMO0002	IMO0002
Stroke-related cognitive dysfunction	IMO0002	IMO0002
Cognitive deficit due to multiple subcortical infarcts	IMO0002	IMO0002
Mood disorder due to cerebrovascular accident	IMO0002	IMO0002
Mood disorder due to stroke	IMO0002	IMO0002
Dysarthria due to cerebrovascular accident	IMO0002	IMO0002
Monoplegia, upper extremity, post-stroke	IMO0002	IMO0002
Monoplegia, dominant upper extremity, post-stroke	IMO0002	IMO0002
Monoplegia, non-dominant upper extremity, post-stroke	IMO0002	IMO0002
Monoplegia, non-dominant lower extremity, post-stroke	IMO0002	IMO0002
Lack of coordination due to stroke	IMO0002	IMO0002
Altered sensation due to stroke	IMO0002	IMO0002
Bilateral paralysis complicating stroke	IMO0002	IMO0002
Apraxia complicating stroke	IMO0002	IMO0002
Monoplegia of non-dominant leg as complication of stroke	IMO0002	IMO0002
Monoplegia of arm as complication of stroke	IMO0002	IMO0002
Monoplegia of dominant arm as complication of stroke	IMO0002	IMO0002
Monoplegia of dominant leg as complication of stroke	IMO0002	IMO0002
Monoplegia of leg as complication of stroke	IMO0002	IMO0002
Monoplegia of non-dominant arm as complication of stroke	IMO0002	IMO0002

© 2021 Eberly LA et al. *JAMA Network Open*.

Nondominant hemiplegia as complication of stroke	434.91, 342.92	IMO0002
Paralysis as complication of stroke	IMO0002	IMO0002
Paralysis of nondominant side as complication of stroke	IMO0002	IMO0002
Depression due to stroke	IMO0002	IMO0002
Personality change due to cerebrovascular accident	IMO0002	IMO0002
Personality change due to stroke	IMO0002	IMO0002
Aphasia complicating stroke	IMO0002	IMO0002
Dysarthria due to cerebellar stroke	IMO0002	IMO0002
Cerebrovascular accident with cognitive communication deficit	IMO0002	IMO0002
Weakness due to cerebrovascular accident	IMO0002	IMO0002
Neurological deficit due to ischemic stroke	IMO0002	IMO0002
Combined receptive and expressive aphasia due to cerebrovascular accident	IMO0002	IMO0002
Combined receptive and expressive aphasia due to stroke	IMO0002	IMO0002
Unilateral paralysis due to cerebrovascular accident	IMO0002	IMO0002
Monoplegia of lower extremity affecting dominant side as complication of stroke	IMO0002	IMO0002
Monoplegia of lower extremity as complication of stroke	IMO0002	IMO0002
Monoplegia of lower extremity affecting non-dominant side as complication of stroke	IMO0002	IMO0002
Monoplegia of upper extremity affecting non-dominant side as complication of stroke	IMO0002	IMO0002
Monoplegia of upper extremity as complication of stroke	IMO0002	IMO0002

© 2021 Eberly LA et al. *JAMA Network Open*.

Monoplegia of upper extremity affecting dominant side as complication of stroke	IMO0002	IMO0002
Nonintractable persistent migraine aura with cerebral infarction	IMO0002	IMO0002
Dysarthria due to cerebrovascular accident (CVA)	IMO0002	IMO0002
Mood disorder due to cerebrovascular accident (CVA)	IMO0002	IMO0002
Personality change due to cerebrovascular accident (CVA)	IMO0002	IMO0002
Apraxia due to cerebrovascular accident (CVA)	IMO0002	IMO0002
Combined receptive and expressive aphasia due to cerebrovascular accident (CVA)	IMO0002	IMO0002
Cerebrovascular accident (CVA) with cognitive communication deficit	IMO0002	IMO0002
Weakness due to cerebrovascular accident (CVA)	IMO0002	IMO0002
Unilateral paralysis due to cerebrovascular accident (CVA)	IMO0002	IMO0002
Paralysis of face due to cerebrovascular accident (CVA)	438.5	IMO0002
Facial paralysis due to stroke	438.5	IMO0002
Hemiplegia of non-dominant side as complication of cerebrovascular accident (CVA)	434.91, 342.92	IMO0002
Spastic hemiplegia of right nondominant side due to infarction of brain	IMO0002	IMO0002
Hemiparesis due to cerebral infarction	438.2	IMO0002
Spastic hemiparesis due to cerebral infarction	IMO0002	IMO0002
Hemiparesis of left dominant side due to cerebral infarction	438.21	IMO0002

© 2021 Eberly LA et al. *JAMA Network Open*.

Spastic hemiparesis of left nondominant side due to cerebral infarction	IMO0002	IMO0002
Spastic hemiparesis of left dominant side due to cerebral infarction	IMO0002	IMO0002
Hemiparesis of right dominant side due to cerebral infarction	438.21	IMO0002
Hemiparesis of right nondominant side due to cerebral infarction	438.22	IMO0002
Hemiparesis of left nondominant side due to cerebral infarction	438.22	IMO0002
Spastic hemiparesis of right dominant side due to cerebral infarction	IMO0002	IMO0002
Spastic hemiparesis of right nondominant side due to cerebral infarction	IMO0002	IMO0002
Facial droop due to stroke	IMO0002	IMO0002
Hemiplegia, nondominant side S/P CVA (cerebrovascular accident)	IMO0002	IMO0002
Hemiplegia of nondominant side following cerebrovascular accident	IMO0002	IMO0002
Hemiplegia affecting non-dominant side, post-stroke	IMO0002	IMO0002
Hemiplegia of nondominant side following CVA (cerebrovascular accident)	IMO0002	IMO0002
Hemiplegia of non-dominant side following cerebrovascular accident (CVA)	IMO0002	IMO0002
Monoplegia, upper limb, S/P CVA (cerebrovascular accident)	IMO0002	IMO0002
Monoplegia of upper limb following cerebrovascular accident	IMO0002	IMO0002
Monoplegia of upper limb following CVA (cerebrovascular accident)	IMO0002	IMO0002
CVA, old, monoplegia upper limb	IMO0002	IMO0002
Monoplegia of upper extremity following cerebrovascular accident	IMO0002	IMO0002
Monoplegia of upper extremity following cerebrovascular accident (CVA)	IMO0002	IMO0002

© 2021 Eberly LA et al. *JAMA Network Open*.

Postoperative myocardial infarction	997.1, 410.90	IMO0002
Chronic vascular insufficiency of intestine	557.1	K55.1
Chronic intestinal ischemic syndrome	557.1	K55.1
Chronic ischemic colitis	557.1	K55.1
Chronic ischemic enteritis	557.1	K55.1
Chronic ischemic enterocolitis	557.1	K55.1
Ischemic stricture of intestine	557.1	K55.1
Mesenteric vascular insufficiency	557.1	K55.1
Abdominal angina	557.1	K55.1
Intestinal angina	557.1	K55.1
Mesenteric angina	557.1	K55.1
Superior mesenteric artery syndrome	557.1	K55.1
Chronic mesenteric arterial insufficiency syndrome	557.1	K55.1
Mesenteric vascular insufficiency syndrome	557.1	K55.1
Angina syndrome, abdominal	557.1	K55.1
Angina, intestinal	557.1	K55.1
Arteriomesenteric duodenal ileus	557.1	K55.1
Cast syndrome	557.1	K55.1
Duodenum, occlusion by superior mesenteric artery	557.1	K55.1
Duodenum, vascular compression	557.1	K55.1
Mesenteric duodenal compression syndrome	557.1	K55.1
Chronic ischemic colitis, enteritis, or enterocolitis	557.1	K55.1
Angina mesenteric	557.1	K55.1
Mesenteric artery syndrome (superior)	557.1	K55.1
Chronic intestinal ischemia	557.1	K55.1
Chronic intestinal vascular insufficiency	557.1	K55.1
Chronic mesenteric ischemia	557.1	K55.1
CMI (chronic mesenteric ischemia)	557.1	K55.1
Mesenteric vascular insufficiency, chronic	557.1	K55.1
Insufficient, vascular, mesenteric	557.1	K55.1

© 2021 Eberly LA et al. *JAMA Network Open*.

Ischemic stricture intestine	557.1	K55.1
SMAS (superior mesenteric artery syndrome)	557.1	K55.1
Stricture, intestine, ischemic	557.1	K55.1
Intestinal vascular insufficiency, chronic	557.1	K55.1
Mesenteric artery stenosis	557.1	K55.1
Mesenteric artery insufficiency	557	K55.1
Arteriosclerosis of mesenteric artery	557.1	K55.1
Mesenteric ischemia, chronic	557.1	K55.1
Arteriosclerosis, mesenteric artery	557.1	K55.1
Chronic vascular disorders of intestine	557.1	K55.1
Superior mesenteric artery atherosclerosis	557.1	K55.1
Atherosclerosis of superior mesenteric artery	557.1	K55.1
Chronic mesenteric insufficiency	557.1	K55.1
Chronic intermittent arteriomesenteric occlusion of the duodenum syndrome	557.1	K55.1
Chronic vascular disorder of intestine	557.1	K55.1
Chronic thrombosis of mesenteric vein	557.1	K55.1
Stenosis of inferior mesenteric artery	557.1	K55.1
Chronic vascular disorders of intestine		K55.1
Other vascular disorders of intestine	557.9	K55.8
Unspecified vascular insufficiency of intestine	557.9	K55.9
Vascular insufficiency of intestine	557.9	K55.9
Ischemic bowel disease	557.9	K55.9
Ischemic enterocolitis	557.9	K55.9
Alimentary tract pain due to vascular insufficiency	557.9	K55.9
Acute ischemic enterocolitis	557	K55.9
Colitis, ischemic	557.9	K55.9
Ischemic colitis	557.9	K55.9
Mesenteric vascular insufficiency, acute	557	K55.9

© 2021 Eberly LA et al. *JAMA Network Open*.

Intestinal ischemia	557.9	K55.9
Ischemia, bowel	557.9	K55.9
Ischemia, intestine	557.9	K55.9
Enteritis, ischemic	557.9	K55.9
Ischemic enteritis	557.9	K55.9
Alimentary pain due to vascular insufficiency	557.9	K55.9
Ischemic colitis, enteritis, or enterocolitis	557.9	K55.9
Ischemic disease of gut	557.9	K55.9
Colonic ischemia	557.9	K55.9
Ischemic colon	557.9	K55.9
Vascular disorder of intestine	569.9	K55.9
Intestinal vascular insufficiency	557.9	K55.9
Ischemic bowel syndrome	557.9	K55.9
Digestive tract pain due to vascular insufficiency	557.9	K55.9
Ischemia of large intestine	557.9	K55.9
Large bowel ischemia	557.9	K55.9
Ischemia of small intestine	557.9	K55.9
Small bowel ischemia	557.9	K55.9
Mesenteric ischemia	557.9	K55.9
Acute vascular disorder of intestine	569.9	K55.9
Acute mesenteric insufficiency	557	K55.9
Vascular disorder of intestine, unspecified	569.9	K55.9
Acute vascular disorders of intestine	569.9	K55.9
Ischemic gastroenteritis	557.9	K55.9
Vascular disorder of intestine, unspecified		K55.9
Small bowel ischemia related to hypovolemia	557.9, 276.52	K55.9, E86.1
Maternal coronary artery disease complicating pregnancy, first trimester	648.63, 414.00	O99.411, I25.10
Maternal coronary artery disease, first trimester	674.03	O99.411, I25.10
Maternal coronary artery disease complicating pregnancy in first trimester	648.63, 414.00	O99.411, I25.10

© 2021 Eberly LA et al. *JAMA Network Open*.

Maternal coronary artery disease affecting pregnancy, antepartum, first trimester	648.63, 414.00	O99.411, I25.10
Coronary artery disease in mother affecting pregnancy in first trimester, antepartum	648.63, 414.00	O99.411, I25.10
Cerebrovascular disorder occurring in pregnancy, childbirth, or the puerperium, first trimester	674.03	O99.411, I67.9
Cerebrovascular disorder, obstetric, delivered, first trimester	674.03	O99.411, I67.9
Cerebrovascular disorder, obstetric, antepartum, first trimester	674.03	O99.411, I67.9
Cerebrovascular disorder, obstetric, first trimester	674.03	O99.411, I67.9
Antepartum cerebrovascular disorder, first trimester	674.03	O99.411, I67.9
Cerebrovascular disorder, antepartum, first trimester	674.03	O99.411, I67.9
Cerebrovascular disorder in pregnancy, childbirth, or the puerperium, first trimester	674.03	O99.411, I67.9
Cerebrovascular disorder, with delivery, first trimester	674.03	O99.411, I67.9
Cerebrovascular disorder, with delivery, with or without mention of antepartum condition, first trimester	674.03	O99.411, I67.9
Antepartum cerebrovascular disorder in first trimester	674.03	O99.411, I67.9
Cerebrovascular disorder affecting pregnancy in first trimester	674.03, 437.9	O99.411, I67.9
Cerebrovascular disorder affecting pregnancy, first trimester	674.03, 437.9	O99.411, I67.9
Maternal coronary artery disease, second trimester	674.03	O99.412, I25.10

© 2021 Eberly LA et al. *JAMA Network Open*.

Maternal coronary artery disease complicating pregnancy, second trimester	648.63, 414.00	O99.412, I25.10
Maternal coronary artery disease complicating pregnancy in second trimester	648.63, 414.00	O99.412, I25.10
Maternal coronary artery disease affecting pregnancy in second trimester, antepartum	648.63, 414.00	O99.412, I25.10
Maternal coronary artery disease affecting pregnancy, antepartum, second trimester	648.63, 414.00	O99.412, I25.10
Cerebrovascular disorder occurring in pregnancy, childbirth, or the puerperium, second trimester	674.03	O99.412, I67.9
Cerebrovascular disorder, obstetric, antepartum, second trimester	674.03	O99.412, I67.9
Antepartum cerebrovascular disorder, second trimester	674.03	O99.412, I67.9
Cerebrovascular disorder, with delivery, second trimester	674.03	O99.412, I67.9
Cerebrovascular disorder, obstetric, second trimester	674.03	O99.412, I67.9
Cerebrovascular disorder in pregnancy, childbirth, or the puerperium, second trimester	674.03	O99.412, I67.9
Cerebrovascular disorder, antepartum, second trimester	674.03	O99.412, I67.9
Cerebrovascular disorder, with delivery, with or without mention of antepartum condition, second trimester	674.03	O99.412, I67.9
Cerebrovascular disorder, obstetric, delivered, second trimester	674.03	O99.412, I67.9
Antepartum cerebrovascular disorder in second trimester	674.03	O99.412, I67.9
Cerebrovascular disorder affecting pregnancy in second trimester	674.03, 437.9	O99.412, I67.9

© 2021 Eberly LA et al. *JAMA Network Open*.

Cerebrovascular disorder affecting pregnancy, second trimester	674.03, 437.9	O99.412, I67.9
Maternal coronary artery disease, third trimester	674.03	O99.413, I25.10
Maternal coronary artery disease complicating pregnancy, third trimester	648.63, 414.00	O99.413, I25.10
Maternal coronary artery disease complicating pregnancy in third trimester	648.63, 414.00	O99.413, I25.10
Maternal coronary artery disease affecting pregnancy in third trimester, antepartum	648.63, 414.00	O99.413, I25.10
Maternal coronary artery disease affecting pregnancy, antepartum, third trimester	648.63, 414.00	O99.413, I25.10
Antepartum cerebrovascular disorder, third trimester	674.03	O99.413, I67.9
Cerebrovascular disorder in pregnancy, childbirth, or the puerperium, third trimester	674.03	O99.413, I67.9
Cerebrovascular disorder, with delivery, third trimester	674.03	O99.413, I67.9
Cerebrovascular disorder, with delivery, with or without mention of antepartum condition, third trimester	674.03	O99.413, I67.9
Cerebrovascular disorder, antepartum, third trimester	674.03	O99.413, I67.9
Cerebrovascular disorder, obstetric, antepartum, third trimester	674.03	O99.413, I67.9
Cerebrovascular disorder, obstetric, third trimester	674.03	O99.413, I67.9
Cerebrovascular disorder, obstetric, delivered, third trimester	674.03	O99.413, I67.9
Cerebrovascular disorder occurring in pregnancy, childbirth, or the puerperium, third trimester	674.03	O99.413, I67.9

© 2021 Eberly LA et al. *JAMA Network Open*.

Antepartum cerebrovascular disorder in third trimester	674.03	O99.413, I67.9
Cerebrovascular disorder affecting pregnancy in third trimester	674.03, 437.9	O99.413, I67.9
Cerebrovascular disorder affecting pregnancy, third trimester	674.03, 437.9	O99.413, I67.9
Maternal coronary artery disease complicating pregnancy	648.60, 414.00	O99.419, I25.10
Maternal coronary artery disease, unspecified trimester	648.60, 414.00	O99.419, I25.10
Maternal coronary artery disease complicating pregnancy, unspecified trimester	648.63, 414.00	O99.419, I25.10
Maternal coronary artery disease affecting pregnancy, antepartum	648.63, 414.00	O99.419, I25.10
Maternal coronary artery disease complicating pregnancy, antepartum	648.63, 414.00	O99.419, I25.10
Maternal coronary artery disease affecting pregnancy, antepartum, unspecified trimester	648.63, 414.00	O99.419, I25.10
Idiopathic ischemic cerebrovascular accident occurring in prenatal-perinatal period	674	O99.419, I63.9
Idiopathic ischemic stroke occurring in prenatal-perinatal period	674	O99.419, I63.9
Cerebrovascular disorder occurring in pregnancy, childbirth, or the puerperium, unspecified as to episode of care(674.00)	674	O99.419, I67.9
Cerebrovascular disorder, antepartum(674.03)	674.03	O99.419, I67.9
Cerebrovascular disorder in pregnancy, childbirth, or the puerperium	674.00, 437.9	O99.419, I67.9
Antepartum cerebrovascular disorder	674.03, 437.9	O99.419, I67.9

© 2021 Eberly LA et al. *JAMA Network Open*.

Cerebrovascular disorder, obstetric, antepartum	674.03, 437.9	O99.419, I67.9
Cerebrovascular disorder, obstetric	674.00, 437.9	O99.419, I67.9
Cerebrovascular disorder occurring in pregnancy, childbirth, or the puerperium	674.00, 437.9	O99.419, I67.9
Antepartum cerebrovascular disorder, unspecified trimester	674.03, 437.9	O99.419, I67.9
Cerebrovascular disorder occurring in pregnancy, childbirth, or the puerperium, unspecified trimester	674	O99.419, I67.9
Cerebrovascular disorder, obstetric, antepartum, unspecified trimester	674.03, 437.9	O99.419, I67.9
Cerebrovascular disorder, with delivery, unspecified trimester	674.01	O99.419, I67.9
Cerebrovascular disorder in pregnancy, childbirth, or the puerperium, unspecified trimester	674	O99.419, I67.9
Cerebrovascular disorder, obstetric, unspecified trimester	674	O99.419, I67.9
Cerebrovascular disorder, with delivery, with or without mention of antepartum condition, unspecified trimester	674.01	O99.419, I67.9
Cerebrovascular disorder, obstetric, delivered, unspecified trimester	674.01	O99.419, I67.9
Cerebrovascular disorder, antepartum, unspecified trimester	674.03, 437.9	O99.419, I67.9
Cerebrovascular disorder occurring in pregnancy, childbirth, or the puerperium, unspecified as to episode of care	674.00, 437.9	O99.419, I67.9
Cerebrovascular disorder, antepartum	674.03, 437.9	O99.419, I67.9
Cerebrovascular disorder affecting pregnancy	674.00, 437.9	O99.419, I67.9
Cerebrovascular disorder affecting pregnancy, unspecified trimester	674.03, 437.8	O99.419, I67.9

© 2021 Eberly LA et al. *JAMA Network Open*.

Cerebrovascular disorder affecting pregnancy, antepartum	674.03, 437.8	O99.419, I67.9
Cerebrovascular disorder, with delivery, with or without mention of antepartum condition	674.01	O99.42, I67.9
Cerebrovascular disorder, with delivery	674.01	O99.42, I67.9
Cerebrovascular disorder, obstetric, delivered	674.01	O99.42, I67.9
Cerebrovascular disorder, with delivery, with mention of postpartum complication	674.02	O99.43, I67.9
Cerebrovascular disorder, postpartum(674.04)	674.04	O99.43, I67.9
Cerebrovascular disorder in the puerperium	674.04	O99.43, I67.9
Cerebrovascular disorder, with delivery, with postpartum complication	674.02	O99.43, I67.9
Puerperal cerebrovascular disorder with postnatal complication	674.04	O99.43, I67.9
Cerebrovascular disorder, obstetric, delivered/postpartum complication	674.02	O99.43, I67.9
Cerebrovascular disorder, obstetric, postpartum condition	674.04	O99.43, I67.9
Cerebrovascular disorder in puerperium	674.04	O99.43, I67.9
Cerebrovascular disorder, postpartum	674.04, 437.9	O99.43, I67.9
Cerebrovascular disorders in the puerperium	674.04	O99.43, I67.9
Puerperal cerebrovascular disorder with antenatal complication	674.04	O99.43, I67.9
Cerebrovascular disorder during puerperium	674.04	O99.43, I67.9
Cerebrovascular disease-delivery w/ postpartum complication	674.02	O99.43, I67.9
Cerebrovascular disorder, postpartum	674.04	O99.43, I67.9
Atherosclerosis, deafness, diabetes, epilepsy, and nephropathy syndrome	759.89, 440.9, 593.9, 345.90, 389.9, 250.40	Q87.89, I70.90, G40.909, H91.90, N28.9, E11.21

© 2021 Eberly LA et al. *JAMA Network Open*.

Atherosclerosis-deafness-diabetes-epilepsy-nephropathy syndrome	759.89, 440.9, 593.9, 345.90, 389.9, 250.40	Q87.89, I70.90, G40.909, H91.90, N28.9, E11.21
Feigenbaum-Bergeron-Richardson syndrome	759.89, 440.9, 389.9, 345.90, 593.9, 250.40	Q87.89, I70.90, H91.90, G40.909, N28.9, E11.21
Poor arterial perfusion of leg	785.9	R09.89
Poor perfusion of leg	785.9	R09.89
Poor arterial perfusion of lower extremity	785.9	R09.89
Suspected cerebrovascular accident	785.9	R09.89
Unknown when suspected stoke patient was last well	785.9	R09.89
Suspected stroke patient last known to be well 3-4.5 hours ago	785.9	R09.89
Suspected stroke patient last known to be well less than 2 hours ago	785.9	R09.89
Suspected stroke patient last known to be well 2-3 hours ago	785.9	R09.89
Suspected stroke patient last known to be well more than 2 hours ago	785.9	R09.89
Suspected cerebrovascular accident (CVA)	785.9	R09.89
Unknown when suspected stroke patient was last well	785.9	R09.89
Suspected stroke patient last known to be well 2 to 3 hours ago	785.9	R09.89
Suspected stroke patient last known to be well 3 to 4.5 hours ago	785.9	R09.89
Alteration in skin integrity related to peripheral vascular disease	782.9, 443.9	R23.8, I73.9
Lateral ST segment elevation	794.31	R94.31
Aortic dissection following procedure	997.79, 441.00	T81.718A, I71.00
Aortic dissection following procedure, initial encounter	997.79, 441.00	T81.718A, I71.00
Aortic dissection following procedure, subsequent encounter	V58.89	T81.718D, I71.00
Aortic dissection following procedure, sequela	909.3	T81.718S, I71.00

© 2021 Eberly LA et al. *JAMA Network Open*.

Coronary stent restenosis due to progression of disease	414.00, V45.82	T82.855A, I25.10
Acute deep venous thrombosis of upper extremity after CABG procedure	453.82, 414.04	T82.868A, I82.629, Z95.1
Acute deep vein thrombosis of upper extremity following coronary artery bypass graft procedure	453.82, 414.04	T82.868A, I82.629, Z95.1
Acute deep vein thrombosis (DVT) of upper extremity following coronary artery bypass graft procedure	453.82, 414.04	T82.868A, I82.629, Z95.1
Stenosis of renal artery in transplanted kidney	440.1	T86.19, I70.1
Renal artery stenosis, transplant	440.1	T86.19, I70.1
Atherosclerosis of renal artery of transplanted kidney	996.81, 440.1	T86.19, I70.1
Renal artery atherosclerosis of kidney transplant	996.81, 440.1	T86.19, I70.1
Non-flow-limiting renal artery stenosis of transplanted kidney	996.81, 440.1	T86.19, I70.1
Renal artery stenosis, non-flow-limiting, of kidney transplant	996.81, 440.1	T86.19, I70.1
Stenosis of one of two renal arteries of transplanted kidney	996.81, 440.1	T86.19, I70.1
Renal artery stenosis in 1 of 2 vessels of kidney transplant	996.81, 440.1	T86.19, I70.1
Renal artery stenosis in hilum of donor kidney at time of transplant	996.81, 440.1	T86.19, I70.1
Stenosis of pancreatic artery of transplanted pancreas	996.86, 447.1	T86.898, I77.1
Pancreas artery stenosis of pancreas transplant	996.86, 447.1	T86.898, I77.1
Intragraft arterial stenosis of transplanted pancreas	996.74, 996.86, E878.0, 447.1	T86.898, I77.1
Pre-operative cardiovascular examination, unstable angina	411.1, V72.81	Z01.810, I20.0

© 2021 Eberly LA et al. *JAMA Network Open*.

Pre-operative cardiovascular examination, class IV angina	413.9, V72.81	Z01.810, I20.9
Class IV angina, pre-operative cardiovascular examination	413.9, V72.81	Z01.810, I20.9
Pre-operative cardiovascular examination, recent MI	V72.81, 410.92	Z01.810, I21.9
Pre-operative cardiovascular examination, recent myocardial infarction	V72.81, 410.92	Z01.810, I21.9
Pre-operative cardiovascular examination, myocardial ischemia	414.8, V72.81	Z01.810, I25.9
Myocardial ischemia, pre-operative cardiovascular examination	414.8, V72.81	Z01.810, I25.9
F/u of acute inferior myocardial infarction	V67.59, 412	Z09, I25.2
Follow-up of acute inferior myocardial infarction	V67.59, 412	Z09, I25.2
F/u of inferior myocardial infarction	V67.59, 412	Z09, I25.2
Follow-up of inferior myocardial infarction	V67.59, 412	Z09, I25.2
Encounter for follow-up of acute inferior myocardial infarction	V67.59, 412	Z09, I25.2
F/u of acute lateral myocardial infarction	V67.59, 412	Z09, I25.2
Follow-up of acute lateral myocardial infarction	V67.59, 412	Z09, I25.2
F/u of lateral myocardial infarction	V67.59, 412	Z09, I25.2
Follow-up of lateral myocardial infarction	V67.59, 412	Z09, I25.2
F/u of acute posterior myocardial infarction	V67.59, 412	Z09, I25.2
Follow-up of acute posterior myocardial infarction	V67.59, 412	Z09, I25.2
F/u of posterior myocardial infarction	V67.59, 412	Z09, I25.2
Follow-up of posterior myocardial infarction	V67.59, 412	Z09, I25.2
Encounter for follow-up of acute lateral myocardial infarction	V67.59, 412	Z09, I25.2

© 2021 Eberly LA et al. *JAMA Network Open*.

Encounter for follow-up of acute posterior myocardial infarction	V67.59, 412	Z09, I25.2
Follow-up of acute heart attack	V67.59, 412	Z09, I25.2
Follow-up of heart attack	V67.59, 412	Z09, I25.2
Follow-up of acute myocardial infarction	V67.59, 412	Z09, I25.2
Encounter for follow-up of acute myocardial infarction	V67.59, 412	Z09, I25.2
F/u of anterior myocardial infarction	410.12	Z09, I25.2
Follow-up of anterior myocardial infarction	410.12	Z09, I25.2
Encounter for follow-up of myocardial infarction of anterior wall	410.12	Z09, I25.2
Cerebrovascular disease consultation	V65.8, 437.9	Z71.89, I67.9
Cerebellar cerebrovascular accident without late effect	V12.54	Z86.73
Cerebellar stroke without late effect	V12.54	Z86.73
Status post multiple cerebral infarctions	V12.54	Z86.73
Cerebellar cerebrovascular accident (CVA) without late effect	V12.54	Z86.73
Peripheral vascular angioplasty status with implants and grafts	V43.4	Z95.820
S/P peripheral artery angioplasty with stent placement	V45.89	Z95.820
Status post peripheral artery angioplasty with insertion of stent	V45.89	Z95.820
Peripheral vascular angioplasty status with implants and grafts		Z95.820
Blood vessel replaced by other means	V43.4	Z95.828
Personal history of extremity bypass graft	V45.89	Z95.828
Status post aortobifemoral bypass surgery	V45.89	Z95.828
S/P aortobifemoral bypass surgery	V45.89	Z95.828
Status post femoral-popliteal bypass surgery	V45.89	Z95.828

© 2021 Eberly LA et al. *JAMA Network Open*.

S/P femoral-popliteal bypass surgery	V45.89	Z95.828
Status post ascending aortic replacement	V43.4	Z95.828
S/P ascending aortic replacement	V43.4	Z95.828
Status post bypass graft of extremity	V45.89	Z95.828
Status post aortic bifurcation bypass graft	V45.89	Z95.828
S/P aortic bifurcation bypass graft	V45.89	Z95.828
Status post insertion of iliac artery stent	V45.89	Z95.828
S/P insertion of iliac artery stent	V45.89	Z95.828
History of blood vessel replacement	V43.4	Z95.828
Status post femoropopliteal bypass surgery	V45.89	Z95.828
S/P aorto-bifemoral bypass surgery	V45.89	Z95.828
History of aorta-iliac-femoral bypass	V15.1	Z95.828
History of aortoiliofemoral vascular bypass	V15.1	Z95.828
S/P bypass graft of extremity	V45.89	Z95.828
Personal hx of extremity bypass graft	V45.89	Z95.828
History of extremity bypass graft	V45.89	Z95.828
Hx of extremity bypass graft	V45.89	Z95.828
S/P femoropopliteal bypass surgery	V45.89	Z95.828
History of aorto-femoral bypass	V15.1	Z95.828
History of arterial bypass of lower limb	V45.89	Z95.828
History of endovascular stent graft for abdominal aortic aneurysm	V43.4	Z95.828
Hx of endovascular stent graft for abdominal aortic aneurysm	V43.4	Z95.828
Presence of stent of bypass graft	V45.89	Z95.828
Presence of bypass graft stent	V45.89	Z95.828
H/O aorta-iliac-femoral bypass	V15.1	Z95.828
Hx of aorta-iliac-femoral bypass	V15.1	Z95.828
H/O extremity bypass graft	V45.89	Z95.828
H/O aorto-femoral bypass	V15.1	Z95.828
Hx of aorto-femoral bypass	V15.1	Z95.828
H/O arterial bypass of lower limb	V45.89	Z95.828
Hx of arterial bypass of lower limb	V45.89	Z95.828

© 2021 Eberly LA et al. *JAMA Network Open*.

H/O endovascular stent graft for abdominal aortic aneurysm	V43.4	Z95.828
History of repair of aneurysm of abdominal aorta using endovascular stent graft	V43.4	Z95.828
Status post vascular bypass	V45.89	Z95.828
S/P vascular bypass	V45.89	Z95.828
History of intravascular stent placement	V45.89	Z95.828
Presence of arterial stent	V49.89	Z95.828
Presence of stent in artery	V49.89	Z95.828
Surgically constructed arteriovenous graft	V45.89	Z95.828
History of arterial bypass of lower extremity	V45.89	Z95.828
Status post femorofemoral bypass surgery	V45.89	Z95.828
Presence of other vascular implants and grafts	V43.4	Z95.828
S/P femoral-femoral bypass surgery	V45.89	Z95.828
History of endovascular stent graft for abdominal aortic aneurysm (AAA)	V43.4	Z95.828
S/P repair of abdominal aortic aneurysm using bifurcation graft	V45.89	Z95.828, Z86.79
S/P AAA repair using bifurcation graft	V45.89	Z95.828, Z86.79
Status post repair of abdominal aortic aneurysm using bifurcation graft	V45.89	Z95.828, Z86.79
Status post repair of abdominal aortic aneurysm (AAA) using bifurcation graft	V45.89	Z95.828, Z86.79
Status post angioplasty with stent	V45.89	Z95.9
S/P angioplasty with stent	V45.89	Z95.9
Presence of cardiac and vascular implant and graft	V45.00	Z95.9
Central venous catheter in place, secondary permanent	V45.89	Z95.9
Status post arterial stent	V45.89	Z95.9

© 2021 Eberly LA et al. *JAMA Network Open*.

S/P arterial stent	V45.89	Z95.9
Presence of cardiac and vascular implant and graft, unspecified	V45.00	Z95.9
Presence of cardiac and vascular implant and graft, unspecified		Z95.9
Acute myocardial infarction of anterolateral wall	410	
Acute myocardial infarction of inferolateral wall	410.2	
Acute myocardial infarction of inferoposterior wall	410.3	
Acute myocardial infarction, true posterior wall infarction	410.6	
Acute myocardial infarction, subendocardial infarction	410.7	
Angina pectoris	413	
Coronary atherosclerosis	414	
Aneurysm and dissection of heart	414.1	
Occlusion and stenosis of basilar artery	433	
Occlusion and stenosis of carotid artery	433.1	
Occlusion and stenosis of vertebral artery	433.2	
Occlusion and stenosis of multiple and bilateral precerebral arteries	433.3	
Cerebral thrombosis	434	
Transient cerebral ischemia	435	
Aortic aneurysm and dissection	441	
Dissection of aorta	441	
Vascular insufficiency of intestine	557	
Late effects of cerebrovascular disease	438	
Occlusion of cerebral arteries	434	
Persistent migraine aura with cerebral infarction	346.6	
Acute myocardial infarction	410	
Atherosclerosis	440	

© 2021 Eberly LA et al. *JAMA Network Open*.

Persistent migraine aura with cerebral infarction, without mention of intractable migraine with status migrainosus(346.62)	346.62	G43.601, I63.9
Persistent migraine aura with cerebral infarction, status migrainosus	346.62, 434.91	G43.601, I63.9
Migraine aura, persistent, with cerebral infarct, status migrainosus	346.62, 434.91	G43.601, I63.9
Migraine aura, persistent, with cerebral infarct, status over 72 hours	346.62, 434.91	G43.601, I63.9
Persistent migraine aura with cerebral infarction and status migrainosus, not intractable	346.62, 434.91	G43.601, I63.9
Persistent migraine aura with cerebral infarction, without mention of intractable migraine with status migrainosus	346.62, 434.91	G43.601, I63.9
Persistent migraine aura with cerebral infarction and with status migrainosus	346.62, 434.91	G43.601, I63.9
Persistent migraine aura with cerebral infarction, not intractable, with status migrainosus	346.62, 434.91	G43.601, I63.9
Persistent migraine aura with cerebral infarction, without mention of intractable migraine without mention of status migrainosus(346.60)	346.6	G43.609, I63.9
Persistent migraine aura with cerebral infarction	346.60, 434.91	G43.609, I63.9
Persistent migraine aura with stroke	346.60, 434.91	G43.609, I63.9
Migraine aura, persistent, with cerebral infarction	346.60, 434.91	G43.609, I63.9
Persistent migraine aura with cerebral infarction and without status migrainosus, not intractable	346.60, 434.91	G43.609, I63.9
Persistent migraine aura with cerebral infarction, without mention of intractable	346.60, 434.91	G43.609, I63.9

© 2021 Eberly LA et al. *JAMA Network Open*.

migraine without mention of status migrainosus		
Persistent migraine aura with cerebral infarction without status migrainosus	346.6	G43.609, I63.9
Persistent migraine aura with cerebral infarction, not intractable, without status migrainosus	346.60, 434.91	G43.609, I63.9
Persistent migraine aura with cerebral infarction, with intractable migraine, so stated, without mention of status migrainosus(346.61)	346.61	G43.611, I63.9
Persistent migraine aura with cerebral infarction, with intractable migraine, so stated, with status migrainosus(346.63)	346.63	G43.611, I63.9
Persistent migraine aura, cerebral infarction, intractable, in status	346.63, 434.91	G43.611, I63.9
Migraine aura, persistent, with cerebral infarct, intractable, status	346.63, 434.91	G43.611, I63.9
Migraine aura, persistent, w/cerebral infarct, intractable, status >72hrs	346.63, 434.91	G43.611, I63.9
Intractable persistent migraine aura with cerebral infarction and status migrainosus	346.63, 434.91	G43.611, I63.9
Persistent migraine aura with cerebral infarction, with intractable migraine, so stated, with status migrainosus	346.63, 434.91	G43.611, I63.9
Persistent migraine aura with cerebral infarction, intractable, with status migrainosus	346.63, 434.91	G43.611, I63.9
Intractable persistent migraine aura with cerebral infarction	346.61, 434.91	G43.619, I63.9
Persistent migraine aura with cerebral infarct, intractable	346.61, 434.91	G43.619, I63.9

© 2021 Eberly LA et al. *JAMA Network Open*.

Migraine aura, persistent, with cerebral infarct, intractable	346.61, 434.91	G43.619, I63.9
Intractable persistent migraine aura with cerebral infarction and without status migrainosus	346.61, 434.91	G43.619, I63.9
Persistent migraine aura with cerebral infarction, with intractable migraine, so stated, without mention of status migrainosus	346.61, 434.91	G43.619, I63.9
Persistent migraine aura with cerebral infarction, with intractable migraine, so stated	346.61, 434.91	G43.619, I63.9
Persistent migraine aura with cerebral infarction, intractable, without status migrainosus	346.61, 434.91	G43.619, I63.9
Transient cerebral ischemic attacks and related syndromes		G45
Basilar artery syndrome	435	G45.0
Vertebral artery syndrome	435.1	G45.0
Vertebrobasilar artery syndrome	435.3	G45.0
Basilar artery insufficiency	435	G45.0
Vertebral artery insufficiency	435.1	G45.0
Vertebro-basilar artery syndrome	435.3	G45.0
Vertebrobasilar circulation transient ischemic attack	435.3	G45.0
Basilar artery ischemia	435	G45.0
Basilar insufficiency	435	G45.0
Vertebrobasilar ischemia	435.3	G45.0
Vertebral artery ischemia	435.1	G45.0
Vertebro basilar insufficiency	435.3	G45.0
Vertebro basilar ischemia	435.3	G45.0
Vertebrobasilar occlusive disease	433.2	G45.0
Vertebral-basilar artery occlusive syndrome	433.2	G45.0
Vertebral basilar insufficiency	435.3	G45.0

© 2021 Eberly LA et al. *JAMA Network Open*.

Vertebrobasilar insufficiency	435.3	G45.0
Insufficiency, arterial, basilar artery	435	G45.0
Insufficiency, arterial, vertebral artery	435.1	G45.0

© 2021 Eberly LA et al. *JAMA Network Open*.

eTable 2. Factors Associated with SGLT2 Inhibitor Use Among Patients with Heart Failure with Reduced Ejection Fraction on Multivariable Analysis

Characteristic	Adjusted OR	95% CI	p-value
**Age**	0.96	0.95-0.96	<.0001
**Female Gender**	0.83	0.75-0.92	<.0001
**Race (White as Ref)**			
Asian	1.27	0.96-1.65	0.10
Black	0.92	0.80-1.05	0.25
Latinx	1.06	0.91-1.22	0.46
**Region of Residence (West as Ref)**			
Midwest	1.00	0.86-1.17	0.96
Northeast	0.96	0.80-1.15	0.62
South	1.25	1.09-1.43	0.002
**Zip Code Linked Household Median Income ($<50K as Ref)**			
≥$100K	1.26	1.11-1.44	0.0004
$50K-$99,999K	1.21	1.08-1.34	0.0006
**Commercial Insurance (Medicare Advantage as Ref)**	1.66	1.47-1.87	<.0001
**Comorbidities**			
Dyslipidemia	1.57	1.31-1.89	<.0001
Myocardial infarction	1.05	0.95-1.17	0.32
Cerebrovascular disease	0.98	0.87-1.09	0.67
Chronic kidney disease	1.12	0.96-1.30	0.14
Obesity	1.26	1.14-1.40	<.0001
Hypertension	1.21	1.00-1.45	0.05
Peripheral vascular disease	1.15	1.01-1.29	0.02
No. of Elixhauser comorbidity	0.90	0.88-0.92	<.0001
**Heart failure Hospitalization within 12 Months (0 as Ref)**			
1	0.76	0.64-0.91	0.0002
>1	0.93	0.69-1.25	0.63
**No of Endocrinology Visits per 12 Months (0 as Ref)**			
1	2.01	1.70-2.37	<.0001
>1	2.85	2.47-3.28	<.0001
**No. of Cardiology Visits per 12 Months (0 as Ref)**			
1	1.17	1.02-1.34	0.03
>1	1.26	1.12-1.42	<.0001
**Metformin Use**	1.85	1.68-2.04	<.0001
**Insulin Use**	1.51	1.36-1.67	<.0001

© 2021 Eberly LA et al. *JAMA Network Open*.

eTable 3. Factors Associated with SGLT2 Inhibitor Use Among Patients with Atherosclerotic Cardiovascular Disease on Multivariable Analysis

Characteristic	Adjusted OR	95% CI	p-value
**Age**	0.97	0.97-0.97	<.0001
**Female Gender**	0.83	0.81-0.85	<.0001
**Race (White as Ref)**			
Asian	1.06	1.00-1.12	0.04
Black	0.84	0.81-0.87	<.0001
Latinx	1.05	1.02-1.08	0.004
**Region of Residence (West as Ref)**			
Midwest	1.00	0.96-1.04	0.87
Northeast	0.96	0.92-1.00	0.06
South	1.25	1.21-1.30	<.0001
**Zip-code Linked Household Median Income ($<50K as Ref)**			
≥$100K	1.08	1.05-1.12	<.0001
$50K-$99,999K	1.05	1.02-1.08	<.0001
**Commercial Insurance (Medicare Advantage as Ref)**	2.17	2.12-2.22	<.0001
**Comorbidities**			
Dyslipidemia	1.61	1.56-1.65	<.0001
Myocardial infarction	1.00	0.97-1.04	0.84
Cerebrovascular disease	0.98	0.95-1.00	0.09
Chronic kidney disease	1.03	0.99-1.07	0.14
Obesity	1.33	1.31-1.36	<.0001
Hypertension	1.49	1.45-1.53	<.0001
Peripheral vascular disease	1.04	1.01-1.07	0.03
HFrEF	0.85	0.79-0.91	<.0001
HFpEF	0.83	0.77-0.89	<.0001
No. of Elixhauser comorbidity	0.90	0.89-0.90	<.0001
**No of Endocrinology Visits per 12 Months (0 as Ref)**			
1	2.06	1.99-2.12	<.0001
>1	2.84	2.76-2.92	<.0001
**No. of Cardiology Visits per 12 Months (0 as Ref)**			
1	1.19	1.16-1.22	<.0001
>1	1.15	1.11-1.18	<.0001
**Metformin Use**	1.55	1.52-1.58	<.0001
**Insulin Use**	1.57	1.53-1.60	<.0001

HFrEF-heart failure with reduced ejection fraction; HFpEF-heart failure with preserved ejection fraction

© 2021 Eberly LA et al. *JAMA Network Open*.

eTable 4. Factors associated with SGLT2i Use Among Patients with Chronic Kidney Disease on Multivariable Analysis

Characteristic	Adjusted OR	95% CI	p-value
**Age**	0.96	0.95-0.96	<.0001
**Female Gender**	0.85	0.81-0.90	<.0001
**Race (White as Ref)**			
Asian	1.42	1.27-1.60	<0.0001
Black	0.89	0.82-0.97	0.009
Latinx	1.23	1.15-1.32	<0.001
**Region of Residence (West as Ref)**			
Midwest	0.80	0.74-0.88	<0.0001
Northeast	0.89	0.80-1.00	0.04
South	1.12	1.05-1.20-	0.001
**Zip-Code Linked Household Median Income ($<50K as Ref)**			
≥$100K	1.15	1.07-1.24	0.0003
$50K-$99,999K	1.05	0.99-1.11	0.15
**Commercial Insurance (Medicare Advantage as Ref)**	1.72	1.60-1.85	<.0001
**Comorbidities**			
Dyslipidemia	1.60	1.44-1.78	<.0001
Myocardial infarction	1.01	0.93-1.11	0.77
Cerebrovascular disease	1.00	0.93-1.07	0.99
Obesity	1.39	1.34-1.48	<.0001
Hypertension	1.29	1.17-1.42	<.0001
Peripheral vascular disease	1.10	1.02-1.18	0.01
HFrEF	0.78	0.67-0.90	0.001
HFpEF	0.86	0.75-1.00	0.05
No. of Elixhauser comorbidity	0.89	0.87-0.90	<.0001
**No of Endocrinology Visits per 12 Months (0 as Ref)**			
1	1.67	1.50-1.85	<.0001
>1	2.31	2.12-2.51	<.0001
**No. of Cardiology Visits per 12 Months (0 as Ref)**			
1	1.22	1.13-1.31	<.0001
>1	1.25	1.16-1.35	<.0001
**Metformin Use**	1.64	1.56-1.73	<.0001
**Insulin Use**	1.76	1.65-1.86	<.0001

HFrEF-heart failure with reduced ejection fraction; HFpEF-heart failure with preserved ejection fraction

© 2021 Eberly LA et al. *JAMA Network Open*.

eTable 5. Factors associated with SGLT2 Inhibitor Use Among Patients on Metformin Therapy on Multivariable Analysis

Characteristic	Adjusted OR	95% CI	p-value
**Age**	0.97	0.97-0.97	<.0001
**Female Gender**	0.82	0.80-0.84	<.0001
**Race (White as Ref)**			
Asian	0.84	0.80-0.88	<0.001
Black	0.79	0.76-0.82	<.0001
Latinx	0.97	0.94-0.99	0.02
**Region of Residence (West as Ref)**			
Midwest	1.04	1.00-1.08	0.02
Northeast	0.98	0.94-1.02	0.37
South	1.23	1.19-1.27	<.0001
**Zip-code Linked Household Median Income ($<50K as Ref)**			
≥ $100K	1.13	1.10-1.17	<.0001
$50K-$99,999K	1.08	1.05-1.10	<0.001
**Commercial Insurance (Medicare Advantage as Ref)**	1.90	1.85-1.96	<.0001
**Comorbidities**			
Dyslipidemia	1.54	1.49-1.60	<.0001
Myocardial infarction	1.03	0.98-1.07	0.28
Cerebrovascular disease	0.97	0.94-1.00	0.09
Obesity	1.20	1.17-1.23	<.0001
Hypertension	1.34	1.30-1.38	<.0001
Peripheral vascular disease	1.02	0.99-1.06	0.23
HFrEF	0.85	0.78-.93	0.0005
HFpEF	0.81	0.74-0.90	<.0001
No. of Elixhauser comorbidity	0.93	0.92-0.94	<.0001
**No of Endocrinology Visits per 12 Months (0 as Ref)**			
1	2.11	2.03-2.20	<.0001
>1	3.19	3.08-3.30	<.0001
**No. of Cardiology Visits per 12 Months (0 as Ref)**			
1	1.16	1.12-1.19	<.0001
>1	1.17	1.13-1.22	<.0001
**Insulin Use**	1.59	1.55-1.63	<.0001

HFrEF-heart failure with reduced ejection fraction; HFpEF-heart failure with preserved ejection fraction

© 2021 Eberly LA et al. *JAMA Network Open*.
